# MCM10 and SLD-2/RECQL4 jointly activate the CMG helicase during metazoan DNA replication initiation

**DOI:** 10.1038/s44318-026-00915-9

**Published:** 2026-09-28

**Authors:** Remi Sonneville, Cecile Evrin, Jane Wright, He Huang, Yisui Xia, Karim P M Labib

**Affiliations:** 1 Department of Genome Integrity, Faculty of Life Sciences, University of Dundee, Dundee, DD1 5EH UK; 2 Guangdong Key Laboratory for Genome Stability & Disease Prevention, the Belt & Road Shenzhen-Almaty Joint Laboratory for Frontiers of Medical Science, Marshall Laboratory of Biomedical Engineering, School of Basic Medical Sciences, Shenzhen University Medical School, Shenzhen University, Shenzhen, 518055 China; 3 Shenzhen University General Hospital, Shenzhen University Medical School, Shenzhen University, Shenzhen, 518055 China

**Keywords:** DNA Replication, Recombination & Repair

## Abstract

Eukaryotic cells regulate the assembly and activation of the essential DNA helicase at the heart of the chromosome replication machinery, to ensure that the chromosomes are copied just once per cell cycle. The Mcm10 protein is essential for helicase activation in budding yeast, but an equivalent role for MCM10 orthologues in animal cells has not been explored. Moreover, complete deletion of the *mcm-10* gene is viable in the nematode *Caenorhabditis elegans*, suggesting the involvement of additional factors. Here we show that MCM-10 and a second factor called SLD-2 are recruited to chromatin after helicase assembly in the *C. elegans* early embryo and are partially redundant for helicase activation. Moreover, deletion of the *Mcm10* gene is viable in mouse embryonic stem cells, but causes synthetic lethality in the absence of RECQL4, which is the orthologue of SLD-2 in vertebrate species. Helicase activation is blocked in the combined absence of MCM10 and RECQL4, mirroring the situation in *C. elegans*. These findings indicate that metazoan helicase activation requires two conserved factors that are mutated in human disease.

## Synopsis

It has remained unclear if MCM10, essential for replicative helicase activation in yeast, plays a similar role in metazoans. This work shows that MCM-10 and SLD-2 are needed to activate CMG helicase during DNA replication initiation in the nematode *C. elegans*, as are their orthologs MCM10 and RECQL4 in mammalian cells.

- Activation of the CMG helicase during DNA replication initiation is delayed in *C. elegans* embryos that lack either MCM-10 or SLD-2.
- In the combined absence of MCM-10 and SLD-2, helicase activation, replisome assembly, and the initiation of DNA replication are blocked.
- MCM-10 and SLD-2 stimulate the activity of the CMG helicase in vitro, together with an additional initiation factor called DNSN-1.
- Activation of the CMG helicase during DNA replication initiation is delayed in mouse embryonic stem cells that lack either MCM10 or the RECQL4 orthologue of SLD-2.
- Helicase activation and replisome assembly during DNA replication initiation are blocked in the combined absence of MCM10 and RECQL4.

## Abstract (web summary)

Parallel action of worm and human MCM10 with a conserved second factor gives it a role in replicative helicase activation similar to the budding yeast homolog.

## Graphical abstract

**Figure:**
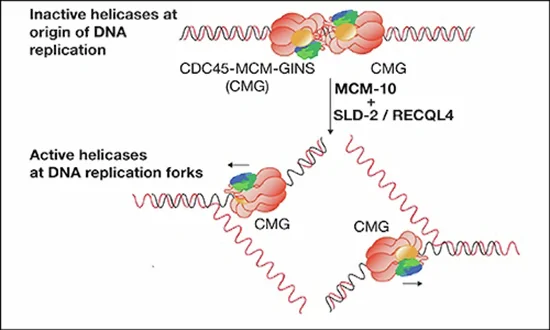

## Introduction

The proliferation of eukaryotic cells is dependent upon making a single copy of every chromosome during each cell cycle. The duplication of eukaryotic chromosomes is controlled by the regulated assembly and disassembly of the chromosome replication machinery, or replisome, to ensure that each chromosome is copied just once (Costa and Diffley, 2022). The replisome assembles around the 11-subunit DNA helicase known as CMG (CDC45, MCM2-7, GINS), which is built in situ at DNA replication origins in a two-step process that can only occur once per cell cycle. Once assembled, the nascent helicases are activated by a mechanism that remains poorly characterised in all species.

During the G1-phase of the cell cycle, two rings of the six MCM2-7 ATPases are loaded around double-stranded DNA (ds DNA) at replication origins, to form an MCM2-7 double-hexamer that lacks helicase activity (Frisbie and Bleichert, 2026; Stillman et al, 2025). Subsequently, when cells enter S-phase, the CDC45 protein and the 4-protein GINS complex are recruited to MCM2-7 double hexamers to form ‘head-to-head’ pairs of CMG helicase complexes, which initially still encircle ds DNA (Gambus et al, 2006; Lewis et al, 2022; Moyer et al, 2006).

Activation of the MCM2-7 ATPases within the double-CMG complex leads to melting of ds DNA within the central cavity of the MCM2-7 hexamer, and also to extrusion of one of the two parental DNA strands through a ‘gate’ in the MCM2-7 ring (Costa and Diffley, 2022), likely between the MCM2 and MCM5 subunits (Weekes et al, 2026). This produces two active CMG helicases that encircle opposite DNA strands at a pair of nascent DNA replication forks. The two helicases translocate along DNA in a 3’ to 5’ direction (Fu et al, 2011), bypassing each other and then moving away from the origin in opposite directions (Douglas et al, 2018; Georgescu et al, 2017). At the same time, each CMG helicase associates with a set of partner proteins to form a replisome that commences DNA synthesis (Gambus et al, 2006; Gambus et al, 2009; Sengupta et al, 2013).

The loading of MCM2-7 double hexamers around origin DNA has been studied extensively, aided by biochemical reconstitution and structural analysis of intermediates (Costa and Diffley, 2022; Evrin et al, 2009; Frisbie and Bleichert, 2026; Remus et al, 2009; Stillman et al, 2025), principally in the budding yeast *Saccharomyces cerevisiae* but more recently with purified human proteins (Weissmann et al, 2024; Wells et al, 2025; Yang et al, 2024). These studies identified a conserved set of MCM2-7 loading factors and also revealed surprising mechanistic diversity, both within an individual species and also between yeast and humans.

The conversion of MCM2-7 double hexamers into a pair of CMG helicase complexes has also been reconstituted with purified budding yeast proteins (Yeeles et al, 2015), and intermediates in the CMG assembly reaction have been studied by cryo-electron microscopy (Costa and Diffley, 2022; Puhringer et al, 2026). Cdc45 is coupled to MCM2-7 double hexamers by Sld3 (Deegan et al, 2016; Li et al, 2025), whereas GINS is recruited by a combination of Sld2, Dpb11 and DNA polymerase epsilon (Pol ε) that subsequently synthesises the leading strand at DNA replication forks (Muramatsu et al, 2010). These factors are all conserved in diverse eukaryotic species. However, recent work indicates important mechanistic differences and additional complexity during CMG assembly in animal species, for which biochemical reconstitution of CMG assembly has yet to be achieved. Studies in the nematode *Caenorhabditis elegans* (Xia et al, 2023), the frog *Xenopus laevis* (Hashimoto et al, 2023; Kingsley et al, 2023; Lim et al, 2023), and also in mouse and humans (Evrin et al, 2023; Lim et al, 2023) showed that a protein called DONSON is essential for the recruitment of GINS to MCM2-7 double hexamers during CMG assembly. In humans, DONSON is mutated in a form of microcephalic primordial dwarfism (Reynolds et al, 2017; Schulz et al, 2018) that is typically caused by mutations in CMG components or assembly factors (Bellelli and Boulton, 2021; Klingseisen and Jackson, 2011; Nielsen-Dandoroff et al, 2023). However, DONSON appears to have been lost during fungal evolution and so is not present in yeast cells.

In budding yeast, Mcm10 is essential for the activation of nascent CMG helicases both in vivo (van Deursen et al, 2012; Watase et al, 2012) and in vitro (Douglas et al, 2018). Similarly, Mcm10 is also required for CMG activation in the fission yeast, *Schizosaccharomyces pombe* (Kanke et al, 2012). However, studies of MCM10 orthologues in animal cells suggested the existence of other CMG activation factors. For example, depletion of more than 99% of MCM10 from *Xenopus laevis* egg extracts did not inhibit DNA replication in vitro (Chadha et al, 2016). Moreover, truncating mutations within *Drosophila* Mcm10 did not prevent the development of adult flies, though the resulting females had greatly reduced fertility (Reubens et al, 2015). Recently, worms lacking the first 96% of the coding sequence of *C. elegans mcm-10* were found to be viable and fertile (Xia et al, 2023), strongly indicating the existence of an MCM-10-independent pathway for CMG helicase activation in animal cells. Here, we take a multi-dimensional approach based on *C. elegans* early embryos and mammalian cells, to show that the orthologues of yeast Sld2 and Mcm10 are partially redundant for helicase activation but are dispensable for assembly of the CMG complex, during the initiation of chromosome replication in metazoa.

## Results

### CMG activation is delayed in the absence of MCM-10 or SLD-2 in the *C. elegans* early embryo

**Figure EV1:**
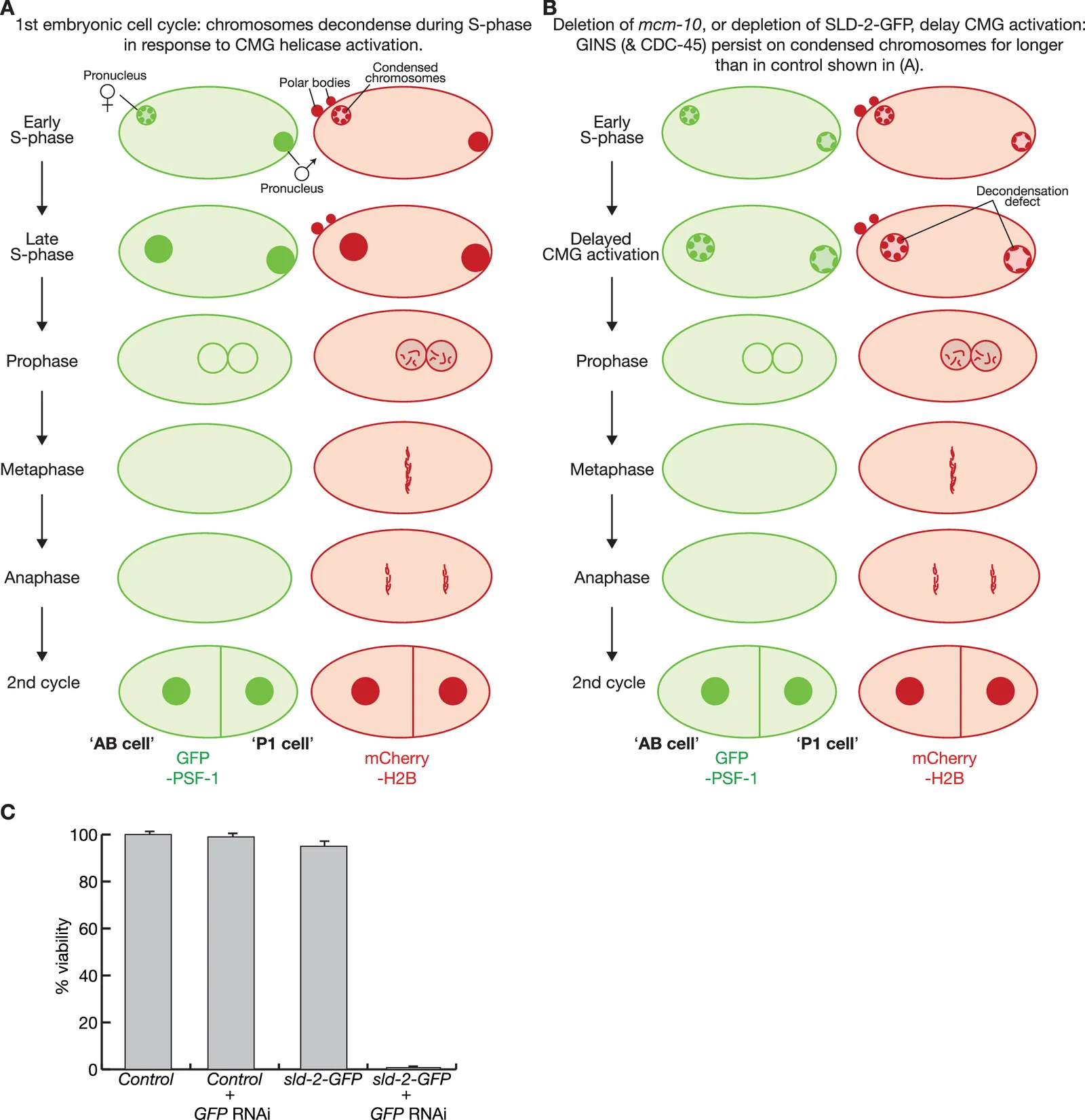
Depletion of SLD-2 is lethal in *C. elegans*. (**A**) Illustration of the first embryonic cell cycle of *C. elegans* expressing GFP-PSF-1 and mCherry-HistoneH2B. (**B**) Illustration of how chromosome decondensation is delayed in response to defects in CMG helicase activation in the first embryonic cell cycle of *mcm-10∆* worms, or in response to depletion of SLD-2. (**C**) Embryonic viability was measured as described in Methods, after treating control (wild type ‘N2’ worms) or *sld-2-GFP* worms with *GFP* RNAi as indicated. Mean values and standard deviations are presented from three independent experiments.

The first cell cycle of the *C. elegans* embryo provides a model system for studying the regulation of the metazoan CMG helicase in vivo (Sonneville et al, 2019; Sonneville et al, 2017; Sonneville et al, 2012; Xia et al, 2021; Xia et al, 2023). After fertilisation, DNA replication initiates in the female and male pronuclei on chromosomes that are still condensed after the second meiotic cell division (Fig. EV1A). The chromosomes then decondense rapidly during early S-phase, in response to DNA unwinding by the newly activated CMG helicase (Sonneville et al, 2015). Therefore, chromosome decondensation is delayed when CMG assembly is blocked by depletion of CMG components (Sonneville et al, 2015), or by depletion of CMG assembly factors such as DNSN-1 that is the worm orthologue of mammalian DONSON (Xia et al, 2023).

**Figure 1:**
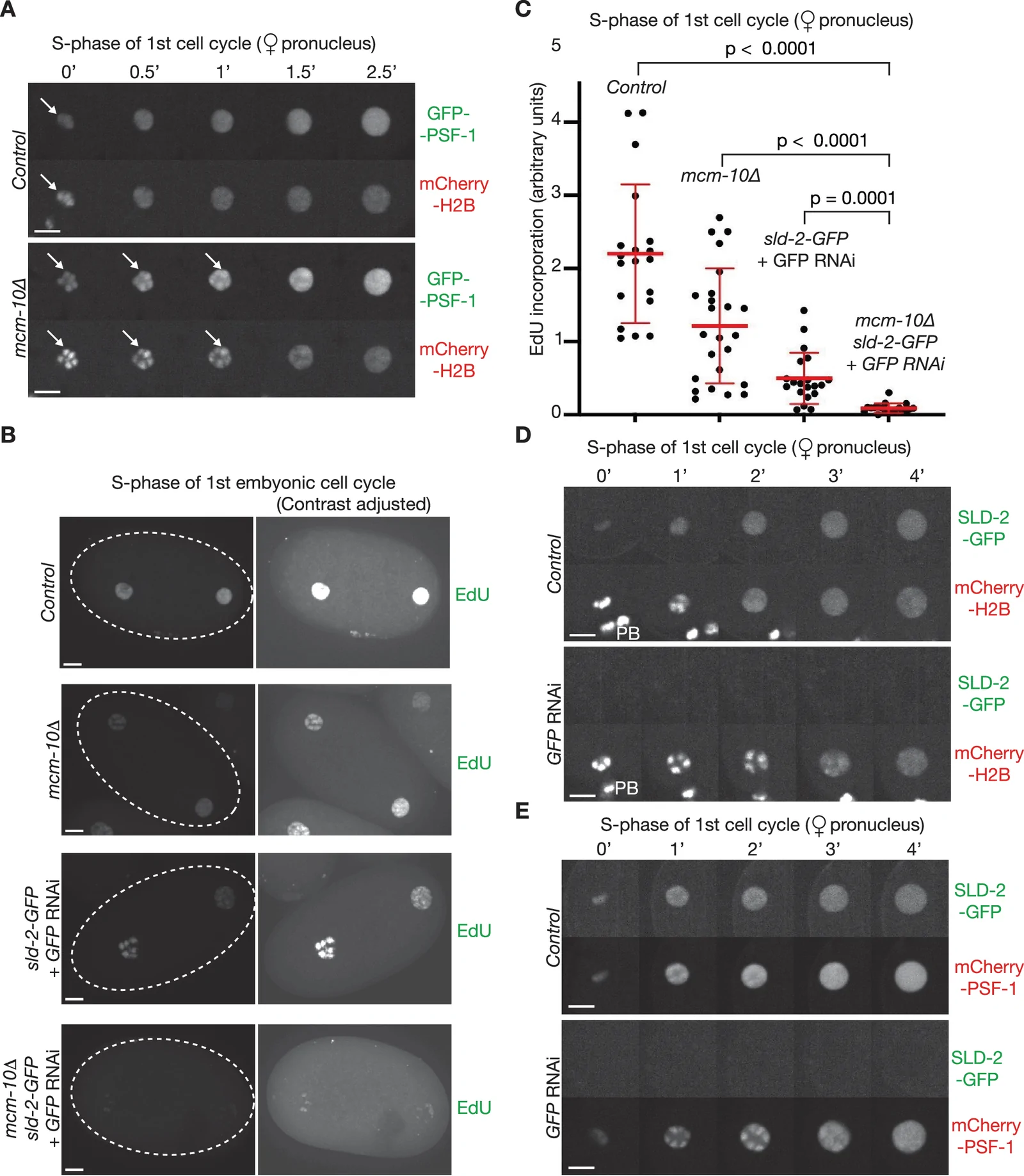
DNA replication is impaired after CMG helicase assembly in the absence of MCM-10 or SLD-2 in the *C. elegans* early embryo. (**A**) Chromosome decondensation was monitored for the indicated strains, upon entry into the first cell cycle of the *C. elegans* embryo. A sequence of images is presented, corresponding to the progression of the female pronucleus through early S-phase at the indicated times. Arrows denote the association of GFP-PSF-1 with condensed chromosomes, which persist for longer in the absence of MCM-10. (**B**) Embryos of the indicated genotypes, treated as indicated with *GFP* RNAi, were given a 10-min pulse of 20 mM EdU and then fixed, before detection of EdU as described in Methods. The right panels show higher contrast versions of the images in the left panels. (**C**) EdU incorporation from (**B**) was quantified as described in Methods. The mean values (red bars) and standard deviation (red error bars) are shown for 20 or more embryos. *P* values were generated via a one-way ANOVA followed by Dunnett’s multiple comparisons test. *P* = 0.00000005 for the comparison of control with *mcm-10∆ sld-2-GFP* + *GFP* RNAi, and 0.000001 for the comparison of *mcm-10∆* with *mcm-10∆ sld-2-GFP* + *GFP* RNAi. (**D**) Worms expressing SLD-2-GFP and mCherry-HistoneH2B were treated as indicated with *GFP* RNAi and then analysed by time-lapse video microscopy. PB polar body, located next to the female pronucleus. (**E**) Analogous experiment with worms expressing SLD-2-GFP and mCherry-PSF-1. The arrows indicate persistence of mCherry-PSF-1 on condensed chromosomes after depletion of GFP-SLD-2. All scalebars correspond to 5 µm. Videos of the embryos shown in this figure can be found in Movies EV1–EV6. Source data are available online for this figure.

In *mcm-10*∆ worms (Xia et al, 2023), chromosome decondensation was delayed during the S-phase of the first embryonic cell cycle (Fig. 1A, mCherry-H2B). Moreover, the GINS subunit PSF-1 was detected on condensed chromosomes throughout the period of the decondensation delay (Figs. 1A, *mcm-10*∆ and EV1B). Since the association of GINS with chromatin requires the prior recruitment of CDC-45 to double hexamers of MCM-2-7 (Xia et al, 2023), these data indicate that the CMG helicase assembles in *mcm-10*∆ worms but then remains inactive for an extended period, thereby delaying the initiation of DNA replication and chromosome decondensation. Correspondingly, DNA synthesis was found to be reduced at the start of the first cell cycle in *mcm-10*∆ compared to control, when embryos were treated with a short pulse of the nucleoside precursor 5-ethynyl-2’-deoxyuridine or EdU (Fig. 1B,C). Subsequently, chromosome replication is completed, and mitosis proceeds normally (Appendix Fig. S1A–C, *mcm-10*∆), and *mcm-10*∆ embryos produce viable adult worms (Xia et al, 2023), suggesting that other factors are subsequently able to activate the CMG helicase, despite the absence of MCM-10.

We previously showed that *mcm-10*∆ is synthetic lethal in combination with incomplete depletion by RNA interference (RNAi) of the essential DNA replication initiation factors SLD-2, DNSN-1 or MUS-101 that is the worm orthologue of yeast Dpb11 and mammalian TOPBP1 (Xia et al, 2023). Therefore, SLD-2, DNSN-1 and MUS-101 are all candidates for factors that contribute to CMG helicase activation in the absence of MCM-10. However, DNSN-1 and MUS-101 are essential for assembly of the CMG helicase (Xia et al, 2023), making it harder to address whether they also have a subsequent role in helicase activation. Until now, SLD-2 was also thought to be important for CMG assembly in *C. elegans* (Gaggioli et al, 2014; Xia et al, 2023), analogous to the role of yeast Sld2 (Muramatsu et al, 2010; Yeeles et al, 2015). However, previous studies of worm SLD-2 did not monitor CMG assembly during S-phase and instead relied on indirect proxies such as the level of chromatin-bound CMG during mitosis of the first embryonic cell cycle, following inactivation of the CDC-48_NPL-4_UFD-1 unfoldase that normally disassembles CMG during DNA replication termination (Sonneville et al, 2017).

To study the role of SLD-2 in assembly of the CMG helicase during the first embryonic cell cycle, we treated *sld-2-GFP* worms with *GFP* RNAi. In contrast to *sld-2* RNAi that depleted SLD-2-GFP incompletely (Xia et al, 2023), *GFP* RNAi led to efficient depletion of SLD-2-GFP (Fig. 1D,E) and caused embryonic lethality (Fig. EV1C), mirroring the lethal effects of deleting the *sld-2* gene (Xia et al, 2023). When embryos were treated with a short pulse of EdU at the start of S-phase, DNA synthesis was reduced about fourfold in the absence of SLD-2 compared to the control (Fig. 1B,C). In addition, chromosome decondensation was delayed when embryos lacking SLD-2 entered S-phase (Fig. 1D), and GINS was recruited to the condensed chromosomes during this period (Fig. 1E), mirroring the situation in *mcm-10*∆ worms (Fig. 1A). In contrast, GINS was not detected on chromatin when assembly of the CMG helicase was blocked and chromosome decondensation delayed by depletion of DNSN-1 (Xia et al, 2023). These data suggest that SLD-2 and MCM-10 are dispensable for CMG helicase assembly during the initiation of DNA replication in *C.elegans*. Instead, both MCM-10 and SLD-2 are important, though not individually essential, for activation of the CMG helicase.

Although DNA replication is delayed during the first embryonic cell cycle in the absence of SLD-2 (Fig. 1B,C), chromosome condensation and segregation in the subsequent mitosis proceed normally (Appendix Fig. S1A, *GFP* RNAi), indicating that chromosome replication is largely completed before the initiation of chromosome segregation. In the second cell cycle of embryos lacking SLD-2, mitosis also proceeded normally in the ‘P1 cell’ (Fig. EV1A,B; Appendix Fig. S1B, *GFP* RNAi), which is the precursor of the germline and has a longer cell cycle than the ‘AB cell’ that gives rise exclusively to somatic cells. However, chromosome condensation was defective, and chromosome segregation failed in the AB cell in all embryos lacking SLD-2 (Appendix Fig. S1C, *GFP* RNAi, *n* = 5), likely due to the shorter cell cycle of the AB cell that does not give enough time to complete replication, when CMG activation is delayed in the absence of SLD-2. These data explain why efficient depletion of SLD-2 leads to a complete loss of embryonic viability (Fig. EV1C), and deletion of the *sld-2* gene is lethal in *C. elegans* (Xia et al, 2023). In contrast, mitosis proceeds normally in the absence of MCM-10 (Appendix Fig. S1), indicating that the initiation of DNA replication is delayed more severely in the absence of SLD-2, compared to worms lacking MCM-10.

### MCM-10 and SLD-2 associate with the CMG helicase on chromatin during DNA replication initiation

**Figure 2:**
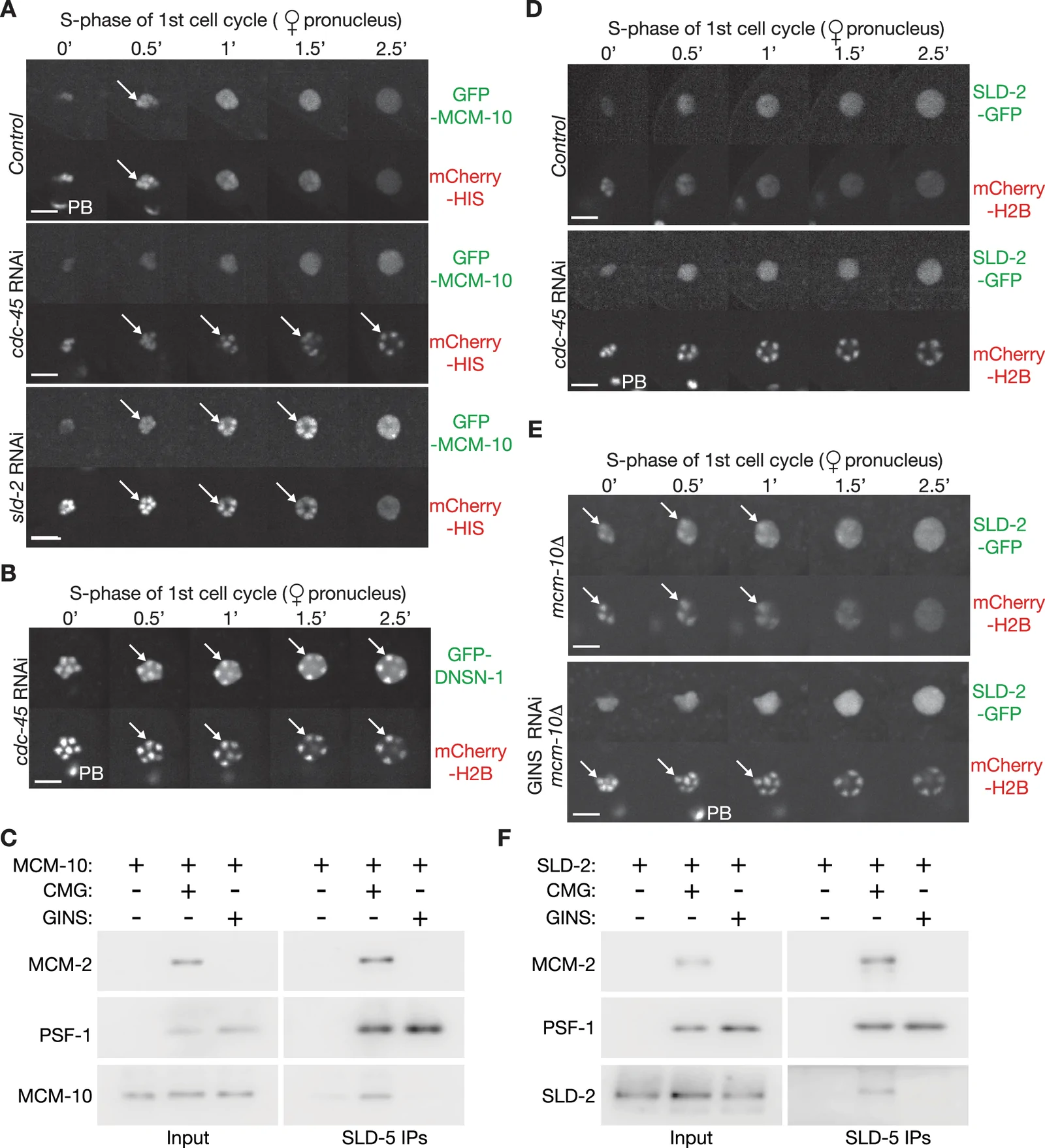
MCM-10 and SLD-2 are recruited to the CMG helicase during early S-phase in the *C. elegans* embryo. (**A**) Worms expressing GFP-MCM-10 and mCherry-HistoneH2B were treated with the indicated RNAi and then analysed by time-lapse video microscopy. Arrows indicate GFP-MCM-10 and mCherry-HistoneH2B on condensed chromosomes. (**B**) Worms expressing GFP-DNSN-1 and mCherry-HistoneH2B were treated with *cdc-45* RNAi and then analysed as above. Arrows indicate GFP-DNSN-1 and mCherry-HistoneH2B on condensed chromosomes. PB polar body. (**C**) Recombinant *C. elegans* MCM-10, CMG and GINS were mixed in vitro as indicated, before immunoprecipitation of the SLD-5 subunit of GINS and CMG (see Methods). The indicated proteins were detected by immunoblotting. (**D**) Worms expressing SLD-2-GFP and mCherry-HistoneH2B were treated as shown and then analysed as above. (**E**) *mcm-10∆* worms expressing SLD-2-GFP and mCherry-HistoneH2B were treated as shown with RNAi to the *sld-5* and psf-1 subunits of GINS, before analysis by time-lapse video microscopy. Arrows indicate SLD-2-GFP and mCherry-HistoneH2B on condensed chromosomes. (**F**) Recombinant *C. elegans* SLD-2, CMG and GINS (see Methods) were mixed and processed as in (**C**). All scalebars correspond to 5 µm. Videos of the embryos shown in this figure can be found in Movies EV7–EV14. Source data are available online for this figure.

Consistent with MCM-10 playing a role during CMG helicase activation in vivo, GFP-MCM-10 associated with chromatin during early S-phase (Fig. 2A, Control) and remained on chromatin when CMG activation and chromosome decondensation were delayed by depletion of SLD-2 (Fig. 2A, *sld-2* RNAi). Furthermore, chromatin association of MCM-10 was lost in the absence of CDC-45, and so was dependent upon assembly of the CMG helicase (Fig. 2A; Appendix Fig. S2). In contrast, the CMG assembly factor DNSN-1 remained on condensed chromosomes in the absence of CDC-45 (Fig. 2B), as reported previously (Xia et al, 2023), since DNSN-1 is part of a ‘pre-initiation complex’ that represents an intermediate en route to CMG assembly. These data indicate that MCM-10 is recruited to nascent CMG helicase complexes during the initiation of DNA replication in *C. elegans*, independently of SLD-2. Consistent with this view, MCM-10 binds directly to recombinant *C. elegans* CMG helicase complexes in vitro (Fig. 2C).

**Figure 3:**
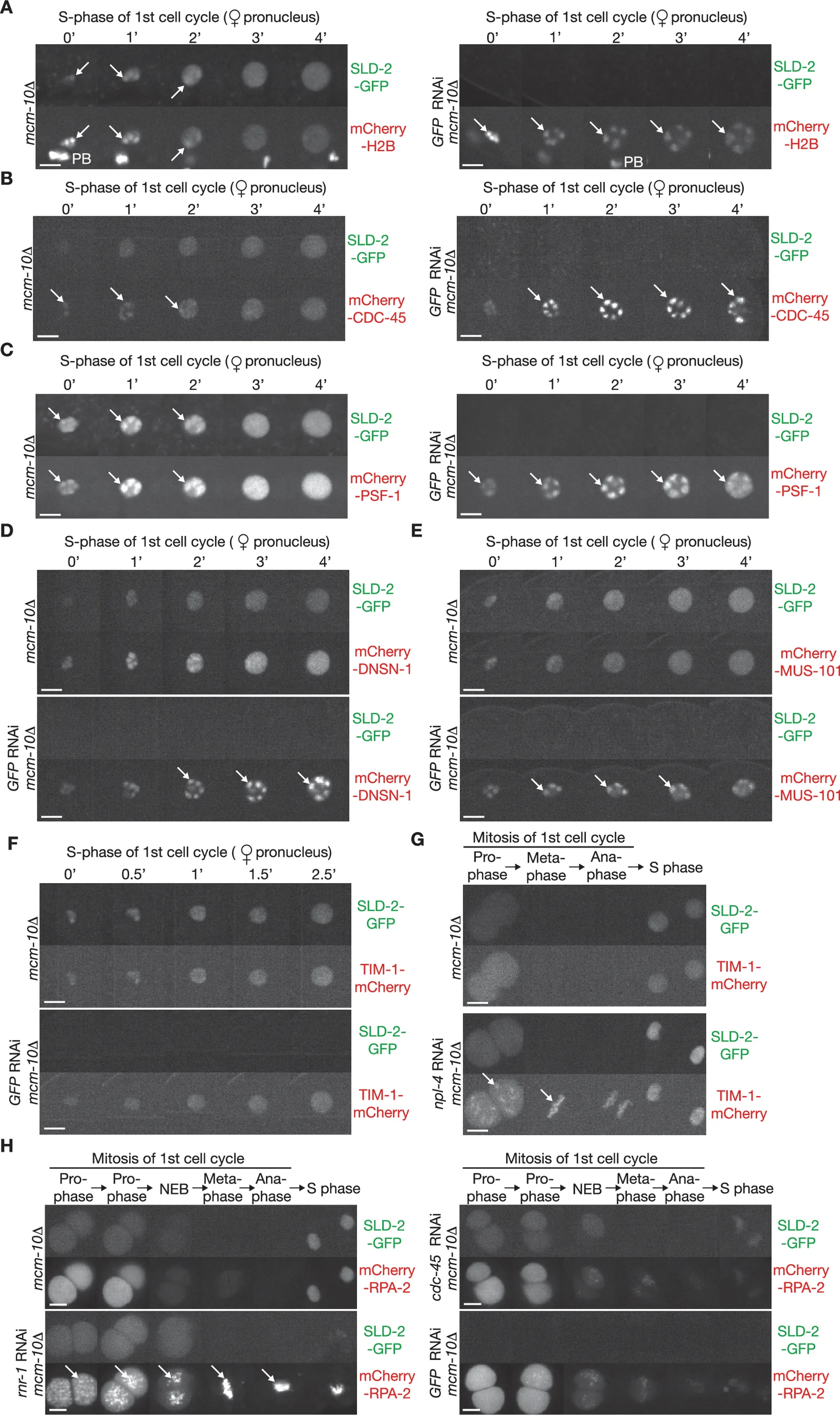
CMG helicase activation is blocked in the combined absence of *C. elegans* MCM-10 and SLD-2. (**A**) Worms of the genotype *mcm-10∆ sld-2-GFP mCherry-histoneH2B* were treated as indicated with *GFP* RNAi, then processed as for Fig. 1D. Arrows indicate SLD-2-GFP and mCherry-HistoneH2B on condensed chromosomes. PB = Polar body. (**B**) Similar experiment with *mcm-10∆ sld-2-GFP mCherry-cdc-45*. Arrows indicate the association of mCherry-CDC-45 with condensed chromosomes in worms lacking both SLD-2 and MCM-10. Note that SLD-2-GFP does not accumulate on condensed chromosomes in *mcm-10∆* worms expressing *mCherry-CDC-45* (top panel), likely due to partial impairment of CDC-45 function upon fusion to the mCherry tag. (**C**–**E**) Analogous experiments with *mcm-10∆ sld-2-GFP mCherry-psf-1* (**C**), *mcm-10∆ sld-2-GFP mCherry-dnsn-1* (**D**) or *mcm-10∆ sld-2-GFP mCherry-mus-101* (**E**). Arrows show the association of mCherry-PSF-1 (**C**), mCherry-DNSN-1 (**D**) and mCherry-MUS-101 (**E**) with condensed chromosomes in embryos lacking both SLD-2 and MCM-10. (**F**) Analogous experiment with *mcm-10∆ sld-2-GFP tim-1-mCherry*. (**G**) The same worms as in (**F**) were treated as shown with *npl-4* RNAi to block CMG helicase disassembly, by depletion of NPL-4, which is an essential ubiquitin receptor of the CDC-48 unfoldase (Mouysset et al, 2008; Sonneville et al, 2017). The association of TIM-1-mCherry with chromatin was then assessed during the first embryonic mitosis (examples are indicated by white arrows). (**H**) *mcm-10∆* worms expressing SLD-2-GFP and mCherry-RPA-2 were treated with RNAi as indicated, before analysis by time-lapse video microscopy of progression through mitosis in the first embryonic cell cycle. Both SLD-2-GFP and mCherry-RPA-2 are nuclear proteins, and mCherry-RPA-2 accumulated in sub-nuclear foci (indicated by arrows) upon depletion of RNR-1, reflecting chromatin association (see Appendix Fig. S3). NEB nuclear envelope breakdown. All scalebars correspond to 5 µm. Videos of the embryos shown in this figure can be found in Movies EV15–EV32. Source data are available online for this figure.

In wild-type embryos, SLD-2 is not detected on chromosomes during early S-phase (Fig. 2D, Control), suggesting that its association with CMG is very transient. Furthermore, and analogous to the behaviour of MCM-10 (Fig. 2A, *cdc-45* RNAi), SLD-2 does not accumulate on condensed chromosomes when CMG assembly is blocked by depletion of CDC-45 (Fig. 2D, *cdc-45* RNAi). However, SLD-2-GFP was detected on condensed chromosomes when CMG activation was delayed in the absence of MCM-10 (Fig. 2E, *mcm-10*∆; also see Fig. 3A,C, *mcm-10*∆). Moreover, the chromatin association of SLD-2 under such conditions was blocked by depletion of GINS (Fig. 2E, GINS RNAi *mcm-10*∆). These data indicate that SLD-2 associates transiently with newly assembled CMG helicase complexes during the initiation of DNA replication, independently of MCM-10. Correspondingly, SLD-2 can bind directly to the CMG helicase in vitro (Fig. 2F).

### MCM-10 and SLD-2 are partially redundant for CMG helicase activation in *C. elegans*

Depletion of SLD-2 in *mcm-10*∆ worms strongly inhibited DNA replication in the first embryonic cell cycle, compared to embryos that individually lacked either MCM-10 or SLD-2 (Fig. 1B,C). Moreover, chromosome decondensation was delayed for longer in embryos lacking both MCM-10 and SLD-2, compared to the effect of removing just one of the two proteins (Figs. 3A and 1D). Both CDC-45 (Fig. 3B) and GINS (Fig. 3C, mCherry-PSF-1) accumulated on condensed chromosomes in the absence of MCM-10 and SLD-2. Similarly, the initiation factors DNSN-1 (Fig. 3D) and MUS-101 (Fig. 3E) persisted on condensed chromosomes in embryos lacking MCM-10 and SLD-2. We also examined the TIMELESS-TIPIN complex (TIM-1 and TIPN-1 in *C. elegans*), which associates with CMG as part of the replisome at replication forks, and found that TIM-1 did not accumulate on condensed chromosomes during early S-phase in the absence of MCM-10 and SLD-2 (Fig. 3F), consistent with recent data indicating that chromatin recruitment of the yeast orthologues of TIMELESS-TIPIN is dependent upon the activation of newly assembled CMG complexes (Weekes et al, 2026). In contrast, TIM-1 persisted on condensed chromatin during mitosis when worms were treated with *npl-4* RNAi (Fig. 3G), which inhibits CMG helicase disassembly during DNA replication termination (Sonneville et al, 2017). These data indicate that CMG helicase complexes assemble in the absence of MCM-10 and SLD-2 but remain in a ‘pre-activation state’ with DNSN-1 and MUS-101, rather than forming active replisomes by association with TIM-1 and other factors.

When DNA synthesis was inhibited after CMG helicase activation in *mcm-10*∆ embryos, by depletion of the ribonucleotide reductase subunit RNR-1 (Fig. 3H, *rnr-1* RNAi + *mcm-10*∆), the RPA-2 subunit of the single-strand DNA-binding protein RPA (Replication Protein A) accumulated in sub-nuclear foci (Fig. 3H). The accumulation of such foci was strongly reduced by co-depletion of CDC-45 (Appendix Fig. S3), indicating that the sub-nuclear foci represent chromatin binding of RPA, which is dependent upon CMG helicase activity. Chromatin-bound foci of RPA-2 were scarcely detected in embryos that lacked MCM-10 and SLD-2, analogous to embryos lacking CDC-45 and contrasting with embryos treated with *rnr-1* RNAi (Fig. 3H). These data are consistent with a defect in CMG helicase function and DNA unwinding in embryos lacking MCM-10 and SLD-2.

Collectively, the above findings indicate that *C. elegans* MCM-10 and SLD-2 are partially redundant for CMG helicase activation, which is profoundly defective in their absence. Correspondingly, chromosome condensation during mitotic prophase of the first embryonic cell cycle was impaired when SLD-2 was depleted in *mcm-10*∆ worms, and chromosome segregation failed during anaphase, producing chromatin bridges in all embryos (Appendix Fig. S1A, *GFP* RNAi + *mcm-10*∆, *n* = 5).

### MCM-10, SLD-2 and DNSN-1 stimulate CMG helicase activity in vitro

**Figure 4:**
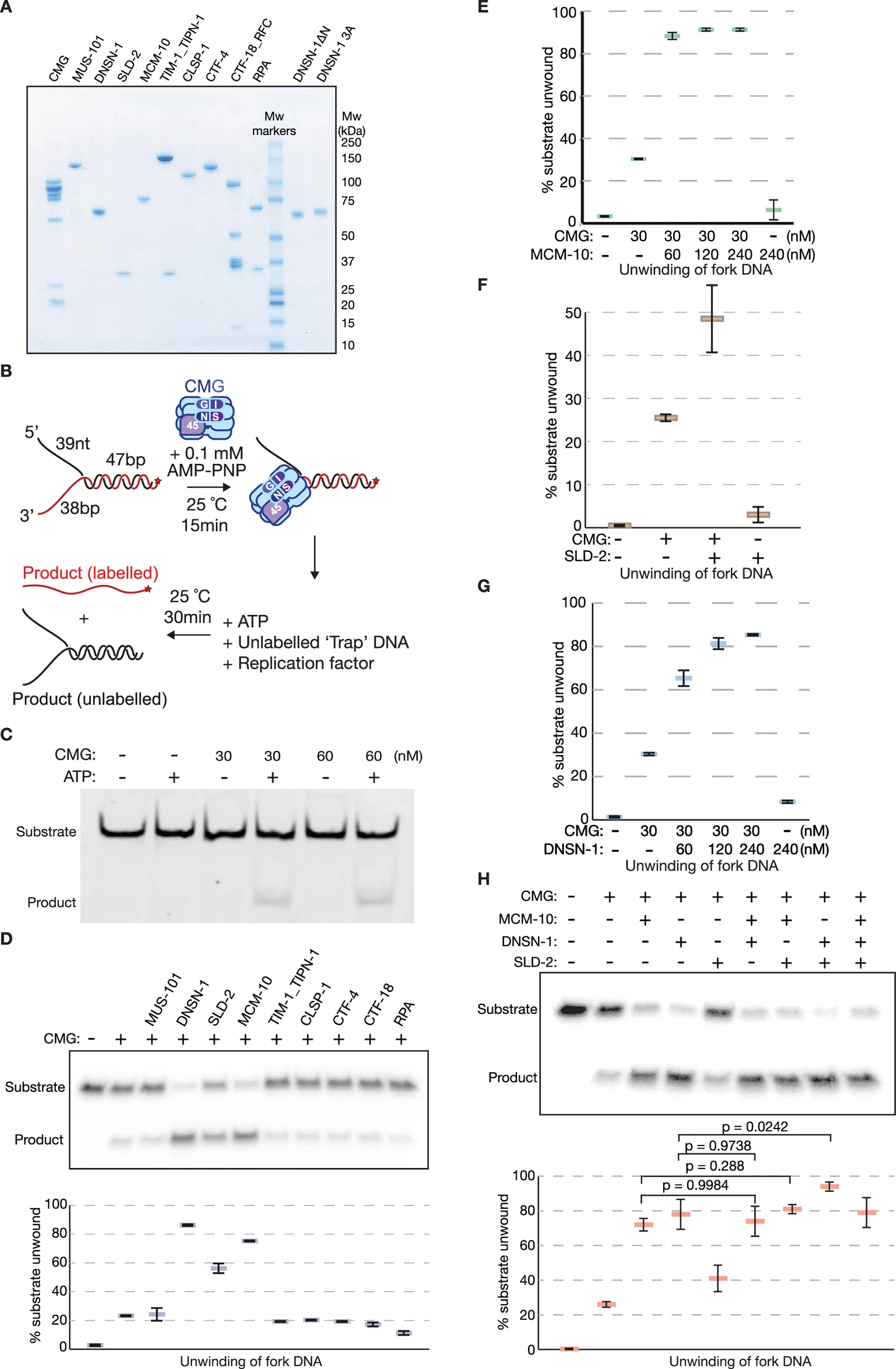
MCM-10, SLD-2 and DNSN-1 stimulate activity of the CMG helicase in vitro. (**A**) The indicated factors were expressed and purified in recombinant form as described in Methods. The purified proteins were resolved by SDS-PAGE and stained with colloidal Coomassie blue. (**B**) Reaction scheme (radio-labelling of the 5’-end of the DNA strand is indicated by a red star). (**C**) Helicase assays were performed as in (**B**), in the presence or absence of ATP and CMG as indicated. Reaction products were separated in a polyacrylamide gel and detected by autoradiography. (**D**) Helicase assays were performed in the presence of the factors shown in (**A**). The percentage of unwound substrate DNA was quantified and shown in the graph at the bottom, indicating mean values and standard deviation from three independent experiments. (**E**) CMG helicase assays in the presence of the indicated concentrations of CMG and MCM-10. The data correspond to the mean values (shown in green) and standard deviation (black error bars) from three independent experiments. (**F**) Similar experiment in the presence of 30 nM CMG and 120 nM SLD-2. Data correspond to the mean values (shown in gold) and standard deviation (black error bars) from three independent experiments. (**G**) Analogous experiment in the presence of the indicated concentrations of CMG and DNSN-1. The data represent the mean values (shown in blue) and standard deviation (black error bars) from three independent experiments. (**H**) CMG helicase assays were performed in the presence of the indicated combinations of MCM-10, DNSN-1 and SLD-2. The mean values from three independent experiments are shown together with corresponding standard deviations. *P* values were then generated via a one-way ANOVA followed by Dunnett’s multiple comparisons test. Also see Appendix Fig. S4E. Source data are available online for this figure.

Given the importance of MCM-10 and SLD-2 for CMG helicase activation in vivo, we tested whether these factors can directly stimulate CMG helicase activity in vitro, as reported previously for budding yeast Mcm10 (Langston et al, 2017; Langston and O’Donnell, 2019; Looke et al, 2017; Wasserman et al, 2019). CMG, MCM-10 and SLD-2 were expressed in recombinant form in budding yeast cells and then purified from cell extracts, as were MUS-101 and DNSN-1 that also promote the initiation of DNA replication in *C. elegans* (Fig. 4A). We also expressed and purified a set of other proteins that bind to the CMG helicase in the *C. elegans* replisome (Sonneville et al, 2017; Xia et al, 2021), such as the TIM-1_TIPN-1 complex, CLSP-1 (CLASPIN), CTF-4 and the CTF-18_RFC clamp loader complex. Finally, we purified RPA, which is a two-protein complex in *C. elegans* rather than three subunits in many other eukaryotic species (Hefel et al, 2021). Subsequently, we assayed the ability of each factor to stimulate ATP-dependent unwinding of a model DNA replication substrate by the *C. elegans* CMG helicase (Fig. 4B,C).

**Figure EV2:**
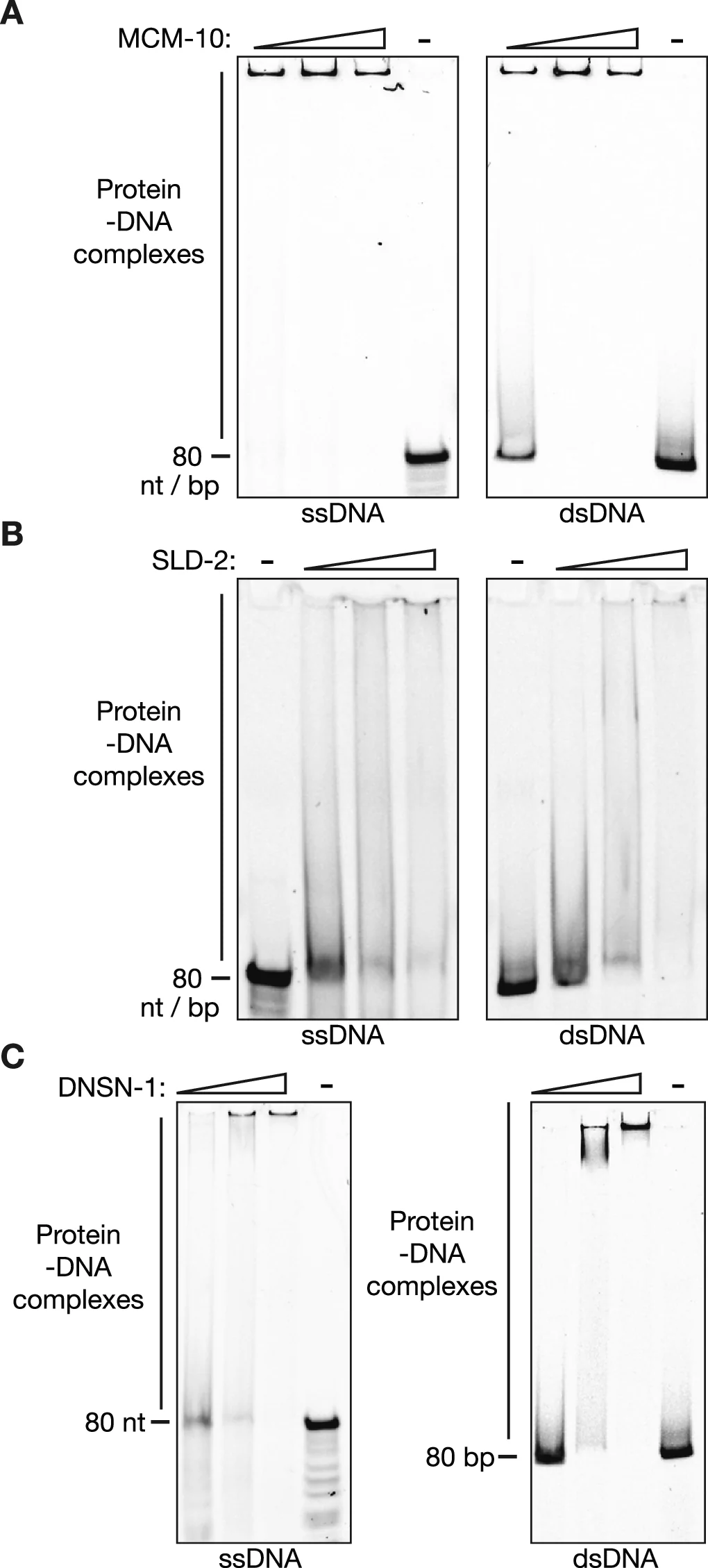
MCM-10, SLD-2 and DNSN-1 all bind to DNA in vitro. As described in Methods, the indicated concentrations of MCM-10 (**A**), SLD-2 (**B**) and DNSN-1 (**C**) were incubated with 5 nM Cy5-labelled DNA at 25 °C for 15 min. Protein–DNA complexes were then analyzed in native polyacrylamide gradient gels before detection of the fluorescently labelled DNA.

**Figure EV3:**
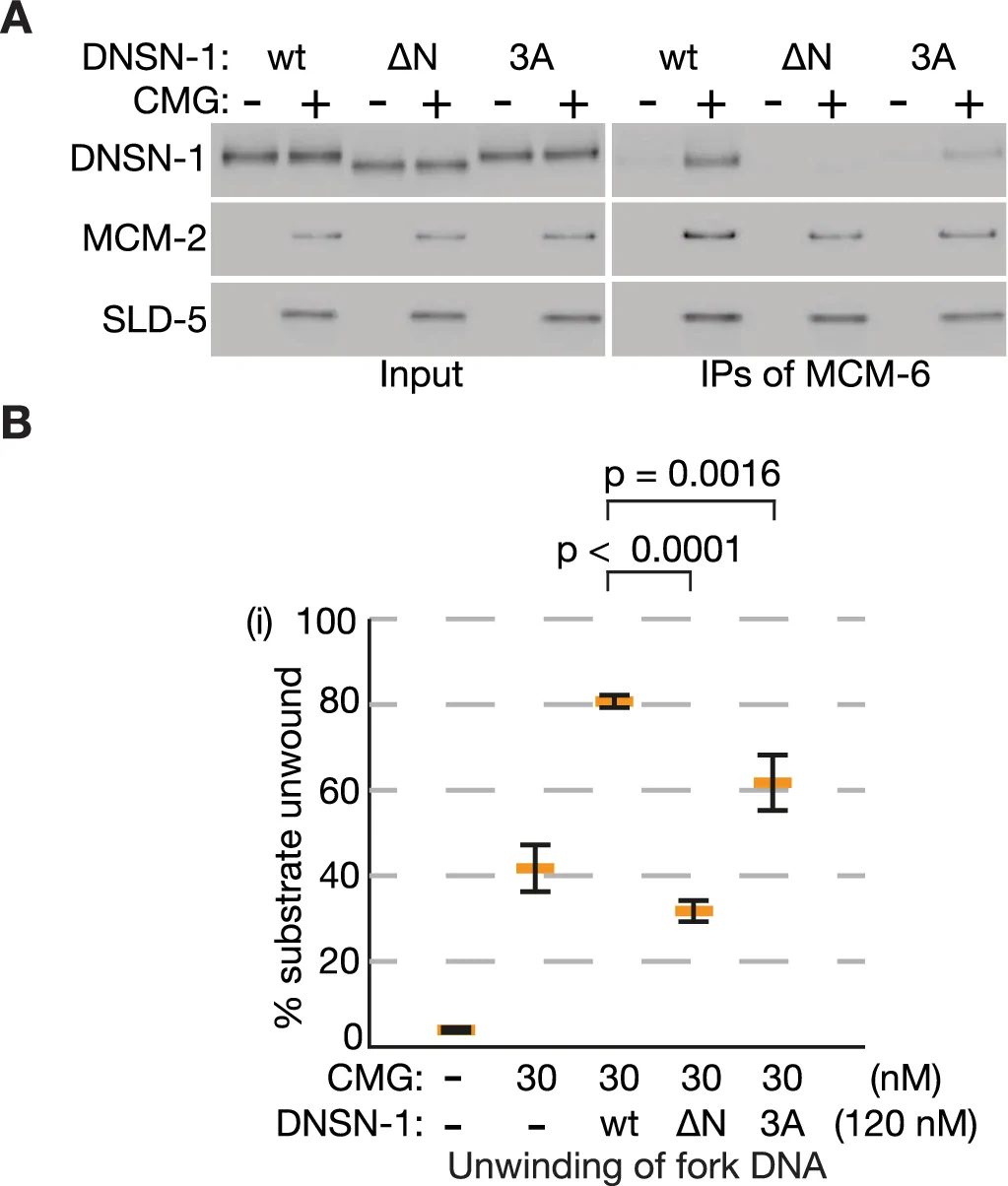
The ability of DNSN-1 to bind CMG is important for the stimulation of helicase activity in vitro. (**A**) Recombinant *C. elegans* CMG was mixed as indicated with wild-type DNSN-1 (wt), DNSN-1 lacking the first 20 amino acids (DNSN-1-∆N), or DNSN-1 with mutations in three conserved residues that contact the MCM-3 subunit of the CMG helicase (DNSN-1-3A). As described above for Fig. 2C, F, the CMG helicase was then isolated by immunoprecipitation of the MCM-6 subunit, and the indicated proteins were detected by immunoblotting. (**B**) The ability of DNSN-1, DNSN-1-∆N and DNSN-1-3A to stimulate CMG helicase activity was monitored in vitro as for Fig. 4D, G. The data correspond to three independent experiments, with mean values and corresponding standard deviations. *P* values were generated via a one-way ANOVA followed by Dunnett’s multiple comparisons test.

**Figure EV4:**
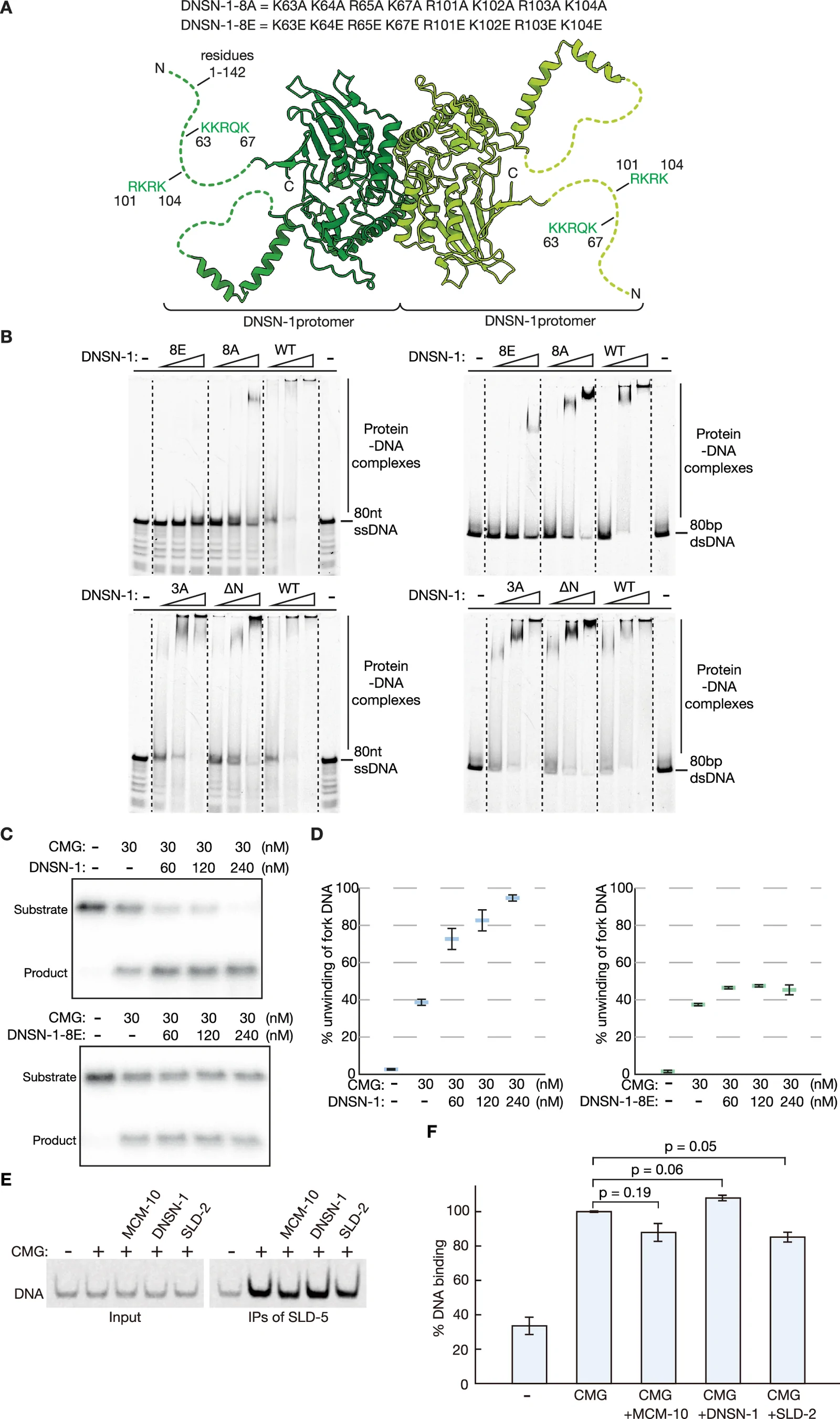
DNA binding by DNSN-1 is important for stimulation of CMG helicase activity. (**A**) AlphaFold prediction of the DNSN-1 dimer, indicating the location within the amino-terminal disordered region of mutations corresponding to the DNSN-1-8A and DNSN-1-8E alleles. (**B**) DNA binding activity of the indicated forms of DNSN-1 were assayed as in Fig. EV2. DNSN-1-∆N = deletion of residues 5–19, whereas DNSN-1-3A = W425A I429A V431A (Xia et al, 2023). (**C**) CMG helicase assays were performed as in Fig. 4, to compare stimulation by the indicated concentrations of DNSN-1 and DNSN-1-8E. (**D**) Quantification of the data from (**D**). The mean values from three independent experiments are shown together with corresponding standard deviations. (**E**) Loading of the CMG helicase onto a forked DNA substrate was assayed as described in Methods. The DNA substrate was labelled with Cy5 to facilitate detection. (**F**) Quantification of the data from (**E**). The mean values from three independent experiments are shown together with corresponding standard deviations. *P* values were generated via a one-way ANOVA followed by Dunnett’s multiple comparisons test.

In contrast to the other tested factors, MCM-10, SLD-2 and DNSN-1 all stimulated DNA unwinding activity in vitro (Fig. 4D), dependent upon the presence of CMG (Fig. 4E–G). MCM-10, SLD-2 and DNSN-1 all share the ability to bind directly to the CMG helicase (Fig. 2C,F and (Xia et al, 2023)) and to DNA (Fig. EV2). Whereas the interaction of metazoan MCM-10 with CMG is poorly characterised and the binding of SLD-2 to CMG has not been studied structurally, the association of DNSN-1 with CMG has been analysed by cryo-electron microscopy, revealing the importance not only of an amino-terminal helix in DNSN-1 that binds to the SLD-5 subunit of GINS, but also of three conserved DNSN-1 residues that bind to MCM-3 (Xia et al, 2023). Removal of the amino-terminal helix prevents DNSN-1 from binding to CMG (Fig. EV3A), as seen previously (Xia et al, 2023), and impairs the ability of DNSN-1 to stimulate CMG helicase activity in vitro (Fig. EV3B). Furthermore, mutation of the MCM-3-binding site led to reduced binding of DNSN-1 to CMG (Fig. EV3A), and partially reduced the ability of DNSN-1 to stimulate CMG helicase activity (Fig. EV3B). In addition, charge reversal of positively-charged residues in the disordered amino-terminal tail of DNSN-1 inhibited DNA binding (Fig. EV4A,B, DNSN-1-8E) and impaired stimulation of CMG helicase activity in vitro (Fig. EV4C,D). DNSN-1 did not affect the loading of the CMG helicase onto fork DNA (Fig. EV4E,F), indicating that it stimulates helicase activity in some other way. In summary, these findings indicate that *C. elegans* MCM-10, SLD-2 and DNSN-1 all stimulate CMG helicase activity in vitro, most likely via direct binding to both CMG and DNA.

We also explored whether the same factors discussed above could stimulate DNA unwinding by a head-to-head pair of CMG helicase complexes, analogous to the situation at origins during initiation. As in previous studies of yeast Mcm10 (Langston and O’Donnell, 2019), we used substrates that promote the loading of one or two CMG helicase complexes around double-strand DNA (Appendix Fig. S4A). Whereas CMG alone had very little activity against such substrates, unwinding of a substrate that loaded two CMG helicase complexes was stimulated by MCM-10, DNSN-1 or SLD-2 but not by the other tested factors (Appendix Fig. S4B,C). In contrast, an analogous substrate that can only load a single CMG helicase was unwound several times less efficiently (Appendix Fig. S4D).

Although a combination of MCM-10 and DNSN-1 did not stimulate DNA unwinding more than the single treatments, the stimulation of CMG activity by SLD-2 was additive with either MCM-10 or DNSN-1 (Fig. 4H; Appendix Fig. S4E).

### Inactivation of MCM10 and RECQL4 causes synthetic lethality in mouse ES cells

Previous studies showed that transient depletion of MCM10 in human cancer cell lines delayed cell cycle progression, probably due to defects in DNA replication (Chattopadhyay and Bielinsky, 2007). Moreover, deletion of the mouse *Mcm10* gene causes lethality around day 8.5 of embryonic development (Lim et al, 2011). These findings suggested that MCM10 is essential for DNA replication in mammalian cells.

In contrast to yeasts, the orthologue of Sld2 in many eukaryotic species contains a DNA helicase of the RecQ family, after the region of Sld2 homology, making a protein that in vertebrates is called RECQL4 (Gaggioli et al, 2014). Studies of RECQL4 in *Xenopus laevis* indicated that it is essential for the initiation of DNA replication, dependent upon the amino-terminal region that is homologous to yeast Sld2 (Matsuno et al, 2006; Sangrithi et al, 2005). However, unlike Sld2 in *Saccharomyces cerevisiae*, *Xenopus* RECQL4 is dispensable for CMG assembly and instead is required for helicase activation (Matsuno et al, 2006; Sangrithi et al, 2005). Nevertheless, recent studies showed that human cells can survive in the absence of detectable expression of RECQL4 (Padayachy et al, 2024). Small deletions or insertions in exon1 of RECQL4 were introduced by CRISPR-Cas9 genome editing in human U2OS cells, producing viable cells in which expression of the RECQL4 protein was not detected, although it remains possible that truncated forms of the protein were still expressed at a low level and might be important to preserve viability (Padayachy et al, 2024). Indeed, a very recent study of mouse myeloid cells showed that cell viability could be maintained by expression of an amino-terminal fragment of RECQL4, containing the region of homology to yeast and *C. elegans* Sld2/SLD-2, whereas complete loss of RECQL4 was lethal (Buco et al, 2026).

To explore whether the joint role of *C. elegans* MCM-10 and SLD-2 in the activation of the CMG helicase is conserved in mammalian cells, we inactivated the *Mcm10* and *Recql4* genes in mouse embryonic stem cells (mouse ES cells), which spend most of the cell cycle in S-phase and provide a model system for studying the regulation of the mammalian CMG helicase (Evrin et al, 2023; Villa et al, 2021). Firstly, we used CRISPR and the Cas9-D10A nickase enzyme, together with pairs of guide RNAs that target exon 3 of *Mcm10* (Appendix Fig. S5A) or exon1 of *Recql4* (Appendix Fig. S5B), to make small deletions at the 5’ end of the coding sequence. In this way, we isolated viable clones that lacked detectable expression of MCM10 or RECQL4 (Appendix Fig. S5C–F). *Mcm10∆* clones grew at a similar rate to control cells, whereas *Recql4∆* cells grew more slowly (19.9 ± 1.8 h for *Recql4∆*, compared to 13 ± 0.5 h for control).

Subsequently, we used CRISPR and wild-type Cas9 to cut both ends of the *Mcm10* coding sequence and delete the entire 22.6 kb gene (Appendix Fig. S6). This produced viable clones that lacked MCM10 protein (Appendix Fig. S6D) and grew slightly slower than control cells (15 ± 0.7 h). These data show that mouse ES cells are able to grow in the complete absence of MCM10, indicating that mammalian cells must have at least one MCM10-independent mechanism for activating the CMG helicase. We used a similar approach to try and delete the entire 7 kb *Recql4* gene (Appendix Fig. S7), but only isolated clones that were heterozygous for the complete deletion of *Recql4*, suggesting that low-level expression of truncated fragments of RECQL4 is important to preserve cell viability in mouse ES cells.

**Figure 5:**
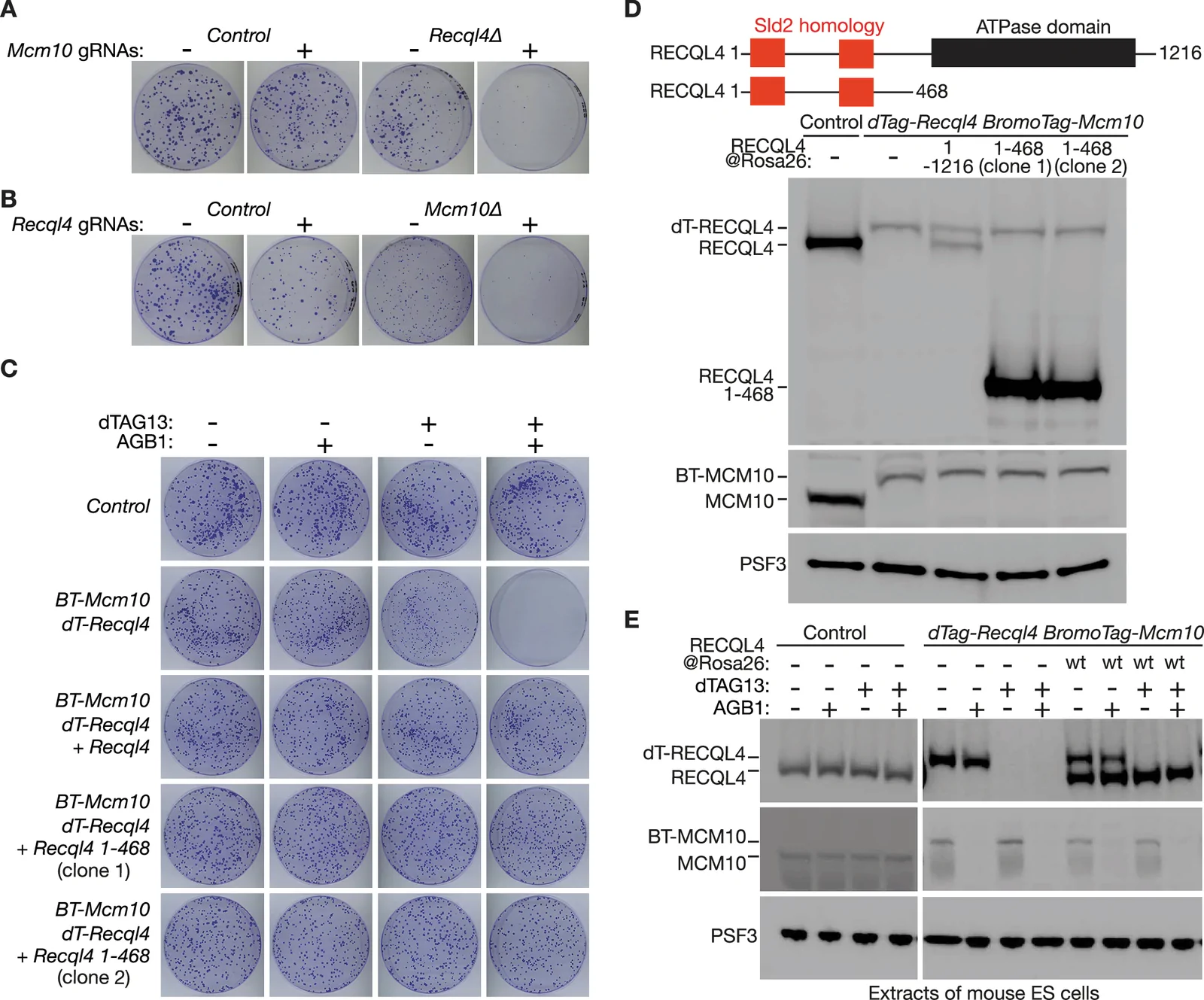
Loss of MCM10 and RECQL4 causes synthetic lethality that is rescued by the SLD-2-homology region of RECQL4. (**A**) Control mouse ES cells and *Recql4∆* cells (Appendix Fig. S5) were transfected as indicated with plasmids expressing the Cas9-D10A nickase and guide RNAs (gRNAs) specific to exon 3 of the murine *Mcm10* gene (Appendix Fig. S5A). Cell proliferation was then assayed as described in Methods. (**B**) Similar experiment to (**A**) but with control mouse ES cells and *Mcm10∆* cells (Appendix Fig. S5) transfected with plasmids expressing the Cas9-D10A nickase and guide RNAs (gRNAs) specific to exon1 of the murine *Recql4* gene (Appendix Fig. S5B). (**C**) Mouse ES cells of the indicated genotypes were treated as shown with 250 nM of the PROTACs AGB1 or dTAG13, before monitoring cell proliferation as described in Methods. BT BromoTag, dT dTAG, ‘+ Recql4’ expression of full-length RECQL4 or RECQL4 1–468 as indicated, from the *Rosa26* locus. (**D**) Immunoblots showing expression of the indicated proteins in the cells shown in (**C**). (**E**) Immunoblots illustrating degradation of dTAG-RECQL4 in response to the PROTAC dTAG13, and depletion of BromoTag-MCM10 in response to the PROTAC AGB1. Source data are available online for this figure.

Since mouse ES cells are able to grow in the absence of MCM10 and with an extremely low level of RECQL4 (below the detection limit in immunoblots), we tested the effect of using CRISPR-Cas9 to make deletions in both genes simultaneously, using the approach in Appendix Fig. S5. Whereas *Mcm10∆* had little impact on cell growth in control cells, the combination of *Mcm10∆* with *Recql4∆* inhibited colony formation (Fig. 5A). Moreover, *Recql4∆* had a modest impact on colony formation in control cells but strongly impaired the growth of *Mcm10∆* cells (Fig. 5B). These findings indicated that simultaneous loss of MCM10 and RECQL4 causes synthetic lethality in mouse ES cells.

To study the phenotype of mammalian cells lacking MCM10 and RECQL4 in more depth, we generated a conditional depletion system. CRISPR-Cas9 was used to make mouse ES cells in which MCM10 was fused to BromoTag that is recognised by the ubiquitin ligase CUL2^VHL^ in the presence of the Proteolysis Targeting Chimera (PROTAC) known as AGB1 (Bond et al, 2021). In addition, RECQL4 was fused to dTAG (Nabet et al, 2018) that is recognised by the ubiquitin ligase CUL4^CRBN^ in the presence of a PROTAC called dTAG13 (Appendix Fig. S8). The growth of control cells was not affected by exposure to either or both of the two PROTACs (Fig. 5C, Control). Moreover, *BromoTag-Mcm10 dTAG-Recql4* cells grew well when BromoTag-MCM10 was depleted by the addition of AGB1, and more slowly after depletion of dTAG-RECQL4 by the addition of dTAG13 (Fig. 5C, *BromoTag-Mcm10 dTAG-Recql4*), consistent with the knockout data described above. However, simultaneous depletion of both BromoTag-MCM10 and dTAG-RECQL4 produced a lethal phenotype (Fig. 5C, *BromoTag-Mcm10 dTAG-Recql4*, AGB1 + dTAG13), which could be rescued by expression from the *Rosa26* ‘safe haven’ locus of either full-length RECQL4, or RECQL4 1–468 that contains the Sld2-homology regions (Fig. 5C–E). These findings indicate that MCM10 and the Sld2-homology regions of RECQL4 are partially redundant for the proliferation of mouse ES cells.

### Absence of MCM10 and RECQL4 blocks DNA replication after assembly of the CMG helicase

**Figure 6:**
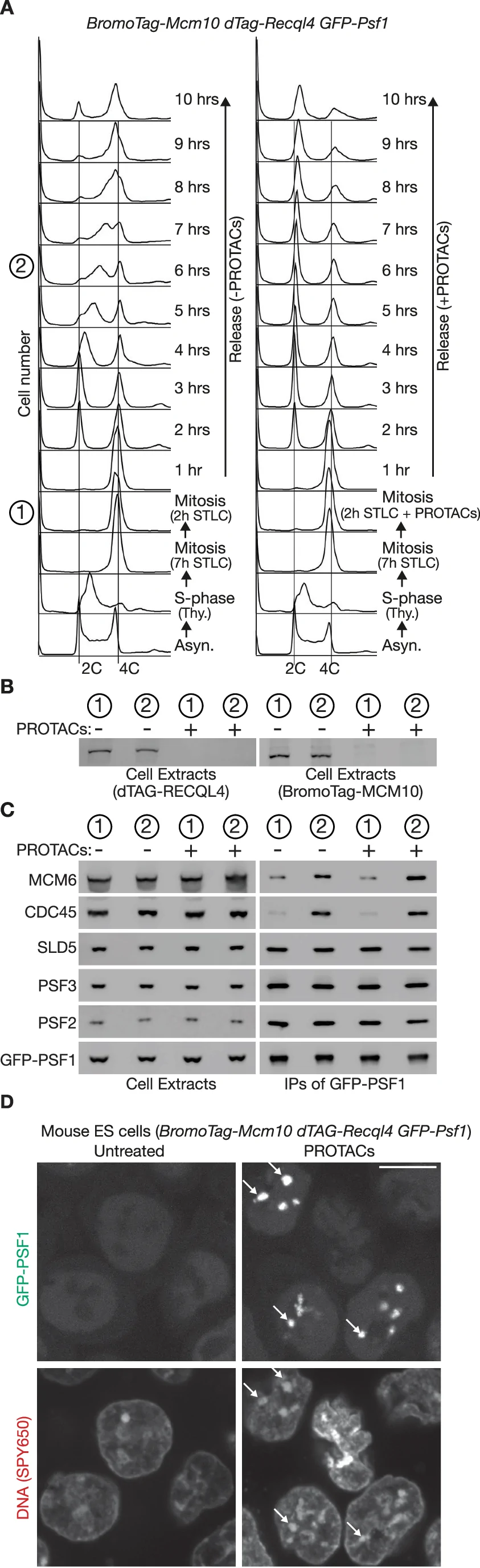
MCM10 and RECQL4 are partially redundant for DNA replication after CMG helicase assembly in mouse ES cells. (**A**) Mouse ES cells of the indicated genotype were synchronised firstly in S-phase by addition of thymidine, and then in mitosis in the presence of the kinesin inhibitor STLC, as described in Methods. Half of the culture was then exposed for two hours to 250 nM of the PROTACs AGB1 and dTAG13, to induce degradation of BromoTag-MCM10 and dTAG-RECQL4, before cells were released from mitosis and allowed to progress into the subsequent cell cycle. DNA content was monitored throughout the experiment by flow cytometry, and additional samples were taken at the end of the mitotic arrest (‘1’) and six hours after release from mitosis (‘2’), in order to monitor protein expression and assembly of the CMG helicase. (**B**) The level of BromoTag-MCM10 (right) and dTAG-RECQL4 (left) was monitored by immunoblotting at the indicated stages of the experiment in (**A**). (**C**) Native protein extracts were generated at the indicated times from the experiment in (A) and used to isolate the GINS sub-complex (SLD5-PSF1-PSF2-PSF3) of the CMG helicase, by immunoprecipitation of GFP-tagged PSF1. The indicated proteins were monitored by immunoblotting. (**D**) Mouse ES cells of the genotype *BromoTag-Mcm10 dTAG-Recql4 GFP-Psf1* were incubated for four hours in the presence or absence of 250 nM of the PROTACs AGB1 and dTAG13. Live cells were then stained with the DNA-binding dye SPY650-DNA (see Methods). Arrows indicate the accumulation of GFP-PSF1 on patches of constitutive heterochromatin. The scale bar corresponds to 10 µm. Source data are available online for this figure.

To examine the impact of depleting MCM10 and RECQL4 on cell cycle progression, we synchronised *BromoTag-Mcm10 dTAG-Recql4* cells in S-phase, released them into the subsequent mitosis, and then held them in mitosis for a further period, in the presence or absence of the PROTACs AGB1 and dTAG13, before releasing cells from mitotic arrest (Fig. 6A). Depletion of MCM10 and RECQL4 (Fig. 6B) had no impact on progression through mitosis or cell division, but inhibited DNA replication in the subsequent cell cycle (Fig. 6A). By immunoprecipitation of a GFP-tagged form of the GINS subunit PSF1, we observed that cells expressing MCM10 and RECQL4 assembled the CMG helicase complex upon entry into S-phase (Fig. 6C, −PROTACs). Importantly, cells lacking MCM10 and RECQL4 also assembled the CMG helicase upon entry into S-phase (Fig. 6C, +PROTACs). To monitor CMG assembly by a different method, we also used spinning disk confocal microscopy. A previous study showed that CMG colocalises transiently with PCNA and other replisome components on patches of heterochromatin, during DNA replication in mouse ES cells (Villa et al, 2021). Using the same approach, GFP-PSF1 was found to accumulate on patches of heterochromatin in cells lacking MCM10 and RECQL4 (Fig. 6D; 45% cells, *n* = 623, compared to 16% of cells in the presence of MCM10 and RECQL4, *n* = 622). These data indicate that MCM10 and RECQL4 act downstream of CMG helicase assembly and are partially redundant for an essential step during chromosome replication in the mammalian cell cycle.

### CMG helicase activation is defective in mammalian cells lacking MCM10 and RECQL4

**Figure 7:**
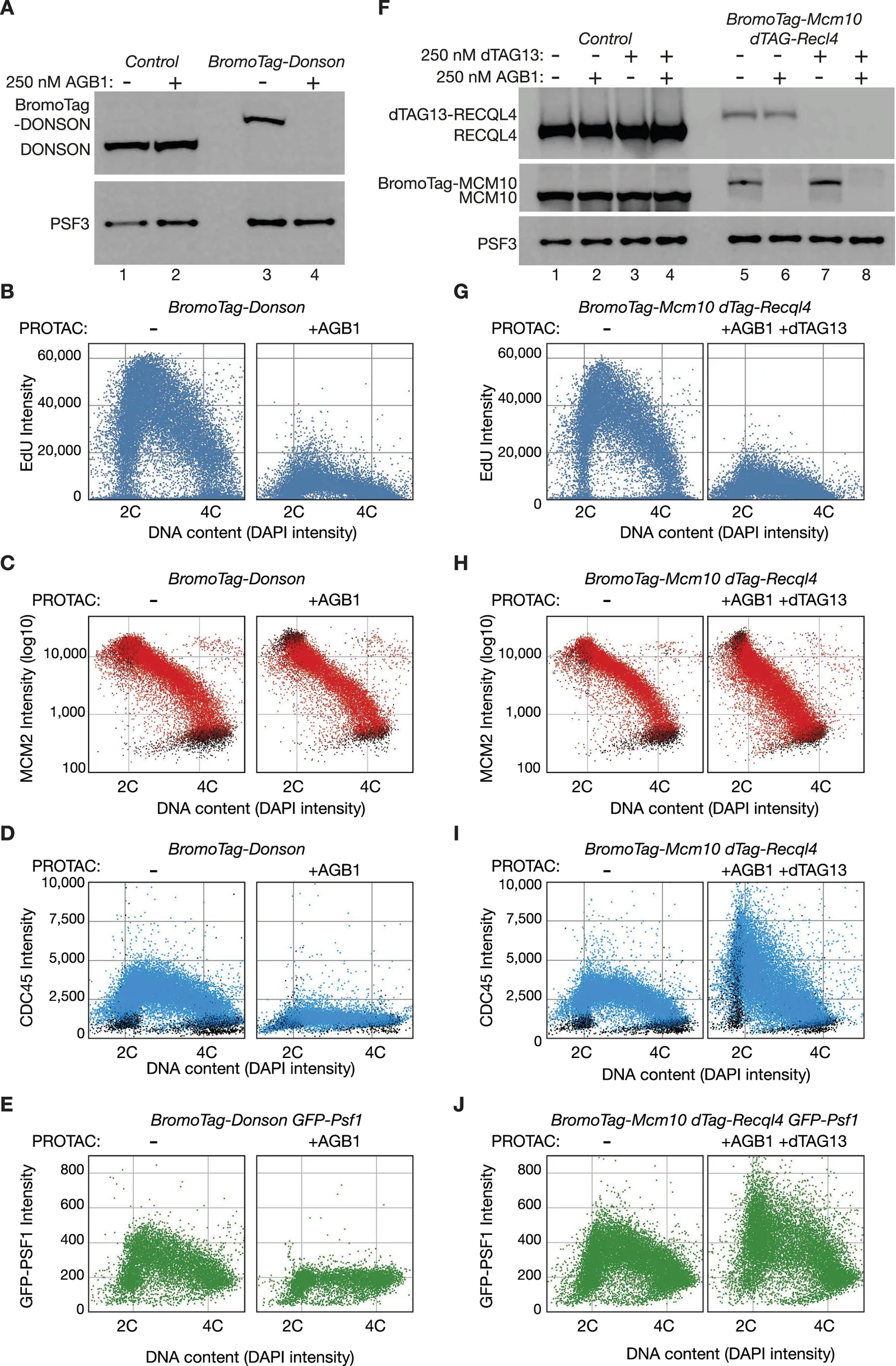
The CMG helicase accumulates on chromatin in mouse ES cells lacking MCM10 and RECQL4. (**A**) Asynchronous cultures of control and *BromoTag-Donson* mouse ES cells were incubated for three hours as indicated in the presence or absence of 250 nM of the PROTAC AGB1. Cell extracts were used to monitor the level of the indicated proteins by immunoblotting. (**B**–**E**) *BromoTag-Donson* mouse ES cells were incubated for eight hours in the presence or absence of 250 nM AGB1, followed by treatment for 20 min with the nucleoside precursor EdU. Cells were then analysed by high-content screening microscopy as described in Methods, to monitor DNA synthesis and the chromatin association of the indicated components of the CMG helicase (‘EdU/CDC45/MCM2/GFP-PSF1 intensity’ = mean EdU/CDC45/MCM2/GFP-PSF1 intensity per nucleus). In contrast to control cells (Appendix Fig. S10), depletion of *BromoTag-Donson* impairs DNA synthesis and CMG helicase assembly, as described previously (Evrin et al, 2023). For panels (**C**, **D**), EdU-negative cells (see Methods) are indicated in black. For panel (**E**), cells also expressed GFP-PSF1. Also see Appendix Fig. S9. (**F**) Control and *BromoTag-Mcm10 dTAG-Recql4* mouse ES cells were incubated for 3 h as indicated in the presence or absence of 250 nM of the PROTACs AGB1 and dTAG13. Cell extracts were used to monitor the level of the indicated proteins by immunoblotting. (**G**–**J**) *BromoTag-Mcm10 dTAG-Recql4* mouse ES cells were incubated for eight hours in the presence or absence of 250 nM AGB1 and dTAG13, followed by treatment for 20 min with EdU. Cells were then analysed as described above by high-content screening microscopy. For panels (**H**) and (**I**), EdU-negative cells (see Methods) are indicated in black. For panel (**J**), cells also expressed GFP-PSF1. Equivalent data from the same experiment for the control and for *BromoTag-Mcm10 dTAG-Recql4* cells, in which just one of BromoTag-MCM10 or dTAG-RECQL4 was depleted, are shown in Appendix Fig. S10. Also see Appendix Fig. S9. Source data are available online for this figure.

**Figure 8:**
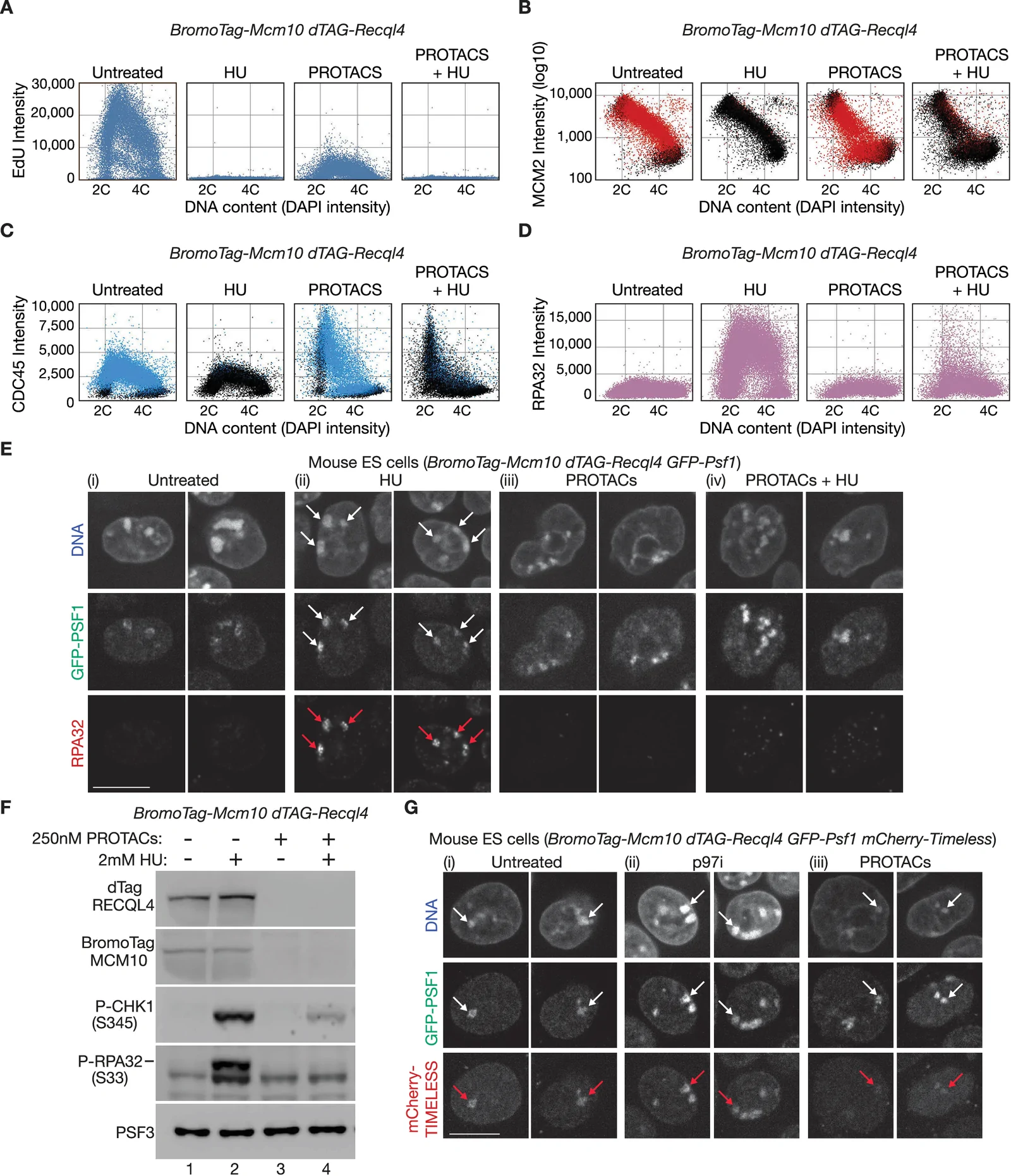
Depletion of MCM10 and RECQL4 blocks recruitment of TIMELESS and RPA to chromatin and inhibits checkpoint activation. (**A**–**C**) Control and *BromoTag-Mcm10 dTAG-Recql4* mouse ES cells were treated as shown (HU = 2 mM hydroxyurea for 2 h; PROTACs = 250 nM AGB1 and dTAG13 for 16 h; PROTACS + HU = 250 nM AGB1 and dTAG13 for 16 h, plus 2 mM hydroxyurea for the last two hours), before treatment for 20 min with EdU. Cells were analysed by high-content screening microscopy as described above for Fig. 7 and in Methods. In panels (**B**, **C**), EdU-negative cells (see Methods) are indicated in black. (**D**) Chromatin-bound RPA32 was monitored in an analogous experiment to (**A**–**C**), but without EdU incorporation. Also see Appendix Fig. S11. (**E**) Mouse ES cells of the indicated genotype were treated as in (**A**–**D**), before fixation and immunofluorescence staining with antibodies to RPA32, together with the DNA-binding dye Hoechst (see Methods). Red arrows indicate the accumulation of RPA32, on patches of constitutive heterochromatin with GFP-PSF1 (white arrows). The scale bar corresponds to 10 µm. (**F**) Samples from the same experiment as in (**A**–**D**) were used to prepare cell extracts that were analysed by immunoblotting for the indicated proteins. ‘P-CHK1 (S345)’ corresponds to phosphorylation of Serine 345 of CHK1, whereas ‘P-RPA32 (S33)’ corresponds to phosphorylation of Serine 33 of RPA32, both of which are mediated by the ATR kinase in response to checkpoint activation. (**G**) Analogous experiment to (**E**), in which mouse ES cells of the indicated genotype were treated as shown with 5 µm CB-5083 (p97i) or a combination of 250 nM AGB1 and 250 nM dTAG13 (PROTACS), before fixation and analysis by spinning disk confocal microscopy. The accumulation of GFP-PSF1 on heterochromatin is indicated by white arrows, whereas red arrows denote mCherry-TIMELESS. The scale bar corresponds to 10 µm. Source data are available online for this figure.

To examine in more detail the impact of entering S-phase in the absence of MCM10 and RECQL4, we used high-content screening microscopy to analyse the behaviour of thousands of individual cells, and compared the impact of degrading MCM10 and RECQL4 with degradation of the essential CMG assembly factor DONSON. Consistent with previous findings (Evrin et al, 2023), degradation of DONSON in mouse ES cells (Fig. 7A) impaired DNA synthesis (Fig. 7B; Appendix Fig. S9A,C), did not prevent association of the MCM2-7 proteins with chromatin during G1-phase (Fig. 7C), but blocked the recruitment of CDC45 (Figs. 7D and S9E) and GINS (Figs. 7E and S9G) to chromatin during S-phase. Depletion of both MCM10 and RECQL4 (Fig. 7F, lane 8) inhibited DNA synthesis to a similar degree to loss of DONSON (Figs. 7G and S9B,D) and also did not affect loading of MCM2-7 proteins on chromatin during G1-phase (Fig. 7H). However, chromatin association of CDC45 (Figs. 7I and 9F) and GINS (Figs. 7J and S9H) was enhanced rather than impaired, in S-phase cells that lacked MCM10 and RECQL4. This phenotype was not seen when the progression of DNA replication forks was blocked by treating cells with hydroxyurea, which inhibits ribonucleotide reductase and impairs the synthesis of dNTPs (Fig. 8A–C, HU), suggesting that enhanced loading of CDC45 and GINS does not result from a defect in the elongation stage of chromosome replication. Instead, these data suggested that the CMG helicase is assembled in the absence of MCM10 and RECQL4 but remains inactive, leading progressively to the accumulation of CMG on chromatin, as cells fire more origins throughout the course of S-phase.

**Figure EV5:**
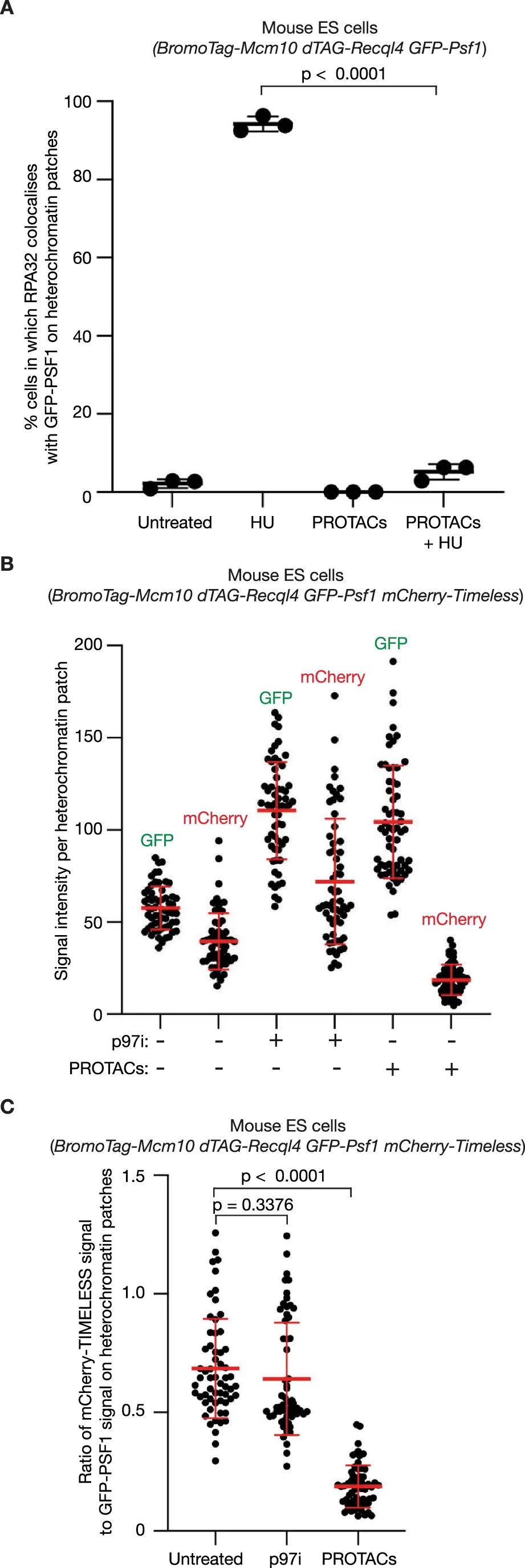
Quantification of recruitment of RPA, TIMELESS and CMG to heterochromatin patches in mouse ES cells. (**A**) Quantification of the data from Fig. 8E, showing the proportion of cells with GFP-PSF1 on heterochromatin patches that also showed colocalisation of RPA32 with GFP-PSF1 (see Methods). The data correspond to the mean values and corresponding standard deviations from three independent experiments. *P* values were generated via a one-way ANOVA followed by Dunnett’s multiple comparisons test. (**B**) Quantification of data from the experiment in Fig. 8G (see Methods), showing the signal intensity for GFP-PSF1 and mCherry-TIMELESS on heterochromatin patches in cells treated as indicated. The data correspond to 20 patches in each of three independent experiments, with mean values shown as red bars, together with corresponding standard deviations. (**C**) The ratio of the mCherry-TIMELESS and GFP-PSF1 signals on individual heterochromatin patches was calculated for the data in (**B**). *P* values were generated via a one-way ANOVA followed by Dunnett’s multiple comparisons test.

To test whether DNA unwinding is inhibited in the absence of MCM10 and RECQL4, we grew *BromoTag-MCM10 dTAG-Recql4* cells in the presence or absence of PROTACs, before treating cells for two hours with hydroxyurea (Fig. 8A–C). In the absence of PROTACs, hydroxyurea treatment led to the accumulation of RPA on chromatin (Figs. 8D,E and EV5A, HU), due to continued DNA unwinding in the absence of DNA synthesis. In contrast, prior depletion of MCM10 and RECQL4 greatly inhibited the chromatin association of RPA in cells treated with hydroxyurea (Figs. 8A–E and EV5A, PROTACs + HU). These findings indicated that CMG assembly in the absence of MCM10 and RECQL4 does not lead to helicase activation.

Hydroxyurea treatment normally induces the S-phase checkpoint pathway, by recruitment of the ATR kinase to RPA-coated single-stranded DNA at stalled replication forks (Yates et al, 2025), However, checkpoint activation was impaired when cells lacking MCM10 and RECQL4 were treated with hydroxyurea (Fig. 8F, compare phosphorylation of CHK1 and RPA32 in lanes 2 and 4), reflecting the assembly of inactive CMG helicase complexes that cannot support DNA unwinding. The lack of checkpoint activation likely explains why inactive CMG complexes not only assemble at the earliest origins of DNA replication, but also form in late replicating heterochromatin (Fig. 6D). In turn, the progressive assembly of inactive CMG complexes throughout S-phase explains the accumulation of CMG to high levels in the absence of MCM10 and RECQL4 (Fig. 7I,J), compared to normal S-phase in which CMG assembly at origins is balanced by CMG disassembly during DNA replication termination (Dewar et al, 2015; Maric et al, 2014; Moreno et al, 2014),

Finally, we monitored the chromatin association of TIMELESS in cells lacking MCM10 and RECQL4, to investigate whether the formation of inactive CMG complexes is associated with replisome assembly. In untreated cells, TIMELESS co-localised on heterochromatin patches with GFP-PSF1 (Fig. 8G (i), 100% of 276 cells, *n* = 3 independent experiments). Moreover, TIMELESS and GFP-PSF1 both accumulated on heterochromatin (Figs. 8G (ii) and EV5B), when disassembly of the CMG helicase was blocked by inhibition of the p97 unfoldase (Villa et al, 2021), which is the mammalian orthologue of *C. elegans* CDC-48. However, the colocalization of chromatin-bound TIMELESS with GFP-PSF1 was impaired in cells lacking MCM10 and RECQL4 (Figs. 8G (iii) and EV5B,C). Collectively, the above data indicate that the CMG helicase is assembled in mammalian cells that lack MCM10 and RECQL4, but the helicase remains inactive and does not support replisome assembly or the initiation of DNA synthesis.

## Discussion

Our data indicate that MCM-10 and SLD-2 play a partially redundant role in CMG helicase activation during DNA replication initiation in *C. elegans*, and the same is true for MCM10 and RECQL4 in mammalian cells, with the role of RECQL4 being dependent upon its amino-terminal region that is homologous to SLD-2.

Mcm10 is essential for CMG helicase activation in budding yeast (Douglas et al, 2018; van Deursen et al, 2012; Watase et al, 2012) and binds both to CMG and to DNA (Gambus et al, 2006; Henrikus et al, 2024; Langston et al, 2017; Looke et al, 2017). Three recent studies indicate that yeast Mcm10 interacts with two head-to-head CMG helicase complexes around double-stranded DNA, locking the CMG dimer in a specific register that likely facilitates the extrusion of one DNA strand through the Mcm2-Mcm5 gate (preprint: Palm et al, 2026; preprint: Taylor-Cross et al, 2026;Weekes et al, 2026).

Another recent study indicates that budding yeast Sld2 is also essential for the activation of dimeric CMG complexes during initiation (Puhringer et al, 2026), although the mechanism remains to be established. Sld2 was reported to bind to DNA, like Mcm10, but the significance of DNA binding remains to be determined for both proteins. Mutations in Mcm10 that abolish DNA binding in vitro do not prevent the initiation of chromosome replication (Henrikus et al, 2024; preprint: Taylor-Cross et al, 2026), but it remains possible that Mcm10 and Sld2 might share a redundant role in DNA binding during helicase activation, in addition to the newly established role of Mcm10 in bridging convergent CMG dimers.

Our data suggest that the DNSN-1 homodimer contributes to helicase activation in *C. elegans*, in addition to SLD-2 and MCM-10. Partial depletion of DNSN-1 is lethal in worms lacking MCM-10 (Xia et al, 2023). Moreover, DNSN-1 accumulates with inactive CMG helicase complexes on chromatin in embryos lacking MCM-10 and SLD-2 (Fig. 3D,E). In contrast, TIM-1 does not associate with inactive CMG helicase complexes on chromatin in *C. elegans* embryos lacking MCM-10 and SLD-2 (Fig. 3F), despite the fact that both DNSN-1 and TIM-1 can bind simultaneously to individual CMG helicase complexes (Xia et al, 2023). These data suggest that inactive CMG complexes comprise head-to-head dimers that are held together by the DNSN-1 dimer, in a register that excludes the TIM-1_TIPN-1 complex that would normally bind to the front of the CMG helicase at active replication forks (Xia et al, 2023). Correspondingly, dimeric *Xenopus* DONSON was previously shown to stabilise a head-to-head dimer of CMG (Cvetkovic et al, 2023), in which the two helicases associate with a similar register to that stabilised by yeast Mcm10 during the initiation of DNA replication (preprint: Palm et al, 2026). Moreover, a recent study showed that a similar head-to-head dimer of recombinant human CMG complexes can be assembled off DNA in vitro, in the presence of dimeric DONSON, facilitating the interaction of dimeric CMG with MCM10, RECQL4 and Pol ε (preprint: Taylor-Cross et al, 2026). Despite the absence of DONSON in budding yeast and other fungi, these finding suggest fundamental similarities in the mechanism of CMG helicase activation in diverse eukaryotic species.

We find that MCM-10, SLD-2 and DNSN-1 all stimulate CMG helicase activity in vitro (Fig. 4A–G), with the effect of SLD-2 being additive with either MCM-10 or DNSN-1 (Fig. 4H). MCM-10, SLD-2 and DNSN-1 all bind to CMG and DNA (Figs. 2 and EV2–4) and both properties are important for the stimulation of CMG helicase activity in the case of DNSN-1 (Figs. EV3 and 4). Further structural studies, together with the reconstitution of metazoan helicase activation from MCM2-7 double hexamers, will likely be required to resolve the mechanism by which MCM-10, SLD-2 and DNSN-1 stimulate CMG helicase activity, together with the relevance of such stimulation to CMG activation during the initiation of chromosome replication.

The data in this study indicate that SLD-2 is dispensable for CMG helicase assembly and instead is important for helicase activation (Fig. 3). Nevertheless, relatively little CMG persists on chromatin during mitosis of the first embryonic cell cycle in the absence of SLD-2, even when CMG helicase disassembly by the CDC-48_UFD-1_NPL-4 unfoldase has been inactivated (Sonneville et al, 2017). This issue requires further investigation, for example to determine whether inactive double-CMG complexes at origins are inherently less stable than double hexamers of MCM2-7 or individual CMG complexes at replication forks.

We show that mouse ES cells can proliferate in the complete absence of MCM10 (Appendix Figs. S6 and S12), mirroring the situation in *C. elegans* (Xia et al, 2023). This indicates the existence of MCM10-independent routes to CMG helicase activation, likely involving RECQL4 and DONSON. Mouse cells that completely lack MCM10 grow more slowly than control cells, with a delay in helicase activation (Appendix Fig. S12) that probably explains why MCM10 becomes essential in some mammalian cell cycles, for example during mouse development (Lim et al, 2011), or in human cancer cell lines (Chattopadhyay and Bielinsky, 2007). In human patients, hypomorphic mutations in MCM10 are associated with a condition called Natural Killer Cell Deficiency, or alternatively with restrictive cardiomyopathy associated with hypoplasia of the spleen and thymus (Baxley et al, 2021; Mace et al, 2020; Schmit et al, 2024).

Our data indicate that RECQL4 plays a partially redundant role with MCM10 during CMG activation in mammalian cells (Figs. 5–8), analogous to the situation in *C. elegans*. Moreover, our findings suggest that inactivation of RECQL4 produces a greater defect in CMG helicase activation in mouse ES cells than inactivation of MCM10 (Appendix Figs. S6, S7, S10C,D and S12). This also resembles the situation in *C. elegans*, for which *mcm-10*∆ is viable (Xia et al, 2023), whereas absence of SLD-2 delays CMG activation and leads to the failure of nuclear division in the second cell cycle (Appendix Fig. S1C). In the absence of both RECQL4 and MCM10, inactive CMG complexes accumulate on chromatin, without a comparable accumulation of TIMELESS-TIPIN or RPA (Figs. 8D,E,G (iii) and EV5A–C; Appendix Fig. S11E), suggesting that CMG remains as an inactive head-to-head dimer, consistent with the phenotype of *C. elegans* embryos that lack MCM-10 and SLD-2.

In line with previous findings in human cells (Padayachy et al, 2024), we were able to generate viable cells with small deletions in the mouse *Recql4* gene, which removed detectable expression of RECQL4 protein (Fig. 5A,B; Appendix Fig. S5D). However, complete deletion of the *Recql4* gene was only obtained in a heterozygous form in mouse ES cells (Appendix Fig. S7). Recent work indicated that RECQL4 is essential in immortalised mouse myeloid cells, and an amino-terminal fragment of mouse RECQL4, which contains the regions with homology to yeast Sld2 and worm SLD-2, is sufficient to preserve viability (Buco et al, 2026). These data are consistent with past work showing that viable mice can be obtained with deletions within the helicase domain of RECQL4 (Hoki et al, 2003; Mann et al, 2005), though these mice show defects in skin and skeleton, and have an increased rate of aneuploidy and cancer prevalence. In humans, mutations that truncate RECQL4 within or after the helicase domain are associated with diseases such as Rothmund-Thompson syndrome and Rapadilino syndrome, with a range of developmental defects and an increased rate of cancers such as osteosarcoma (Kitao et al, 1999; Siitonen et al, 2003; Wang et al, 2003).

While this work was under review, two additional studies reported that MCM10 and RECQL4 play partially redundant roles during CMG helicase activation in extracts of *Xenopus laevis* eggs (preprint: Terui et al, 2025) and in human cells (Bektash et al, 2026). In both cases, single depletion of RECQL4 was found to inhibit DNA replication in vitro to a greater degree than depletion of MCM10, consistent with our findings in *C. elegans* and mammalian cells. All together, these findings indicate a strongly conserved role for MCM10 and RECQL4/SLD-2 during DNA replication initiation in animal cells.

Finally, we note that CDK phosphorylation of both *C. elegans* SLD-2 (Gaggioli et al, 2014) and human RECQL4 (Shin et al, 2019; Thakur et al, 2025) has been reported to regulate the initiation of DNA replication. Since SLD-2 and RECQL4 are dispensable for CMG assembly and instead promote helicase activation, these findings challenge our view of how CDK controls chromosome replication during DNA replication initiation in eukaryotes. The possibility that CDK not only controls assembly of the CMG helicase complex but also regulates its subsequent activation merits further investigation.

## Methods

**Table:** Reagents and tools table

Reagent/resource	Reference or Source	Identifier or catalogue number
**Experimental models**		
*Caenorhabditis elegans*	N2	N/A
*C. elegans:* Strain *mcm-10*∆:*mcm-10* ∆ *(syb2118) III*	(Xia et al, 2023)	KAL259
*C. elegans:* Strain *GFP-PSF-1;mCherry-H2B psf-1(lab1[gfp::TEV::S-tag::psf-1 + loxP unc-119(+) loxP]) II; ltIs37[pie-1p::mCherry::his-58 + unc-119(* + *)] IV*	(Sonneville et al, 2017)	KAL395
*C. elegans:* Strain *GFP-PSF-1;mCherry-H2B mcm-10∆ psf-1(lab1[gfp::TEV::S-tag::psf-1 + loxP unc-119(+) loxP]) II; mcm-10 ∆ (syb2118) III ; ltIs37[pie-1p::mCherry::his-58 + unc-119(* + *)] IV*	This study	KAL341
*C. elegans:* Strain *SLD-2-GFP sld-2(syb6453 [sld-2::GFP]) I*	This study	KAL282
*C. elegans:* Strain *SLD-2-GFP; mcm-10∆ sld-2(syb6453[sld-2::GFP])I; mcm-10 ∆ (syb2118) III*	This study	KAL417
*C. elegans:* Strain *SLD-2-GFP; mCherry-H2B sld-2(syb6453 [sld-2::GFP]) I; ltIs37[pie-1p::mCherry::his-58 + unc-119(+)]*	This study	KAL270
*C. elegans:* Strain *SLD-2-GFP; mCherry-PSF-1 sld-2(syb6453 [sld-2::GFP]) I; psf-1(syb6425 [mCherry::psf-1]) II*	This study	KAL272
*C. elegans:* Strain *SLD-2-GFP; mCherry-H2B; mcm-10∆ sld-2(syb6453 [sld-2::GFP]) I; mcm-10 ∆ (syb2118) III; ltIs37[pie-1p::mCherry::his-58 + unc-119(+)]:mCherry::his-58 + unc-119(* + *)] IV*	This study	KAL333
*C. elegans:* Strain *SLD-2-GFP; mCherry-CDC-45; mcm-10∆ sld-2(syb6453 [sld-2::GFP]) I; evl-18(syb6509 [mCherry::cdc-45]) III; mcm-10 ∆ (syb2118) III*	This study	KAL336
*C. elegans:* Strain *SLD-2-GFP; mCherry-PSF-1; mcm-10∆ sld-2(syb6453 [sld-2::GFP]) I; psf-1(syb6425 [mCherry::psf-1]) II; mcm-10 ∆ (syb2118) III*	This study	KAL335
*C. elegans:* Strain *SLD-2-GFP; mCherry-RPA-2; mcm-10∆ sld-2(syb6453[sld-2::GFP])I ;rpa-2(syb6422[mCherry::rpa-2]) I; mcm-10(sub2118[-8226bp])III*	This study	KAL403
*C. elegans:* Strain *GFP-MCM-10; mCherry-H2B mcm-10(syb6364[GFP::mcm-10]) III; ltIs37[pie-1p::mCherry::his- 58 + unc-119(* + *)] IV*	This study	KAL407
*C. elegans:* Strain *GFP-DNSN-1; mCherry-H2B dnsn-1(syb5085 [gfp::dnsn-1]) II; ltIs37[pie-1p::mCherry::his-58 + unc-119(+)]:mCherry::his-58 + unc-119(* + *)] IV*	(Xia et al, 2023)	KAL267
*C. elegans:* Strain *GFP-sld-2; tim-1-mCherry; mcm-10∆ sld-2(syb6453[sld-2::GFP])I; mcm-10∆ (syb2118) III; tim-1(syb11653 [tim-1::mCherry]) III;*	This study	KAL458
*Escherichia coli*: Rosetta™ (DE3) pLysS cells: F^-^ *ompT hsdS*_*B*_(r_B_^-^ m_B_^-^) *gal dcm* (DE3) pLysSRARE (Cam^R^)	Novagen	70956
E14tg2A mouse ES cells		N/A
E14tg2A *BromoTag-Donson*	Evrin et al, 2023	N/A
E14tg2A *BromoTag-Donson GFP-Psf1*	This study	N/A
E14tg2A *BromoTag-Mcm10 dTAG-Recql4*	This study	N/A
E14tg2A *BromoTag-Mcm10 dTAG-Recql4 GFP-Psf1*	This study	N/A
E14tg2A *BromoTag-Mcm10 dTAG-Recql4 GFP-Psf1 mCherry-Timeless*	This study	N/A
E14tg2A *BromoTag-Mcm10 dTAG-Recql4 Rosa26::Recql4*	This study	N/A
E14tg2A *BromoTag-Mcm10 dTAG-Recql4 Rosa26::Recql4 1–468*	This study	N/A
E14tg2A *GFP-Psf1*	This study	N/A
E14tg2A *Mcm10∆*	This study	N/A
E14tg2A *Mcm10∆* (deletion of all gene)	This study	N/A
E14tg2A *Recql4∆*	This study	N/A
*S. cerevisiae*: Strain *yJF1*: *MAT***a** *ade2-1 ura3-1 his3-11,15 trp1-1 leu2-3,112 can1-100 bar1∆::hphNT pep4∆::kanMX*	(Frigola et al, 2013)	N/A
*S. cerevisiae*: Strain *yYX37*(CMG purification): *MATα/ MAT***a** *ade2-1 / ade2-1 ura3-1 URA3::pRS306-mcm-2-GAL1,10-mcm-3 / ura3-1 URA3::pRS306-TAP-psf-1-GAL1,10-sld-5 his3-11,15 / his3-11,15 HIS3::pRS303-GAL1,10-cdc-45 trp1-1 TRP1::pRS304-mcm-6-GAL1,10-mcm-7 / trp1-1 leu2-3,112 LEU2::pRS305-mcm-4-GAL1,10-mcm-5 / leu2-3,112 LEU2::pRS305-psf-3-GAL1,10-psf-2 can1-100 / can1-100 pep4∆::ADE2 / pep4∆::ADE2*	(Xia et al, 2021)	N/A
*S. cerevisiae: Strain yYX36(GINS and CDC-45 expression): MATa ade2-1 ura3-1 URA3::pRS306-TAP-psf-1-GAL1,10-sld-5 his3-11,15 HIS3::pRS303-GAL1,10-cdc-45 trp1-1 leu2-3,112 LEU2::pRS305-psf-3-GAL1,10-psf-2 can1-100 pep4∆::ADE2*	(Xia et al, 2021)	N/A
*S. cerevisiae*: Strain *yYX1*(TIM-1_TIPN-1 purification): *MAT***a** *ade2-1 ura3-1 his3-11,15 trp1-1 leu2-3,112 LEU2::pRS305-CBP-PreScission-tipn-1-GAL1,10-tim-1 can1-100 pep4∆::ADE2*	(Xia et al, 2021)	N/A
*S. cerevisiae*: Strain yYX40(CLSP-1 purification): MATa ade2-1 ura3-1 his3-11,15 HIS3::pRS303-GAL1,10-clsp-1-TEV-5FLAG trp1-1 leu2-3,112 can1-100 pep4∆::ADE2	(Xia et al, 2021)	N/A
*S. cerevisiae*: Strain yYX41(CTF-4 purification): MATaade2-1 ura3-1 his3-11,15 HIS-3::pRS303-GAL1,10-5FLAG-PreScission-ctf-4 trp1-1 leu2-3,112 can1-100 pep4∆::ADE2	(Xia et al, 2021)	N/A
*S. cerevisiae*: Strain yYX48(CTF-18_RFC purification):MATa/MATa ade2-1 / ade2-1 ura3-1 URA3::pRS306-dscc-1-GAL1,10-ctf-8 / ura3-1 his3-11,15HIS3::pRS303-GAL1,10-ProteinA-3TEV-ctf-18/his3-11,15 trp1-1 / trp1-1 TRP1::pRS304-rfc-4-GAL1,10-rfc-5leu2-3,112 / leu2-3,112 LEU2::pRS305-rfc-2-GAL1,10-rfc-3 can1-100 / can1-100 pep4∆::ADE2/pep4∆::ADE2	(Xia et al, 2021)	N/A
*S. cerevisiae*: Strain yYX77(RPA purification): MATa ade2-1 ura3-1 his3-11,15 HIS3::pRS303-rpa-1-GAL1,10-ProteinA-TEV-rpa-2 trp1-1 leu2-3,112 can1-100 pep4∆::ADE2	This study	N/A
*S. cerevisiae*: Strain yYX84(MUS-101 purification): MATa ade2-1 ura3-1 his3-11,15 HIS3::pRS303-GAL1,10-ProteinA-TEV-mus-101 trp1-1 leu2-3,112 can1-100 pep4∆::ADE2	This study	N/A
*S. cerevisiae*: Strain yYX93(SLD-2 purification): MATa ade2-1 ura3-1 his3-11,15 HIS3::pRS303-GAL1,10-ProteinA-TEV-sld-2 trp1-1 leu2-3,112 can1-100 pep4∆::ADE2	This study	N/A
*S. cerevisiae*: Strain yYX97(MCM-10 purification): MATa ade2-1 ura3-1 his3-11,15 HIS3::pRS303-GAL1,10-ProteinA-TEV-mcm-10 trp1-1 leu2-3,112 can1-100 pep4∆::ADE2	This study	N/A
**Recombinant DNA**		
*no RNAi, Control*		DU70356
*GFP* RNAi: L4440-GFP	(Xia et al, 2023)	DU75877
*rnr-1* RNAi: L4440-rnr-1	(Sonneville et al, 2015)	DU83608
*sld-2* + *GFP* RNAi: L4440-cdc-45 + GFP	This study	DU76891
*sld-2+cdc-45* RNAi: L4440-cdc-45+sld-2	This study	DU83606
*cdc-45* RNAi: L4440-cdc-45	(Sonneville et al, 2012)	DU75883
*sld-2* RNAi: L4440-sld-2	(Xia et al, 2023)	DU75882
*npl-4* RNAi: L4440-npl-4	(Sonneville et al, 2017)	DU70399
*GINS* RNAi: L4440-sld-5+psf-1	This study	DU83607
pFGET19-Ulp1(403-621)	Addgene	64697
pYXC1 [expresses TIM-1 and CBP-PreScission-TIPN-1, for TIM-1_TIPN-1 purification]	(Xia et al, 2021)	DU70332
pYXC14 [expresses MCM-2a and MCM-3, for CMG purification]	(Xia et al, 2021)	DU70334
pYXC15 [expresses MCM-4a and MCM-5, for CMG purification]	(Xia et al, 2021)	DU70335
pYXC17 [expresses CDC-45, for CMG purification]	(Xia et al, 2021)	DU70336
pYXC19 [expresses PSF-3 and PSF-2, for CMG purification]	(Xia et al, 2021)	DU70337
pYXC28 [expresses MCM-6a and MCM-7, for CMG purification]	(Xia et al, 2021)	DU70338
pYXC29 [expresses TAP-PSF-1 and SLD-5a, for CMG purification]	(Xia et al, 2021)	DU70339
pYXC35 [expresses CLSP-1a-TEV-5FLAG, for CLSP-1a purification]	(Xia et al, 2021)	DU70342
pYXC36 [expresses 5FLAG-PreScission-CTF-4, for CTF-4 purification]	(Xia et al, 2021)	DU70343
pYXC37 [expresses PrA-3TEV-CTF-18, for CTF-18_RFC purification]	(Xia et al, 2021)	DU70344
pYXC38 [expresses DSCC-1 and CTF-8, for CTF-18_RFC purification]	(Xia et al, 2021)	DU70345
pYXC39 [expresses RFC-2 and RFC-3, for CTF-18_RFC purification]	(Xia et al, 2021)	DU70346
pYXC40 [expresses RFC-4 and RFC-5, for CTF-18_RFC purification]	(Xia et al, 2021)	DU70347
pYXC54 [expresses ProteinA-TEV-MCM-10, for MCM-10 purification]	This study	N/A
pYXC103 [expresses RPA-1 and ProteinA-TEV-RPA-2, for RPA purification]	This study	N/A
pYXC106 [expresses 14HIS-SUMO-DNSN-1c, for DNSN-1c purification]	(Xia et al, 2023)	DU75873
pYXC112 [expresses 14HIS-SUMO-*dnsn-1c 1–20∆*, for DNSN-1c ∆N purification]	(Xia et al, 2023)	DU75875
pYXC116 [expresses ProteinA-TEV-MUS-101, for MUS-101 purification]	This study	N/A
pYXC117 [expresses 14HIS-SUMO-*dnsn-1c 3A(W425AI429AV431A)*, for DNSN-1c 3 A purification]	This study	N/A
pYXC128 [expresses ProteinA-TEV-SLD-2, for SLD-2 purification]	This study	N/A
pYXC132 [expresses 14HIS-SUMO-*dnsn-1c 8A(K63AK64AR65AK67AR101AK102AR103AK104A)*, for DNSN-1c 8 A purification]	This study	N/A
pYXC133 [expresses 14HIS-SUMO-*dnsn-1c 8E(K63EK64ER65EK67ER101EK102ER103AK104E)*, for DNSN-1c 8E purification]	This study	N/A
pCE330 [pKN7 gRNA2 *Mcm10* Exon1]	MRC PPU Reagents and Services (This study)	DU78843
pCE427 [pX335 gRNA1b *Mcm10* Exon 1]	MRC PPU Reagents and Services (This study)	DU78842
pCE446 [donor vector dTAG-*Recql4*]	MRC PPU Reagents and Services (This study)	DU78841
pCE459 [donor vector BromoTag-*Mcm10*]	MRC PPU Reagents and Services (This study)	DU78840
pCE460 [pX335 gRNA1 *Mcm10* Exon 3 pair 1]	MRC PPU Reagents and Services (This study)	DU78839
pCE461 [pKN7 gRNA2 *Mcm10* Exon 3 pair 1]	MRC PPU Reagents and Services (This study)	DU78838
pCE464 [pX335 gRNA1 *Mcm10* Exon 3 pair 3]	MRC PPU Reagents and Services (This study)	DU78837
pCE465 [pKN7 gRNA2 *Mcm10* Exon 3 pair 3]	MRC PPU Reagents and Services (This study)	DU78836
pCE476 [donor vector GFP-PSF1 – Blasticidin]	MRC PPU Reagents and Services (This study)	DU83251
pCE488 [RECQL4 WT for integration at *Rosa26* locus]	MRC PPU Reagents and Services (This study)	DU83248
pCE494 [donor vector GFP-PSF1]	MRC PPU Reagents and Services (This study)	DU83180
p3481 [donor vector BromoTag-DONSON - Hygromycin]	MRC PPU Reagents and Services Evrin et al, 2023	DU75983
pCE501 [pX459 gRNA Exon 1 *Recql4*]	MRC PPU Reagents and Services (This study)	DU76713
pCE502 [pX459 gRNA Exon 22 *Recql4*]	PRC PPU Reagents and Services (This study)	DU76714
pCE507 [pX459 gRNA Exon 1 *Mcm10*]	MRC PPU Reagents and Services (This study)	DU83252
pCE508 [pX459 gRNA Exon 20 *Mcm10*]	MRC PPU Reagents and Services (This study)	DU83253
pCE518 [RECQL4 1–468 for integration at *Rosa26* locus]	MRC PPU Reagents and Services (This study)	DU76886
pCE519 [donor vector mCherry-TIMELESS – Neomycin]	MRC PPU Reagents and Services (This study)	DU76885
pJA14 [pX335 gRNA1 Timeless Exon1]	MRC PPU Reagents and Services (This study)	DU76884
pJA15 [pKN7 gRNA2 Timeless Exon1]	MRC PPU Reagents and Services (This study)	DU76883
pKN7 [expression vector for gRNA and Puromycin-resistance gene]	MRC PPU Reagents and Services	DU70250
pKN88 [pKN101 gRNA *Rosa26*]	MRC PPU Reagents and Services (Villa et al, 2021)	DU70256
pKN101 [expression vector for gRNA, Cas9-D10A ‘nickase’ and Puromycin-resistance gene]	MRC PPU Reagents and Services (Villa et al, 2021)	DU70263
pLP1 [pX335 gRNA1 *Recql4* Exon 1]	MRC PPU Reagents and Services (This study)	DU78835
pLP2 [pKN7 gRNA2 *Recql4* Exon 1]	MRC PPU Reagents and Services (This study)	DU78834
pX335 [expression vector for gRNA and Cas9-D10A ‘nickase’]	Pyzocha et al, 2014	Addgene
pX459 v2.0 [expression vector for gRNA, 3FLAG-Cas9-D10A-2A-Puromycin resistance]	MRC PPU Reagents and Services (Ran et al, 2013)	DU70729 Addgene
**Antibodies**		
Anti-Mouse IgG [from goat] conjugated to Alexa Fluor 546 Use 1 in 1000 for immunofluorescence	Invitrogen	A-11030
Anti-Rabbit IgG [from donkey] conjugated to Alexa Fluor 488 Use 1 in 1000 for immunofluorescence	Invitrogen	A-21206
anti-Sheep IgG HRP [from donkey] Use 1 in 10,000 for immunoblotting	Sigma-Aldrich	A3415
*C. elegans* CDC-45 [antigen 1–222, sheep polyclonal] Use 1 in 500 for immunoblotting	MRC PPU Reagents and Services (Sonneville et al, 2017)	DU49540
*C. elegans* MCM-2 [antigen 1–222, sheep polyclonal] Use 1 in 2000 for immunoblotting	MRC PPU Reagents and Services (Sonneville et al, 2017)	DU49542
*C. elegans* MCM-6 [antigen 589–810, sheep polyclonal] Use 1 in 1000 for immunoblotting	MRC PPU Reagents and Services (Xia et al, 2021)	DU57531
*C. elegans* MCM-10 [antigen 1–222, sheep polyclonal] Use 1 in 500 for immunoblotting	MRC PPU Reagents and Services (This study)	DU65471
*C. elegans* SLD-2 [antigen 1–249, sheep polyclonal] Use 1 in 500 for immunoblotting	MRC PPU Reagents and Services (This study)	DU72919
*C. elegans* SLD-5 [antigen 1–222, sheep polyclonal] Use 1 in 500 for immunoblotting	MRC PPU Reagents and Services (Xia et al, 2021)	DU57533
Human CDC45 [rabbit monoclonal] Use 1 in 50 for immunofluorescence	Cell Signaling Technology	11881
Human CHK1 (DCS-310) [mouse monoclonal] Use 1 in 1000 for immunoblotting	Santacruz	Sc-56291
Human MCM2 [mouse monoclonal] Use 1 in 250 for immunofluorescence	BD Biosciences	610701
Human RPA2/RPA32 (9H8) [mouse monoclonal] Use 1 in 100 for immunofluorescence	Abcam	Ab2175
Mouse CDC45 [antigen 1–222; sheep polyclonal] Use 1 in 1000 for immunoblotting	MRC PPU Reagents and Services (Villa et al, 2021)	DU35753
Mouse DONSON [antigen 1–222; sheep polyclonal] Use 1 in 1000 for immunoblotting	MRC PPU Reagents and Services (Evrin et al, 2023)	DU62042
Mouse MCM6 [antigen 1–222; sheep polyclonal] Use 1 in 5000 for immunoblotting	MRC PPU Reagents and Services (Villa et al, 2021)	DU51793
Mouse MCM10 [antigen 375–696; sheep polyclonal] Use 1 in 1000 for immunoblotting	MRC PPU Reagents and Services (This study)	DU27961
Mouse PSF1 [antigen 1–163; sheep polyclonal] Use 1 in 1000–3000 for immunoblotting	MRC PPU Reagents and Services (Villa et al, 2021)	DU51938
Mouse PSF2 [antigen 1–185; sheep polyclonal] Use 1 in 1000–3000 for immunoblotting	MRC PPU Reagents and Services (Villa et al, 2021)	DU51832
Mouse PSF3 [antigen 1–216; sheep polyclonal] Use 1 in 1000–3000 for immunoblotting	MRC PPU Reagents and Services (Villa et al, 2021)	DU24601
Mouse RECQL4 [antigen 1–222; sheep polyclonal] Use 1 in 1000 for immunoblotting	MRC PPU Reagents and Services (This study)	DU27956
Mouse SLD5 [antigen 1–223; sheep polyclonal] Use 1 in 3000 for immunoblotting	MRC PPU Reagents and Services (Villa et al, 2021)	DU24572
Phospho CHK1 (S345) [rabbit polyclonal] Use 1 in 1000 for immunoblotting	Cell signaling	2341
Phospho RPA32 (S4/8) [rabbit polyclonal] Use 1 in 200 for immunofluorescence	Bethyl laboratories Thermo Fisher	A300-245A
Phospho RPA32 (S33) [rabbit polyclonal] Use 1 in 1000 for immunoblotting	Bethyl laboratories ThermoFisher	A300-246A
**Oligonucleotides and other sequence-based reagents**		
*5’ oligo to make annealed substrate with 47 bp double-strand fork DNA for helicase assay and with 46 bp double-strand DNA and 39nt of single-strand DNA (3’ flap) for in vitro CMG IP* TAGAGTAGGAAGTGATGGTAAGTGATTAGAGAATTGGAGAGTGTGTTTTTTTTTTTTTTTTTTTTTTTTTTTTTTTTTTT*T*T*T*T*T [* denotes phosphorothioate bonds]	(Deegan et al, 2020)	N/A
*3’ oligo to make annealed substrate with 46 bp double-strand DNA and 39nt of single-strand DNA (3’ flap) for in vitro CMG IP* *ACACACTCTCCAATTCTCTAATCACTTACCATCACTTCCTACTCTA*	(Deegan et al, 2020)	N/A
*3’ oligo to make annealed substrate with 47 bp double-strand fork DNA for helicase assay* *GGCAGGCAGGCAGGCAGGCAGGCAGGCAGGCAGGCAGGCAACACACTCTCCAATTCTCTAATCACTTACCATCACTTCCTACTCTA*	This study	N/A
*3’ oligo (Cy5 labelled at 5’ end) to make annealed substrate with 47 bp double-strand fork DNA for helicase assay and DNA binding assay* *[Cy5]GGCAGGCAGGCAGGCAGGCAGGCAGGCAGGCAGGCAGGCAACACACTCTCCAATTCTCTAATCACTTACCATCACTTCCTACTCTA*	This study	N/A
*5’ oligo to make single-strand DNA or annealed substrate with 80 bp double-strand DNA for DNA binding assay* *[Cyanine5]CAGTGCCAAGCTTGCATGCCTGCAGGTCGACTCTAGAGGATCCCCGGGTACCGAGCTCGAATTCGTAATCATGGTCATAG*	This study	N/A
*3’ oligo to make annealed substrate with 80 bp double-stranded DNA for DNA binding assay* *CTATGACCATGATTACGAATTCGAGCTCGGTACCCGGGGATCCTCTAGAGTCGACCTGCAGGCATGCAAGCTTGGCACTG*	This study	N/A
*Poly-dT oligo for helicase assay* TTTTTTTTTTTTTTTTTTTTTTTTTTTTTTTTTTT*T*T*T*T*T [* denotes phosphorothioate bonds]	This study	N/A
*5’ oligo to make ‘ds DNA with two 3’ flaps’ as shown in* Appendix Fig. S4A TAAGATTTTTTTCTTTGATAAATTCTTGTTTTCATATCCTAAAATTAAAGGGAAAATAAACAATACATAACAAAACATATAAAAACCATTTTTTTTTTTTTTTTTTTTTTTTTTTTTTTTTTT*T*T*T*T*T [* denotes phosphorothioate bonds]	This study	N/A
*3’ oligo to make ‘ds DNA with two 3’ flaps’, as shown in* Appendix Fig. S4A TGGTTTTTATATGTTTTGTTATGTATTGTTTATTTTCCCTTTAATTTTAGGATATGAAAACAAGAATTTATCAAAGAAAAAAATCTTATTTTTTTTTTTTTTTTTTTTTTTTTTTTTTTTTTT*T*T*T*T*T [* denotes phosphorothioate bonds]	This study	N/A
*‘Trap DNA’ shown in* Appendix Fig. S4A. *TTTTTTTTTTTTTTTTTTTTTTTTTTTTTTTTTTTTTTTTTGGTTTTTATATGTTTTGTTATGTATTGTTTATTTTCCCTTTAATTTTAGGATATGAAAACAAGAATTTATCAAAGAAAAAAATCTTA*	This study	N/A
*3’ oligo to make ‘ds DNA with one 3’ flap’, as shown in* Appendix Fig. S4A *TGGTTTTTATATGTTTTGTTATGTATTGTTTATTTTCCCTTTAATTTTAGGATATGAAAACAAGAATTTATCAAAGAAAAAAATCTTA*	This study	N/A
Mouse *Mcm10* Exon 1 gRNA1b_oligo 1, 10344: caccgAGCTGAGATGACAAGAATAG	This study	N/A
Mouse *Mcm10* Exon 1 gRNA1b_oligo 2, 10345: aaacCTATTCTTGTCATCTCAGCTc	This study	N/A
Mouse *Mcm10* Exon1 gRNA2_oligo 1, 9894: caccgGGTAAGGCCTTCTAGTTTTA	This study	N/A
Mouse *Mcm10* Exon1 gRNA2_oligo 2, 9895: aaacTAAAACTAGAAGGCCTTACCc	This study	N/A
Mouse *Mcm10* Exon 3 gRNA1_oligo 1, pair 3, 10586: caccgAGAGCTCATCAAATTCATCA	This study	N/A
Mouse *Mcm10* Exon 3 gRNA1_oligo 2, pair 3 10587: aaacTGATGAATTTGATGAGCTCTc	This study	N/A
Mouse *Mcm10* Exon 3 gRNA2_oligo 1, pair 3, 10588: caccgGAGTCTTACACAGAAGAGGC	This study	N/A
Mouse *Mcm10* Exon 3 gRNA2_oligo 2, pair 3, 10589: aaacGCCTCTTCTGTGTAAGACTCc	This study	N/A
Mouse *Mcm10* Exon 20 gRNA2_oligo 1, 10128: caccgCTCTTCAGACACCGCCCAAC	This study	N/A
Mouse *Mcm10* Exon 20 gRNA2_oligo 2, 10129: aaacGTTGGGCGGTGTCTGAAGAGc	This study	N/A
Mouse *Recql4* Exon 1 gRNA1_oligo 1, 10146: caccgCAGCCCGAACGTGAGTTTAG	This study	N/A
Mouse *Recql4* Exon 1 gRNA1_oligo 2, 10147: aaacCTAAACTCACGTTCGGGCTGc	This study	N/A
Mouse *Recql4* Exon 1 gRNA2_oligo 1, 10142: caccgGACCGTTCGCGCGCGGCTAC	This study	N/A
Mouse *Recql4* Exon 1 gRNA2_oligo 2, 10143: aaacGTAGCCGCGCGCGAACGGTCc	This study	N/A
Mouse *Recql4* Exon 22 gRNA2_oligo 1, 10601: caccgGATGACCACCTTACATGGGA	This study	N/A
Mouse *Recql4* Exon 22 gRNA2)oligo2, 10602: aaacTCCCATGTAAGGTGGTCATCc	This study	N/A
Mouse *Timeless* Exon 1 gRNA1_oligo 1, 8832: caccgCTAGCCACGTGTAGCGCCCT	This study	N/A
Mouse *Timeless* Exon 1 gRNA1_oligo 2, 8833: aaacAGGGCGCTACACGTGGCTAGc	This study	N/A
Mouse *Timeless* Exon 1 gRNA2_oligo 1, 8834: caccgTGTACAAGTCCATACACCAG	This study	N/A
Mouse *Timeless* Exon 1 gRNA2_oligo 2, 8835: aaacCTGGTGTATGGACTTGTACAc	This study	N/A
M13 Forward: GTAAAACGACGGCCAGT		N/A
M13 reverse: GGAAACAGCTATGACCATG		N/A
**Chemicals, enzymes and other reagents**		
*C. elegans* CMG helicase (including MCM-2 isoform a, MCM-4 isoform a, MCM-6 isoform a and SLD-5 isoform a; other components only have one isoform)	(Xia et al, 2021)	N/A
*C. elegans* GINS	(Xia et al, 2023)	N/A
*C. elegans* CLSP-1	(Xia et al, 2021)	N/A
*C. elegans* CTF-4	(Xia et al, 2021)	N/A
*C. elegans* CTF-18_RFC	(Xia et al, 2021)	N/A
*C. elegans* DNSN-1	(Xia et al, 2023)	N/A
*C. elegans* DNSN-1 DN	(Xia et al, 2023)	N/A
*C. elegans* DNSN-1 3 A	This study	N/A
*C. elegans* DNSN-1 8E	This study	N/A
*C. elegans* DNSN-1 8 A	This study	N/A
*C. elegans* MCM-10	This study	N/A
*C. elegans* MUS-101	This study	N/A
*C. elegans* RPA	This study	N/A
*C. elegans* SLD-2	This study	N/A
*C. elegans* TIM-1_TIPN-1	(Xia et al, 2021)	N/A
AcTEV	Thermo Fisher Scientific	12575015
AEBSF	Sigma-Aldrich	A8456
AGB1	Tocris	7686
Basal Medium Eagle vitamins	Thermo Fisher Scientific	21010046
β-mercaptoethanol	Sigma-Aldrich	M6520
4–12% Bis-Tris NuPAGE	Life Technologies	WG1402BOX
Blasticidin	Invitrogen	ant-bl-10p
Calmodulin sepharose 4B	GE Healthcare	17052901
CB-5083	Selleckchem	S8101
Cell culture plates, 96-well, CELLSTAR with µClear bottom	Greiner	655090
Chemically defined lipid concentrate	Thermo Fisher Scientific	11905031
Chromatography paper	GE Healthcare	3030861
Chromotek GFP-Trap Magnetic Particles M-270	Thermo Fisher Scientific	17353353
Clear-bottomed black wall 96-well plates	Greiner	655090
Click-iT EdU Imaging Kit	Thermo Fisher Scientific Inc.	B10184
Concentrated penicillin-streptomycin	Thermo Fisher Scientific	11568876
Cytivia MicroSpin G-50 columns	Thermo Fisher Scientific	27533001
Crystal violet	Sigma-Aldrich	HT90132
DAPI	Thermo Fisher Scientific	62248
DMEM	Thermo Fisher Scientific	11960044
dTAG13	MRC PPU Reagents and Services	N/A
Dynabeads M-270 Epoxy	Thermo Fisher Scientific	14302D
5-ethynyl-2’-deoxyuridine (EdU)	Thermo Fisher Scientific Inc.	E10187
FBS	LabTech	FCS-SA/500
Bovine FCS	Thermo Fisher Scientific	A5256701
Formaldehyde	Thermo Scientific	11586711
Formaldehyde 4% stabilised, buffered	VWR	9713.1000
Formaldehyde 16% (w/v), Methanol free	Thermo Fisher Scientific	28908
Gelatin	Sigma-Aldrich	G1890
Glass beads	Stratech	11079105
L-Glutamine	Thermo Fisher Scientific	25030081
Hoescht 33342	Sigma-Aldrich	B2261
Hydroxyurea	Molekula	10872383
Hygromycin	Invitrogen	ant-hg-5
iBlot membrane	Thermo Fisher Scientific	IB301031
IgG sepharose 6 Fast Flow	GE Healthcare	17096901
Inulin	Sigma-Aldrich	I2255
Knockout serum replacement	Thermo Fisher Scientific	10828028
LIF	MRC PPU Reagents and Services	DU1715
Neomycin (G418)	Formedium	G418S
Ni-NTA agarose	Qiagen	30210
Non-essential amino acids	Thermo Fisher Scientific	11140050
Novex Hi-Density TBE Sample Buffer	Thermo Fisher Scientific	LC6678
4%-20% Novex TBE gels	Thermo Fisher Scientific	EC62252BOX
4x NuPAGE LDS sample buffer	Thermo Fisher Scientific	NP0007
PEI	Polysciences, Inc	24765-2
Penicillin-Streptomycin	Thermo Fisher Scientific	15140122
Pepstatin A	Sigma-Aldrich	P5318
Pierce Universal Nuclease	Thermo Fisher Scientific	88702
PreScission protease	MRC PPU Reagents & Services	DU34905
PrimeStar Hot Start DNA polymerase	Takara Bio	R010A
Propidium iodide	Sigma-Aldrich	P4170
Protease Inhibitor Cocktail	Sigma-Aldrich	P8215
Puromycin	Thermo Fisher Scientific	A1113802
RNase A	Thermo Fisher Scientific	EN0531
Roche cOmplete EDTA-free protease inhibitor cocktail	Roche	11873580001
Schneider’s Drosophila Medium	Thermo Fisher Scientific	21720024
Sigma protease inhibitor cocktail	Sigma-Aldrich	P8215
SimplyBlue SafeStain (Coomassie G-250 stain)	Thermo Fisher Scientific	LC6065
Sodium Pyruvate	Thermo Fisher Scientific	11360070
SPY650-DNA probe	Spirochrome	SC501
STLC	Sigma-Aldrich	164739
StrataClone Blunt PCR cloning kit	Agilent Technologies	240207
T4 DNA ligase	New England Biolabs	M202
T4 polynucleotide kinase	New England Biolabs	M201
TEV protease	Axel Knebel, MRC PPU Reagents & Services	DU6811
Thymidine	Sigma-Aldrich	T9250
Tissue culture–grade polyvinylpyrrolidone	Sigma-Aldrich	P2307
3–8% Tris-Acetate NuPAGE	Life Technologies	WG1602BOX
Trypsin-EDTA	Thermo Fisher Scientific	25300054
Tween-20	Sigma-aldrich	P1379
*C. elegans* CMG helicase (including MCM-2 isoform a, MCM-4 isoform a, MCM-6 isoform a and SLD-5 isoform a; other components only have one isoform)	(Xia et al, 2021)	N/A
*C. elegans* GINS	(Xia et al, 2023)	N/A
*C. elegans* CLSP-1	(Xia et al, 2021)	N/A
*C. elegans* CTF-4	(Xia et al, 2021)	N/A
*C. elegans* CTF-18_RFC	(Xia et al, 2021)	N/A
*C. elegans* DNSN-1	(Xia et al, 2023)	N/A
*C. elegans* DNSN-1 DN	(Xia et al, 2023)	N/A
*C. elegans* DNSN-1 3 A	This study	N/A
*C. elegans* DNSN-1 8E	This study	N/A
*C. elegans* DNSN-1 8 A	This study	N/A
*C. elegans* MCM-10	This study	N/A
*C. elegans* MUS-101	This study	N/A
*C. elegans* RPA	This study	N/A
*C. elegans* SLD-2	This study	N/A
*C. elegans* TIM-1_TIPN-1	(Xia et al, 2021)	N/A
AcTEV	Thermo Fisher Scientific	12575015
AEBSF	Sigma-Aldrich	A8456
AGB1	Tocris	7686
Basal Medium Eagle vitamins	Thermo Fisher Scientific	21010046
β-mercaptoethanol	Sigma-Aldrich	M6520
4–12% Bis-Tris NuPAGE	Life Technologies	WG1402BOX
Blasticidin	Invitrogen	ant-bl-10p
Calmodulin sepharose 4B	GE Healthcare	17052901
CB-5083	Selleckchem	S8101
Cell culture plates, 96-well, CELLSTAR with µClear bottom	Greiner	655090
Chemically defined lipid concentrate	Thermo Fisher Scientific	11905031
Chromatography paper	GE Healthcare	3030861
Chromotek GFP-Trap Magnetic Particles M-270	Thermo Fisher Scientific	17353353
Clear-bottomed black wall 96-well plates	Greiner	655090
Click-iT EdU Imaging Kit	Thermo Fisher Scientific Inc.	B10184
Concentrated penicillin-streptomycin	Thermo Fisher Scientific	11568876
Cytivia MicroSpin G-50 columns	Thermo Fisher Scientific	27533001
Crystal violet	Sigma-Aldrich	HT90132
DAPI	Thermo Fisher Scientific	62248
DMEM	Thermo Fisher Scientific	11960044
dTAG13	MRC PPU Reagents and Services	
Dynabeads M-270 Epoxy	Thermo Fisher Scientific	14302D
5-ethynyl-2’-deoxyuridine (EdU)	Thermo Fisher Scientific Inc.	E10187
FBS	LabTech	FCS-SA/500
Bovine FCS	Thermo Fisher Scientific	A5256701
Formaldehyde	Thermo Scientific	11586711
Formaldehyde 4% stabilised, buffered	VWR	9713.1000
Formaldehyde 16% (w/v), Methanol free	Thermo Fisher Scientific	28908
Gelatin	Sigma-Aldrich	G1890
Glass beads	Stratech	11079105
L-Glutamine	Thermo Fisher Scientific	25030081
Hoescht 33342	Sigma-Aldrich	B2261
Hydroxyurea	Molekula	10872383
Hygromycin	Invitrogen	ant-hg-5
iBlot membrane	Thermo Fisher Scientific	IB301031
IgG sepharose 6 Fast Flow	GE Healthcare	17096901
Inulin	Sigma-Aldrich	I2255
Knockout serum replacement	Thermo Fisher Scientific	10828028
LIF	MRC PPU Reagents and Services	DU1715
Neomycin (G418)	Formedium	G418S
Ni-NTA agarose	Qiagen	30210
Non-essential amino acids	Thermo Fisher Scientific	11140050
Novex Hi-Density TBE sample Buffer	Thermo Fisher Scientific	LC6678
4–20% Novex TBE gels	Thermo Fisher Scientific	EC62252BOX
4x NuPAGE LDS sample buffer	Thermo Fisher Scientific	NP0007
PEI	Polysciences, Inc	24765-2
Penicillin-Streptomycin	Thermo Fisher Scientific	15140122
Pepstatin A	Sigma-Aldrich	P5318
Pierce Universal Nuclease	Thermo Fisher Scientific	88702
PreScission protease	MRC PPU Reagents & Services	DU34905
PrimeStar Hot Start DNA polymerase	Takara Bio	R010A
Propidium iodide	Sigma-Aldrich	P4170
Protease Inhibitor Cocktail	Sigma-Aldrich	P8215
Puromycin	Thermo Fisher Scientific	A1113802
RNase A	Thermo Fisher Scientific	EN0531
Roche cOmplete EDTA-free protease inhibitor cocktail	Roche	11873580001
Schneider’s *Drosophila* Medium	Thermo Fisher Scientific	21720024
Sigma protease inhibitor cocktail	Sigma-Aldrich	P8215
SimplyBlue SafeStain (Coomassie G-250 stain)	Thermo Fisher Scientific	LC6065
Sodium pyruvate	Thermo Fisher Scientific	11360070
SPY650-DNA probe	Spirochrome	SC501
STLC	Sigma-Aldrich	164739
StrataClone Blunt PCR cloning kit	Agilent Technologies	240207
T4 DNA ligase	New England Biolabs	M202
T4 polynucleotide kinase	New England Biolabs	M201
TEV protease	Axel Knebel, MRC PPU Reagents & Services	DU6811
Thymidine	Sigma-Aldrich	T9250
Tissue culture–grade polyvinylpyrrolidone	Sigma-Aldrich	P2307
3–8% Tris-Acetate NuPAGE	Life Technologies	WG1602BOX
Trypsin-EDTA	ThermoFisher Scientific	25300054
Tween-20	Sigma-aldrich	P1379
**Software**		
FlowJo	BD Biosciences	https://www.flowjo.com/
ImageJ	National Institute of Health	https://imagej.net/ij/
Prism 9	GraphPad	https://www.graphpad.com/scientific-software/prism/
Tableau	Tableau	https://www.tableau.com
**Other**		
ScanR High-Content Screening Microscopy (Olympus).	Thermo Fisher Scientific Inc.	N/A

Resources and reagents from this study are listed in the Reagents and tools table and are available from MRC PPU Reagents and Services (https://mrcppureagents.dundee.ac.uk), or upon request.

### Maintenance of *C. elegans* strains

All *C. elegans* strains in this study are derived from the wild-type strain known as ‘Bristol N2’ and are described in the Reagents and tools table. Alleles generated by genome editing via CRISPR-Cas9 (Suny Biotech) were outcrossed eight times with the N2 wild type before use.

Worms were maintained according to standard procedures (Brenner, 1974) and grown on ‘Nematode Growth Medium’ (NGM: 3 g/l NaCl; 2.5 g/l peptone; 20 g/l agar; 5 mg/l cholesterol; 1 mM CaCl_2_; 1 mM MgSO_4_; 2.7 g/l KH_2_PO_4_; 0.89 g/l K_2_HPO_4_).

### Expression of recombinant C. elegans proteins

To express recombinant worm proteins in budding yeast (see the Reagents and tools table for details), the *Saccharomyces cerevisiae* strain yJF1 (*MAT***a** *ade2-1 ura3-1 his3-11,15 trp1-1 leu2-3,112 can1-100 bar1∆::hphNT pep4∆::kanMX*) was transformed with the indicated linearised plasmids, containing fragments generated by DNA synthesis (Genscript). Codon usage of the coding sequences for worm proteins was optimised for high-level expression in *Saccharomyces cerevisiae*, as described previously (Yeeles et al, 2015).

To express worm proteins in bacteria, plasmids listed in the Reagents and Tools table were transformed into the *E. coli* strain Rosetta™ (DE3) pLysS (70956, Novagen).

### Depletion of *C. elegans* proteins by RNA interference

Worms were fed with the RNAse III-deficient bacterial strain HT115 that had been transformed with plasmids expressing double-stranded RNA (plasmids based on the ‘L4440’ parental plasmid - see the Reagents and tools table). Feeding was performed on 6 cm plates that contained the following medium: 3 g/l NaCl, 20 g/l agarose, 5 mg/l cholesterol, 1 mM CaCl_2_, 1 mM MgSO_4_, 2.7 g/l KH_2_PO_4_, 0.89 g/l K_2_HPO_4_, 1 mM IPTG and 100 mg/l Ampicillin.

Plasmids expressing dsRNA were made by cloning PCR products into the vector L4440 (Reagents and Tools table), after amplification of 1 kb cDNA products (*rnr-1*) or full-length cDNA (*cdc-45*, *sld-2*), using a cDNA library that was kindly provided by Sarah-Lena Offenburger and Giulia Saredi. An equivalent plasmid for depletion of SLD-2-GFP was made by amplification of the *GFP-*tag sequence from KAL213. To deplete all four subunits of GINS simultaneously, we cloned contiguous fragments representing the four GINS subunits into a single L4440 plasmid. Details of sequences used in the RNAi vectors are provided in the Reagents and tools table. Bacteria expressing *perm-1* dsRNA were obtained from a genome-wide RNAi library (Source Bioscience). An empty L4440 vector was used as the control for the RNAi experiments.

Bacterial cultures were grown to an optical density (OD600) of 1, supplemented with 3 mM IPTG to induce dsRNA expression, and then 400 µl of culture was added to each 6 ml plate, before incubation overnight at room temperature. The next day, 10–20 worms were added to each plate and allowed to feed for 48 h at 20 °C.

### Analysis of *C. elegans* embryos by spinning disk confocal microscopy

Worms at the larval L4 stage were incubated for 48 h at 20 °C on 6 cm plates that had been prepared as above for RNAi feeding. Subsequently, adult worms were dissected in M9 medium (6 g/L Na_2_HPO_4_, 3 g/L KH_2_PO_4_, 5 g/L NaCl, 0.25 g/L MgSO_4_), before five embryos were transferred to a 2% agarose pad and recorded simultaneously from the one-cell stage to the four-cell stage. To monitor events during the early S phase of the first embryonic cell cycle, we selected embryos that exhibited mild cortical contractions, which are characteristic of anaphase of the second meiotic division, and then transferred single embryos to a 1% agarose pad prior to imaging. This procedure was repeated to obtain a total of five embryos for each experimental condition.

Time-lapse video microscopy images were recorded at 24 °C with images taken every 10 s as described previously (Sonneville et al, 2017). We used a Zeiss Cell Observer SD microscope with a Yokogawa CSU-X1 spinning disk and HAMAMATSU C13440 camera, fitted with a PECON incubator and a 60X/1.40 Plan Apochromat oil immersion lens (Olympus). For each time point, a single optical section (z-layer) was captured using ZEN blue software (Zeiss). Subsequently, images were processed and analyzed with ImageJ software (National Institute of Health). The raw images were cropped and the intensity scale was adjusted identically for all conditions. The ‘bit depth’ was then changed from 16 bits to 8 bits, and videos were assembled. To generate time-lapse sequences for the figures, images were selected at the indicated times, unless the chromosomes were out of focus at any given time point, in which case the previous or subsequent image was utilised (±10 s). The precise details of the selected timepoints for each experiment are given in the legends to Movies EV1–EV45. Images were then cropped around the chromatin and assembled into a contiguous sequence.

For the experiment in Appendix Fig. S2, the fluctuation of pixel intensity within the female pronucleus was determined as follows, by calculating the coefficient of variation. For five embryos at the 1-min time point, the image intensity scale was adjusted identically. The female pronucleus was selected as the region of interest, and the mean pixel intensity and standard deviation were measured using ImageJ. The coefficient of variation was then determined as the standard deviation divided by the mean. The resulting values were imported into Prism software (GraphPad) for statistical analysis.

In Appendix Fig. S3B,C, the association of mCherry-RPA32 with metaphase chromatin was quantified in five embryos by selecting the timepoint with the maximum in-focus signal. The ‘integrated fluorescence intensity’ was calculated within a region encompassing the chromatin, as the sum of the fluorescence intensity values of all pixels within the selected region. Background fluorescence was then subtracted, using the integrated density of a region of identical size that did not contain chromatin. Data were analysed using Prism software (GraphPad).

### Monitoring the viability of *C. elegans* embryos

For the experiment in Fig. EV1C, experiments were performed in triplicate, using RNAi feeding as above. Subsequently, five adult worms were allowed to produce embryos on a plate during a period of 150 min, before the adults were removed and the embryos counted (typically between 60 and 100 embryos). Three days afterwards, the percentage of embryos that had developed into viable adults was determined, together with the mean and standard deviation for each triplicate set.

### EdU incorporation to monitor DNA synthesis in *C. elegans* embryos

To permeabilize the eggshell to EdU, a bacterial culture expressing *perm-1* dsRNA was diluted 1 in 5 with control bacteria (transformed with the empty L4440 vector), or bacteria expressing GFP dsRNA (for co-depletion of SLD-2-GFP). Otherwise, feeding plates were prepared as described above.

Isotonic growth medium for blastomere culture (Shelton and Bowerman, 1996) was prepared in two steps as follows. Firstly, 1 ml of 5 mg/ml inulin (Sigma-Aldrich, I2255), 50 mg tissue culture–grade polyvinylpyrrolidone (Sigma-Aldrich, P2307), 100 µl Basal Medium Eagle vitamins (Thermo Fisher Scientific, 21010046), 100 µl chemically defined lipid concentrate (Thermo Fisher Scientific, 11905031), 100 µl of 100X concentrated penicillin-streptomycin (Thermo Fisher Scientific, 11568876) and 9 ml Schneider’s *Drosophila* Medium (Thermo Fisher Scientific, 21720024) were mixed and stored at 4 °C. Secondly, 650 µl of this mixture was added to 350 µl of bovine FCS (Thermo Fisher Scientific, A5256701) that had been heat-treated for 30 min at 56 °C, giving a final concentration of 35% FCS.

Permeabilised embryos were then dissected from ten worms in 150 µl of isotonic growth medium for blastomere culture. After dissection, the mixture of embryos was supplemented with an additional 150 µl of isotonic growth medium containing 40 mM EdU (Thermo Fisher Scientific, E10187), making a final concentration of 20 mM EdU. The embryos were then incubated for 10 min, during which time we identified embryos entering the first S phase, on the basis that embryos in early-mid S-phase have strong cortical contractions but have not yet undergone pseudo cleavage. Subsequently, around eight embryos in early-mid S-phase were transferred in 2–3 µl to 1 ml fixation solution comprising 4% formaldehyde (Thermo Scientific, 11586711) in phosphate-buffered saline (PBS) and left at 4 °C for 3 days.

Finally, embryos were washed twice with PBS, and EdU was detected using the Alexa-647 click-iT EdU Imaging Kit (ThermoFisher Scientific, C10640). After detection, embryos were washed for 5 min in PBS containing 0.05% Tween-20 (Sigma-Aldrich, P1379) and 5 µg/ml Hoechst 33342 (supplied with EdU Imaging Kit), and then washed twice for 5 min in PBS. Embryos were then mounted on 2% agarose pads and imaged immediately using the Zeiss Cell Observer SD microscope described above. A z-stack of images was acquired from the top to the bottom of each embryo, spanning a depth of ~20 µm in total, with a step size of 0.5 µm. The images shown in Fig. 1 represent a maximum-intensity projection of each z-stack.

An ImageJ macro was developed to automate the estimation of EdU intensity. For each z-stack of images corresponding to the female pronucleus (about 15 images per embryo), the EdU signal intensity was calculated as the sum of the Raw Integrated Density within the area encompassing the female pronucleus, after subtraction of background measured from an adjacent cytoplasmic region of identical size.

The experiment was repeated three times to determine mean values and standard deviation. Due to occasional embryo loss during processing, a total of 19–24 embryos were quantified for each condition.

### Purification of *C. elegans* proteins

Proteins purified in this study are listed in the Reagents and Tools table. TEV protease (DU6811, MRC PPU Reagents and Services) and PreScission (DU34905, MRC PPU Reagents and Services) protease were kindly provided by Dr. Axel Knebel. Other proteins were produced as described in the following sections, using the following buffer: Buffer A: 25 mM Hepes-KOH, pH 7.6, 10% glycerol, 0.02% IGEPAL CA-630, 1 mM TCEP.

The following cocktails of protease inhibitors were used as indicated in the sections below:

1. 1X cocktail corresponding to one ‘Roche EDTA-free protease inhibitor tablet’ (11873580001, Roche) per 25 ml of buffer (one tablet dissolved in 1 ml water makes a 25X stock solution), together with 0.5 mM PMSF, 5 mM benzamidine HCl, 1 mM AEBSF (A8456, Sigma-Aldrich) and 1 mg/ml Pepstatin A (P5318, Sigma-Aldrich).
2. 1X cocktail corresponding to one Roche EDTA-free protease inhibitor tablet (11873580001, Roche) per 25 ml of buffer (one tablet dissolved in 1 ml water makes a 25X stock solution), plus 0.5 mM PMSF.

### Expression of *C. elegans* proteins in budding yeast

The *Saccharomyces cerevisiae* strains used in this study are shown in the Reagents and Tools table. Yeast cells were grown at 25 °C in YP medium (1% Yeast Extract, 21275, Becton Dickinson; 2% bacteriological peptone, LP0037B, Oxoid) supplemented with 2% Raffinose. To express SLD-2 and MUS-101, a 12-litre exponential culture was grown to 1–2 × 10^7^ cells/ml and then synchronised in G1-phase by adding α factor to 0.6 µg/ml for 3.5 h at 25 °C. The G1 culture was induced for 6 h at 20 °C by the addition of Galactose to a final concentration of 2%. To express MCM-10 and RPA, a 12-litre exponential culture was grown to 2–3 × 10^7^ cells/ml and then directly induced for 6 h at 20 °C by the addition of Galactose to a final concentration of 2%. Cells were collected by centrifugation and washed once with lysis buffer (indicated below for each purification) lacking protease inhibitors. Cell pellets (~30 g) were then resuspended in 0.3 volumes of the indicated lysis buffer containing protease inhibitors. The resulting suspensions were then frozen dropwise in liquid nitrogen and stored at −80 °C. Subsequently, the entire sample of frozen yeast cells were ground in the presence of liquid nitrogen, using a SPEX CertiPrep 6850 Freezer/Mill with three cycles of 2 min at a rate of 15. The resulting powders were then stored at −80 °C.

The CMG helicase was purified as previously described (Xia et al, 2023). TIM-1_TIPN-1, CTF-4, CLSP-1,and CTF-18_RFC were purified as previously described (Xia et al, 2021).

#### C.e. MUS-101

Yeast cell powder was thawed in buffer A/0.5 M NaCl/1X protease inhibitor cocktail 1. The mixture was centrifuged at 100,000×*g* at 4 °C for 0.5 h, followed by another step of centrifugation at 235,000×*g* at 4 °C for 1 h. After spinning, the soluble extract was recovered and mixed with 3 ml IgG resin (17096901, GE). The mixture was incubated at 4 °C for 2 h with rotation.

Resin was collected and washed extensively with buffer A/0.5 M NaCl/1X protease inhibitor cocktail 1. The resin was then incubated with 40 ml buffer A/0.5 M NaCl/10 mM Mg(OAc)_2_/2 mM ATP at 4 °C for 10 min to remove chaperones, then washed extensively with buffer A/0.5 M NaCl. The purified proteins were then eluted by overnight incubation with rotation in 2 ml buffer A/0.5 M NaCl containing 100 µg TEV protease.

The supernatant was collected, and the resin was further eluted twice with 4 ml buffer A/0.5 M NaCl. The elute was concentrated and loaded onto a 24 ml Superdex 200 column in buffer A/0.5 M NaCl. Peak fractions containing MUS-101 were pooled, aliquoted, and snap frozen in liquid nitrogen and stored at −80 °C.

#### C.e. SLD-2

Yeast cell powder was thawed in buffer A/0.5 M NaCl/1X protease inhibitor cocktail 1. The mixture was centrifuged at 100,000×*g* at 4 °C for 0.5 h, followed by another step of centrifugation at 235,000×*g* at 4 °C for 1 h. After spinning, the soluble extract was recovered and mixed with 3 ml IgG resin (17096901, GE). The mixture was incubated at 4 °C for 2 h with rotation.

Resin was collected and washed extensively with buffer A/0.5 M NaCl/1X protease inhibitor cocktail 1. The resin was then incubated with 40 ml buffer A/0.5 M NaCl/10 mM Mg(OAc)_2_/2 mM ATP at 4 °C for 10 min to remove chaperones, then washed extensively with buffer A/0.5 M NaCl. The purified proteins were then eluted by 20 h incubation with rotation in 2 ml buffer A/0.5 M NaCl containing 100 µg TEV protease.

The supernatant was collected, and the resin was further eluted twice with 2 ml buffer A/0.5 M NaCl. The elute was concentrated and loaded onto a 24 ml Superdex 200 column in buffer A/0.5 M NaCl. Peak fractions containing SLD-2 were pooled, concentrated and re-loaded onto a 24 ml Superdex 200 column in buffer A/0.5 M NaCl. Peak fractions were pooled, aliquoted, and snap frozen in liquid nitrogen and stored at −80 °C.

#### MCM-10

Yeast cell powder was thawed in buffer A/0.2 M NaCl/1X protease inhibitor cocktail 1. The mixture was centrifuged at 100,000×*g* at 4 °C for 0.5 h, followed by another step of centrifugation at 235,000×*g* at 4 °C for 1 h. After spinning, the soluble extract was recovered and mixed with 3 ml IgG resin (17096901, GE). The mixture was incubated at 4 °C for 2 h with rotation.

Resin was collected and washed extensively with buffer A/0.2 M NaCl/1X protease inhibitor cocktail 1. The resin was then incubated with 40 ml buffer A/0.2 M NaCl/10 mM Mg(OAc)_2_/2 mM ATP at 4 °C for 10 min to remove chaperones, then washed extensively with buffer A/0.2 M NaCl. The purified proteins were then eluted by overnight incubation with rotation in 2 ml buffer A/0.2 M NaCl containing 100 µg TEV protease.

The supernatant was collected, and the resin was further eluted up to 10 ml with buffer A/0.2 M NaCl. The eluate was loaded onto a 1 ml HiTrap SP HP column in buffer A/0.2 M NaCl. MCM-10 was eluted with a 20 ml gradient from 0.2 to 0.6 M NaCl. The peak fractions were pooled, concentrated, aliquoted, snap frozen in liquid nitrogen and stored at −80 °C.

#### C.e. RPA

Yeast cell powder was thawed in buffer A/0.5 M NaCl/1X protease inhibitor cocktail 1. The mixture was centrifuged at 100,000×*g* at 4 °C for 0.5 h, followed by another step of centrifugation at 235,000×*g* at 4 °C for 1 h. After spinning, the soluble extract was recovered and mixed with 3 ml IgG resin (17096901, GE). The mixture was incubated at 4 °C for 2 h with rotation.

Resin was collected and washed extensively with buffer A/0.5 M NaCl/1X protease inhibitor cocktail 1. The resin was then incubated with 40 ml buffer A/0.5 M NaCl/10 mM Mg(OAc)_2_/2 mM ATP at 4 °C for 10 min to remove chaperones, then washed extensively with buffer A/0.5 M NaCl. The purified proteins were then eluted by overnight incubation with rotation in 2 ml buffer A/0.5 M NaCl containing 100 µg TEV protease.

The supernatant was collected, and the resin was further eluted twice with 4 ml buffer A/0.5 M NaCl. The elute was concentrated and loaded onto a 24 ml Superdex 200 column in buffer A/0.5 M NaCl. Peak fractions containing RPA were pooled, aliquoted, and snap frozen in liquid nitrogen and stored at −80 °C.

### Protein expression in *E. coli*

*C. elegans* DNSN-1 and the truncated or mutated variants DNSN-1-∆N and DNSN-1-3A were purified as previously described (Xia et al, 2023).

### Preparation of DNA substrates for helicase and DNA binding assays

For the DNA helicase assays in Figs. 4D–H, EV3B and EV4C,D with radiolabelled substrate, model DNA replication forks were assembled in vitro from annealed oligonucleotides. Firstly, 400 nM of PAGE-purified 5’ oligonucleotides (see the Reagents and Tools table for oligonucleotide sequences) were labelled with γ-[32 P]-ATP using 10 units of T4 Polynucleotide Kinase (T4 PNK, New England Biolabs M0201S), in a 10 µl volume for 30 min at 37 °C. The reactions were then heated at 95 °C for 10 min to inactive T4 PNK. The 10 µl labelling reaction was then mixed with 200 nM 3’ oligonucleotides (see the Reagents and tools table for oligonucleotide sequences), 100 mM NaCl, and 10 mM Mg(OAc)_2_ in a total volume of 20 µl, before heating to 95 °C for 10 min in a metal heating block. Finally, the metal block was placed at room temperature and the sample left to cool for 3 h, allowing the oligonucleotides to anneal with each other. Unincorporated γ-[32 P]-ATP was then removed using Cytiva MicroSpin G-50 columns (Thermo Fisher Scientific, 27533001).

For the DNA helicase assays in Appendix Fig. S4, radiolabelled substrates were prepared as described above, using the oligos described in the Reagents and tools table.

For the Cy5 labelled fork DNA substrate used in Figs. 4C and EV4E,F, model DNA replication forks were again assembled in vitro from annealed oligonucleotides. Firstly, 200 nM 3’ oligonucleotides that were labelled with Cy5 at the 5’ end and 200 nM 5’ oligonucleotides (see the Reagents and tools table for oligonucleotide sequences) were assembled in a 20 µl annealing reaction that also contained 100 mM NaCl and 10 mM Mg(OAc)_2_, before heating to 95 °C for 10 min in a metal heating block. Subsequently, the metal block was placed at room temperature and the sample left to cool for 3 h, allowing the oligonucleotides to anneal with each other.

To prepare a double-stranded DNA substrate for the DNA binding assay in Fig. EV2, 500 nM of Cy5-labelled 80 nt 5’ oligonucleotides was incubated with 500 nM of 80 nt 3’ oligonucleotides (see the Reagents and Tools table for oligonucleotide sequences), in a total volume of 20 µl containing 100 mM NaCl and 10 mM Mg(OAc)_2_. The reactions were then heated to 95 °C for 10 min before cooling to room temperature, as described above.

### CMG helicase assays

CMG helicase assays with radiolabelled substrate were conducted in a ‘helicase buffer’ consisting of 25 mM HEPES-KOH (pH 7.6), 60 mM potassium glutamate, 0.02% NP-40-S, 1 mM DTT, 10 mM Mg(OAc)2, 0.1 mM EDTA and 0.2 mg/ml bovine serum albumin. Unless indicated otherwise in the figure legends, the DNA, protein and nucleotide concentrations in the final reaction were: 1 nM fork DNA, 30 nM CMG, 120 nM MCM-10, DNSN-1, or SLD-2, 5 mM ATP, plus 40 nM unlabelled 5’ oligonucleotides as ‘trap DNA’ to prevent re-annealing of the product (the experiments in Appendix Fig. S4 used 0.5 nM substrate and 20 nM unlabelled ‘trap DNA’ described in the Reagents and tools table). Reactions were set up as follows: recombinant *C. elegans* CMG (30 nM) was pre-incubated with the DNA substrate (1 nM) at 25 °C for 15 min in helicase buffer supplemented with 0.1 mM AMP-PNP. Reactions were then initiated by the addition of a 10X solution containing ATP, trap DNA and MCM10, DNSN-1 or SLD-2, before incubation at 25 °C for 30 min. The reactions were then stopped by the addition of 3 µl of buffer containing 250 mM EDTA, 5% SDS and 0.6 Units of proteinase K, and the incubation continued at 37 °C for 1 h. The samples were supplemented with Novex Hi-Density TBE Sample Buffer (Thermo Fisher Scientific, LC6678) and analyzed in 4–20% Novex TBE Gels (Thermo Fisher Scientific, EC62252BOX) at 200 V for 30 min in 0.5X TBE with 0.1% SDS. Gels were mounted onto chromatography paper (GE Healthcare, 3030861) and then exposed to BAS-MS Imaging Plates (Fujifilm), which were developed on a Typhoon phosphorimager (GE Healthcare).

Analogous assays with Cy5-labelled substrate were performed as above, except that reactions contained 2 nM fork DNA. At the end of the reaction, 1 mM poly-dT (Reagents and tools table) was added to prevent rebinding of CMG to the ssDNA product, and incubation was continued for 10 min at 25 °C. The reactions were then stopped and analysed as above, before detection of Cy5 with an Amersham ImageQuant 800 imaging system (Cytiva).

### Loading assay for CMG helicase on fork DNA

For the experiment in Fig. EV4E,F, reactions (typically 20 µl in volume) were assembled containing 25 mM Hepes-KOH (pH 7.6), 0.02% IGEPAL CA-630, 0.1 mg/ml BSA, 1 mM DTT, 200 mM KOAc, 10 mM Mg(OAc)_2_, 0.5 mM AMP-PNP, 50 nM Cy5 labelled fork DNA substrate, 30 nM CMG and 120 nM MCM-10/DNSN-1/SLD-2. Samples were then incubated at 4 °C with 5 µl magnetic beads (Dynabeads M-270 Epoxy; 14302D, Thermo Fisher Scientific) that had been coupled to anti-SLD-5 antibodies as described below. After 1 h, protein complexes bound to the magnetic beads were washed twice with 1 ml of buffer containing 25 mM Hepes-KOH (pH 7.6), 0.02% IGEPAL CA-630, 1 mM DTT, 10 mM Mg(OAc)_2_ and 200 mM KOAc. The bound DNA was eluted at 37 °C for 1 h in 20 µl of 1X Laemmli buffer. The samples were then supplemented with Novex Hi-Density TBE Sample Buffer (Thermo Fisher Scientific LC6678) and analyzed in 4–20% Novex TBE Gels (Thermo Fisher Scientific EC62252BOX) at 200 V for 30 min in 0.5X TBE with 0.1% SDS. Cy5 signals were detected with an Amersham ImageQuant 800 imaging system (Cytiva).

### DNA binding assay

DNA binding reactions were set up as follows: 15 nM, 45 nM, or 135 nM of indicated protein was pre-incubated with 5 nM Cy5-labelled DNA at 25 °C for 15 min, in ‘DNA binding buffer’ consisting of 25 mM HEPES-KOH (pH 7.6), 50 mM potassium glutamate, 0.02% NP-40-S, 1 mM DTT, 10 mM Mg(OAc)_2_ and 10% glycerol. The samples were analyzed in 4–20% Novex TBE Gels (Thermo Fisher Scientific, EC62252BOX) at 200 V for 40 min in 0.5X TBE. The gels were then analyzed with a Bio-Rad ChemiDoc.

### Immunoprecipitation of reconstituted complexes of *C. elegans* proteins

Reactions with a final volume of 10 µl were set up on ice as follows: 25 mM Hepes-KOH (pH 7.6), 0.02% IGEPAL CA-630, 0.1 mg / ml BSA, 1 mM DTT, 100 mM KOAc, 10 mM Mg(OAc)_2_, 0.5 mM AMP-PNP, 50 nM DNA substrate comprising 46 bp double-stranded DNA and a 39 nt ‘3’-flap’ of single-stranded DNA (sequences are shown in the Reagents and tools table) and 3.3 µl ‘protein mix’ containing 15 nM CMG and either MCM-10 (30 nM), SLD-2 (30 nM), or DNSN-1 (30 nM dimer), in 300 mM KOAc (giving a final concentration of 200 mM KOAc). Reactions were incubated on ice for 30 min, before addition of 5 µl magnetic beads (Dynabeads M-270 Epoxy; 14302D, Thermo Fisher Scientific) that had been coupled to anti-MCM-6 antibodies as described below, in order to bind the CMG helicase complex. After 1 h, protein complexes bound to the magnetic beads were washed twice with 1 ml of buffer containing 25 mM Hepes-KOH (pH 7.6), 0.02% IGEPAL CA-630, 0.1 mg/ml BSA, 1 mM DTT, 10 mM Mg(OAc)_2_ and 200 mM KOAc. The bound proteins were eluted at 95 °C for 5 min in 30 µl of 1X Laemmli buffer.

### Preparation of antibody-coated magnetic beads

A slurry of activated magnetic beads (Dynabeads M-270 Epoxy; 14302D, Thermo Fisher Scientific) was prepared by resuspending 300 mg of beads in 10 ml of dimethyl formamide. Each coupling reaction involved 425 µl of a slurry of activated magnetic beads, which corresponded to ~1.4 × 10^9^ beads. After removing the supernatant, the beads were washed twice with 1 ml of 0.1 M NaPO_3_ pH 7.4. Subsequently, the beads were incubated with 300 mg of rabbit IgG (S1265, Sigma-Aldrich), *C. elegans* MCM-6 antibody (SA417, MRC PPU Reagents and Services), or *C. elegans* SLD-5 antibody (SA419, MRC PPU Reagents and Services), 300 µl of 3 M (NH4)_2_SO_4_, plus 0.1 M NaPO_3_, pH 7.4, up to a total volume of 900 µl. The mixture was then incubated at 4 °C for 2 days with rotation. Subsequently, the supernatant was removed, and the beads were washed four times with 1 ml of PBS. The beads were then incubated for 10 min in 1 ml of PBS/0.5% IGEPAL CA-630 with rotation at room temperature, before washing twice with 1 ml of PBS. Finally, the washed beads were resuspended with 900 µl of PBS containing 5 mg/ml BSA.

### Culture of mouse ES cells

Mouse ES cells of the E14tg2A background were maintained at 37 °C in a humidified atmosphere of 5% CO_2_, 95% air in Dulbecco’s Modified Eagle Medium (DMEM, Thermo Fisher Scientific, 11960044) supplemented with 10% foetal bovine serum (FBS, FCS-SA/500, LabTech), 5% KnockOut Serum Replacement (Thermo Fisher Scientific, 10828028), 2 mM L-glutamine (Thermo Fisher Scientific, 25030081), 100 U/ml Penicillin-Streptomycin (Thermo Fisher Scientific, 15140122), 1 mM sodium pyruvate (Thermo Fisher Scientific, 11360070), 1X non-essential amino acids (Thermo Fisher Scientific, 11140050), 0.05 mM β-mercaptoethanol (Sigma-Aldrich, M6250) and 0.1 μg/ml LIF (MRC PPU Reagents and Services, DU1715). Around 2  × 10^6^ cells were plated on 10 cm plates precoated with 0.1% gelatin (Sigma, G1890), and then on alternate days, the cells were released using 0.05% Trypsin-EDTA (Thermo Fisher Scientific, 25300054), before 1 in 10 dilution in fresh medium and re-plating. Cells were treated as indicated in the figures and legends with hydroxyurea (Molekula, 10872383), 250 nM AGB1 (Tocris, 7686), 250 nM dTAG13 (MRC PPU Reagents and Services) or CB-5083 (Selleckchem, S8101).

### CRISPR-Cas9 genome editing, transfection of mouse ES cells and selection of clones

We used the ‘CRISPR Finder’ provided by the Wellcome Sanger Institute (https://wge.stemcell.sanger.ac.uk//find_crisprs) to design pairs of guide RNAs (gRNAs) to target each specific site of interest in the mouse genome. For each gRNA, two oligos containing the homology region to the targeted gene were annealed and cloned into the vectors pX335 or pKN7 after digestion by BbsI, as previously described (Pyzocha et al, 2014).

To introduce tags at the amino termini of RECQL4 or MCM10, cells were transfected with 2 µg each of three plasmids. The first plasmid expressed the Cas9-D10A nickase and a gRNA, the second expressed the Puromycin-resistance gene and a second gRNA. The third was a ‘donor plasmid’ in which the chosen tag was flanked by around 500–1000 bp homology to the targeted gene, together with the Hygromycin or Blasticidin-resistance gene. The plasmids were added to 200 µl of DMEM plus 15 µl of 1 mg/ml linear Polyethylenimine (PEI; Polysciences, Inc, 24765-2). Subsequently, 1.0 × 10^6^ cells were gently mixed with the DNA-PEI-DMEM mix and incubated at room temperature for 30 min. Cells were then transferred to a single well of a six-well plate containing 2 ml of complete DMEM. Cells were subjected to two 24-h rounds of selection with fresh medium containing 2 μg/ml Puromycin (Thermo Fisher Scientific, A1113802) followed by 1 to 2 weeks of selection with fresh medium containing 200–500 µg/ml Hygromycin (Invitrogen, ant-hg-5) or 7.5 μg/ml Blasticidin (Invitrogen, ant-bl-10p) as appropriate, to select for the marker in the donor vector (Hygromycin-resistance for *BromoTag-Mcm10* and Blasticidin-resistance for *dTAG-Recql4*). A series of dilutions from 10 to 10,000 times was made of the surviving cells, in complete DMEM containing an antibiotic corresponding to the second selection marker, before re-plating to allow the picking of single colonies after 5 to 7 days additional incubation. Cells were re-plated once again in selective medium, and then after 6 to 10 days the clones were expanded and subsequently analysed by immunoblotting, PCR and DNA sequencing of the target locus.

To insert the GFP tag into the *Psf1* locus in *BromoTag-Mcm10 dTAG-Recql4* cells, the procedure was similar except that the donor plasmid lacked a selective marker, and cells were initially subjected to two 24-h rounds of Puromycin selection. Subsequently, viable GFP-positive cells were sorted by flow cytometry into individual wells of a 96-well plate containing complete DMEM lacking antibiotics.

To create small deletions in exon1 of *Recql4* or exon 3 of *Mcm10*, or to insert *mCherry* at the start of the *Timeless* coding sequence, cells were transfected with plasmids expressing the Cas9-D10A nickase, two gRNAs and Puromycin resistance. After two 24-h rounds of Puromycin selection, surviving cells were sorted by flow cytometry into individual wells of a 96-well plate containing complete DMEM.

To delete the entire *Mcm10* or *Recql4* gene, as summarised in Appendix Figs. S6 and S7, we adapted a previously described protocol (Saito et al, 2024). Cells were transfected with plasmids expressing wild-type Cas9, two gRNAs targeting the 5’ and 3’ end of *Mcm10* or *Recql4*, and the Puromycin-resistance gene. After two 24-h rounds of Puromycin selection, surviving cells were diluted, and then 50–100 cells were plated onto new 10 cm plates and incubated until colonies formed. Single colonies were then picked and grown in a 24-well plate containing complete DMEM lacking antibiotics.

To express *Recql4* from the *Rosa26* ‘safe haven’ locus, we transfected cells with two plasmids. The first expressed the Cas9-D10A nickase, the Puromycin-resistance gene, and a single gRNA for *Rosa26* as described previously (Villa et al, 2021). The second was a donor plasmid expressing wild-type or mutant RECQL4 from the constitutive EF1a promotor, together with a Neomycin resistance gene. These sequences in the donor vector were flanked by around 800 bp 5’ and 3’ homology to the *Rosa26* locus. After two rounds of 24-h selection with puromycin, integration of the donor DNA was selected by exposing cells for one to two weeks to fresh medium containing 700 μg/ml G418 (Formedium, G418S). Single clones were then selected and analysed as described above for *BromoTag-Mcm10* and *dTAG-Recql4*.

### PCR and sequence analysis for genotyping of mouse ES cells

Cells from a 10 cm plate were released as described above. One third of the cells were pelleted and resuspended in 100 μl of 50 mM NaOH, and the sample was heated at 95 °C for 15 min. The pH was then neutralised by the addition of 11 µl of 1 M Tris-HCl (pH 6.9). Subsequently, a PCR reaction was performed with PrimeStar Hot Start DNA polymerase (Takara Bio, R010A) and the product was subcloned with StrataClone Blunt PCR cloning kit (Agilent Technologies, 240207), before the DNA was sequenced with M13 Forward, M13 Reverse primers and primers specific to the target locus (see the Reagents and tools table).

### Cell proliferation assay for mouse ES cells

In Fig. 5A,B, cells were transfected as indicated with pairs of gRNAs to create small deletions at the beginning of exon1 of *Recql4* or exon 3 of Mcm10, as described above (plasmids expressing gRNAs and the Cas9-D10A nickase were omitted in the control). After two rounds of puromycin selection, 500 cells (*Control* and *Mcm10∆*) or 1000 cells (*Recql4∆*) were seeded on a 10 cm dish and incubated at 37 °C for 7 days (*Control* and *Mcm10∆*) or 10 days (Recql4∆). The cells were then washed with phosphate-buffered saline (PBS) and fixed with 20% methanol. Subsequently, cells were stained with 0.5% crystal violet solution (Sigma-Aldrich, HT90132) and the plates were scanned.

In Fig. 5C, 500 cells were seeded on a 10 cm dish. After 24 h, 250 nM AGB1 and 250 nM dTAG13 were added to the medium, before incubation at 37 °C for 7 days. The cells were then washed with PBS and fixed with 20% methanol, before staining and scanning as above.

Generation times were measured in at least three independent experiments, before calculation of the mean and standard deviation.

### Denatured protein extracts of mouse ES cells

Firstly, 1.0 × 10^6^ cells were seeded in a six-well plate. The next day, the cells were treated with 250 nM AGB1 (Tocris, 7686) and/or 250 nM dTAG13 for 3 h, as indicated in Figs. 5E and 7A,F. Cells were then released from the dish with 0.05% Trypsin-EDTA and washed with PBS. The pellet was resuspended in 150 µl of 20% TCA, and then 150 µl of acid-washed glass beads (Stratech, 11079105) was added, before vortexing for one minute. The supernatant was then transferred into a fresh 1.5 ml Eppendorf tube. The glass beads were then washed with 150 µl of 5% TCA, and this second supernatant was added to the first one, before centrifugation for 10 min at 845×*g* at room temperature. The pellet was then resuspended in 37.5 µl ‘High pH Laemmli buffer’ (150 µl of 1 M Tris base per 1 ml of 1X Laemmli buffer) and 12.5 µl 4xNuPAGE LDS sample buffer (Thermo Fisher Scientific, NP0007) before incubation at 95 °C for 5 min. For the experiments in Fig. 8, 250 nM PROTACs and 2 mM Hydroxyurea (HU, Molekula, 10872383) was added to the cells for the times indicated in the figure legends.

### Synchronisation of mouse ES cells with respect to the cell cycle

For the experiment in Fig. 6, 2.5 × 10^6^ cells were seeded on a 10 cm dish for subsequent flow cytometry, or 5 × 10^6^ cells on a 20 cm dish for the generation of native cell extracts for immunoprecipitation analysis. After 24 h, 1.25 mM thymidine (Sigma-Aldrich, T9250) was added to cells for 18 h to synchronise them in early S- phase. The cells were then washed twice with PBS and then incubated for nine hours in fresh medium containing 5 µM *S*-trityl-L-cysteine or STLC (Sigma-Aldrich, 164739), which inhibits the kinesin Eg5 and arrests cells in mitosis. During the last 2 h of the arrest with STLC, 250 nM of PROTACs (both AGB1 and dTAG13) was added to degrade both BromoTag-MCM10 and dTAG-RECQL4. The cells were then washed three times with PBS and released into fresh medium in the presence or absence of 250 nM PROTACs as indicated in Fig. 6.

### Measuring the DNA content of mouse ES cells by flow cytometry

Cells were released from a 10 cm dish and fixed with 1 ml 70% ethanol. Subsequently, 300 μl of fixed cells were washed once with PBS and then resuspended in 1 ml PBS containing 0.05 mg/ml propidium iodide (Sigma-Aldrich, P44170) and 0.05 mg/ml RNase A (Thermo Fisher Scientific, EN0531). The cells were then incubated at 24 °C for 1 h to digest RNA. The samples were processed with an LRS Fortessa flow cytometer (Becton Dickinson) and the data analysed with FlowJo software.

### Immunoprecipitation of protein complexes from extracts of mouse ES cells

For the experiment in Fig. 6C, four 15 cm dishes were used for each sample. Cells were released from the plates by incubation with PBS containing 1 mM EDTA and 1 mM EGTA for 10 min and then harvested by centrifugation. The cells were then resuspended in one volume of lysis buffer: 100 mM Hepes-KOH, pH 7.9, 100 mM potassium acetate, 10 mM magnesium acetate, 2 mM EDTA, 10% glycerol, 0.1% Triton X-100, 2 mM sodium fluoride, 2 mM sodium β-glycerophosphate pentahydrate, 1 mM dithiothreitol, 1% Protease Inhibitor Cocktail (Sigma-Aldrich, P8215), 1x Complete Protease Inhibitor (Roche, 05056489001). Chromosomal DNA was digested for 30 min at 4 °C by the addition of 1600 U/ml of Pierce Universal Nuclease (Thermo Fisher Scientific, 88702), before centrifugation at 20,000×*g* for 30 min at 4 °C. The resulting extract was added to 20 µl ChromoTekGFP-Trap magnetic Particles M-270 (Thermo Fisher Scientific, 17353353) and incubated for 2 h at 4 °C. Bound proteins were eluted by heating the beads at 95 °C for 5 min in the presence of 50 µl Laemmli buffer. Proteins were separated on a 4–12% Bis-Tris NuPAGE gel (Life Technologies, NP0301) in 1X MOPS buffer, and then detected by immunoblotting.

### High-content screening microscopy to monitor CMG assembly in mouse ES cells

Mouse ES cells were seeded at 20,000 cells per well in clear-bottomed black wall 96-well plates (Greiner, 655090). Cells were grown for 24 h, followed by an additional period in the presence or absence, as indicated in the figures, of 250 nM AGB1, 250 nM dTAG13 or 2 mM hydroxyurea. Cells were then treated with 20 μM EdU for 20 min, except for a small number of wells that were left untreated to serve as controls for the detection of EdU incorporation.

Subsequently, cells were washed with cold PBS containing 1 mM CaCl_2_, 1 mM MgCl_2_, and 20 µM thymidine. To remove proteins not bound to chromatin, the cells were pre-extracted for 5 min with cold pre-extraction buffer (20 mM HEPES-KOH pH 7.6, 20 mM NaCl, 5 mM MgCl_2_, 0.5% NP-40, 1 mM DTT) supplemented with protease inhibitors (Roche, Complete EDTA-free, 11873580001). Cells were then fixed for 15 min in 3.5% formaldehyde in PBS, before washing with PBS containing 1 mM CaCl_2_, 1 mM MgCl_2_. Afterwards, EdU was covalently linked to the fluorescent dye Alexa-647 using the click-iT EdU Imaging Kit (Thermo Fisher Scientific, B10184). The cells were then washed in PBS, followed by incubation in methanol for 15 min at 4 °C, before blocking for 30 min with 5% BSA in PBS supplemented with 0.05% Tween-20.

Subsequently, the cells were incubated with antibodies to the chosen epitopes. CDC45 was detected with a 1 in 50 dilution of an anti-CDC45 antibody (Cell Signalling Technology, 11881, rabbit monoclonal) for 66 h at 4 °C. MCM2 was detected with a 1 in 250 dilution of anti-MCM2 antibody (BD Biosciences, 610701, mouse monoclonal) for 3 h at room temperature in the dark. Detection of RPA32 (ABCAM, Ab2175, mouse monoclonal) was performed on cells without EdU or methanol treatment, by incubating cells with a 1 in 200 dilution overnight at 4 °C. Subsequently, cells were washed in 5% BSA/PBST followed by incubation with donkey anti-rabbit IgG coupled to Alexa Fluor 488 (Invitrogen, A-21206) or goat anti-mouse IgG coupled to Alexa Fluor 546 (Invitrogen, A-11030), for 1 h at room temperature in the presence of 2.5 μg/ml 4′,6-diamidino-2-phenylindole or DAPI (Thermo, 62248). To detect GFP-PSF1, cells were prepared as above, but the methanol fixation and antibody incubation steps were omitted.

The stained cells were then washed and stored in PBS at 4 °C, before imaging as previously described (Evrin et al, 2023) with a ScanR inverted microscope high-content screening station (Evident Scientific), equipped with wide-field optics and UPLXAPO20X air objective, together with appropriate filters for DAPI, FITC (Alexa Fluor 488), Cy3 (Alexa Fluor 546) and Cy5 (Alexa-647). For each condition, 25 images were acquired automatically in each of two to three wells, via the ScanR acquisition software (Olympus, 3.2).

Images were then processed and analysed with ScanR analysis software (Olympus, 3.2). Firstly, a dynamic background correction was applied to all images. Then, the DAPI signal was used to identify individual nuclei as ‘main objects’ using the edge-detection module. The identification of nuclei was further refined using a scatter plot of ‘circularity factor’ versus area. The pixel intensities in the various channels were then calculated for each nucleus (mean and total intensities). These values were then exported and analysed with Tableau v2025.3.1 (www.tableau.com), to generate colour-coded scatter plots. Within each experiment, similar cell numbers were processed for the various conditions (at least 5000–25,000 cells in each case). For scatter plots involving CDC45 and MCM2, the EdU-negative cell population was identified by applying a threshold, equal to the mean nuclear Cy5 intensity in wells from the same plate that had not been treated with EdU.

For the quantification of the EdU incorporation in Appendix Fig. S11D, and chromatin-bound RPA32 in Appendix Fig. S11E, the mean fluorescence intensity per nucleus for each well was calculated using ScanR analysis software. Measurements were obtained using the ‘Well Results’ feature with ‘Nuclei’ selected as the reference gate. For the quantification of EdU incorporation in Appendix Figs. S9C,D and S12B, the mean fluorescence intensity per nucleus was firstly calculated as above for each well. The relative EdU intensity was then calculated by dividing the mean fluorescence intensity per nucleus in each well, by the average of the mean fluorescence intensity per nucleus across the control wells.

### Live-cell imaging of GFP-PSF1 on heterochromatin patches in mouse ES cells

For the experiment in Fig. 6D, live mouse ES cells were stained with a 4000-fold dilution of SPY650-DNA (SC501, Spirochrome), before imaging by spinning disk confocal microscopy as described above for *C. elegans*. Over 600 cells were examined for each sample, and the proportion of cells in which GFP-PSF1 co-localised with heterochromatin patches was determined.

For the experiment in Fig. 8E, cells were permeabilised and fixed by incubation for 10 min in cold buffer containing 20 mM PIPES (pH 6.8), 10 mM EGTA, 0.2% Triton X-100, 1 mM MgCl_2_ and 4% (w/v) paraformaldehyde (Life Technologies, 28908). The fixed cells were then washed with PBS supplemented with 3% (w/v) BSA, 1 mM MgCl_2_, 1 mM CaCl_2_ and incubated for 30 min in the same buffer. Subsequently, cells were incubated at 4 °C overnight in a 1:100 dilution of RPA32 antibody (Abcam, ab2175) and 1 μg/ml Hoechst 3342 (Sigma, B2261) in PBS supplemented with 3% (w/v) BSA, 1 mM MgCl_2_ and 1 mM CaCl_2_. After three washes with PBS supplemented with 3% (w/v) BSA, 1 mM MgCl_2_ and 1 mM CaCl_2_, cells were incubated for one hour at room temperature in the dark with a 1:1000 dilution of the secondary antibody ‘AlexaFluor594 donkey anti-mouse’ (Invitrogen, A-21203). Finally, cells were washed three times with PBS containing 1 mM MgCl_2_ and 1 mM CaCl_2_ before imaging by spinning disk confocal microscopy. For each condition, 60–170 were examined in three independent experiments, and the proportion of cells in which RPA32 co-localised with GFP-PSF1 foci was calculated.

For the experiment in Fig. 8G, cells were fixed in 4% paraformaldehyde (VWR, 9713.1000) in PBS supplemented with 1 mM MgCl_2_ and 1 mM CaCl_2_ for 15 min at room temperature. After several washes with PBS containing 1 mM MgCl_2_ and 1 mM CaCl_2_, cells were incubated at 4 °C overnight in PBS supplemented with 3% (w/v) BSA, 1 mM MgCl_2_, 1 mM CaCl_2_ and 1 μg/ml Hoechst 3342 (Sigma, B2261) to stain the nuclear DNA. As above, cells were then washed and imaged. The signal intensity of 20 heterochromatin patches containing GFP-PSF1 was then measured using ImageJ, together with the associated mCherry-TIMELESS signal intensity, in three independent experiments. Subsequently, the ratio of the mCherry-TIMELESS and GFP-PSF1 signal intensities was calculated.

## Supplementary information

Appendix

Peer Review File

Movie EV1

Movie EV2

Movie EV3

Movie EV4

Movie EV5

Movie EV6

Movie EV7

Movie EV8

Movie EV9

Movie EV10

Movie EV11

Movie EV12

Movie EV13

Movie EV14

Movie EV15

Movie EV16

Movie EV17

Movie EV18

Movie EV19

Movie EV20

Movie EV21

Movie EV22

Movie EV23

Movie EV24

Movie EV25

Movie EV26

Movie EV27

Movie EV28

Movie EV29

Movie EV30

Movie EV31

Movie EV32

Movie EV33

Movie EV34

Movie EV35

Movie EV36

Movie EV37

Movie EV38

Movie EV39

Movie EV40

Movie EV41

Movie EV42

Movie EV43

Movie EV44

Movie EV45

Source data Fig. 1

Source data Fig. 2

Source data Fig. 3

Source data Fig. 4

Source data Fig. 5

Source data Fig. 6

Source data Fig. 7

Source data Fig. 8

Source Data for Expanded View and Appendix

Expanded View Figures

## Data Availability

This study includes no data deposited in external repositories. The source data of this paper are collected in the following database record: biostudies:S-SCDT-10_1038-S44318-026-00915-9.
