# Supplementary material for: MCM10 and SLD-2/RECQL4 jointly activate the CMG helicase during metazoan DNA replication initiation
Source: EMBO J. 2026 Sep 28;45(21):7582–620. doi: 10.1038/s44318-026-00915-9 (PMC13728148; doi:10.1038/s44318-026-00915-9)
Supplement: Supplementary file 1 [file 44318_2026_915_MOESM1_ESM.pdf]

Appendix - Table of Contents

MCM10 and SLD-2/RECQL4 jointly activate the CMG helicase during metazoan DNA replication initiation

Remi Sonneville, Cecile Evrin, Jane Wright, He Huang, Yisui Xia and Karim P.M. Labib

Appendix Figure S1	Page 2
Appendix Figure S2	Page 3
Appendix Figure S3	Page 4
Appendix Figure S4	Page 5
Appendix Figure S5	Page 6
Appendix Figure S6	Page 7
Appendix Figure S7	Page 8
Appendix Figure S8	Page 9
Appendix Figure S9	Page 10
Appendix Figure S10	Page 11
Appendix Figure S11	Page 12
Appendix Figure S12	Page 13

Appendix Figure S1

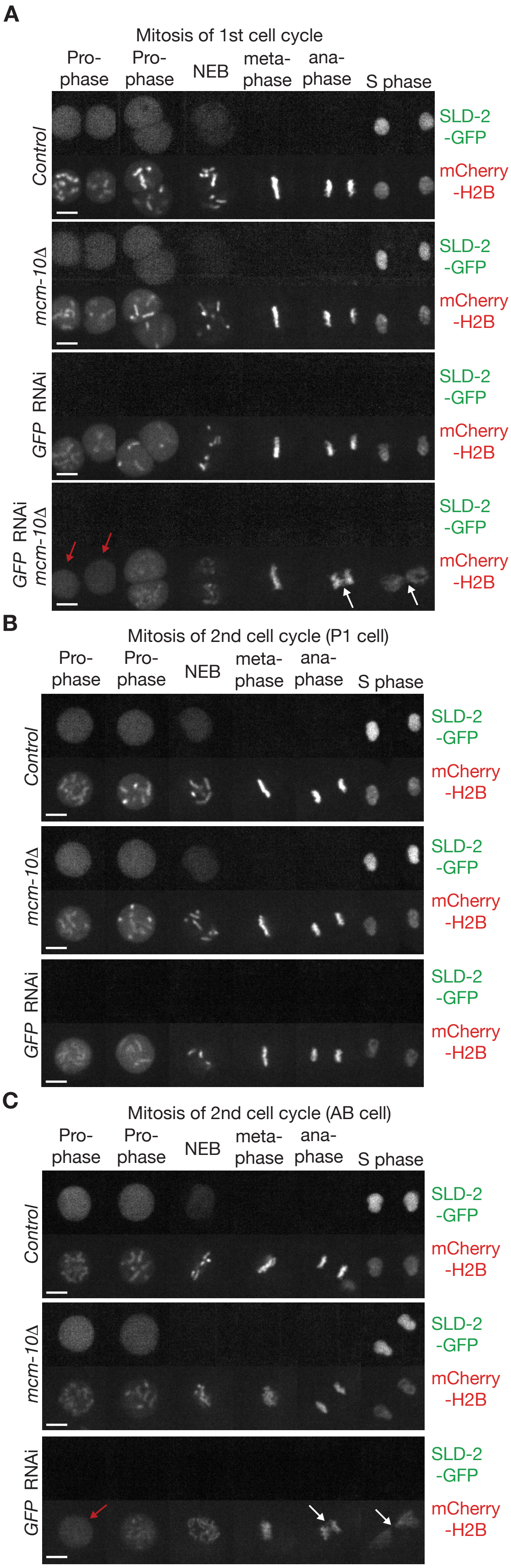

Appendix Figure S1
Mitosis proceeds normally in *mcm-10Δ* worms but fails during the second cell cycle in the AB cell of worms lacking SLD-2.
(**A**) Progression through mitosis of the first embryonic cell cycle was monitored by time-lapse video microscopy of control and *mcm-10Δ* worms expressing SLD-2-GFP and mCherry-HistoneH2B, after treatment as indicated with *GFP* RNAi. Red arrows highlight defective chromosome condensation during prophase, whereas white arrows indicate the failure of chromosome segregation. Both defects were observed in all *mcm-10Δ sld-2-GFP* embryos from worms that had been treated with *GFP* RNAi (n = 5).
(**B**) Progression of the P1 cell through mitosis of the second embryonic cell cycle, in the same experiment as in (A).
(**C**) Progression of the AB cell through mitosis of the second embryonic cell cycle, in the same experiment as in (A). The red arrow indicates the defect in mitotic chromosome condensation in prophase, whereas white arrows denote the failure of chromosome segregation during anaphase. Both defects were observed in all *sld-2-GFP* embryos from worms treated with *GFP* RNAi (n = 5), indicating that cells had entered mitosis before completion of chromosome replication.
Videos of the embryos shown in this figure can be found in Movies EV33-EV36 for (A), EV41-EV43 for (B) and EV43-EV45 for (C).

Appendix Figure S2

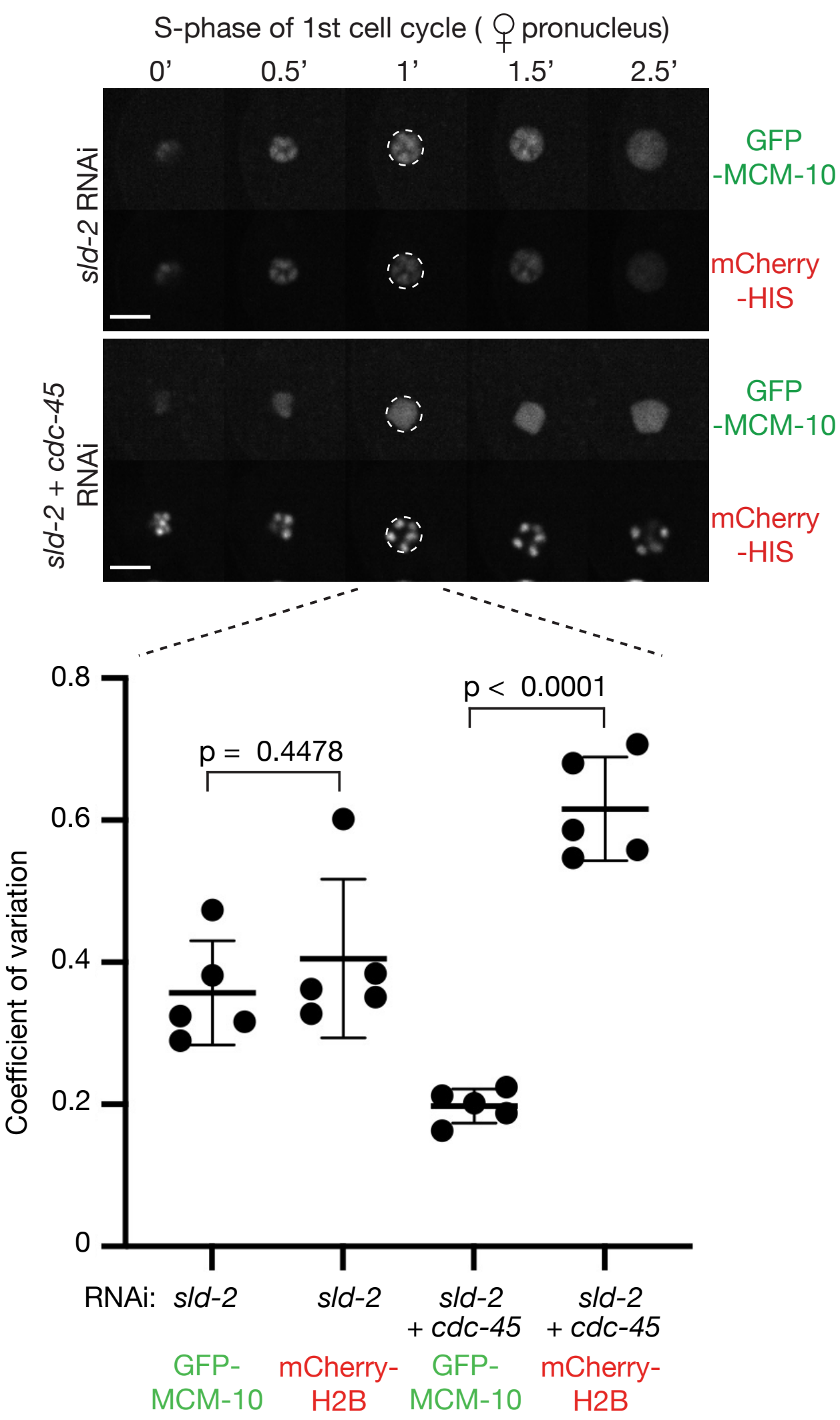

(Coefficient of variation = standard deviation / mean intensity)

Appendix Figure S2
Association of GFP-MCM-10 with chromatin is dependent upon CMG helicase assembly.
Analogous experiment to Figure 2A, with worms expressing GFP-MCM-10 and mCherry-HistoneH2B, treated as shown with *sld-2* RNAi or *sld-2* + *cdc-45* RNAi. Scalebars correspond to 5μm. The lower panel shows quantification of the data from the 1 minute timepoint, as described in Methods.
The coefficient of variation provides a measure of how variable the pixel intensities of GFP-MCM-10 and mCherry-HistoneH2B are within the nucleus.
A higher value is produced when the protein is concentrated on condensed chromosomes, and a lower value corresponds to a more even distribution of the protein throughout the nucleus. The data correspond to five biological repeats (five embryos) with mean and standard deviation. P-values were generated via a two-tailed Welch's t-test. Videos of the embryos shown in this figure can be found in Movies EV37-EV38.

Appendix Figure S3

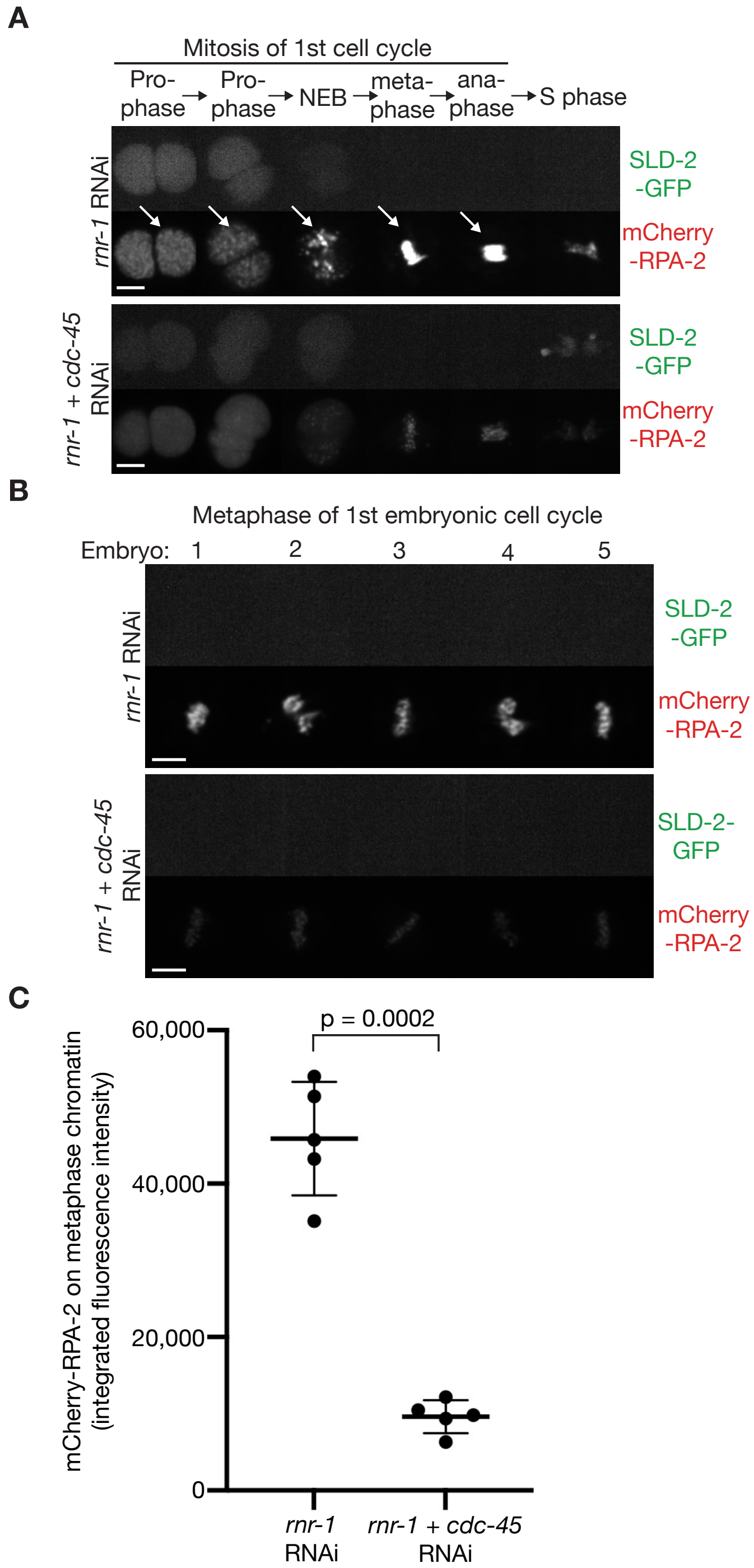

Appendix Figure S3

RPA chromatin association is dependent upon CMG helicase assembly.

(**A**) Analogous experiment to Figure 3H, with worms expressing SLD-2-GFP and mCherry-RPA-2, treated as indicated with *cdc-45* RNAi. Arrows indicate mCherry-RPA-2 on chromatin. All scalebars correspond to 5μm.

(**B**) Five examples of metaphase from the experiment in (A).

(**C**) Quantification of the data in (B). The data correspond to five biological repeats (five embryos) with mean and standard deviation. Integrated fluorescence intensity was calculated as described in Methods. P-values were generated via a two-tailed Welch's t-test.

Videos of the embryos shown in this figure can be found in Movies EV39-EV40.

Appendix Figure S4

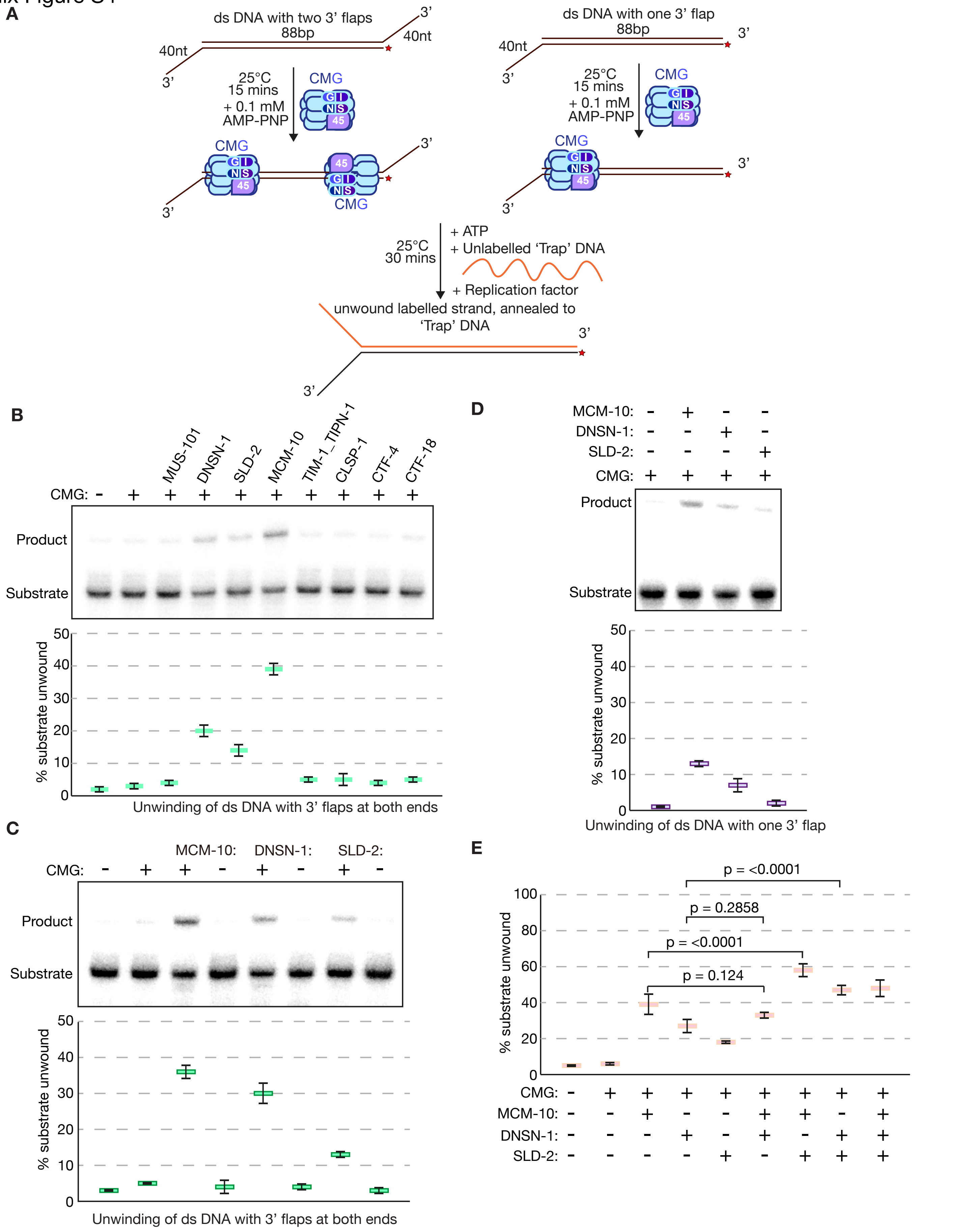

Appendix Figure S4

Stimulation of CMG helicase activity by SLD-2 is additive with MCM-10 or DNSN-1.

(**A**) Reaction scheme (radio-labelling of 5'-end of DNA strand is indicated by a red star).

(**B**) CMG helicase assays with the substrate from (A) containing two 3' flaps, in the presence of the indicated factors. Reaction products were separated in a polyacrylamide gel and detected by autoradiography. The percentage of unwound substrate DNA was quantified and shown in the graph at the bottom. The data correspond to the mean values (green) and standard deviation (black error bars) from three independent experiments.

(**C**) Analogous experiment to (B) in the presence or absence of MCM-10, SLD-2 and DNSN-1 as indicated.

(**D**) Helicase assays were performed as in (A) with the substrate containing one 3' flap, in the presence of the indicated factors. The data correspond to the mean values (purple) and standard deviation (black error bars) from three independent experiments.

(**E**) CMG helicase assays were performed in the presence of the indicated combinations of MCM-10, DNSN-1 and SLD-2. The mean values from three independent experiments are shown (pink) together with corresponding standard deviations (black error bars). P-values were then generated via a one-way ANOVA followed by Dunnett's multiple comparisons test.

Appendix Figure S5

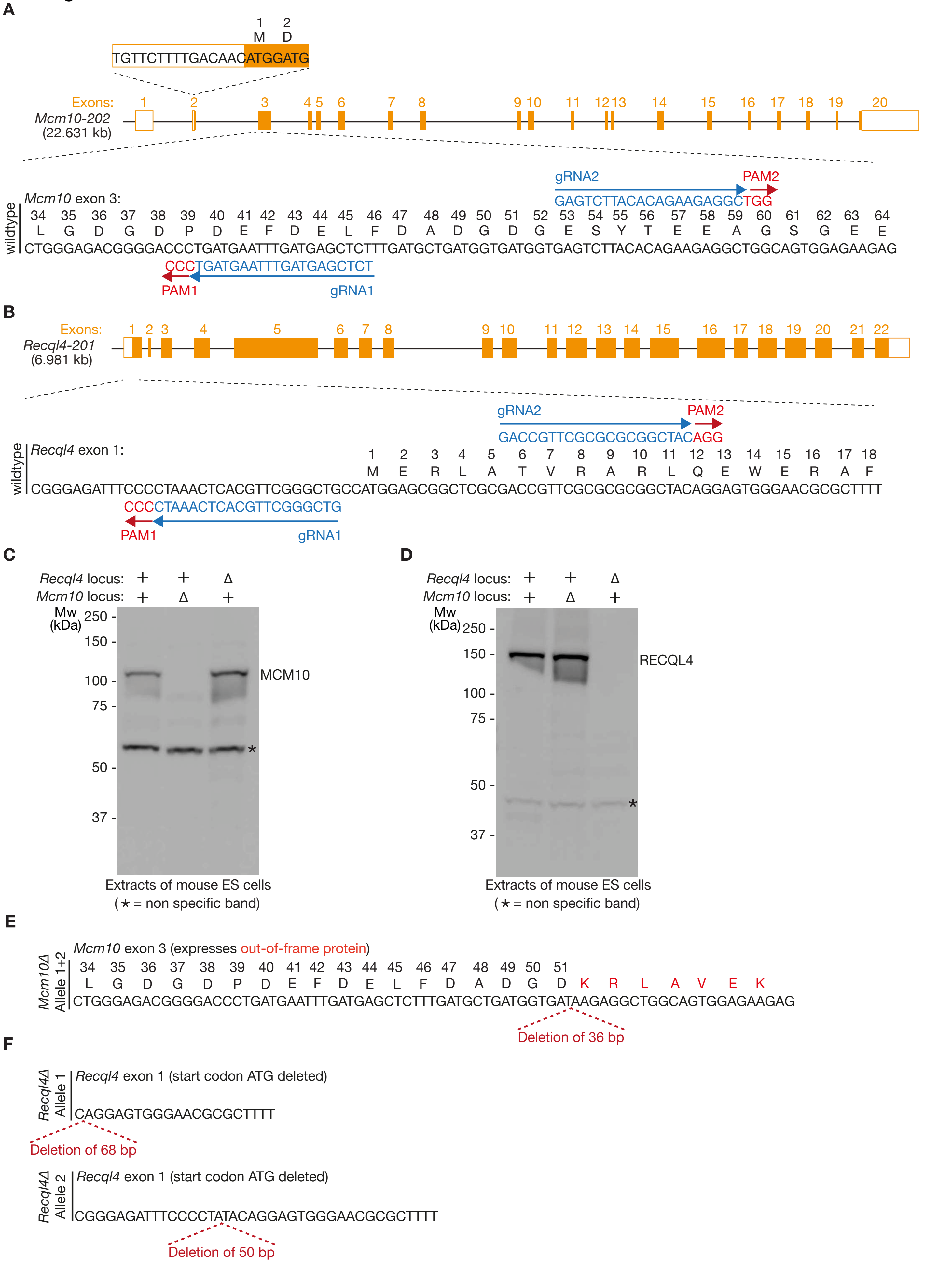

Appendix Figure S5

Small deletions at the start of the coding sequence of *Mcm10* or *Recql4* impair protein expression but do not cause inviability in mouse ES cells.

(**A**) Depiction of the mouse *Mcm10* locus, showing the location of two guide RNAs (gRNAs) that were used in combination with Cas9-D10A to make small deletions in exon 3. PAM = Protospacer Adjacent Motif.

(**B**) Equivalent illustration of the mouse Recql4 locus, with corresponding gRNAs that were used to make small deletions in exon 1.

(**C** - **D**) Clones generated with the gRNAs shown in (A-B) were analysed by immunoblotting.

(**E**) DNA sequence analysis of the *Mcm10Δ* clone from (C).

(**F**) DNA sequence analysis of the *Recql4Δ* clone from (D).

Appendix Figure S6

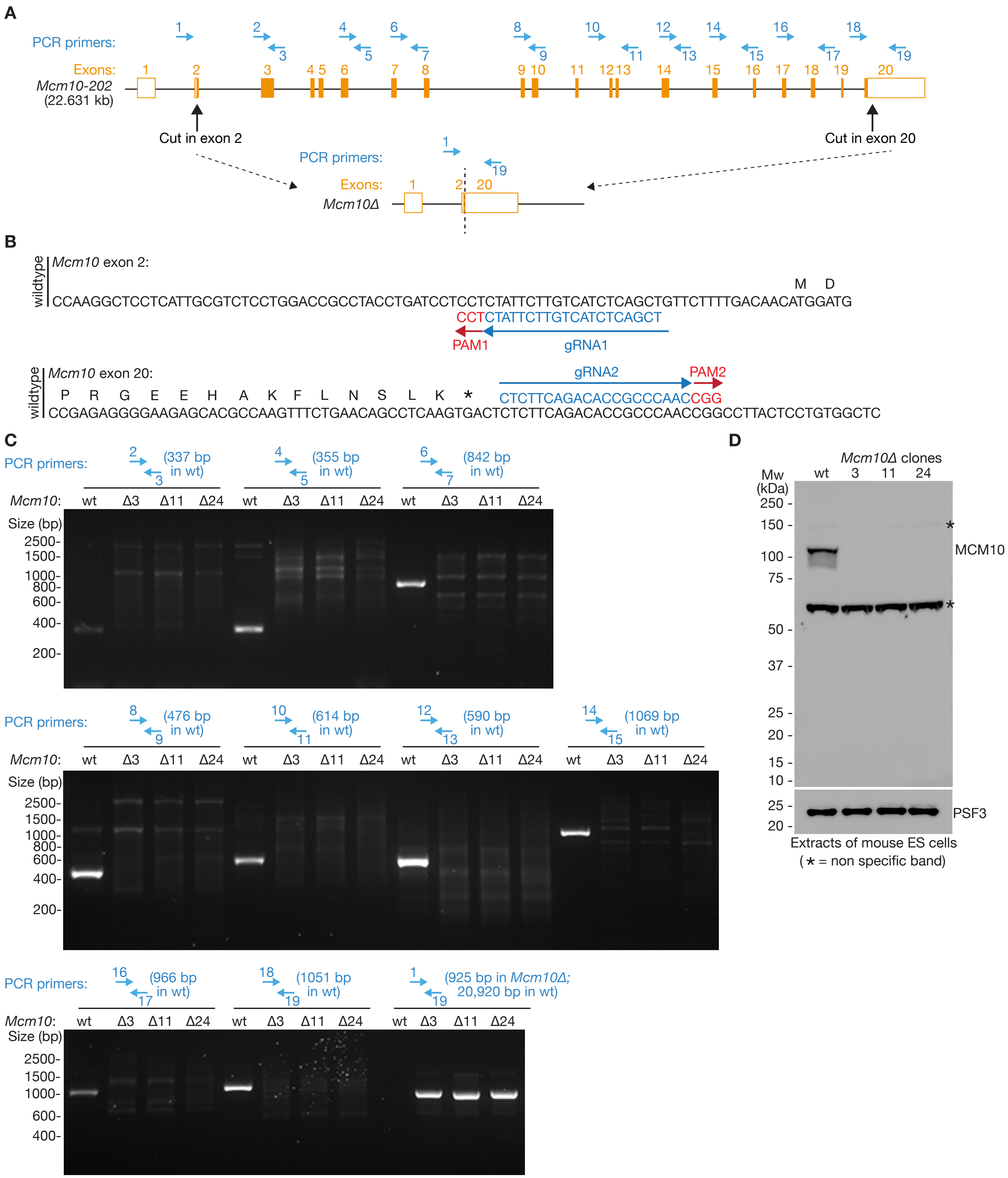

Appendix Figure S6

Complete deletion of the *Mcm10* gene is viable in mouse ES cells.

(**A**) Depiction of the mouse *Mcm10* locus, showing the location of the sites in exon 2 and exon 20 where wild type Cas9 was directed by single gRNAs to cut both strands of the DNA template, leading to deletion of the entire gene (indicated at the bottom). Blue arrows indicate PCR primers that were used to monitor the locus.

(**B**) DNA sequence of the relevant sections of exon 2 and exon 20 of *Mcm10*, showing each of the two guide RNAs (gRNAs) that were used in combination with wild type Cas9. PAM = Protospacer Adjacent Motif.

(**C**) Genomic DNA from wild type mouse ES cells (wt) and three *Mcm10Δ* clones (Δ3, Δ11 and Δ24) was analysed by PCR with the indicated primer pairs. For each pair, the size of the expected band is shown in brackets.

(**D**) Cell extracts of wild type and the three *Mcm10Δ* clones were analysed by immunoblotting.

Appendix Figure S7

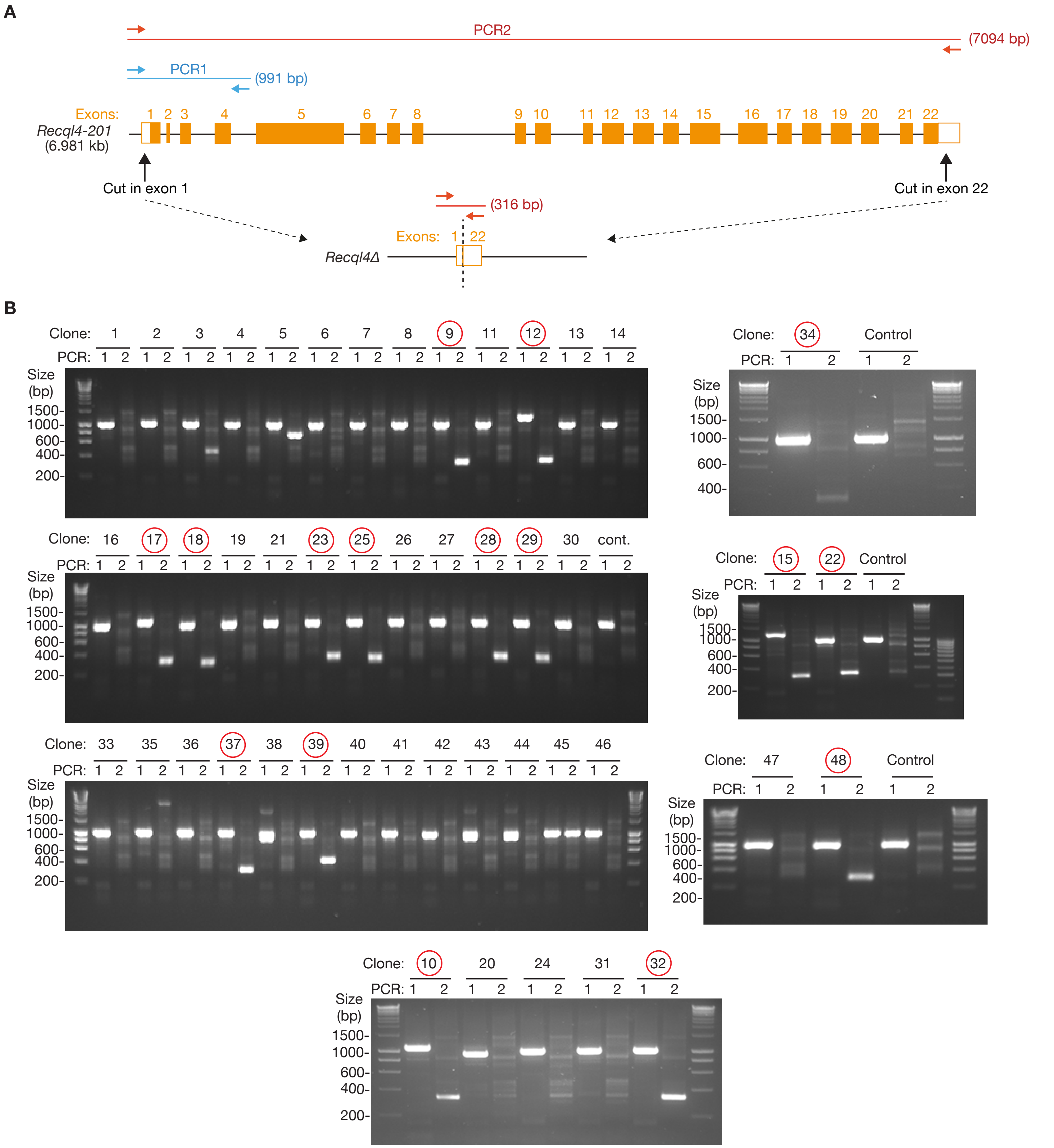

Appendix Figure S7
Complete deletion of the *Recql4* gene is only viable in heterozygous mouse ES cells.
(**A**) Depiction of the mouse *Recql4* locus, showing the location of the sites before exon 1 and in exon 22 where wild type Cas9 was directed by single gRNAs to cut both strands of the DNA template, leading to deletion of the entire gene (indicated at the bottom). PCR1 (blue) produced a 991 bp product that indicates the presence of the wild type locus, whereas PCR2 (red) produced a 316 bp product that is indicative of complete deletion of the Recql4 gene (amplification of the 7094 bp product for PCR2 from the wild type locus was too inefficient under these conditions and so was not observed).
(**B**) Genomic DNA from wild type mouse ES cells (Control), and from 48 candidate *Recql4Δ* clones, was analysed using PCR1 and PCR2 that are summarised in (A). The sixteen clones shown in red were all heterozygous for the Recql4 locus, with one allele producing a PCR2 product that indicates a complete deletion of the *Recql4* gene, and the second generating a PCR1 product of approximately wild type size.

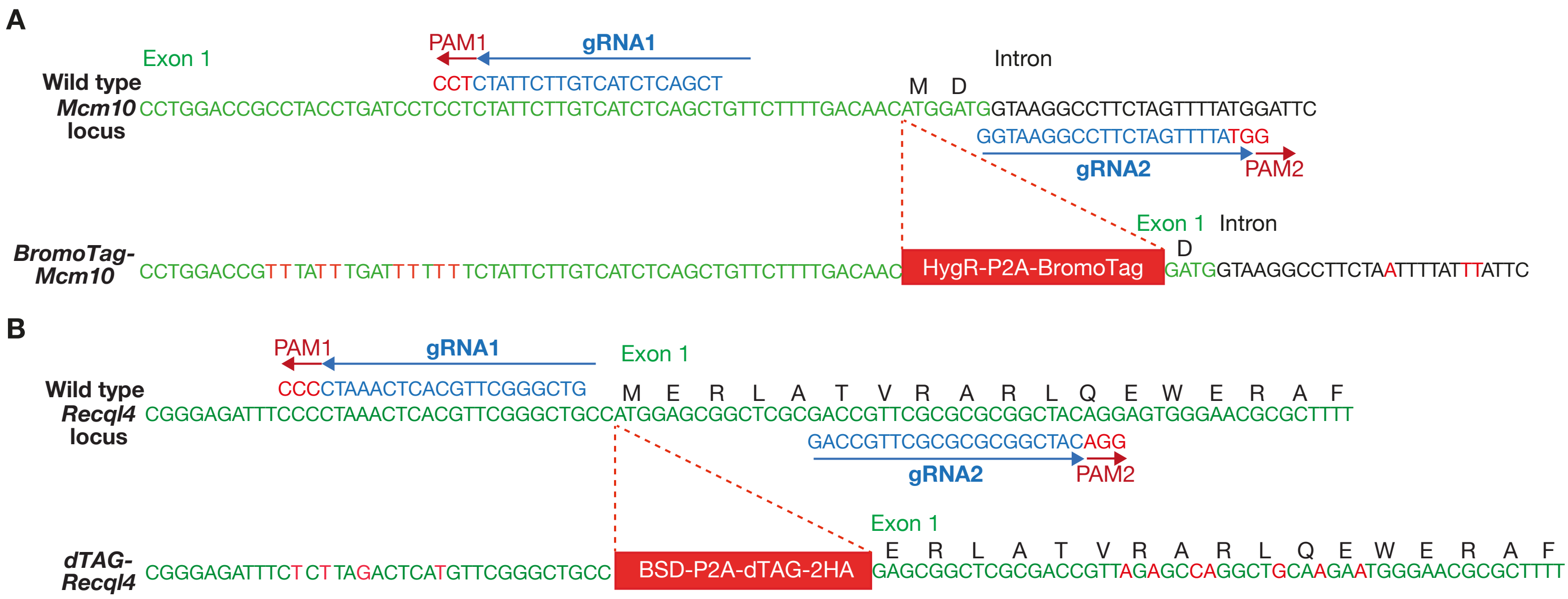

Appendix Figure S8

Generation of *BromoTag-Mcm10 dTAG-Recql4* mouse ES cells by genome editing with CRISPR-Cas9.

(**A**) Exon 1 of the mouse *Mcm10* locus in wild type cells and *BromoTag-Mcm10*. Wild type cells were transfected with plasmids expressing gRNA1 and gRNA2 (PAM = Protospacer Adjacent Motif), together with the Cas9-D10A nickase, and a donor vector to allow repair of the locus after cutting, thereby inserting HygR-P2A-BromoTag (HygR = coding sequence for Hygromycin resistance gene; P2A = ribosome skipping sequence from porcine teschovirus-1 2A, which leads to expression of BromoTag-MCM10 as a separate protein to the HygR-P2A sequence). The 5' and 3' homology sequences in the donor vector also contained silent mutations (shown in red) that prevented re-cleavage by Cas9-D10A.

(**B**) Exon 1 of the mouse *Recql4* locus in wild type cells and dTAG-Recql4. As above, the figure shows the location of the gRNAs that were used to direct Cas9-D10A to exon 1 of Recql4, allowing repair via a donor vector containing BSD-P2A-dTAG-2HA (BSD = coding sequence for Blastocidin resistance gene; P2A leads to expression of dTAG-RECQL4 as a separate protein to the BSD-P2A sequence; 2HA = two copies of the nine amino acid tag from human influenza hemagglutinin (HA) protein). As above, the 5' and 3' homology sequences in the donor vector also contained silent mutations (shown in red) that prevented re-cleavage by Cas9-D10A.

Appendix Figure S9

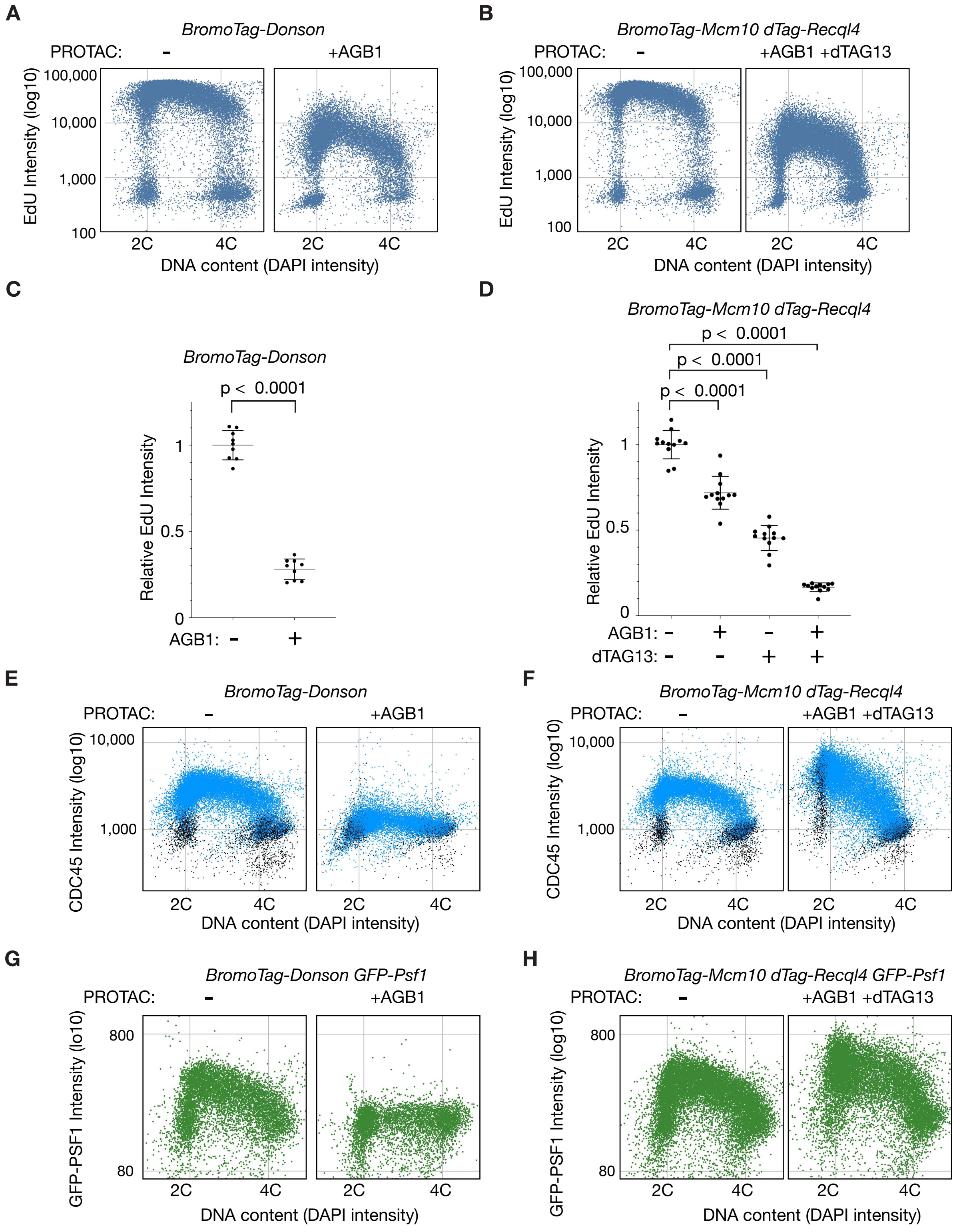

Appendix Figure S9
Accumulation of the CMG helicase on chromatin in mouse ES cells lacking MCM10 and RECQL4.
(**A**-**B**) Data corresponding to Figure 7B+G but on log10 rather than linear scale.
(**C**) Quantification of the data from (A). For each well of a 96-well plate containing cells of a given treatment (-/+ AGB1), the relative EdU intensity per nucleus was calculated as described in Methods. Each dot represents the relative EdU intensity per nucleus from a single well. Mean and standard deviations are shown from three independent experiments. P-values were generated via a two-tailed Welch's t-test.
(**D**) Relative EdU intensity was calculated as above, for the data from (B) and Appendix Figure S10A. Mean and standard deviations are shown from three independent experiments. P-values were generated via a one-way ANOVA followed by Dunnett's multiple comparisons test.
(**E**-**F**) Data corresponding to Figure 7D+I but on log10 rather than linear scale.
(**G**-**H**) Data corresponding to Figure 7E+J but on log10 rather than linear scale.

Appendix Figure S10

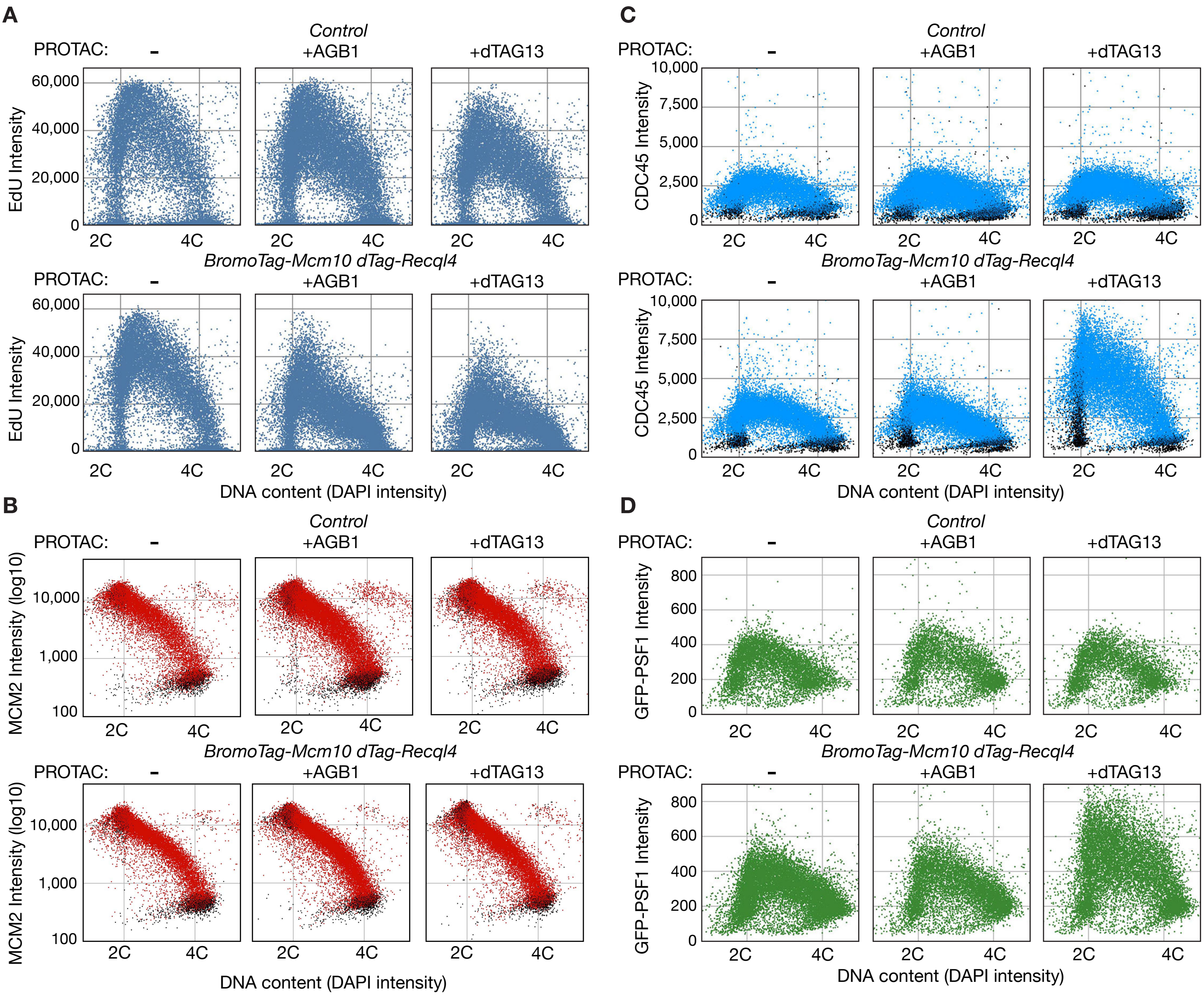

Appendix Figure S10
Depletion of RECQL4 has a greater impact on CMG helicase activation in mouse ES cells than depletion of MCM10.
(**A**-**D**) correspond to data from the experiment in Figure 7F-J, for control and *BromoTag-Mcm10 dTAG-Recql4*, grown for eight hours as indicated in the absence of PROTACs (-), or in the presence of 250 nM AGB1 (+AGB1), or 250 nM dTAG13 (+dTAG13). Cells were processed as described above for Figure 7F-J.
The data for the untreated *BromoTag-Mcm10 dTag-Recql4* cells (-) are reproduced from Figure 7G-J, and are included here for the sake of clarity.

Appendix Figure S11

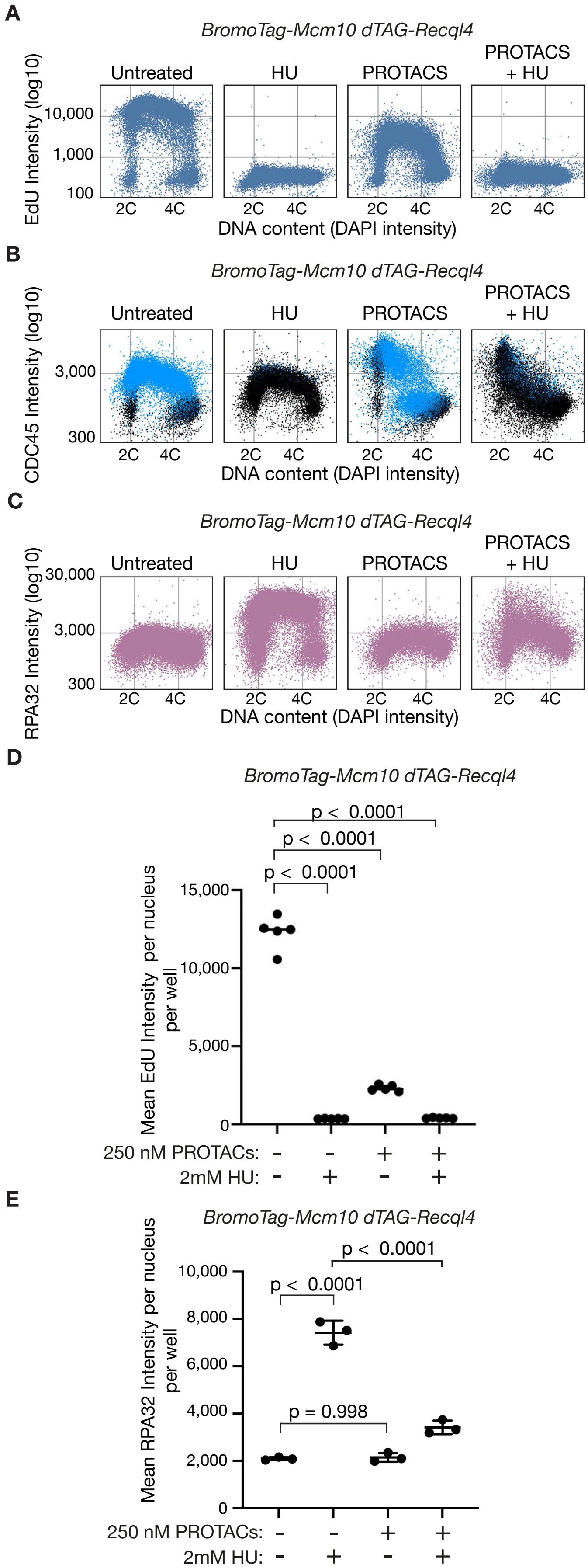

Appendix Figure S11
Depletion of MCM10 and RECQL4 impairs the recruitment of RPA to chromatin.
(**A**-**C**) Data corresponding to Figure 8A+C+D but on log10 rather than linear scale.
(**D**) Quantification of the data from panel (A) and Figure 8A. Mean EdU intensity per nucleus per well was calculated as described in Methods, for each well on a 96-well plate that contained cells with one of the four indicated treatments. Each dot represents the mean EdU intensity per nucleus within a single well containing cells of a particular treatment. Mean and standard deviations are shown, together with P-values that were generated via a one-way ANOVA followed by Dunnett's multiple comparisons test.
(**E**) Quantification of the data from panel (C) and Figure 8D. Mean RPA32 intensity per nucleus per well (representing chromatin-bound RPA32) was calculated as described above for EdU, for each well on a 96-well plate that contained cells with one of the four indicated treatments. Each dot represents the mean RPA32 intensity per nucleus within a single well containing cells of a particular treatment. Mean and standard deviations are shown, together with P-values that were generated via a one-way ANOVA followed by Dunnett's multiple comparisons test.

Appendix Figure S12

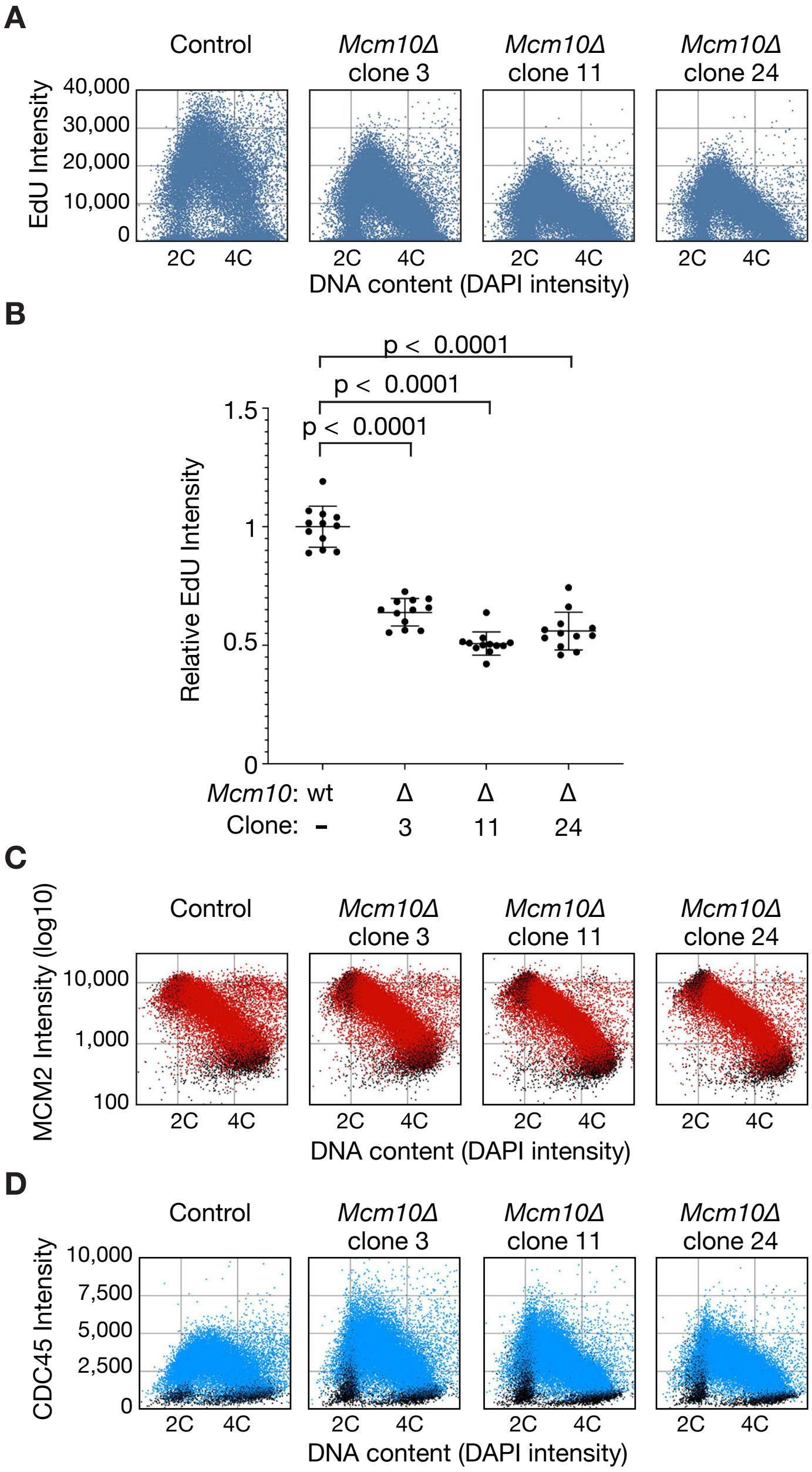

Appendix Figure S12

Complete deletion of the *Mcm10* gene in mouse ES cells causes a modest defect in CMG helicase activation.

(**A**) Control and three clones of *Mcm10Δ* mouse ES cells (Appendix Figure S6) were analysed by high content screening microscopy, as in Figures 7-8, before detection of EdU incorporation.

(**B**) Quantification of the data from panel (A). For each well of a 96-well plate containing wild type mouse ES cells, or the indicated three clones of *Mcm10Δ* cells, the relative EdU intensity was calculated as described in Methods. Each dot represents the relative EdU intensity for a single well. Mean and standard deviations are shown, together with P-values that were generated via a one-way ANOVA followed by Dunnett's multiple comparisons test.
