# Supplementary material for: MCM10 and SLD-2/RECQL4 jointly activate the CMG helicase during metazoan DNA replication initiation
Source: EMBO J. 2026 Sep 28;45(21):7582–620. doi: 10.1038/s44318-026-00915-9 (PMC13728148; doi:10.1038/s44318-026-00915-9)
Supplement: Supplementary file 2 [file 44318_2026_915_MOESM2_ESM.pdf]

THE EMBO JOURNAL

MCM10 and SLD-2/RECQL4 jointly activate the CMG helicase during metazoan DNA replication initiation

Remi Sonneville, Cecile Evrin, Jane Wright, He Huang, Yisui Xia, and Karim Labib

Corresponding author(s): Karim Labib (k.p.m.labib@dundee.ac.uk) , Yisui Xia (yyxia@szu.edu.cn)

Review Timeline:

Submission Date:	6th Feb 26
Editorial Decision:	9th Mar 26
Revision Received:	17th Jul 26
Editorial Decision:	25th Aug 26
Revision Received:	9th Sep 26
Accepted:	11th Sep 26

Editor: Hartmut Vodermaier

Transaction Report:

(Note: With the exception of the correction of typographical or spelling errors that could be a source of ambiguity, letters and reports are not edited. Depending on transfer agreements, referee reports obtained elsewhere may or may not be included in this compilation. Referee reports are anonymous unless the Referee chooses to sign their reports.)

Prof. Karim Labib
University of Dundee
Division of Genome Integrity
Faculty of Life Sciences
Dow Street
Dundee DD1 5EH
United Kingdom

9th Mar 2026

Re: EMBOJ-2026-123821
MCM10 and SLD-2/RECQL4 jointly activate the CMG helicase during metazoan DNA replication initiation

Dear Karim,

Thank you again for submitting your study on DNA replication roles for metazoan counterparts of Mcm10 and Sld2 to The EMBO Journal. We have now received input from three expert referees, copied below for your information. Since all referees acknowledge the interest and importance of the work as well as its overall quality, we would be happy to consider a revised version further for EMBO Journal publication. Nevertheless, each reviewer has a few important concerns, addressing of which should in my view significantly strengthen the insightfulness of the work and its conclusions.

I am therefore inviting you to prepare and resubmit a new version of the manuscript, revised along the lines suggested in the reports below. Should you want to discuss any of these points, or how to best address them, please do not hesitate to get back to me anytime during your revision; this may be helpful in light of our single-major-revision round policy. We would also be open to extending the revision deadline if that should be helpful.

Detailed information on preparing, formatting and uploading a revised manuscript can be found below and in our Guide to Authors, and adhering to them as closely as possible shall greatly facilitate editorial processing upon resubmission. Thank you again for the opportunity to consider this work for The EMBO Journal, and I look forward to your revision in due time.

With kind regards,

Hartmut

Hartmut Vodermaier, PhD
Senior Editor, The EMBO Journal
h.vodermaier@embojournal.org

*** PLEASE NOTE: All revised manuscript are subject to initial checks for completeness and adherence to our formatting guidelines. Revisions may be returned to the authors and delayed in their editorial re-evaluation if they fail to comply to the following requirements. As a first step please read our guidelines for revised submissions:
https://link.springer.com/journal/44318/submission-guidelines#cms-Revised-submissions

1) Every manuscript requires a Data Availability section (even if only stating that no deposited datasets are included). Primary datasets or computer code produced in the current study have to be deposited in appropriate public repositories prior to resubmission, and reviewer access details provided in case that public access is not yet allowed.

2) Each figure legend must specify
- size of the scale bars that are mandatory for all micrograph panels
- the statistical test used to generate error bars and P-values
- the type error bars (e.g., S.E.M., S.D.)
- the number (n) and nature (biological or technical replicate) of independent experiments underlying each data point
- Figures may not include error bars for experiments with n<3; scatter plots showing individual data points should be used instead.

3) Revised manuscript text (including main tables, and figure legends for main and EV figures) has to be submitted as editable text file (e.g., .docx format). We encourage highlighting of changes (e.g., via text color) for the referees' reference.

4) Each main and each Expanded View (EV) figure should be uploaded as individual production-quality files (preferably in .eps,

.tif, .jpg formats). For suggestions on figure preparation/layout, please refer to our Figure Preparation Guidelines: https://media.springernature.com/original/springer-cms/rest/v1/content/27825798/data/v1

5) Point-by-point response letters should include the original referee comments in full together with your detailed responses to them (and to specific editor requests if applicable), and also be uploaded as editable (e.g., .docx) text files.

6) Please complete our Author Checklist, and make sure that information entered into the checklist is also reflected in the manuscript; the checklist will be available to readers as part of the Review Process File.

7) All authors listed as (co-)corresponding need to deposit, in their respective author profiles in our submission system, a unique ORCiD identifier linked to their name. Please see our Guide to Authors for detailed instructions.

8) Please note that supplementary information at EMBO Press has been superseded by the 'Expanded View' for inclusion of additional figures, tables, movies or datasets; with up to five EV Figures being typeset and directly accessible in the HTML version of the article.

9) To facilitate reproducibilty and cross-laboratory adoption of methodologies, please structure the Materials & Methods section as outlined in our guide to authors, including a completed Reagents and Tools Table.

10) Digital image enhancement is acceptable practice, as long as it accurately represents the original data and conforms to community standards. If a figure has been subjected to significant electronic manipulation, this must be clearly noted in the figure legend and/or the 'Materials and Methods' section. The editors reserve the right to request original versions of figures and the original images that were used to assemble the figure. Finally, we generally encourage uploading of numerical as well as gel/blot image source data.

At EMBO Press we ask authors to provide source data for the main manuscript figures. You will receive a separate email with instructions for providing source data with your revised manuscript, including how to upload and organize the files.

In the interest of ensuring the conceptual advance provided by the work, we recommend submitting a revision within 3 months (7th Jun 2026). Please discuss the revision progress ahead of this time with the editor if you require more time to complete the revisions. Use the link below to submit your revision:

Link Not Available

Referee #1:

How the replicative CMG helicase is assembled and activated is an enduring mystery. In yeast, the Sld2 protein is required for CMG assembly, whereas Mcm10 acts after assembly and seems to stimulate the extrusion of one strand from the central channel. In multicellular eukaryotes, the roles of MCM10 and the Sld2 homolog (SLD-2 in worms, RECQL4 in vertebrates) are murky. For example, in worms and frog egg extracts, MCM10 appears dispensable for replication (and worm viability), whereas SLD-2/RECQL4 is essential and the knockout is lethal. On the other hand, in mammals, there are conflicting reports as to the essentiality of RECQL4, and in egg extracts, RECQL4 was reported to function post-CMG activation. Thus, the relative importance for viability, and specific roles in replication, of MCM10 and SLD2/RECQL4 remain rather murky.

In this paper, Labib and colleagues shed much-needed light on these important issues. They showed that although MCM-10 is not essential in worms, its absence delays CMG activation (but not assembly) during the first embryonic cell cycle after fertilization (based on recruitment of CMG components in microscopy but absence of chromosome decondensation or binding of the ssDNA binding protein RPA). Similarly, the absence of SLD-2 delays CMG activation in this early cell cycle, but in the next cell cycle, it becomes essential, explaining the lethal phenotype of the knockout. Interestingly, eliminating MCM-10 leads to accumulation of SLD-2 on chromatin, and vice versa, demonstrating the two proteins can function independently, and consistent with them performing similar functions (but struggling to do so when acting alone).

To explore the roles of MCM10 and RECQL4 in mouse embryonic stem cells, the authors made small deletions at the 5'ends of the coding sequences and showed the resulting cells were viable, even though the gene products were undetectable. However, combining these
hypomorphic mutations led to cell lethality. Similar results were observed with degron alleles. When they deleted the full gene, they were able to obtain viable cells lacking MCM10 but not RECQL4. When MCM10 and RECQL4 were depleted before S phase entry, DNA synthesis was inhibited, but CMG assembly still occurred and the abundance of CMGs on chromatin was enhanced compared to controls. Finally, in the absence of MCM10 and RECQL4, there was no checkpoint signaling

Overall, the data provide strong evidence that MCM10 and RECQL4 both perform overlapping functions after CMG assembly, likely in CMG activation, with RECQL4 being the more important factor. These conclusions are of great importance for the field. The experiments are performed to a high standard and (with one or two exceptions noted below) interpreted appropriately.

Main points
The case for MCM10 and RECQL4 promoting helicase activation in mammals is based on the absence of checkpoint signaling in cells lacking these proteins. However, WT cells also lack checkpoint signaling so the absence of an effect in the mutants is not highly informative. A better experiment is to degrade MCM10 and RECQL4 in the presence of HU, to determine whether their absence suppresses the enhanced checkpoint activation normally observed in the presence of this drug. Also, it would be helpful if the authors could monitor RPA association with chromosomes under these conditions.

The authors speculate that MCM-10, SLD-2, and DNSN-1 function by binding CMG and using their affinity for DNA to help extract one strand from the CMG central channel. However, the CMG helicase assay they use involves forked structures where CMG is likely threaded onto the 3' ssDNA tail, without the need for strand extrusion. These biochemical data therefore do not provide direct support for their proposal, and instead suggest that these factors might activate CMG in additional ways beyond promoting strand extrusion. This should be discussed.

The IPs in Figures 2C and 2F lack controls to show that the interaction of CMG with MCM-10 and SLD-2 is not non-specific. A deletion mutant (as for DNSN-1), or other protein that does not bind to these factors would be important. The authors should at least acknowledge this shortcoming of the data.

Minor Issues
1. Figure 2A: please quantify the MCM10 signal. The effect of cdc-45 RNAi is a bit subtle.

2. Line 221: I am confused by the authors' assertion that SLD-2 does not accumulate on chromosomes in control cells when the SLD-2-GFP signal is clearly visible. Do they mean "condensed chromosomes"?

3. Line 286: "displacement" is not the best description for a loss of affinity.

4. Please specify the nature of the MCM10 allele used (same 96% deletion of coding sequence?)

5. Please label the MW ladder in Figure 4A

Referee #2:

- general summary and opinion about the principle significance of the study, its questions and findings

The roles of Mcm10 and Sld2 in yeast DNA replication have been comparatively well characterized: Sld2 facilitates the CMG helicase assembly by delivering GINS and Pol epsilon to the replication origin, and Mcm10 activates the CMG helicase. By contrast, no clear evidence has been obtained indicating similar roles for their metazoan homologs. Therefore, MCM10 and SLD2/RECQL4 remain among the most enigmatic factors involved in the initiation of metazoan DNA replication.

In this study, Sonneville et al. demonstrated that dual inactivation of MCM10 and SLD-2/RECQL4 causes synthetic lethality both in C. elegans and ES cells without affecting mitotic progression and CMG assembly. They further showed that MCM-10 and SLD-2 were able to directly activate DNA unwinding activity of CMG complex in an in vitro helicase assay. These findings appear to be sufficiently novel for the field. Although it remains unclear how these factors jointly activate CMG helicase, this question can be addressed in future studies. Overall, I believe this manuscript merits publication in The EMBO Journal.

- specific major concerns essential to be addressed to support the conclusions

1. Because vertebrate RecQL4 contains both an N-terminal Sld2 homology domain and a C-terminal RecQ helicase domain, the reduced viability of ES cells following RecQL4 inactivation might be partly due to the loss of the helicase domain. Therefore, distinct mechanisms may underlie the inability to generate double knockout mutants in ES cells and C. elegans. It will be important to determine whether the N-terminal SLD2 domain alone can rescue cell growth in the BT-Mcm10/dT-Recql4 ES cells in Fig.5.

2. In Fig. 4 (and related EVs), CMG helicase activities were examined only in the presence of excess amounts of activators (MCM-10, DNSN-1, SLD-2). What happens when the amounts of these activators are reduced and combined? It would be informative to assess the joint effect on the CMG helicase activation. Although it is unclear whether those activators are required transiently or constitutively, a single activator may mediate the reaction multiple times in combination with another activator.

- minor concerns that should be addressed

1. Regarding the explanation in Lines 234-236, it is uncertain whether chromosome decondensation was delayed in mcm-10Δ+GFP RNAi (Fig. 3A) compared with GFP RNAi (Fig. 1D), because the time-course in Fig. 1D ended at 2.5'. Judging from the mCherry-PSF-1 signal in Fig. 1E, it is likely that mCherry-H2B will also show a decondensed pattern at 3'.
2. In Fig. 3F, why are the mCherry-RPA-2 signals so strong in prophase even in the absence of rnr-1 RNAi? Are they associated with chromatin?
3. In Line 345, "RECQL" should be corrected to "RECQL4".
4. In Fig. 8E, to test whether DNA unwinding occurs by monitoring checkpoint activation, HU should be included in the presence of PROTACs. Without HU, the activation cannot be detected even under control conditions.

- any additional non-essential suggestions for improving the study (which will be at the author's/editor's discretion)

1. If two factors are jointly involved in the same pathway, overexpression of one may rescue the defect caused by the inactivation of the other. Therefore, it would be informative to determine whether overexpression of MCM10 can compensate for the loss of SLD-2 in C. elegans and the loss of RECQL4 in ES cells.

Referee #3:

This study addresses the mechanism of CMG helicase activation in metazoa. While the budding yeast system has long served as a paradigm for eukaryotic DNA replication, recent studies uncovered mechanistic differences between the yeast and metazoan systems, emphasizing the necessity to investigate replication mechanisms in higher eukaryotes. Here, the authors employ C. elegans and mouse ES cells to interrogate the roles of Sld2/RECQL4 and Mcm10 in CMG activation. They demonstrate that the worm and mouse Sld2/RECQL4 and Mcm10 orthologs are partially redundant and jointly essential, functioning downstream of CMG assembly in CMG activation. Since Sld2 is required for CMG assembly in budding yeast, these observations indicate that the mechanism of CMG assembly and activation has diverged in higher eukaryotes.

Overall, the experiments are elegant and of high quality and support the central claims. The combination of the worm and mouse ES cell systems is a major strength of the study and lends weight to the evolutionary conservation of the observed mechanism in higher eukaryotes. The conceptual advance is significant, highlighting differences in the DNA replication initiation mechanisms between yeast and metazoa.

Major limitation of this study:

Based on in vitro helicase assays with purified proteins and oligo-based DNA substrates in Figure 4, the authors claim that C. elegans SLD-2, DNSN-1 and MCM-10 "stimulate" the CMG helicase activity. It should be noted that the presented assays in their current form do not distinguish between a stimulation of DNA substrate unwinding due to increased helicase loading or helicase activity (i.e. increased rate or processivity of unwinding), each of which could explain the observed results. Given the proposed function of SLD-2 and MCM-10 in CMG activation, although it may be technically too challenging to determine helicase rates and processivity, it seems prudent and feasible to test these proteins' impact on CMG-DNA loading. Moreover, the experiment lacks essential controls, such as assessment of the ATP-dependence of DNA unwinding and the impact of individual proteins on DNA substrate unwinding in the absence of CMG. These controls are important in light of the supplemental data indicating DNA binding activity for SLD-2, DNSN-1 and MCM-10 (Figure EV4).

Minor limitations:

Figures 1-3: Chromatin association of GINS/CDC45 is used as a proxy for CMG assembly. This data could be strengthened and complemented by direct demonstration of CMG assembly (e.g. native CMG complex isolation from chromatin, as the authors have done in previous studies).

Figure 8: CMG helicase inactivity is inferred from absence of checkpoint signaling and DNA synthesis, but not directly measured. Perhaps RECQL4 and MCM10 have additional roles in checkpoint signaling? For example, given the little-understood function of the RECQL4 helicase domain, it is conceivable that mammalian orthologues of these replication factors have acquired additional functions in this context.

Referee 1:

"How the replicative CMG helicase is assembled and activated is an enduring mystery. In yeast, the Sld2 protein is required for CMG assembly, whereas Mcm10 acts after assembly and seems to stimulate the extrusion of one strand from the central channel. In multicellular eukaryotes, the roles of MCM10 and the Sld2 homolog (SLD-2 in worms, RECQL4 in vertebrates) are murky. For example, in worms and frog egg extracts, MCM10 appears dispensable for replication (and worm viability), whereas SLD-2/RECQL4 is essential and the knockout is lethal. On the other hand, in mammals, there are conflicting reports as to the essentiality of RECQL4, and in egg extracts, RECQL4 was reported to function post-CMG activation. Thus, the relative importance for viability, and specific roles in replication, of MCM10 and SLD2/RECQL4 remain rather murky.

In this paper, Labib and colleagues shed much-needed light on these important issues. They showed that although MCM-10 is not essential in worms, its absence delays CMG activation (but not assembly) during the first embryonic cell cycle after fertilization (based on recruitment of CMG components in microscopy but absence of chromosome decondensation or binding of the ssDNA binding protein RPA). Similarly, the absence of SLD-2 delays CMG activation in this early cell cycle, but in the next cell cycle, it becomes essential, explaining the lethal phenotype of the knockout. Interestingly, eliminating MCM-10 leads to accumulation of SLD-2 on chromatin, and vice versa, demonstrating the two proteins can function independently, and consistent with them performing similar functions (but struggling to do so when acting alone).

To explore the roles of MCM10 and RECQL4 in mouse embryonic stem cells, the authors made small deletions at the 5'ends of the coding sequences and showed the resulting cells were viable, even though the gene products were undetectable. However, combining these hypomorphic mutations led to cell lethality. Similar results were observed with degron alleles. When they deleted the full gene, they were able to obtain viable cells lacking MCM10 but not RECQL4. When MCM10 and RECQL4 were depleted before S phase entry, DNA synthesis was inhibited, but CMG assembly still occurred and the abundance of CMGs on chromatin was enhanced compared to controls. Finally, in the absence of MCM10 and RECQL4, there was no checkpoint signaling

Overall, the data provide strong evidence that MCM10 and RECQL4 both perform overlapping functions after CMG assembly, likely in CMG activation, with RECQL4 being the more important factor. These conclusions are of great importance for the field. The experiments are performed to a high standard and (with one or two exceptions noted below) interpreted appropriately.

Main points

The case for MCM10 and RECQL4 promoting helicase activation in mammals is based on the absence of checkpoint signaling in cells lacking these proteins. However, WT cells also lack checkpoint signaling so the absence of

an effect in the mutants is not highly informative. A better experiment is to degrade MCM10 and RECQL4 in the presence of HU, to determine whether their absence suppresses the enhanced checkpoint activation normally observed in the presence of this drug. Also, it would be helpful if the authors could monitor RPA association with chromosomes under these conditions.

The authors speculate that MCM-10, SLD-2, and DNSN-1 function by binding CMG and using their affinity for DNA to help extract one strand from the CMG central channel. However, the CMG helicase assay they use involves forked structures where CMG is likely threaded onto the 3' ssDNA tail, without the need for strand extrusion. These biochemical data therefore do not provide direct support for their proposal, and instead suggest that these factors might activate CMG in additional ways beyond promoting strand extrusion. This should be discussed.

The IPs in Figures 2C and 2F lack controls to show that the interaction of CMG with MCM-10 and SLD-2 is not non-specific. A deletion mutant (as for DNSN-1), or other protein that does not bind to these factors would be important. The authors should at least acknowledge this shortcoming of the data.

Minor Issues

1. Figure 2A: please quantify the MCM10 signal. The effect of cdc-45 RNAi is a bit subtle.

2. Line 221: I am confused by the authors' assertion that SLD-2 does not accumulate on chromosomes in control cells when the SLD-2-GFP signal is clearly visible. Do they mean "condensed chromosomes"?

3. Line 286: "displacement" is not the best description for a loss of affinity.

4. Please specify the nature of the MCM10 allele used (same 96% deletion of coding sequence?)

5. Please label the MW ladder in Figure 4A"

We thank the reviewer for their helpful comments and were glad to see the reviewer's conclusion that our data "***shed much-needed light on these important issues***" and ***"provide strong evidence that MCM10 and RECQL4 both perform overlapping functions. These conclusions are of great importance for the field. The experiments are performed to a high standard and (with one or two exceptions noted below) interpreted appropriately***".

Major Issues:

1. "***The case for MCM10 and RECQL4 promoting helicase activation in mammals is based on the absence of checkpoint signaling in cells lacking these proteins. However, WT cells also lack checkpoint signaling so the absence of an effect in the mutants is not highly informative. A better experiment is to degrade MCM10 and RECQL4 in the presence of HU, to determine whether their absence suppresses the enhanced checkpoint***

activation normally observed in the presence of this drug. Also, it would be helpful if the authors could monitor RPA association with chromosomes under these conditions."

We thank the reviewer for this helpful point. In the revised manuscript, we have removed the previous checkpoint data and included data from new experiments that test the effects of degrading MCM10 and RECQL4 before addition of hydroxyurea. In Figure 8A-E and Figure EV5A, we use two different approaches to monitor RPA association under such conditions. We show that RPA accumulates on chromatin, as expected, in response to hydroxyurea, but this is blocked if we first degrade MCM10 and RECQL4 and then add hydroxyurea, consistent with a defect in helicase function.

Moreover, we now show in Figure 8F that checkpoint activation is impaired if we first degrade MCM10 and RECQL4 and then treat cells with hydroxyurea, consistent with the data presented in the original version of the manuscript.

Finally, we also take a new approach to show that the CMG complexes that are assembled in the absence of MCM10 and RECQL4 are inactive. This approach is based on recent structural studies from the Costa and Yeeles groups (Palm et al, 2026 and Taylor-Cross et al, 2026), which indicate that Mcm10 binds to a double CMG intermediate that would exclude binding of the TIMELESS-TIPIN complex (the latter binds at forks to the 'front' of the helicase, on an interface that is hidden within the inactive double CMG structure). Correspondingly, an additional new manuscript on bioRxiv (Weekes et al, 2026), which we also cite in our revised manuscript, shows that yeast Tof1-Csm3 (TIMELESS-TIPIN) do not bind to chromatin when CMG activation is delayed. Inspired by these findings, we have monitored recruitment of the TIMELESS-TIPIN to chromatin when cells enter S-phase in the absence of MCM10 and SLD-2/RECL4, in both *C. elegans* and mouse ES cells. The new data are shown in Figure 3F-G (*C. elegans*), and Figure 8G and Figure EV5B-C (mouse ES cells), showing that recruitment of TIMELESS-TIPIN to chromatin is impaired in the absence of MCM10 and RECQL4.

Collectively and together with the above-discussed new data from other groups, these findings indicate that inactive double CMG complexes are formed in the absence of MCM10 and RECQL4, supporting a role for MCM10 and RECQL4 in the helicase activation step during DNA replication initiation in animal cells.

2. The authors speculate that MCM-10, SLD-2, and DNSN-1 function by binding CMG and using their affinity for DNA to help extract one strand from the CMG central channel. However, the CMG helicase assay they use involves forked structures where CMG is likely threaded onto the 3' ssDNA tail, without the need for strand extrusion. These biochemical data therefore do not provide direct support for their proposal, and instead suggest that these factors might activate CMG in additional ways beyond promoting strand extrusion. This should be discussed.

We take the reviewer's point and now discuss recent structural studies from the Yeeles and Costa labs (Palm et al, 2026 and Taylor-Cross et al, 2026), which indicate that Mcm10 stabilises a specific register of the CMG double hexamer that likely facilitates strand exclusion during initiation and also appears to induce allosteric changes in double CMG (lines 473-477 and 494-507).

In the original version of the manuscript, we showed that CMG-binding is important for stimulation of helicase activity by DNSN-1 (data now in Figure EV3). We now include additional data showing that DNA-binding activity by DNSN-1 is also important for the stimulation of helicase activity (Figure EV4A-D). We also show that

DNSN-1, MCM-10 and SLD-2 do not stimulate loading of CMG onto fork DNA (Figure EV4E-F), suggesting that they stimulate CMG helicase activity in some other way.

To try and address the reviewer's point more directly, we also followed an approach taken by the O'Donnell lab (Langston et al, 2023, doi: 10.1073/pnas.2316466120), to study the role of yeast Mcm10 in stimulating CMG helicase activity on a dsDNA substrate. We monitored the ability of CMG to unwind a double-stranded DNA substrate that has 3' flaps at either or both ends to load CMG, but lacks 5' flaps, so that the CMG helicase complexes should load via the 3' flaps and then move onto dsDNA (Appendix Figure S4A). In this assay, MCM-10, SLD-2 and DNSN-1 all stimulate DNA unwinding by CMG (Appendix Figure S4A-D), in contrast to other tested factors (Appendix Figure S4C). DNA unwinding by CMG in this assay is several times more effective with a substrate that has 3' flaps at both ends (to load two CMG helicases), compared to a dsDNA substrate with one 3' flap (compare Appendix Figure S4B+D). We do not want to over-interpret these data (we only discuss them very simply in the manuscript), and we recognise that more definitive conclusions about the underlying mechanism by which MCM-10 / SLD-2 / DNSN-1 stimulate CMG will require additional structural and biochemical studies, eventually including biochemical reconstitution of the initiation reaction itself. Nevertheless, these findings point to a specific role for MCM-10, SLD-2 and DNSN-1 in stimulating the ability of CMG to unwind DNA, which correlates with their important roles *in vivo*.

3. The IPs in Figures 2C and 2F lack controls to show that the interaction of CMG with MCM-10 and SLD-2 is not non-specific. A deletion mutant (as for DNSN-1), or other protein that does not bind to these factors would be important. The authors should at least acknowledge this shortcoming of the data.

Thanks for pointing this out – we now include new experiments to compare the association of CMG or GINS with MCM-10 and SLD-2, showing that MCM-10 and SLD-2 bind specifically to CMG (Figure 2C+F).

Minor Issues

1. "***Figure 2A: please quantify the MCM10 signal. The effect of cdc-45 RNAi is a bit subtle.***"

Appendix Figure S2 presents a new experiment in which we quantified the GFP-MCM-10 signal on chromatin after *sld-2* RNAi, or *sld-2* + *cdc-45* RNAi. Depletion of SLD-2 delays CMG activation and chromosome decondensation, and GFP-MCM-10 accumulates on chromatin during this period. In worms treated with a combination of *sld-2* + *cdc-45* RNAi, GFP-MCM-10 does not accumulate on chromatin during the delay in chromosome decondensation.

To quantify the data, we measured the heterogeneity [or the variability] of the GFP-MCM-10 and mCherry-H2B signals within the nucleus by calculating the coefficient of variation in each case. When GFP-MCM-10 accumulates on chromatin, the coefficient of variation is similarly high for GFP-MCM-10 and mCherry-H2B. When GFP-MCM-10 does not accumulate on chromatin, the coefficient of variation is lower for GFP-MCM-10 than for mCherry-H2B (Appendix Figure S2, lower panel).

2. "***Line 221: I am confused by the authors' assertion that SLD-2 does not accumulate on chromosomes in control cells when the SLD-2-GFP signal is clearly visible. Do they mean "condensed chromosomes"?*** "

Yes – in the revised manuscript we now state on lines 217-218 that "***SLD-2 does not accumulate on condensed chromosomes when CMG assembly is blocked by depletion of CDC-45 (Figure 2D, cdc-45 RNAi)***."

3. "***Line 286: "displacement" is not the best description for a loss of affinity.***"

Thank you – we now say on lines 293-294 that "***mutation of the MCM-3-binding site led to reduced binding of DNSN-1 to CMG***".

4. "***Please specify the nature of the MCM10 allele used (same 96% deletion of coding sequence?)***".

When we first refer to using the *mcm-10*Δ allele in the revised manuscript (line 133), we cite Xia et al (2023) to indicate that it is indeed the same allele that we reported previously (lacking 96% of the coding sequence, as spelled out on line 110). We also cite Xia et al (2023) when describing the allele in Appendix Table S1.

5. "***Please label the MW ladder in Figure 4A***"

Thank you – we now do this.

Referee 2

"- general summary and opinion about the principle significance of the study, its questions and findings

The roles of Mcm10 and Sld2 in yeast DNA replication have been comparatively well characterized: Sld2 facilitates the CMG helicase assembly by delivering GINS and Pol epsilon to the replication origin, and Mcm10 activates the CMG helicase. By contrast, no clear evidence has been obtained indicating similar roles for their metazoan homologs. Therefore, MCM10 and SLD2/RECQL4 remain among the most enigmatic factors involved in the initiation of metazoan DNA replication.

In this study, Sonneville et al. demonstrated that dual inactivation of MCM10 and SLD-2/RECQL4 causes synthetic lethality both in C. elegans and ES cells without affecting mitotic progression and CMG assembly. They further showed that MCM-10 and SLD-2 were able to directly activate DNA unwinding activity of CMG complex in an in vitro helicase assay. These findings appear to be sufficiently novel for the field. Although it remains unclear how these factors jointly activate CMG helicase, this question can be addressed in future studies. Overall, I believe this manuscript merits publication in The EMBO Journal.

- specific major concerns essential to be addressed to support the conclusions

1. Because vertebrate RecQL4 contains both an N-terminal Sld2 homology domain and a C-terminal RecQ helicase domain, the reduced viability of ES cells following RecQL4 inactivation might be partly due to the loss of the helicase domain. Therefore, distinct mechanisms may underlie the inability to generate double knockout mutants in ES cells and C. elegans. It will be important to determine whether the N-terminal SLD2 domain alone can rescue cell growth in the BT-Mcm10/dT-Recql4 ES cells in Fig.5.

2. In Fig. 4 (and related EVs), CMG helicase activities were examined only in the presence of excess amounts of activators (MCM-10, DNSN-1, SLD-2). What happens when the amounts of these activators are reduced and combined? It would be informative to assess the joint effect on the CMG helicase activation. Although it is unclear whether those activators are required transiently or constitutively, a single activator may mediate the reaction multiple times in combination with another activator.

- minor concerns that should be addressed

1. Regarding the explanation in Lines 234-236, it is uncertain whether chromosome decondensation was delayed in mcm-10Δ+GFP RNAi (Fig. 3A) compared with GFP RNAi (Fig. 1D), because the time-course in Fig. 1D ended at 2.5'. Judging from the mCherry-PSF-1 signal in Fig. 1E, it is likely that mCherry-H2B will also show a decondensed pattern at 3'.
2. In Fig. 3F, why are the mCherry-RPA-2 signals so strong in prophase even in

the absence of rnr-1 RNAi? Are they associated with chromatin?
3. In Line 345, "RECQL" should be corrected to "RECQL4".
4. In Fig. 8E, to test whether DNA unwinding occurs by monitoring checkpoint activation, HU should be included in the presence of PROTACs. Without HU, the activation cannot be detected even under control conditions.

- any additional non-essential suggestions for improving the study (which will be at the author's/editor's discretion)

1. If two factors are jointly involved in the same pathway, overexpression of one may rescue the defect caused by the inactivation of the other. Therefore, it would be informative to determine whether overexpression of MCM10 can compensate for the loss of SLD-2 in C. elegans and the loss of RECQL4 in ES cells."

We thank the reviewer for their helpful comments and were glad to see the reviewer's conclusion that "***Overall, I believe this manuscript merits publication in The EMBO Journal.***"

Major issues

1. "***Because vertebrate RecQL4 contains both an N-terminal Sld2 homology domain and a C-terminal RecQ helicase domain, the reduced viability of ES cells following RecQL4 inactivation might be partly due to the loss of the helicase domain. Therefore, distinct mechanisms may underlie the inability to generate double knockout mutants in ES cells and C. elegans. It will be important to determine whether the N-terminal SLD2 domain alone can rescue cell growth in the BT-Mcm10/dT-Recql4 ES cells in Fig.5.***"

Figure 5C-D now include new data from two clones of cells expressing RECQL4 1-468 (SLD-2 homology without the helicase domain), showing that RECQL4 1-468 can rescue cell growth after depletion of BromoTag-MCM10 and dTAG-RECQL4.

2. "***In Fig. 4 (and related EVs), CMG helicase activities were examined only in the presence of excess amounts of activators (MCM-10, DNSN-1, SLD-2). What happens when the amounts of these activators are reduced and combined? It would be informative to assess the joint effect on the CMG helicase activation. Although it is unclear whether those activators are required transiently or constitutively, a single activator may mediate the reaction multiple times in combination with another activator.***"

Figure 4H now includes new data to show that stimulation of CMG by MCM-10 is epistatic with DNSN-1. In contrast, the effect of SLD-2 is additive with DNSN-1 and with MCM-10 (though in the latter case the effect was not statistically significant under the experimental conditions). To explore this issue further, we also used a new substrate that is much harder for CMG to unwind, since it comprises double strand DNA that only has 3' flaps for loading of CMG but does not have 5' flaps, meaning that CMG would be predicted to slide onto dsDNA. New data in Appendix Figure S4C-D shows that CMG cannot unwind such a substrate on its own but does so in the presence of MCM-10, SLD-2 or DNSN-1 (but not other CMG partner proteins). Appendix Figure S4E shows that the stimulation of CMG by MCM-10 is

epistatic with DNSN-1, whereas the effect of SLD-2 is additive (and statistically significant) with either DNSN-1 or MCM-10.

These data also raise the possibility that MCM-10, SLD-2 and DNSN-1 can stimulate two converging CMG complexes to unwind dsDNA (more like the reaction during CMG helicase activation at origins). However, we don't want to over-interpret these data, as MCM-10, SLD-2 and DNSN-1 also have a limited ability to stimulate CMG to unwind a substrate with a single 3' flap (Appendix Figure S4B), which complicates interpretation as this substrate should only load a single CMG, rather than two converging CMG complexes. These data are discussed briefly and simply in the text, focusing on the additive or epistatic effects of MCM-10, SLD-2 and DNSN-1 (lines 304-305), which is the most relevant point in the context of our experiments.

<u>Minor issues:</u>

1. "***Regarding the explanation in Lines 234-236, it is uncertain whether chromosome decondensation was delayed in mcm-10Δ+GFP RNAi (Fig. 3A) compared with GFP RNAi (Fig. 1D), because the time-course in Fig. 1D ended at 2.5'. Judging from the mCherry-PSF-1 signal in Fig. 1E, it is likely that mCherry-H2B will also show a decondensed pattern at 3'.***"

We have now revised Figure 1D-E so that they show the same timepoints (up to four minutes) as in Figure 3A. This supports our original interpretation, namely that chromosome decondensation is delayed in *mcm-10Δ* +GFP RNAi (Fig. 3A) compared with GFP RNAi (Fig. 1D).

To help the readers assess the imaging data that represent stills from videos of early *C. elegans* embryos in this study, we now also include all the corresponding videos as 'Appendix Movies S1-S45'.

2. "***In Fig. 3F, why are the mCherry-RPA-2 signals so strong in prophase even in the absence of rnr-1 RNAi? Are they associated with chromatin?***"

These are foci of RPA-2 that accumulate on chromatin when DNA synthesis is blocked (they are the source of the RPA-2 that is observed on chromatin during mitosis).

To demonstrate this more clearly, we now include a new experiment in Appendix Figure S3, comparing worms treated either with *rnr-1* RNAi (mCherry-RPA-2 foci are seen on chromatin during prophase, metaphase and anaphase) or with double *rnr-1* + *cdc-45* RNAi (the mCherry-RPA-2 foci are suppressed during prophase/metaphase/anaphase, indicating that the foci reflect RPA on chromatin, when CMG continues unwinding DNA after inhibition of DNA synthesis).

3. "***In Line 345, "RECQL" should be corrected to "RECQL4***".

Thank you – we have now corrected this (line 362 of the revised manuscript).

4. "***In Fig. 8E, to test whether DNA unwinding occurs by monitoring checkpoint activation, HU should be included in the presence of PROTACs. Without HU, the activation cannot be detected even under control conditions.***"

We thank the reviewer for this helpful point, which was also raised by Reviewer 1. As noted above, we use several different approaches to address this point in the revised manuscript.

In Figure 8A-E and Figure EV5A, we use two different approaches to monitor RPA association under such conditions. We show that RPA accumulates on

chromatin, as expected, in response to hydroxyurea, but this is blocked if we first degrade MCM10 and RECQL4 and then add hydroxyurea, consistent with a defect in helicase function.

Moreover, we now show in Figure 8F that checkpoint activation is impaired if we first degrade MCM10 and RECQL4 and then treat cells with hydroxyurea, consistent with the data presented in the original version of the manuscript.

Finally, we also take a new approach to show that the CMG complexes that are assembled in the absence of MCM10 and RECQL4 are inactive. This approach is based ons recent structural studies from the Costa and Yeeles groups (Palm et al, 2026 and Taylor-Cross et al, 2026), which indicate that Mcm10 binds to a double CMG intermediate that would exclude binding of the TIMELESS-TIPIN complex (the latter binds at forks to the 'front' of the helicase, on an interface that is hidden within the inactive double CMG structure). Correspondingly, an additional new manuscript on bioRxiv (Weekes et al, 2026) that we cite in our revised manuscript shows that yeast Tof1-Csm3 (TIMELESS-TIPIN) do not bind to chromatin when CMG activation is delayed. Inspired by these findings, we have monitored recruitment of the TIMELESS-TIPIN to chromatin when cells enter S-phase in the absence of MCM10 and SLD-2/RECL4, in both *C. elegans* and mouse ES cells. The new data are shown in Figure 3F-G (*C. elegans*), and Figure 8G and Figure EV5B-C (mouse ES cells), showing that recruitment of TIMELESS-TIPIN to chromatin is impaired in the absence of MCM10 and RECQL4.

Collectively and together with the above-discussed new data from other groups, these findings indicate that inactive double CMG complexes are formed in the absence of MCM10 and RECQL4, supporting a role for MCM10 and RECQL4 in the helicase activation step during DNA replication initiation in animal cells.

Additional non-essential suggestion for improving the study (which will be at the author's/editor's discretion)

1. "***If two factors are jointly involved in the same pathway, overexpression of one may rescue the defect caused by the inactivation of the other. Therefore, it would be informative to determine whether overexpression of MCM10 can compensate for the loss of SLD-2 in C. elegans and the loss of RECQL4 in ES cells***".

Unfortunately, we do not have the expression systems to test this idea in *C. elegans* and we did not have the time to attempt this in mouse ES cells.

<u>Referee 3</u>

"***This study addresses the mechanism of CMG helicase activation in metazoa. While the budding yeast system has long served as a paradigm for eukaryotic DNA replication, recent studies uncovered mechanistic differences between the yeast and metazoan systems, emphasizing the necessity to investigate replication mechanisms in higher eukaryotes. Here, the authors employ C. elegans and mouse ES cells to interrogate the roles of Sld2/RECQL4 and Mcm10 in CMG activation. They demonstrate that the worm and mouse Sld2/RECQL4 and Mcm10 orthologs are partially redundant and jointly essential, functioning downstream of CMG assembly in CMG activation. Since Sld2 is required for CMG assembly in budding yeast, these observations indicate that the mechanism of CMG assembly and activation has diverged in higher eukaryotes.***

Overall, the experiments are elegant and of high quality and support the central claims. The combination of the worm and mouse ES cell systems is a major strength of the study and lends weight to the evolutionary conservation of the observed mechanism in higher eukaryotes. The conceptual advance is significant, highlighting differences in the DNA replication initiation mechanisms between yeast and metazoa.

Major limitation of this study:

Based on in vitro helicase assays with purified proteins and oligo-based DNA substrates in Figure 4, the authors claim that C. elegans SLD-2, DNSN-1 and MCM-10 "stimulate" the CMG helicase activity. It should be noted that the presented assays in their current form do not distinguish between a stimulation of DNA substrate unwinding due to increased helicase loading or helicase activity (i.e. increased rate or processivity of unwinding), each of which could explain the observed results. Given the proposed function of SLD-2 and MCM-10 in CMG activation, although it may be technically too challenging to determine helicase rates and processivity, it seems prudent and feasible to test these proteins' impact on CMG-DNA loading. Moreover, the experiment lacks essential controls, such as assessment of the ATP-dependence of DNA unwinding and the impact of individual proteins on DNA substrate unwinding in the absence of CMG. These controls are important in light of the supplemental data indicating DNA binding activity for SLD-2, DNSN-1 and MCM-10 (Figure EV4).

Minor limitations:

Figures 1-3: Chromatin association of GINS/CDC45 is used as a proxy for CMG assembly. This data could be strengthened and complemented by direct demonstration of CMG assembly (e.g. native CMG complex isolation from chromatin, as the authors have done in previous studies).

Figure 8: CMG helicase inactivity is inferred from absence of checkpoint signaling and DNA synthesis, but not directly measured. Perhaps RECQL4 and

***MCM10 have additional roles in checkpoint signaling? For example, given the little-understood function of the RECQL4 helicase domain, it is conceivable that mammalian orthologues of these replication factors have acquired additional functions in this context.*"**

We thank the reviewer for their helpful comments and were glad to see the reviewer's summary that "***Overall, the experiments are elegant and of high quality and support the central claims. The combination of the worm and mouse ES cell systems is a major strength of the study and lends weight to the evolutionary conservation of the observed mechanism in higher eukaryotes. The conceptual advance is significant, highlighting differences in the DNA replication initiation mechanisms between yeast and metazoa.*"**

<u>Major issue</u>

"***Based on in vitro helicase assays with purified proteins and oligo-based DNA substrates in Figure 4, the authors claim that C. elegans SLD-2, DNSN-1 and MCM-10 "stimulate" the CMG helicase activity. It should be noted that the presented assays in their current form do not distinguish between a stimulation of DNA substrate unwinding due to increased helicase loading or helicase activity (i.e. increased rate or processivity of unwinding), each of which could explain the observed results. Given the proposed function of SLD-2 and MCM-10 in CMG activation, although it may be technically too challenging to determine helicase rates and processivity, it seems prudent and feasible to test these proteins' impact on CMG-DNA loading. Moreover, the experiment lacks essential controls, such as assessment of the ATP-dependence of DNA unwinding and the impact of individual proteins on DNA substrate unwinding in the absence of CMG. These controls are important in light of the supplemental data indicating DNA binding activity for SLD-2, DNSN-1 and MCM-10 (Figure EV4).***"

Figure EV4E-F present new data to show that SLD-2, DNSN-1 and MCM-10 do not detectably increase CMG loading onto fork DNA. Nevertheless, new data in Figure EV4A-D show that DNA binding by DNSN-1 is important for the observed stimulation of CMG *in vitro*. We note that recent studies indicate that DNA binding by Mcm10 is not essential for its role during initiation in yeast, but it remains possible that DNA binding by Mcm10 is redundant with DNA binding by Sld2 (or by SLD-2 or DNSN-1 in metazoa). These data are discussed in the revised text on lines 482-486.

In addition, new data in Figure 4C show ATP-dependence of the observed DNA unwinding in our assay conditions.

We already included data in the original version of the manuscript to show the impact of MCM-10, SLD-2 or DNSN-1 in the absence of CMG (this was in Figure EV3) – for clarity, we have now moved these data to Figure 4E-G.

<u>Minor issues</u>

1. "***Figures 1-3: Chromatin association of GINS/CDC45 is used as a proxy for CMG assembly. This data could be strengthened and complemented by direct demonstration of CMG assembly (e.g. native CMG complex isolation from chromatin, as the authors have done in previous studies).***"

We take the reviewer's point, but sadly this is not possible for technical reasons. When we monitored CMG by IP in our previous work, this was under conditions where CMG assembly and activation proceed as normal, but CMG

disassembly is blocked. This means that we could harvest embryos from cultures of worms, which would contain many thousands of cells that contained large numbers of CMG complexes for us to analyse. In contrast, if we block CMG activation by depletion of MCM-10 and SLD-2, then embryos die in the first couple of cell cycles. This means that we would end up with much less material, prohibiting us from carrying out IP analysis (the control would have lots of CMG, but the mutant would have much less.

2. "***Figure 8: CMG helicase inactivity is inferred from absence of checkpoint signaling and DNA synthesis, but not directly measured. Perhaps RECQL4 and MCM10 have additional roles in checkpoint signaling? For example, given the little-understood function of the RECQL4 helicase domain, it is conceivable that mammalian orthologues of these replication factors have acquired additional functions in this context.***"

We thank the reviewer for this helpful point, which relates to points raised by Reviewers 1 and 2. We have used several different approaches to address this point further in the revised manuscript.

In Figure 8A-E and Figure EV5A, we use two different approaches to monitor RPA association under such conditions. We show that RPA accumulates on chromatin, as expected, in response to hydroxyurea, but this is blocked if we first degrade MCM10 and RECQL4 and then add hydroxyurea, consistent with a defect in helicase function.

In addition, we also take a new approach to show that the CMG complexes that are assembled in the absence of MCM10 and RECQL4 are inactive. This approach was inspired by recent structural studies from the Costa and Yeeles groups (Palm et al, 2026 and Taylor-Cross et al, 2026), which indicate that Mcm10 binds during initiation to a double CMG intermediate that would exclude binding of the TIMELESS-TIPIN complex (the latter binds at forks to the 'front' of the helicase, on an interface that is hidden within the inactive double CMG structure). Correspondingly, an additional new manuscript on bioRxiv (Weekes et al, 2026) that we cite in our revised manuscript shows that yeast Tof1-Csm3 (TIMELESS-TIPIN) do not bind to chromatin when CMG activation is delayed. Inspired by these findings, we monitored recruitment of the TIMELESS-TIPIN to chromatin when cells enter S-phase in the absence of MCM10 and SLD-2/RECL4, in both *C. elegans* and mouse ES cells. The new data are shown in Figure 3F-G (*C. elegans*), and Figure 8G and Figure EV5B-C (mouse ES cells), showing that recruitment of TIMELESS-TIPIN to chromatin is impaired in the absence of MCM10 and RECQL4.

Collectively and together with the above-discussed new data from other groups, these findings indicate that inactive double CMG complexes are formed in the absence of MCM10 and RECQL4, supporting a role for MCM10 and RECQL4 in the helicase activation step during DNA replication initiation in animal cells.

Prof. Karim Labib
University of Dundee
Division of Genome Integrity
Faculty of Life Sciences
Dow Street
Dundee DD1 5EH
United Kingdom

25th Aug 2026

Re: EMBOJ-2026-123821R
MCM10 and SLD-2/RECQL4 jointly activate the CMG helicase during metazoan DNA replication initiation

Dear Karim,

Thank you for submitting your revised manuscript on replicative helicase activation to The EMBO Journal. Two of the original referees have now reviewed it once more, and overall found the study significantly strengthened and most major concerns adequately addressed. Referee 1 retains a few presentational reservations, which I would ask you to incorporate in a final round of minor revision. In addition, there are still a few editorial modifications to be made at this stage:

- Importantly, our routine pre-acceptance image checks indicated a potentially inadvertent image duplication within Figure 5C, and a re-display of a FACS control image between Figure 7 and Appendix Figure S10, which is however not explicitly mentioned and rationalized in the respective figure legends - please carefully double-check and clarify! Also, please make sure to upload the Expanded View figures at higher resolution/in production quality, as especially blots appeared too pixelated during our image analyes.

- Please carefully go through the reference list and make sure that all references have their complete citation information - including year, journal name & volume, and page/elocator numbers - some of this information is currently missing for several of them.

- For preprint citations: Please change in-text reference format according to "preprint: XXX et al, 2025"; and in the reference list, name of the platform plus DOI + preprint label - e.g. "bioRxiv doi: 1234/002.dfj123 [PREPRINT]". For preprints that have in the meantime been accepted, please try to already include final citation information where possible, or update the preprint citation during proof stage.

- Please double-check all in-text figure references, making sure that all Appendix figures and tables are always called out as such (e.g. 'Appendix Figure S9E', 'Appendix Table S1', etc.).

- The Data Availability section should only refer to any newly generated datasets deposited in public repositories; should there be none, all it should state is "This study includes no data deposited in external repositories."

- Please amend the ToC on the title page of the Appendix with page numbers, and remove the information re. 'Appendix Movies'. Instead, all video files should be renamed into Expanded View Movies (within their legends, file names, and in-text references: 'Movie EV1/2/3...'). Apart from this, they are correctly uploaded in ZIP format.

- Please move the Reagents and Tools table from the Appendix file, and upload it as a separate text file. Also, please make sure to adhere to the template table downloadable from our author guidelines:
https://www.embopress.org/page/journal/14693178/authorguide#structuredmethods

- Please provide suggestions for a short 'blurb' text prefacing and summing up the conceptual aspect of the study in two sentences (max. 250 characters), followed by 3-5 one-sentence 'bullet points' with brief factual statements of key results of the paper; they will form the basis of an editor-written 'Synopsis' accompanying the online version of the article. Please also upload a synopsis image, which can be used as a "visual title" for the synopsis section of your paper. The image should be in JPG format and exactly 550 pixels wide and 300 pixels high, and please make sure that it remains clear enough even in these modest dimensions.

- Finally, during routine pre-acceptance checks, our data editors have raised the following queries regarding figures, data, and legends; I would appreciate if you briefly answered to them in the cover letter of your final submission, and made the requested text modifications with changes/additions highlighted via the "Track changes" option, to facilitate our final checking.
1. Please provide the exact p values in the legend of figure 1C
2. Please proved information related to n in the legends of figures 4F, G

3. Please define the error bars in the legends of figures 1C, 4F, G

I am returning the manuscript to you for minor revision, hoping you will be able to return the final modified files within one week. Once we will have received them, we should hopefully be able to swiftly proceed with formal acceptance and production of the manuscript.

Yours sincerely,

Hartmut

Hartmut Vodermaier, PhD
Senior Editor, The EMBO Journal
h.vodermaier@embojournal.org

*** PLEASE NOTE: All revised manuscript are subject to initial checks for completeness and adherence to our formatting guidelines. Revisions may be returned to the authors and delayed in their editorial re-evaluation if they fail to comply to the following requirements. As a first step please read our guidelines for revised submissions:
https://link.springer.com/journal/44318/submission-guidelines#cms-Revised-submissions

1) Every manuscript requires a Data Availability section (even if only stating that no deposited datasets are included). Primary datasets or computer code produced in the current study have to be deposited in appropriate public repositories prior to resubmission, and reviewer access details provided in case that public access is not yet allowed.

2) Each figure legend must specify
- size of the scale bars that are mandatory for all micrograph panels
- the statistical test used to generate error bars and P-values
- the type error bars (e.g., S.E.M., S.D.)
- the number (n) and nature (biological or technical replicate) of independent experiments underlying each data point
- Figures may not include error bars for experiments with n<3; scatter plots showing individual data points should be used instead.

3) Revised manuscript text (including main tables, and figure legends for main and EV figures) has to be submitted as editable text file (e.g., .docx format). We encourage highlighting of changes (e.g., via text color) for the referees' reference.

4) Each main and each Expanded View (EV) figure should be uploaded as individual production-quality files (preferably in .eps, .tif, .jpg formats). For suggestions on figure preparation/layout, please refer to our Figure Preparation Guidelines:
https://media.springernature.com/original/springer-cms/rest/v1/content/27825798/data/v1

5) Point-by-point response letters should include the original referee comments in full together with your detailed responses to them (and to specific editor requests if applicable), and also be uploaded as editable (e.g., .docx) text files.

6) Please complete our Author Checklist, and make sure that information entered into the checklist is also reflected in the manuscript; the checklist will be available to readers as part of the Review Process File.

7) All authors listed as (co-)corresponding need to deposit, in their respective author profiles in our submission system, a unique ORCiD identifier linked to their name. Please see our Guide to Authors for detailed instructions.

8) Please note that supplementary information at EMBO Press has been superseded by the 'Expanded View' for inclusion of additional figures, tables, movies or datasets; with up to five EV Figures being typeset and directly accessible in the HTML version of the article.

9) To facilitate reproducibilty and cross-laboratory adoption of methodologies, please structure the Materials & Methods section as outlined in our guide to authors, including a completed Reagents and Tools Table.

10) Digital image enhancement is acceptable practice, as long as it accurately represents the original data and conforms to community standards. If a figure has been subjected to significant electronic manipulation, this must be clearly noted in the figure legend and/or the 'Materials and Methods' section. The editors reserve the right to request original versions of figures and the original images that were used to assemble the figure. Finally, we generally encourage uploading of numerical as well as gel/blot image source data.

At EMBO Press we ask authors to provide source data for the main manuscript figures. You will receive a separate email with

instructions for providing source data with your revised manuscript, including how to upload and organize the files.

In the interest of ensuring the conceptual advance provided by the work, we recommend submitting a revision within 3 months (23rd Nov 2026). Please discuss the revision progress ahead of this time with the editor if you require more time to complete the revisions. Use the link below to submit your revision:

Link Not Available

Referee #1:

The authors have done a very good job of responding to my criticisms. For further clarity, the authors should consider addressing the following points.

1. Throughout the manuscript, the authors state that MCM-10 and SLD-2 are "jointly required" for CMG activation. This language is ambiguous and would seem to imply that both factors are essential, which is not what the authors intend, which is that they are partially (if not completely) redundant, as stated on Lines 537. Please use "partially redundant" or something equivalent throughout.

2. In Figure 3H, the strong RPA signal seen in the absence of RNR is clearly CMG-dependent, but no evidence is presented that the uniform RPA signal seen in the presence of RNR (RNR+) reflects CMG-dependent unwinding. Therefore, it's not clear what is the purpose of showing the RPA staining in the different genotypes in the RNR+ setting, especially since there is no comparison with WT. This data should be re-interpreted or removed. The finding that mutations in MCM-10 and SLD-2 enhance CMG retention while inhibiting DNA synthesis and chromosome decondensation would be adequate to make the point that these factors promote CMG activation.

3. Lines 295-299 make reference to DNSN-1 charge reversal mutations' effect on CMG helicase loading in Figure EV4E-F, but that data is not shown in this figure.

4. Lines 301-305: "epistatic" is not generally used to describe biochemical data, and it's not clear what the authors mean by it here, especially since they do not point to specific lanes to accompany their statements. Furthermore, the "additive" effect of SLD-2 with the other factors was not visible in the primary figure, which caused confusion for this reviewer. It would be better if Figure S4, which documents this point and is a more physiological mimic of the dsDNA CMG binding mode upon which these factors act, were more prominently displayed, or at least discussed separately as the evidence for the claim of additivity. The fact that the unwinding assays in Figure 4 do not involve activation of CMG on dsDNA, as is the case during replication initiation, should be stated more clearly.

5. Line 474: use "head-to-head" since "converging" invokes termination.

6. The discussion took a long time to address the main points of the paper. A traditional summary paragraph at the start would be helpful.

Referee #2:

The authors addressed all the issues I previously raised in this revised version, and I have no further concerns.

Referee 1:

"***The authors have done a very good job of responding to my criticisms. For further clarity, the authors should consider addressing the following points.***"

"***1. Throughout the manuscript, the authors state that MCM-10 and SLD-2 are "jointly required" for CMG activation. This language is ambiguous and would seem to imply that both factors are essential, which is not what the authors intend, which is that they are partially (if not completely) redundant, as stated on Lines 537. Please use "partially redundant" or something equivalent throughout.***"

Done – changes shown in red.

"***2. In Figure 3H, the strong RPA signal seen in the absence of RNR is clearly CMG-dependent, but no evidence is presented that the uniform RPA signal seen in the presence of RNR (RNR+) reflects CMG-dependent unwinding. Therefore, it's not clear what is the purpose of showing the RPA staining in the different genotypes in the RNR+ setting, especially since there is no comparison with WT. This data should be re-interpreted or removed. The finding that mutations in MCM-10 and SLD-2 enhance CMG retention while inhibiting DNA synthesis and chromosome decondensation would be adequate to make the point that these factors promote CMG activation.***"

The 'uniform RPA signal' is simply the nuclear localisation of this protein. The corresponding section has been re-worded to make this clear (lines 251-255).

"***3. Lines 295-299 make reference to DNSN-1 charge reversal mutations' effect on CMG helicase loading in Figure EV4E-F, but that data is not shown in this figure.***"

Thanks for pointing this out – this sentence needed to refer to wild type DNSN-1 and we have adjusted the text accordingly on lines 300-302.

"***4. Lines 301-305: "epistatic" is not generally used to describe biochemical data, and it's not clear what the authors mean by it here, especially since they do not point to specific lanes to accompany their statements. Furthermore, the "additive" effect of SLD-2 with the other factors was not visible in the primary figure, which caused confusion for this reviewer. It would be better if Figure S4, which documents this point and is a more physiological mimic of the dsDNA CMG binding mode upon which these factors act, were more prominently displayed, or at least discussed separately as the evidence for the claim of additivity. The fact that the unwinding assays in Figure 4 do not involve activation of CMG on dsDNA, as is the case during replication initiation, should be stated more clearly.***"

We have rewritten this section (lines 305-317) and now discuss Appendix Figure S4 more prominently.

"***5. Line 474: use "head-to-head" since "converging" invokes termination.***"

We now do so – changes shown in red.

"***6. The discussion took a long time to address the main points of the paper. A traditional summary paragraph at the start would be helpful.***"

We have added a new paragraph at the start of the discussion.

Referee 2

"***The authors addressed all the issues I previously raised in this revised version, and I have no further concerns.***"

EMBO Press Author Checklist

Corresponding Author Name: KARIM LABIB	
Journal Submitted to: THE EMBO JOURNAL	
Manuscript Number: EMBOJ-2026-123821	

USEFUL LINKS FOR COMPLETING THIS FORM
The EMBO Journal - Author Guidelines
EMBO Reports - Author Guidelines
Molecular Systems Biology - Author Guidelines
EMBO Molecular Medicine - Author Guidelines

Reporting Checklist for Life Science Articles (updated January
This checklist is adapted from Materials Design Analysis Reporting (MDAR) Checklist for Authors. MDAR establishes a minimum set of requirements in transparent reporting in the life sciences (see Statement of Task: 10.31222/osf.io/9sm4x). Please follow the journal's guidelines in preparing your
Please note that a copy of this checklist will be published alongside your article.

Abridged guidelines for figures
1. Data
The data shown in figures should satisfy the following conditions:
- the data were obtained and processed according to the field's best practice and are presented to reflect the results of the experiments in an accurate and unbiased manner.
- ideally, figure panels should include only measurements that are directly comparable to each other and obtained with the same assay.
- plots include clearly labeled error bars for independent experiments and sample sizes. Unless justified, error bars should not be shown for technical
- if n<5, the individual data points from each experiment should be plotted. Any statistical test employed should be justified.
- Source Data should be included to report the data underlying figures according to the guidelines set out in the authorship guidelines on Data

2. Captions
Each figure caption should contain the following information, for each panel where they are relevant:
- a specification of the experimental system investigated (eg cell line, species name).
- the assay(s) and method(s) used to carry out the reported observations and measurements.
- an explicit mention of the biological and chemical entity(ies) that are being measured.
- an explicit mention of the biological and chemical entity(ies) that are altered/varied/perturbed in a controlled manner.
- the exact sample size (n) for each experimental group/condition, given as a number, not a range;
- a description of the sample collection allowing the reader to understand whether the samples represent technical or biological replicates (including how many animals, litters, cultures, etc.).
- a statement of how many times the experiment shown was independently replicated in the laboratory.
- definitions of statistical methods and measures:
  - common tests, such as t-test (please specify whether paired vs. unpaired), simple χ2 tests, Wilcoxon and Mann-Whitney tests, can be unambiguously identified by name only, but more complex techniques should be described in the methods section;
  - are tests one-sided or two-sided?
  - are there adjustments for multiple comparisons?
  - exact statistical test results, e.g., P values = x but not P values < x;
  - definition of 'center values' as median or average;
  - definition of error bars as s.d. or s.e.m.

Please complete ALL of the questions below.
Select "Not Applicable" only when the requested information is not relevant for your study.

Materials

Newly Created Materials	Information included in the manuscript?	In which section is the information available? (Reagents and Tools Table, Materials and Methods, Figures, Data Availability Section)
New materials and reagents need to be available; do any restrictions apply?	Not Applicable	METHODS, ACKNOWLEDGEMENTS

Antibodies	Information included in the manuscript?	In which section is the information available? (Reagents and Tools Table, Materials and Methods, Figures, Data Availability Section)
For **antibodies** provide the following information:<br>- Commercial antibodies: RRID (if possible) or supplier name, catalogue number and or/clone number<br>- Non-commercial: RRID or citation	Yes	Reagents and Tools Table

DNA and RNA sequences	Information included in the manuscript?	In which section is the information available? (Reagents and Tools Table, Materials and Methods, Figures, Data Availability Section)
**Short novel DNA or RNA including primers, probes**: provide the sequences.	Yes	Reagents and Tools Table

Cell materials	Information included in the manuscript?	In which section is the information available? (Reagents and Tools Table, Materials and Methods, Figures, Data Availability Section)
**Cell lines:** Provide species information, strain. Provide accession number in repository **OR** supplier name, catalog number, clone number, and/**OR** RRID.	Yes	Reagents and Tools Table
**Primary cultures:** Provide species, strain, sex of origin, genetic modification status.	Not Applicable	
Report if the cell lines were recently **authenticated** (e.g., by STR profiling) and tested for mycoplasma contamination.	Not Applicable	

Experimental animals	Information included in the manuscript?	In which section is the information available? (Reagents and Tools Table, Materials and Methods, Figures, Data Availability Section)
**Laboratory animals or Model organisms:** Provide species, strain, sex, age, genetic modification status. Provide accession number in repository **OR** supplier name, catalog number, clone number, **OR** RRID.	Not Applicable	Reagents and Tools Table
**Animal observed in or captured from the field:** Provide species, sex, and age where possible.	Not Applicable	
Please detail **housing and husbandry conditions**.	Not Applicable	

Plants and microbes	Information included in the manuscript?	In which section is the information available? (Reagents and Tools Table, Materials and Methods, Figures, Data Availability Section)
**Plants:** provide species and strain, ecotype and cultivar where relevant, unique accession number if available, and source (including location for collected wild specimens).	Not Applicable	
**Microbes:** provide species and strain, unique accession number if available, and source.	Yes	Reagents and Tools Table

Human research participants	Information included in the manuscript?	In which section is the information available? (Reagents and Tools Table, Materials and Methods, Figures, Data Availability Section)
If collected and within the bounds of privacy constraints report on age, sex and gender or ethnicity for all study participants.	Not Applicable	

Core facilities	Information included in the manuscript?	In which section is the information available? (Reagents and Tools Table, Materials and Methods, Figures, Data Availability Section)
If your work benefited from core facilities, was their service mentioned in the acknowledgments section?	Yes	Reagents and Tools Table

Design

Study protocol	Information included in the manuscript?	In which section is the information available? (Reagents and Tools Table, Materials and Methods, Figures, Data Availability Section)
If study protocol has been **pre-registered, provide DOI in the manuscript**. For clinical trials, provide the trial registration number **OR** cite DOI.	Not Applicable	
Report the **clinical trial registration number** (at ClinicalTrials.gov or equivalent), where applicable.	Not Applicable	

Laboratory protocol	Information included in the manuscript?	In which section is the information available? (Reagents and Tools Table, Materials and Methods, Figures, Data Availability Section)
Provide DOI OR other citation details if **external detailed step-by-step protocols** are available.	Not Applicable	

Experimental study design and statistics	Information included in the manuscript?	In which section is the information available? (Reagents and Tools Table, Materials and Methods, Figures, Data Availability Section)
Include a statement about **sample size** estimate even if no statistical methods were used.	Yes	MATERIALS AND METHODS, FIGURE LEGENDS
Were any steps taken to minimize the effects of subjective bias when allocating animals/samples to treatment (e.g. **randomization procedure**)? If yes, have they been described?	Not Applicable	
Include a statement about **blinding** even if no blinding was done.	Not Applicable	
Describe **inclusion/exclusion criteria** if samples or animals were excluded from the analysis. Were the criteria pre-established?<br><br>If sample or data points were omitted from analysis, report if this was due to attrition or intentional exclusion and provide justification.	Not Applicable	
For every figure, are **statistical tests** justified as appropriate? Do the data meet the assumptions of the tests (e.g., normal distribution)? Describe any methods used to assess it. Is there an estimate of variation within each group of data? Is the variance similar between the groups that are being statistically compared?	Yes	MATERIALS AND METHODS, FIGURE LEGENDS

Sample definition and in-laboratory replication	Information included in the manuscript?	In which section is the information available? (Reagents and Tools Table, Materials and Methods, Figures, Data Availability Section)
In the figure legends: state number of times the experiment was **replicated** in laboratory.	Yes	FIGURE LEGENDS
In the figure legends: define whether data describe **technical or biological replicates**.	Not Applicable	

Ethics

Ethics	Information included in the manuscript?	In which section is the information available? (Reagents and Tools Table, Materials and Methods, Figures, Data Availability Section)
Studies involving **human participants**: State details of **authority granting ethics approval** (IRB or equivalent committee(s), provide reference number for approval.	Not Applicable	
Studies involving **human participants**: Include a statement confirming that **informed consent** was obtained from all subjects and that the experiments conformed to the principles set out in the WMA Declaration of Helsinki and the Department of Health and Human Services Belmont Report.	Not Applicable	
Studies involving **human participants:** For publication of **patient photos**, include a statement confirming that consent to publish was obtained.	Not Applicable	
Studies involving experimental **animals**: State details of **authority granting ethics approval** (IRB or equivalent committee(s), provide reference number for approval. Include a statement of compliance with ethical regulations.	Not Applicable	
Studies involving **specimen and field samples:** State if relevant **permits** obtained, provide details of authority approving study; if none were required, explain why.	Not Applicable	

Dual Use Research of Concern (DURC)	Information included in the manuscript?	In which section is the information available? (Reagents and Tools Table, Materials and Methods, Figures, Data Availability Section)
Could your study fall under dual use research restrictions? Please check biosecurity documents and list of **select agents and toxins** (CDC): https://www.selectagents.gov/sat/list.htm	Not Applicable	
If you used a select agent, is the security level of the lab appropriate and reported in the manuscript?	Not Applicable	
If a study is subject to dual use research of concern regulations, is the name of the **authority granting approval and reference number** for the regulatory approval provided in the manuscript?	Not Applicable	

Reporting

The MDAR framework recommends adoption of discipline-specific guidelines, established and endorsed through community initiatives. Journals have their own policy about requiring specific guidelines and recommendations to complement MDAR.

Adherence to community standards	Information included in the manuscript?	In which section is the information available? (Reagents and Tools Table, Materials and Methods, Figures, Data Availability Section)
State if relevant guidelines or checklists (e.g., **ICMJE, MIBBI, ARRIVE, PRISMA**) have been followed or provided.	Not Applicable	
For **tumor marker prognostic studies,** we recommend that you follow the **REMARK** reporting guidelines (see link list at top right). See author guidelines, under 'Reporting Guidelines'. Please confirm you have followed these guidelines.	Not Applicable	
For **phase II and III randomized controlled trials**, please refer to the **CONSORT** flow diagram (see link list at top right) and submit the CONSORT checklist (see link list at top right) with your submission. See author guidelines, under 'Reporting Guidelines'. Please confirm you have submitted this list.	Not Applicable	

Data Availability

Data availability	Information included in the manuscript?	In which section is the information available? (Reagents and Tools Table, Materials and Methods, Figures, Data Availability Section)
Have **primary datasets** been deposited according to the journal's guidelines (see 'Data Deposition' section) and the respective accession numbers provided in the Data Availability Section?	Not Applicable	
Were **human clinical and genomic datasets** deposited in a public access-controlled repository in accordance to ethical obligations to the patients and to the applicable consent agreement?	Not Applicable	
Are **computational models** that are central and integral to a study available without restrictions in a machine-readable form? Were the relevant accession numbers or links provided?	Not Applicable	
If publicly available data were reused, provide the respective **data citations in the reference list**.	Not Applicable	
