# Supplementary material for: MCM10 and SLD-2/RECQL4 jointly activate the CMG helicase during metazoan DNA replication initiation
Source: EMBO J. 2026 Sep 28;45(21):7582–620. doi: 10.1038/s44318-026-00915-9 (PMC13728148; doi:10.1038/s44318-026-00915-9)
Supplement: Supplementary file 3 [file 44318_2026_915_MOESM57_ESM.pdf]

# Expanded View Figures

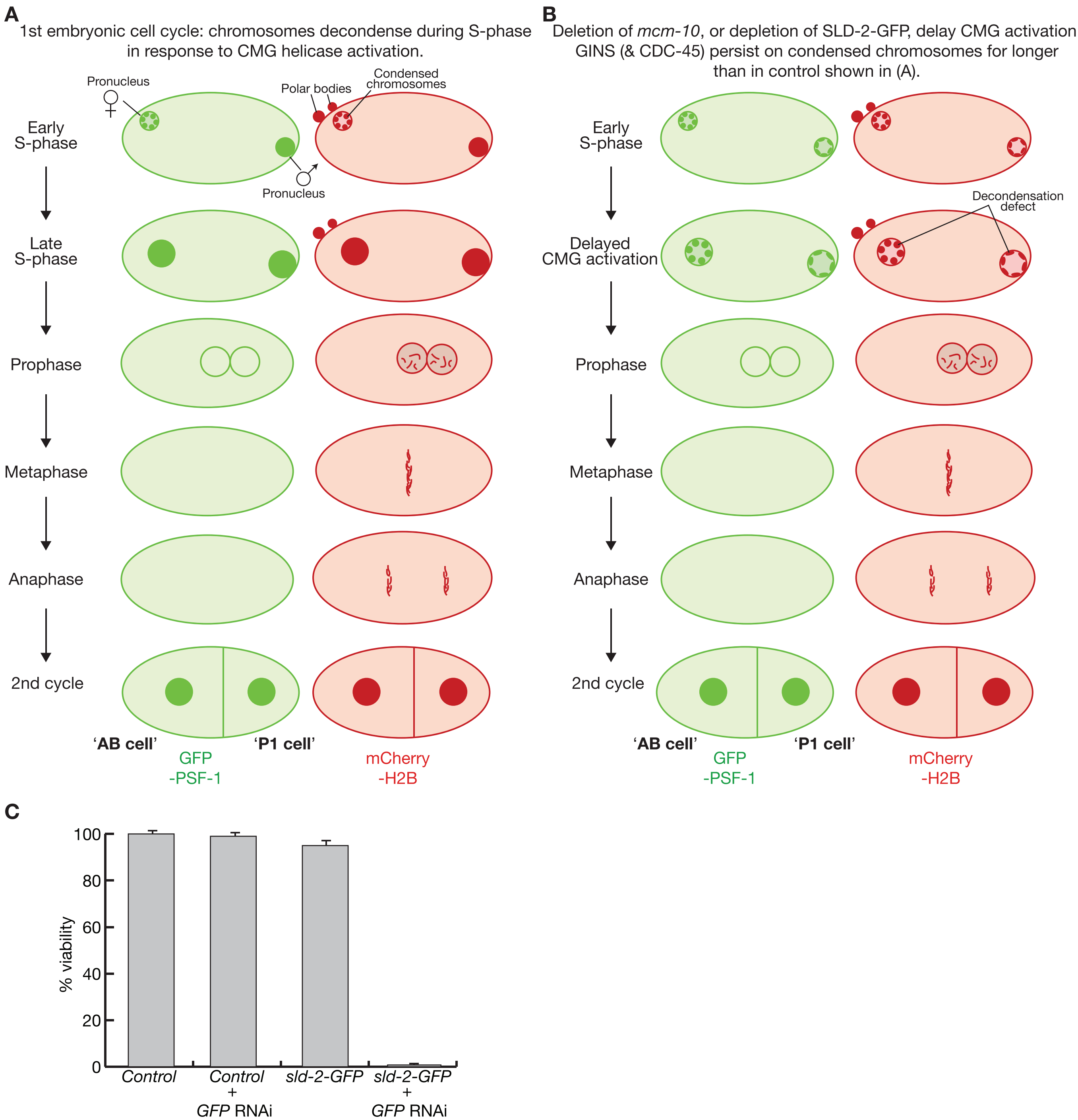


**Figure EV1. Depletion of SLD-2 is lethal in *C. elegans*.**

**(A)** Illustration of the first embryonic cell cycle of *C. elegans* expressing GFP-PSF-1 and mCherry-HistoneH2B. **(B)** Illustration of how chromosome decondensation is delayed in response to defects in CMG helicase activation in the first embryonic cell cycle of *mcm-10Δ* worms, or in response to depletion of SLD-2. **(C)** Embryonic viability was measured as described in Methods, after treating control (wild type 'N2' worms) or *sld-2-GFP* worms with *GFP* RNAi as indicated. Mean values and standard deviations are presented from three independent experiments.

© The Author(s)

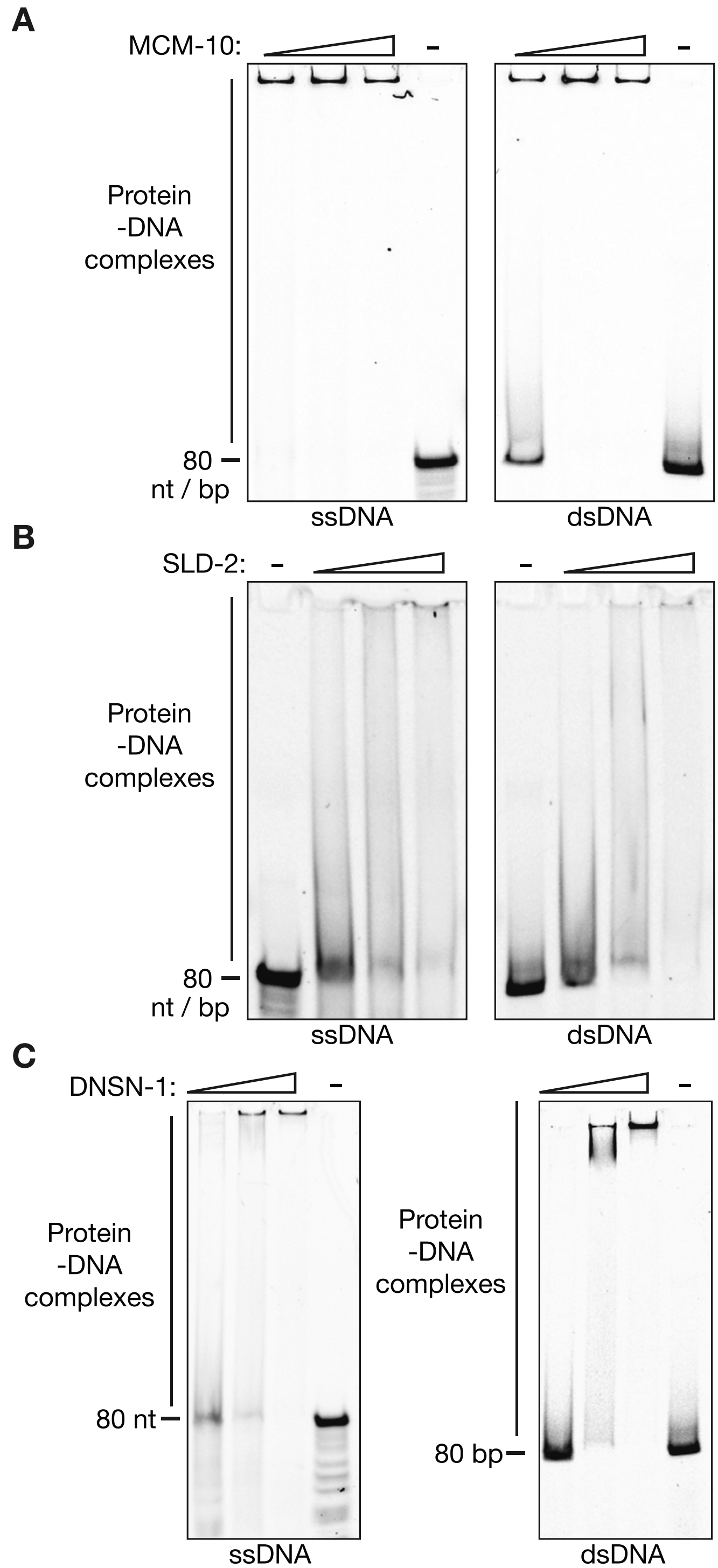


**Figure EV2. MCM-10, SLD-2 and DNSN-1 all bind to DNA in vitro.**

As described in Methods, the indicated concentrations of MCM-10 (**A**), SLD-2 (**B**) and DNSN-1 (**C**) were incubated with 5 nM Cy5-labelled DNA at 25 °C for 15 min. Protein–DNA complexes were then analyzed in native polyacrylamide gradient gels before detection of the fluorescently labelled DNA.

 © The Author(s)

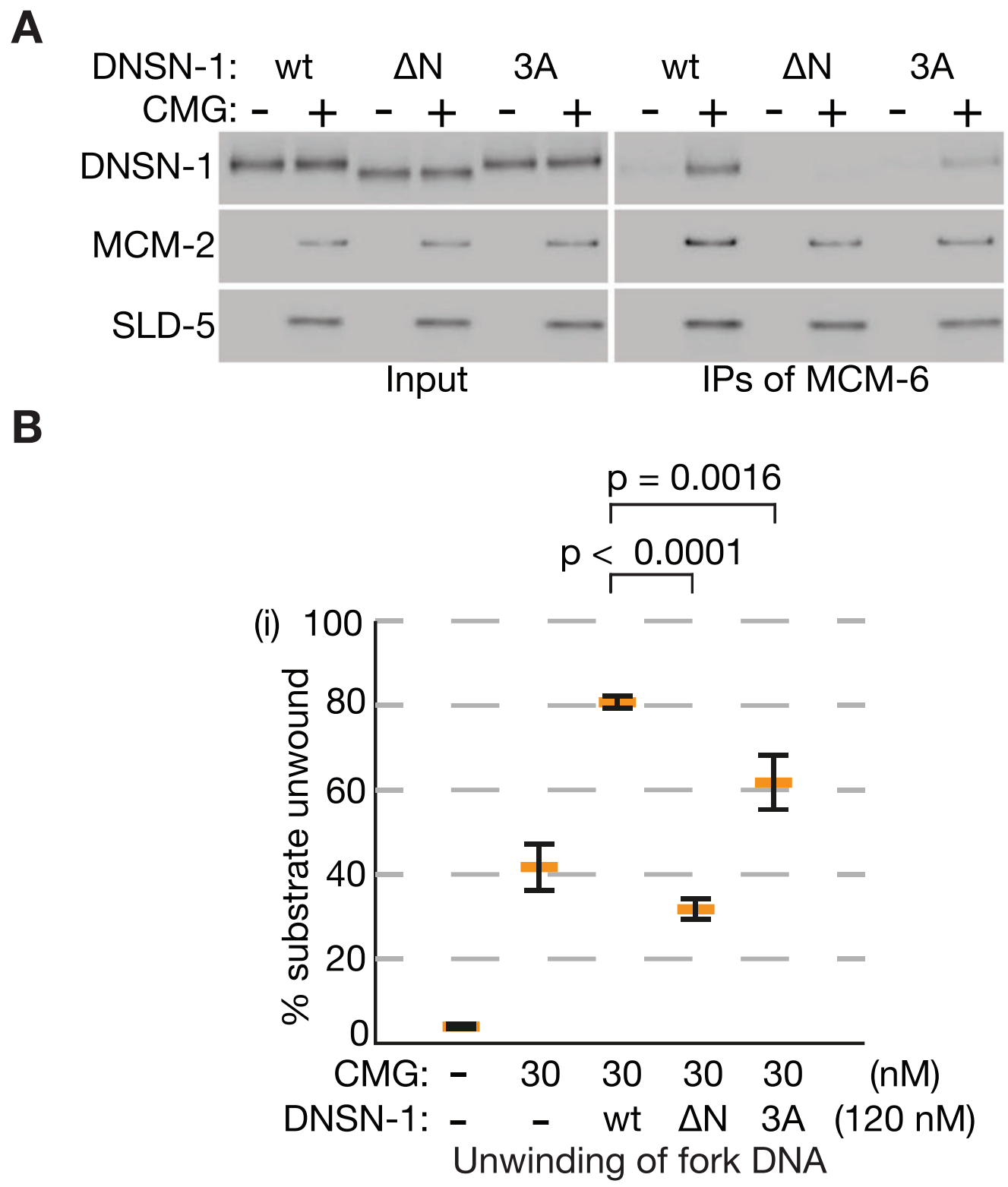


**Figure EV3. The ability of DNSN-1 to bind CMG is important for the stimulation of helicase activity in vitro.**

(**A**) Recombinant *C. elegans* CMG was mixed as indicated with wild-type DNSN-1 (wt), DNSN-1 lacking the first 20 amino acids (DNSN-1-ΔN), or DNSN-1 with mutations in three conserved residues that contact the MCM-3 subunit of the CMG helicase (DNSN-1-3A). As described above for Fig. 2C, F, the CMG helicase was then isolated by immunoprecipitation of the MCM-6 subunit, and the indicated proteins were detected by immunoblotting. (**B**) The ability of DNSN-1, DNSN-1-ΔN and DNSN-1-3A to stimulate CMG helicase activity was monitored in vitro as for Fig. 4D, G. The data correspond to three independent experiments, with mean values and corresponding standard deviations. *P* values were generated via a one-way ANOVA followed by Dunnett's multiple comparisons test.

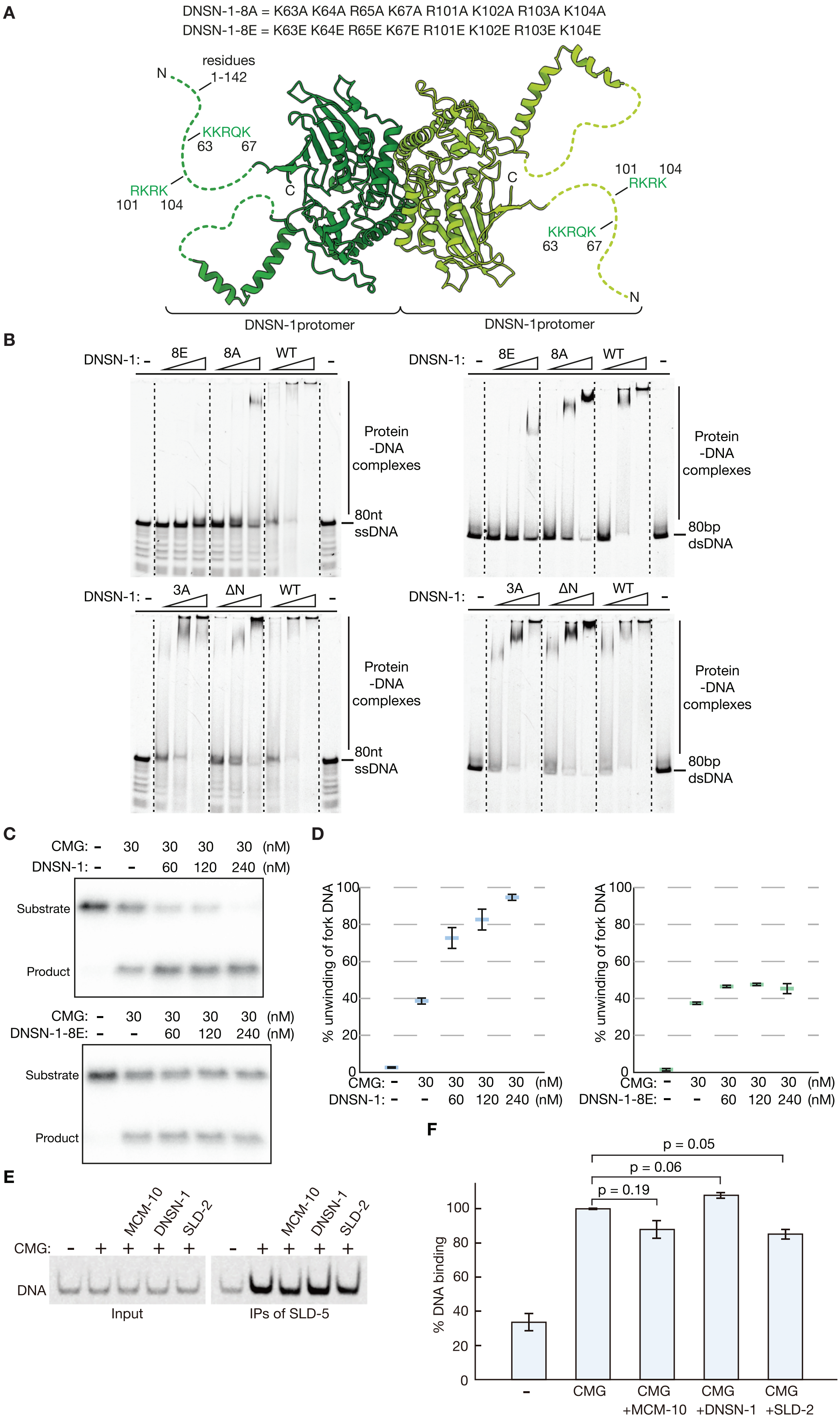
A
DNSN-1-8A = K63A K64A R65A K67A R101A K102A R103A K104A
DNSN-1-8E = K63E K64E R65E K67E R101E K102E R103E K104E
residues 1-142
N
KKRQK
63 67
RKRK
101 104
C
C
101 104
RKRK
KKRQK
63 67
N
DNSN-1protomer
DNSN-1protomer
B
DNSN-1: – 8E 8A WT –
Protein -DNA complexes
80nt ssDNA
DNSN-1: – 8E 8A WT –
Protein -DNA complexes
80bp dsDNA
DNSN-1: – 3A ΔN WT –
Protein -DNA complexes
80nt ssDNA
DNSN-1: – 3A ΔN WT –
Protein -DNA complexes
80bp dsDNA
C
CMG: – 30 30 30 30 (nM)
DNSN-1: – – 60 120 240 (nM)
Substrate
Product
CMG: – 30 30 30 30 (nM)
DNSN-1-8E: – – 60 120 240 (nM)
Substrate
Product
D
% unwinding of fork DNA
0 20 40 60 80 100
CMG: – 30 30 30 30 (nM)
DNSN-1: – – 60 120 240 (nM)
% unwinding of fork DNA
0 20 40 60 80 100
CMG: – 30 30 30 30 (nM)
DNSN-1-8E: – – 60 120 240 (nM)
E
MCM-10 DNSN-1 SLD-2
MCM-10 DNSN-1 SLD-2
CMG: – + + + + – + + + +
DNA
Input
IPs of SLD-5
F
p = 0.05
p = 0.06
p = 0.19
% DNA binding
0 20 40 60 80 100
– CMG CMG +MCM-10 CMG +DNSN-1 CMG +SLD-2

 © The Author(s)

**Figure EV4. DNA binding by DNSN-1 is important for stimulation of CMG helicase activity.**

(**A**) AlphaFold prediction of the DNSN-1 dimer, indicating the location within the amino-terminal disordered region of mutations corresponding to the DNSN-1-8A and DNSN-1-8E alleles. (**B**) DNA binding activity of the indicated forms of DNSN-1 were assayed as in Fig. EV2. DNSN-1-ΔN = deletion of residues 5–19, whereas DNSN-1-3A = W425A I429A V431A (Xia et al, 2023). (**C**) CMG helicase assays were performed as in Fig. 4, to compare stimulation by the indicated concentrations of DNSN-1 and DNSN-1-8E. (**D**) Quantification of the data from (**D**). The mean values from three independent experiments are shown together with corresponding standard deviations. (**E**) Loading of the CMG helicase onto a forked DNA substrate was assayed as described in Methods. The DNA substrate was labelled with Cy5 to facilitate detection. (**F**) Quantification of the data from (**E**). The mean values from three independent experiments are shown together with corresponding standard deviations. *P* values were generated via a one-way ANOVA followed by Dunnett's multiple comparisons test.

© The Author(s)

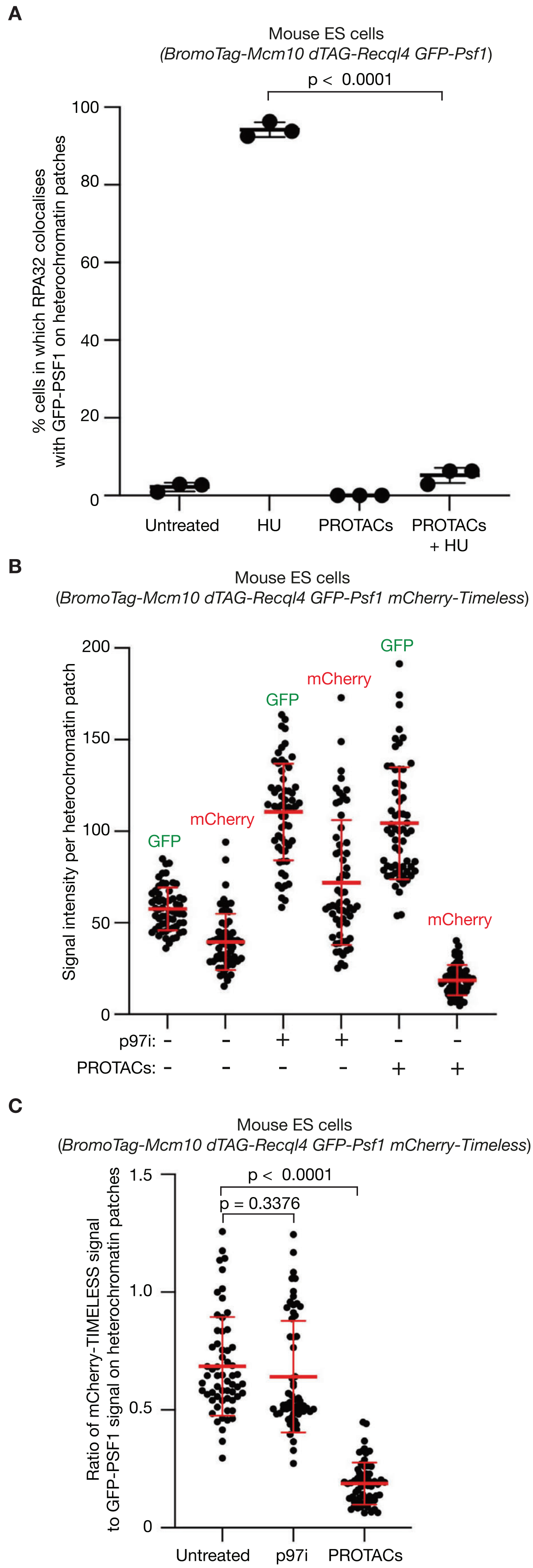
A
Mouse ES cells
(BromoTag-Mcm10 dTAG-Recql4 GFP-Psf1)
p < 0.0001
% cells in which RPA32 colocalises with GFP-PSF1 on heterochromatin patches
100
80
60
40
20
0
Untreated
HU
PROTACs
PROTACs + HU
B
Mouse ES cells
(BromoTag-Mcm10 dTAG-Recql4 GFP-Psf1 mCherry-Timeless)
Signal intensity per heterochromatin patch
200
150
100
50
0
GFP
mCherry
GFP
mCherry
GFP
mCherry
p97i: - - + + - -
PROTACs: - - - - + +
C
Mouse ES cells
(BromoTag-Mcm10 dTAG-Recql4 GFP-Psf1 mCherry-Timeless)
Ratio of mCherry-TIMELESS signal to GFP-PSF1 signal on heterochromatin patches
1.5
1.0
0.5
0
p < 0.0001
p = 0.3376
Untreated
p97i
PROTACs

 © The Author(s)

**Figure EV5. Quantification of recruitment of RPA, TIMELESS and CMG to heterochromatin patches in mouse ES cells.**

(**A**) Quantification of the data from Fig. 8E, showing the proportion of cells with GFP-PSF1 on heterochromatin patches that also showed colocalisation of RPA32 with GFP-PSF1 (see Methods). The data correspond to the mean values and corresponding standard deviations from three independent experiments. *P* values were generated via a one-way ANOVA followed by Dunnett's multiple comparisons test. (**B**) Quantification of data from the experiment in Fig. 8G (see Methods), showing the signal intensity for GFP-PSF1 and mCherry-TIMELESS on heterochromatin patches in cells treated as indicated. The data correspond to 20 patches in each of three independent experiments, with mean values shown as red bars, together with corresponding standard deviations. (**C**) The ratio of the mCherry-TIMELESS and GFP-PSF1 signals on individual heterochromatin patches was calculated for the data in (**B**). *P* values were generated via a one-way ANOVA followed by Dunnett's multiple comparisons test.

© The Author(s)
