# Supplementary material for: MCM10 and SLD-2/RECQL4 jointly activate the CMG helicase during metazoan DNA replication initiation
Source: EMBO J. 2026 Sep 28;45(21):7582–620. doi: 10.1038/s44318-026-00915-9 (PMC13728148; doi:10.1038/s44318-026-00915-9)

Figure 1A - control - 0’
This image is rotated by 180° in Figure 1A and by 270° in Appendix Movie S1.
The 0’ timepoint Figure 1A corresponds to frame 15 (timestamp 2’10”) of Appendix Movie S1.

GFP-PSF-1 mCherry-H2B

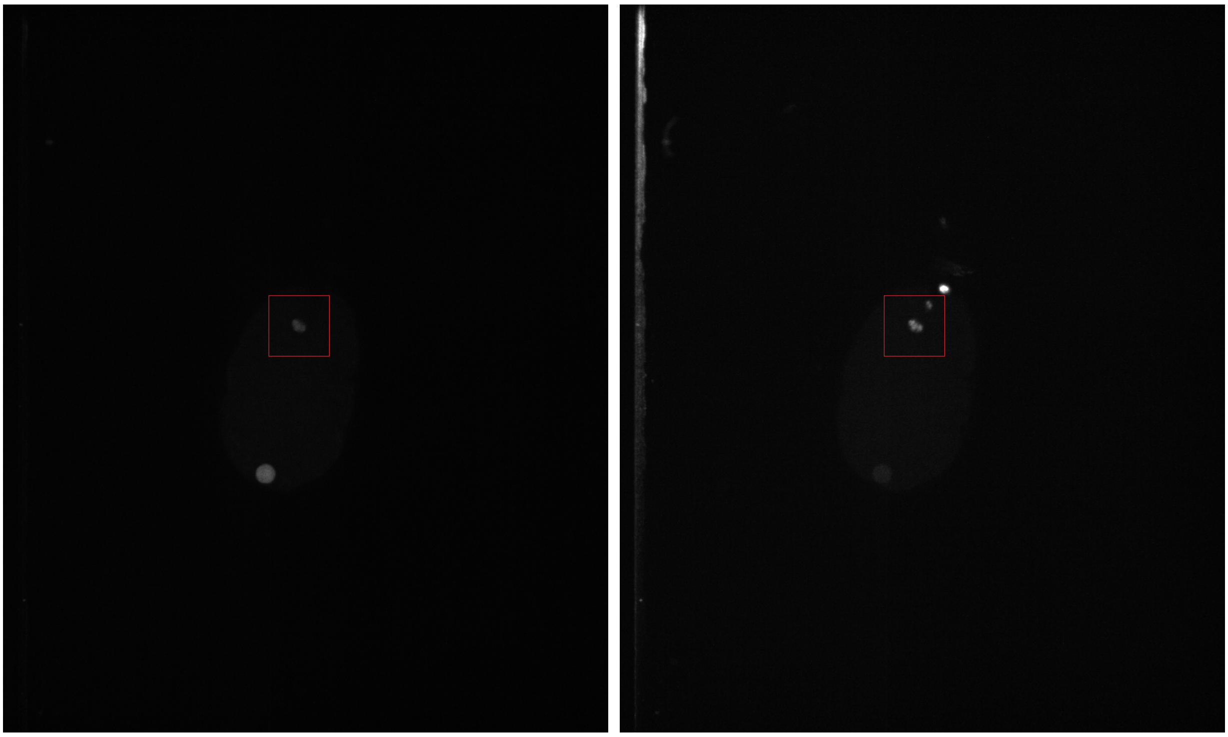

Figure 1A - *mcm10Δ* - 0’
This image is rotated by 270° in Appendix Movie S2.
The 0’ timepoint Figure 1A corresponds to frame 22 (timestamp 3’20”) of Appendix Movie S2.

GFP-PSF-1 mCherry-H2B

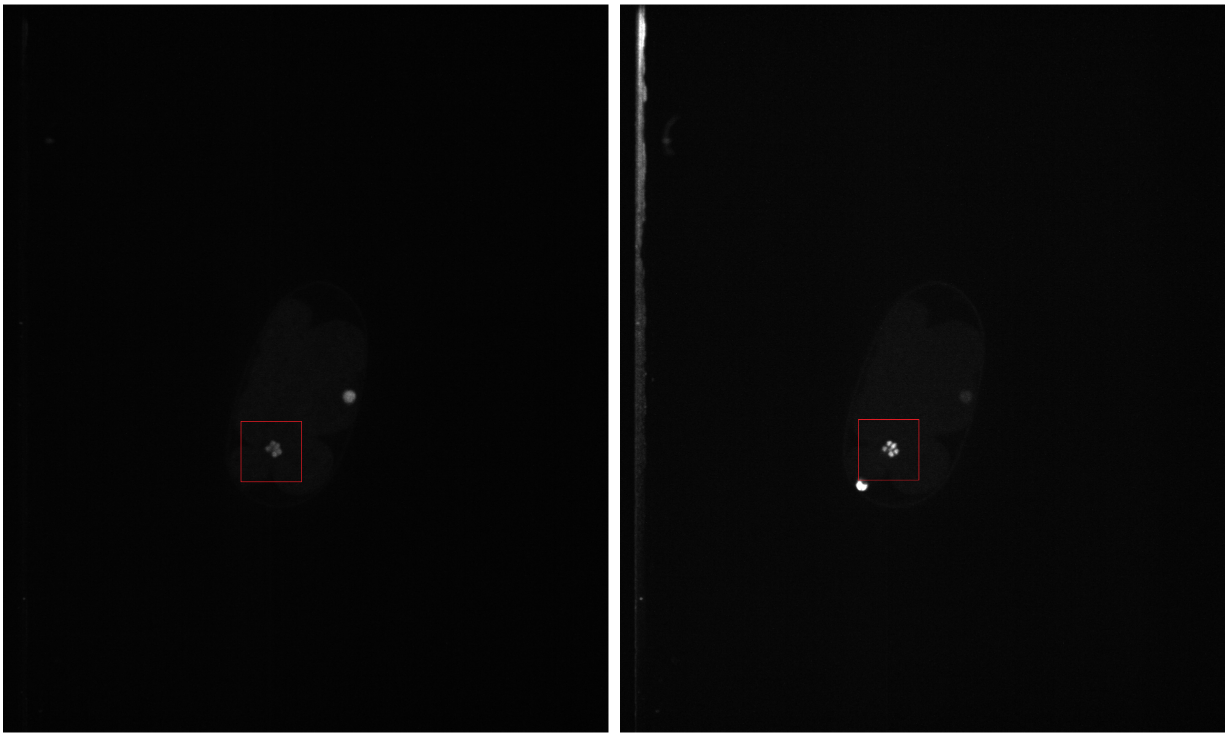

Supplement: Supplementary file 43 [file 44318_2026_915_MOESM48_ESM.zip › Figure 1_Source Data/1A/READ ME.pdf]
