# Supplementary material for: MCM10 and SLD-2/RECQL4 jointly activate the CMG helicase during metazoan DNA replication initiation
Source: EMBO J. 2026 Sep 28;45(21):7582–620. doi: 10.1038/s44318-026-00915-9 (PMC13728148; doi:10.1038/s44318-026-00915-9)

Figure 1B - *Control*
This image is rotated by 90° in Figure 1B

EdU

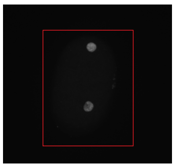

EdU
Contrast adjusted

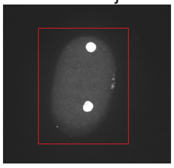

Figure 1B - *mcm-10Δ*
This image is rotated by 270° in Figure 1B

EdU

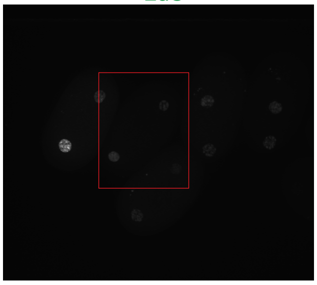

EdU
Contrast adjusted

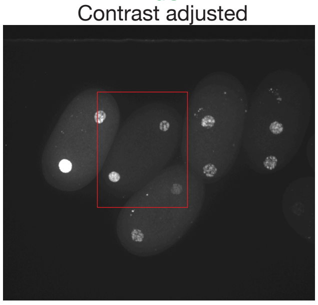

Figure 1B - *sld-2-GFP* + *GFP* RNAi

EdU

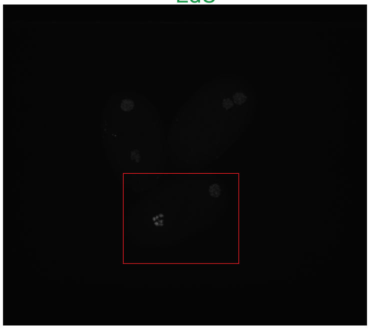

EdU
Contrast adjusted

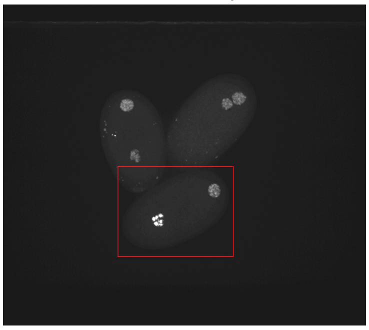

Figure 1B - *mcm-10Δ* + *sld-2-GFP* + *GFP* RNAi

EdU

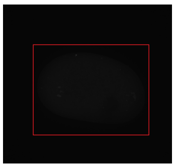

EdU
Contrast adjusted

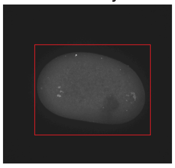

Supplement: Supplementary file 43 [file 44318_2026_915_MOESM48_ESM.zip › Figure 1_Source Data/1B/READ ME.pdf]
