# Supplementary material for: MCM10 and SLD-2/RECQL4 jointly activate the CMG helicase during metazoan DNA replication initiation
Source: EMBO J. 2026 Sep 28;45(21):7582–620. doi: 10.1038/s44318-026-00915-9 (PMC13728148; doi:10.1038/s44318-026-00915-9)

Figure 1E - control - 0’
The 0’ timepoint Figure 1E corresponds to frame 7 (timestamp 1’00”) of Appendix Movie S5.

SLD-2-GFP mCherry-PSF-1

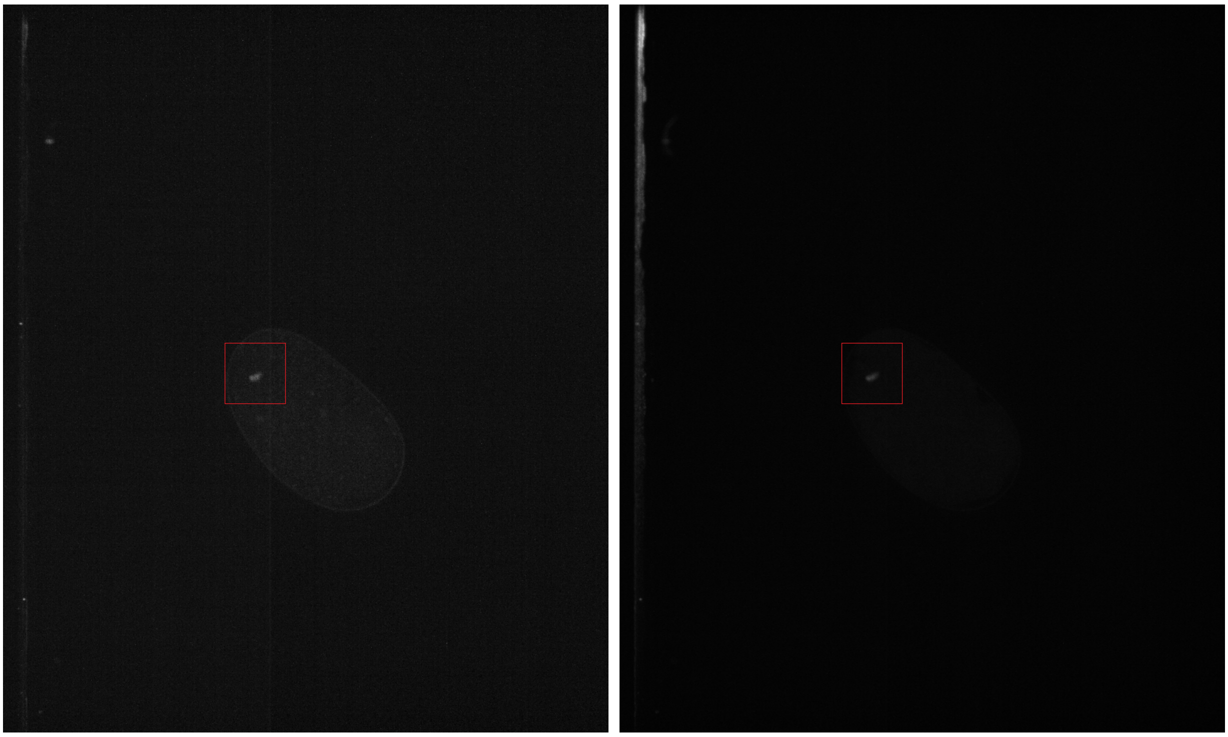

Figure 1E - *GFP* RNAi - 0’
The 0’ timepoint Figure 1E corresponds to frame 7 (timestamp 1’00”) of Appendix Movie S6.

SLD-2-GFP mCherry-PSF-1

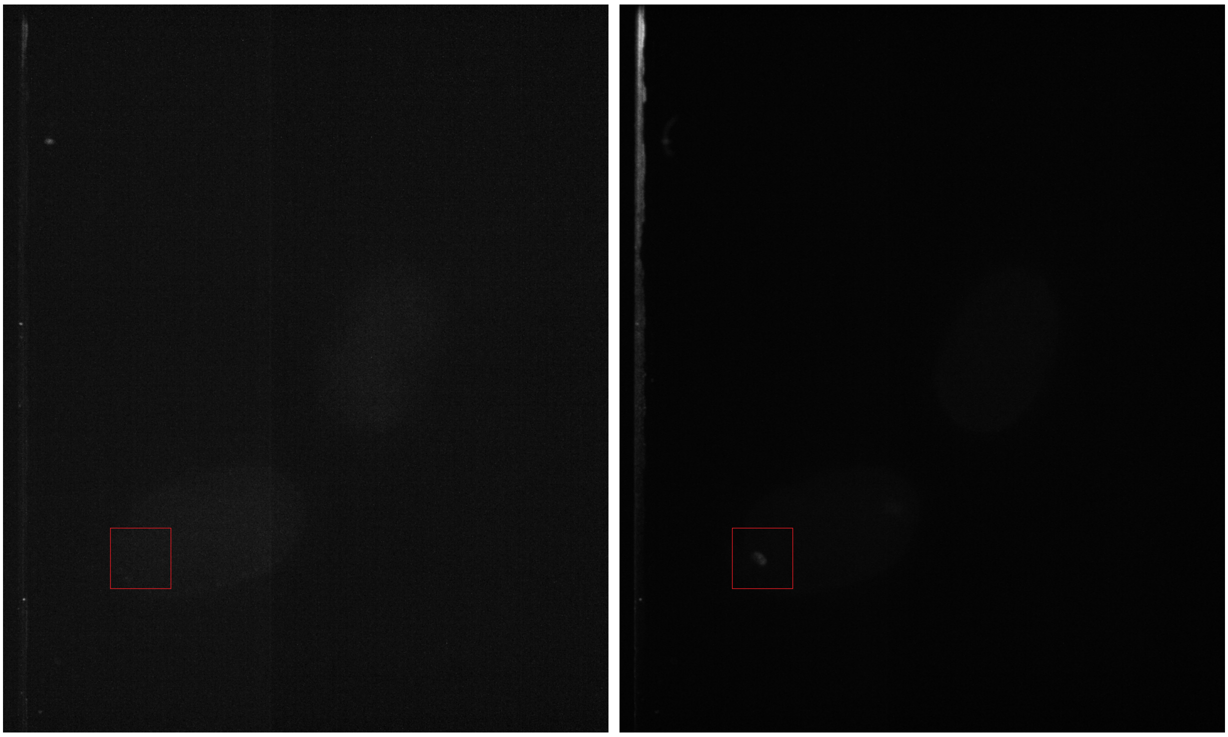

Supplement: Supplementary file 43 [file 44318_2026_915_MOESM48_ESM.zip › Figure 1_Source Data/1E/READ ME.pdf]
