# Supplementary material for: MCM10 and SLD-2/RECQL4 jointly activate the CMG helicase during metazoan DNA replication initiation
Source: EMBO J. 2026 Sep 28;45(21):7582–620. doi: 10.1038/s44318-026-00915-9 (PMC13728148; doi:10.1038/s44318-026-00915-9)

Figure 1D - control - 0’
This image is rotated by 180° in Figure 1D and by 270° in Appendix Movie S3.
The 0’ timepoint Figure 1D corresponds to frame 5 (timestamp 0’40”) of Appendix Movie S3.

SLD-2-GFP mCherry-H2B

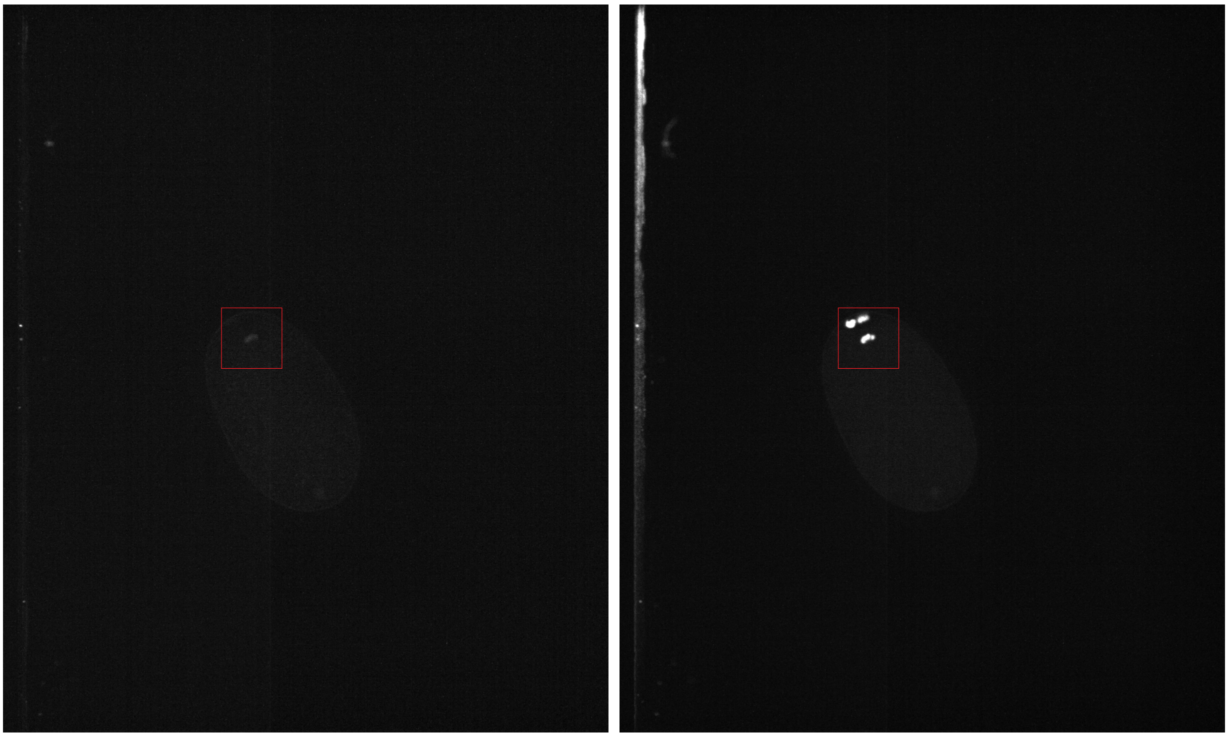

Figure 1D - *GFP* RNAi - 0’
This image is rotated by 90° in Appendix Movie S4.
The 0’ timepoint Figure 1D corresponds to frame 10 (timestamp 1’30”) of Appendix Movie S4.

SLD-2-GFP mCherry-H2B

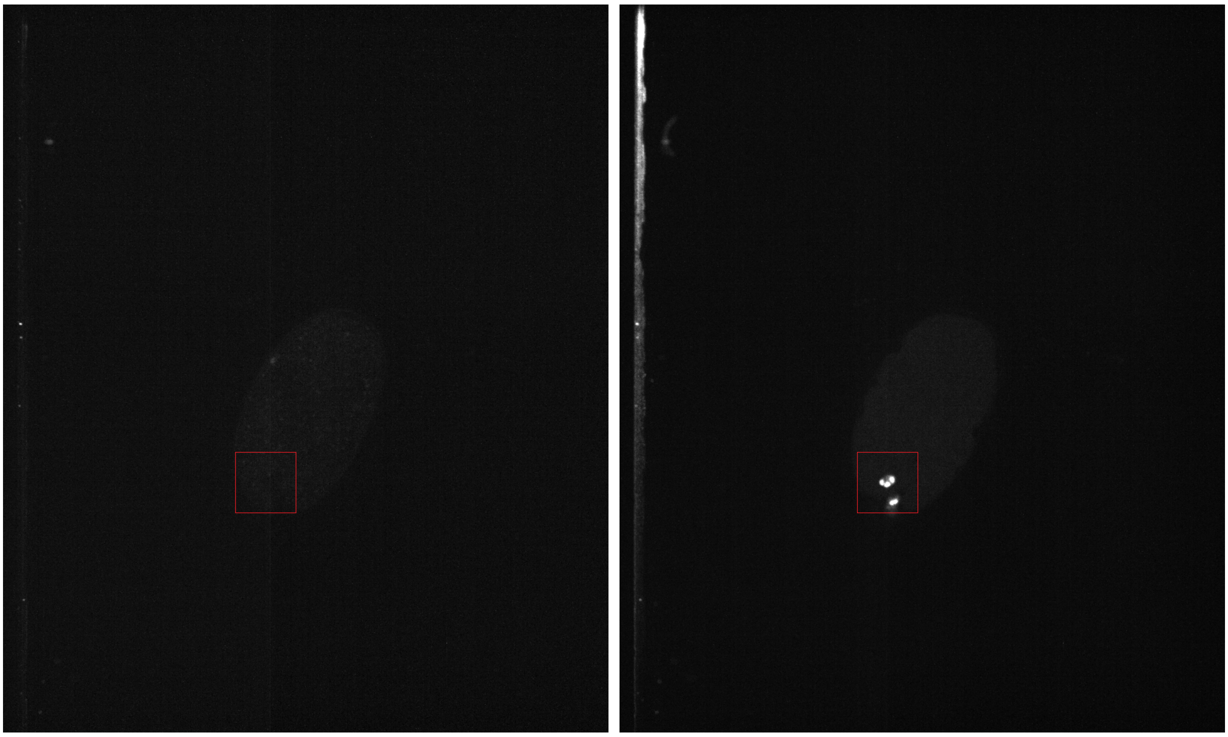

Supplement: Supplementary file 43 [file 44318_2026_915_MOESM48_ESM.zip › Figure 1_Source Data/1D/READ ME.pdf]
