# Supplementary material for: MCM10 and SLD-2/RECQL4 jointly activate the CMG helicase during metazoan DNA replication initiation
Source: EMBO J. 2026 Sep 28;45(21):7582–620. doi: 10.1038/s44318-026-00915-9 (PMC13728148; doi:10.1038/s44318-026-00915-9)

Figure 2A - *Control* - 0'
This image is rotated by 270° in Figure 2A and in Appendix Movie S7.
The 0' timepoint Figure 2A corresponds to frame 15 (timestamp 2'30") of Appendix Movie S7.

GFP-MCM-10

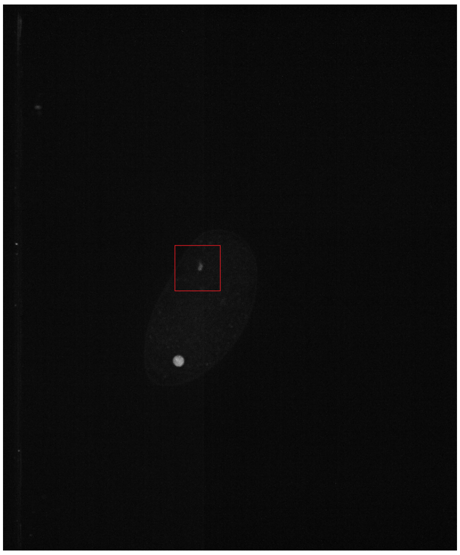

mCherry-HIS

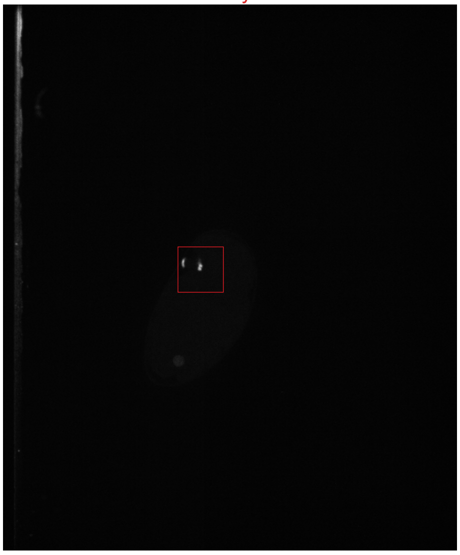

Figure 2A - *cdc-45* RNAi - 0'
This image is rotated by 90° in Appendix Movie Sxx.
The 0' timepoint Figure 2A corresponds to frame 25 (timestamp 3'50") of Appendix Movie S8.

GFP-MCM-10

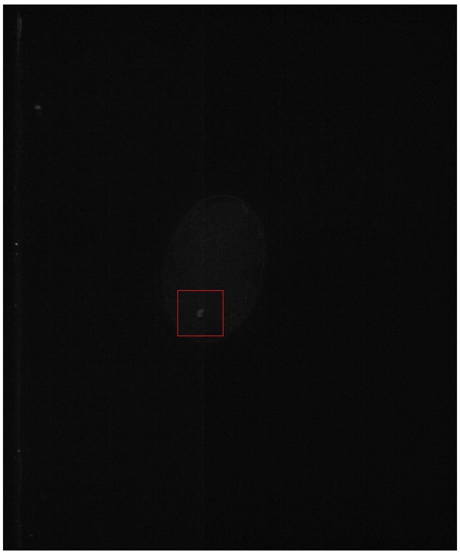

mCherry-HIS

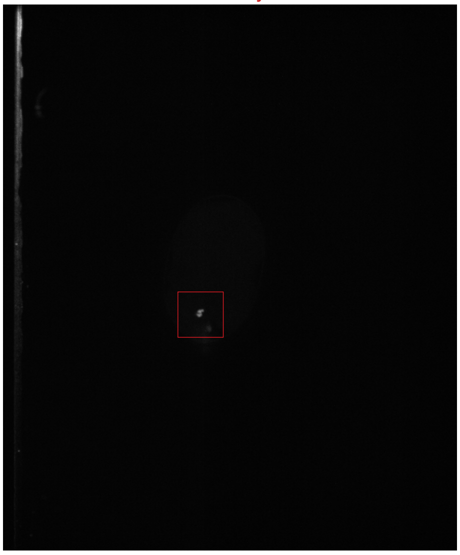

Figure 2A - *sld-2* RNAi - 0'
This image is rotated by 270° in Appendix Movie S9.
The 0' timepoint Figure 2A corresponds to frame 8 (timestamp 1'40") of Appendix Movie S9.

GFP-MCM-10

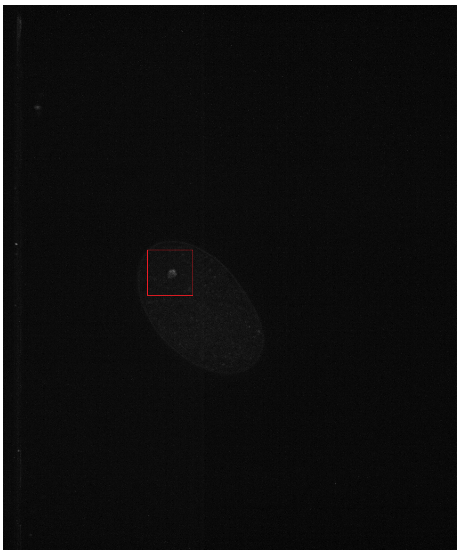

mCherry-HIS

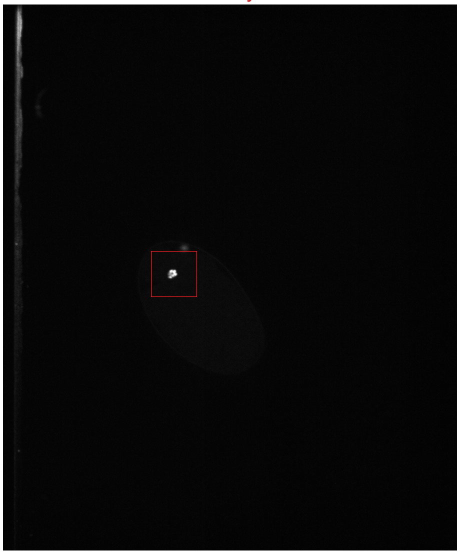

Supplement: Supplementary file 44 [file 44318_2026_915_MOESM49_ESM.zip › Figure 2_Source Data/2A/READ ME.pdf]
