# Supplementary material for: MCM10 and SLD-2/RECQL4 jointly activate the CMG helicase during metazoan DNA replication initiation
Source: EMBO J. 2026 Sep 28;45(21):7582–620. doi: 10.1038/s44318-026-00915-9 (PMC13728148; doi:10.1038/s44318-026-00915-9)
Supplement: Supplementary file 44 [file 44318_2026_915_MOESM49_ESM.zip › Figure 2_Source Data/2C/Figure 2C - Blot_anti MCM-10.pdf]

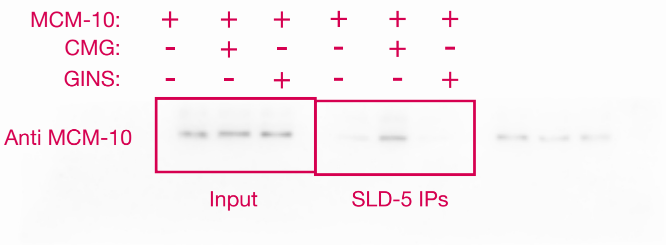
MCM-10: + + + + + +
CMG: - + - - + -
GINS: - - + - - +
Anti MCM-10
Input
SLD-5 IPs
