# Supplementary material for: MCM10 and SLD-2/RECQL4 jointly activate the CMG helicase during metazoan DNA replication initiation
Source: EMBO J. 2026 Sep 28;45(21):7582–620. doi: 10.1038/s44318-026-00915-9 (PMC13728148; doi:10.1038/s44318-026-00915-9)

Figure 2D - *Control* - 0’
This image is rotated by 180° in Figure 2D and in Appendix Movie S11.
The 0’ timepoint Figure 2D corresponds to frame 13 (timestamp 1’50”) of Appendix Movie S11.

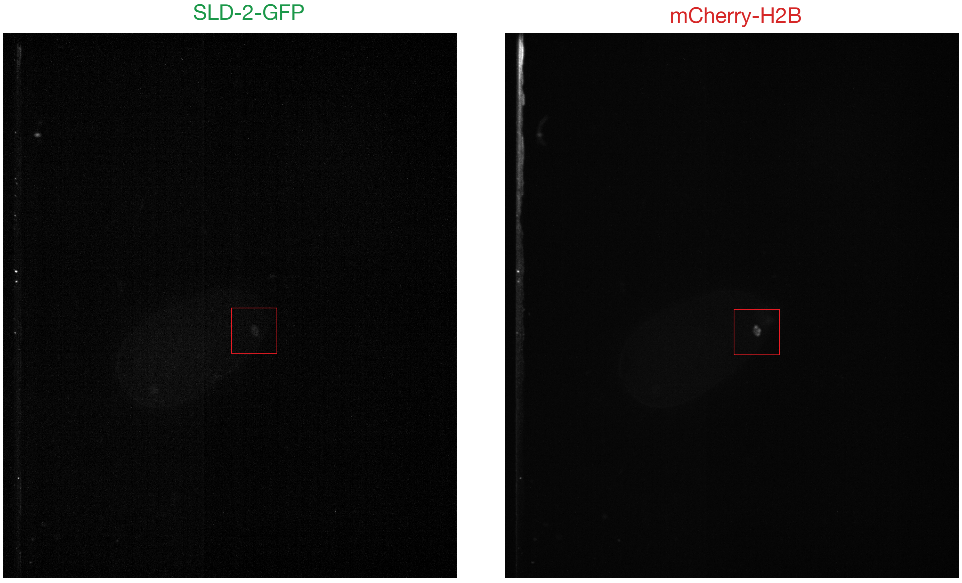

Figure 2D - *cdc-45* RNAi - 0’
This image is rotated by 270° in Figure 2D.
The 0’ timepoint Figure 2D corresponds to frame 10 (timestamp 1’30”) of Appendix Movie S12.

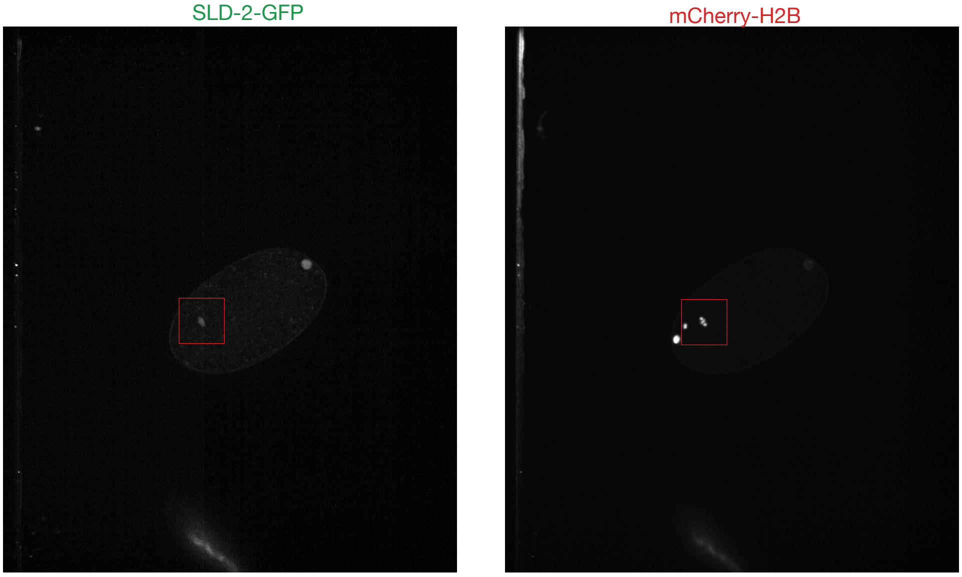

Supplement: Supplementary file 44 [file 44318_2026_915_MOESM49_ESM.zip › Figure 2_Source Data/2D/READ ME.pdf]
