# Supplementary material for: MCM10 and SLD-2/RECQL4 jointly activate the CMG helicase during metazoan DNA replication initiation
Source: EMBO J. 2026 Sep 28;45(21):7582–620. doi: 10.1038/s44318-026-00915-9 (PMC13728148; doi:10.1038/s44318-026-00915-9)

Figure 2E - *mcm-10Δ* - 0’
This image is rotated by 270° in Figure 2E.
The 0’ timepoint Figure 2E corresponds to frame 26 (timestamp 4’10”) of Appendix Movie S13.

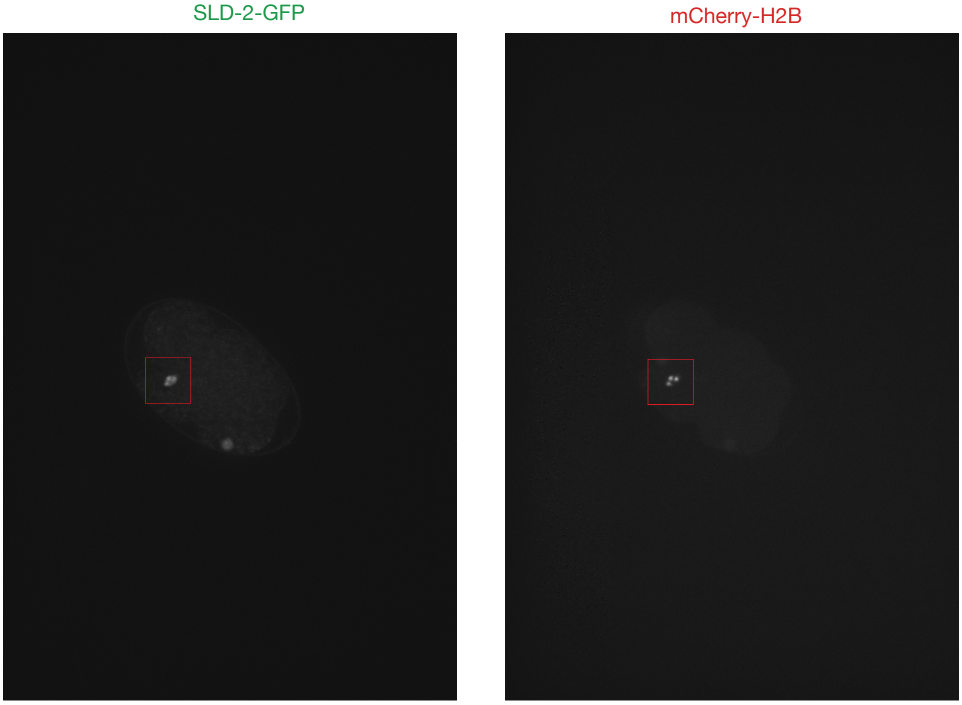

Figure 2E - *mcm-10Δ* + *GINS* RNAi - 0’
This image is rotated by 270° in Figure 2E.
The 0’ timepoint Figure 2E corresponds to frame 16 (timestamp 2’30”) of Appendix Movie S14.

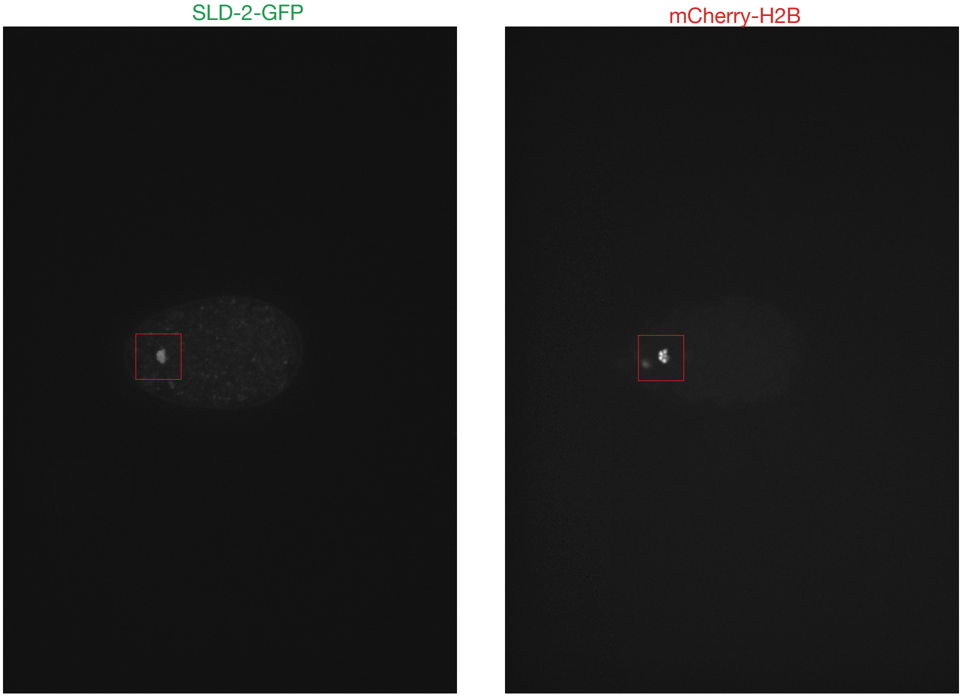

Supplement: Supplementary file 44 [file 44318_2026_915_MOESM49_ESM.zip › Figure 2_Source Data/2E/READ ME.pdf]
