# Supplementary material for: MCM10 and SLD-2/RECQL4 jointly activate the CMG helicase during metazoan DNA replication initiation
Source: EMBO J. 2026 Sep 28;45(21):7582–620. doi: 10.1038/s44318-026-00915-9 (PMC13728148; doi:10.1038/s44318-026-00915-9)

Figure 3E - *mcm-10*Δ - 0’
This image is rotated by 90° in Figure 3E and in Appendix Movie S23.
The 0’ timepoint Figure 3E corresponds to frame 5 (timestamp 0’40”) of Appendix Movie S23.

SLD-2-GFP mCherry-MUS-101

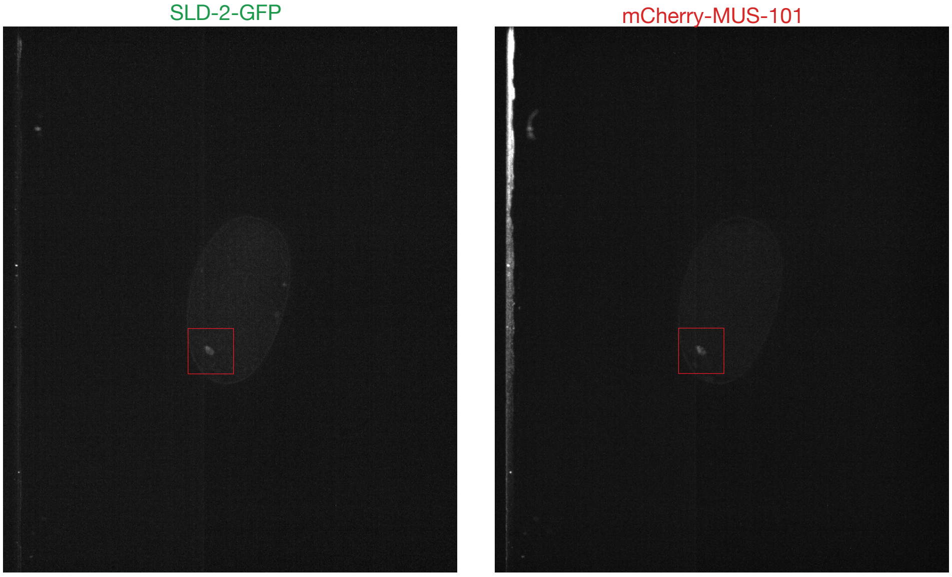

Figure 3E - *mcm-10*Δ + *GFP* RNAi - 0’
This image is rotated by 270° in Figure 3E and in Appendix Movie S24.
The 0’ timepoint Figure 3E corresponds to frame 3 (timestamp 0’20”) of Appendix Movie S24.

SLD-2-GFP mCherry-MUS-101

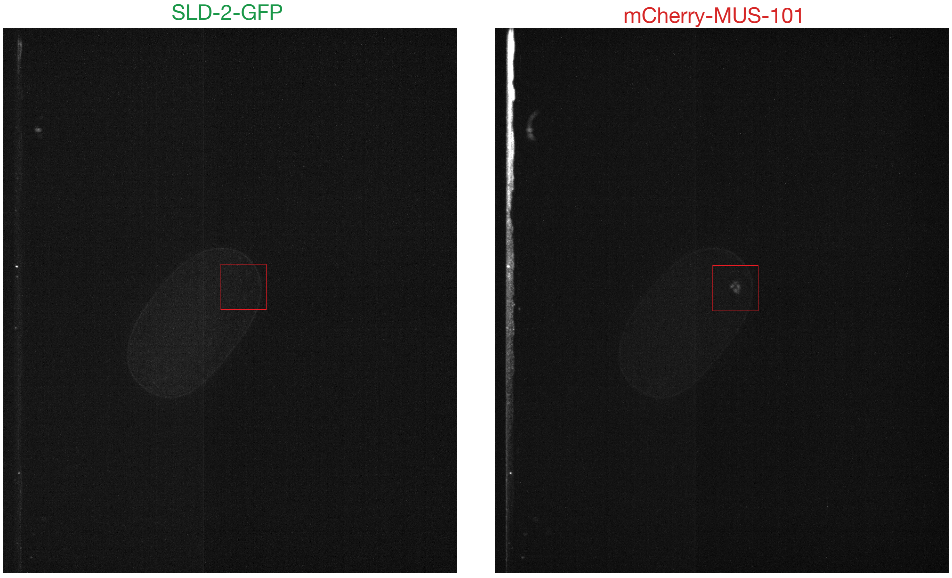

Supplement: Supplementary file 46 [file 44318_2026_915_MOESM50_ESM.zip › Figure 3_Source Data/3E/READ ME.pdf]
