# Supplementary material for: MCM10 and SLD-2/RECQL4 jointly activate the CMG helicase during metazoan DNA replication initiation
Source: EMBO J. 2026 Sep 28;45(21):7582–620. doi: 10.1038/s44318-026-00915-9 (PMC13728148; doi:10.1038/s44318-026-00915-9)

Figure 3D - *mcm-10Δ* - 0’
This image is rotated by 90° in Figure 3D and in Appendix Movie S21.
The 0’ timepoint Figure 3D corresponds to frame 21 (timestamp 3’20”) of Appendix Movie S21.

SLD-2-GFP

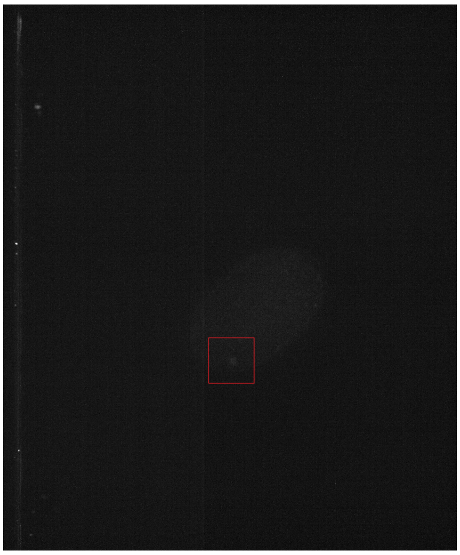

mCherry-DNSN-1

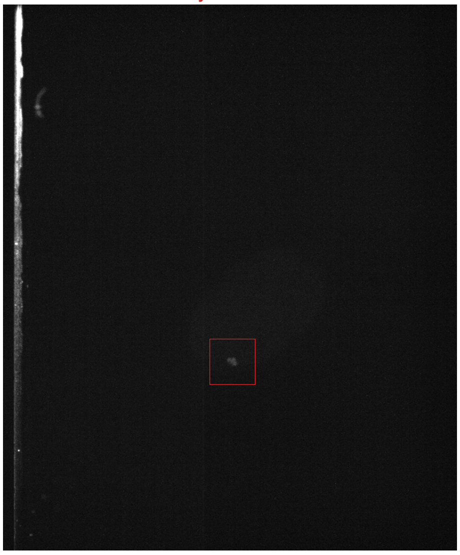

Figure 3D - *mcm-10Δ* + *GFP* RNAi - 0’
The 0’ timepoint Figure 3D corresponds to frame 8 (timestamp 1’10”) of Appendix Movie S22.

SLD-2-GFP

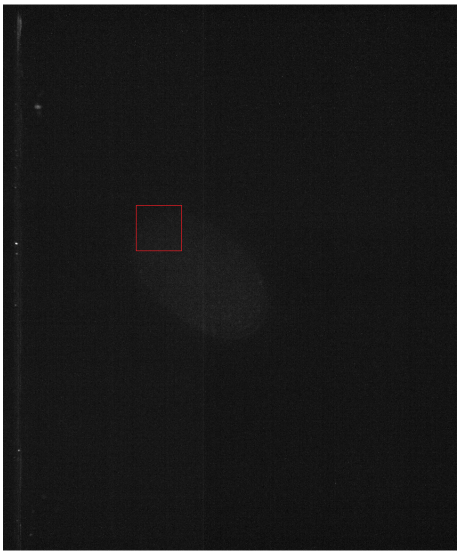

mCherry-DNSN-1

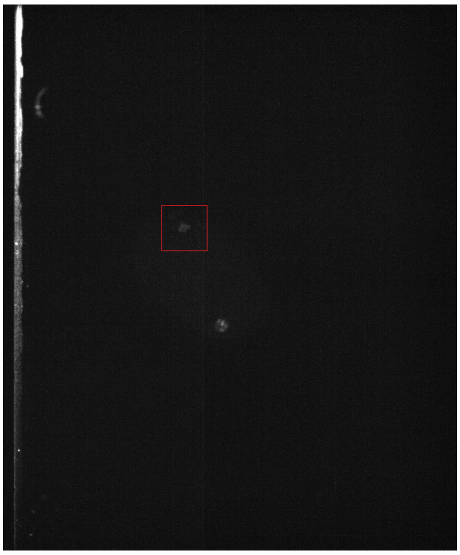

Supplement: Supplementary file 46 [file 44318_2026_915_MOESM50_ESM.zip › Figure 3_Source Data/3D/READ ME.pdf]
