# Supplementary material for: MCM10 and SLD-2/RECQL4 jointly activate the CMG helicase during metazoan DNA replication initiation
Source: EMBO J. 2026 Sep 28;45(21):7582–620. doi: 10.1038/s44318-026-00915-9 (PMC13728148; doi:10.1038/s44318-026-00915-9)

SLD-2-GFP | mCherry-RPA-2

Figure 3H - *mcm-10*Δ - Prophase
The first Prophase timepoint in Figure 3H corresponds to frame 61 (timestamp 13’40”) of Appendix Movie S29.

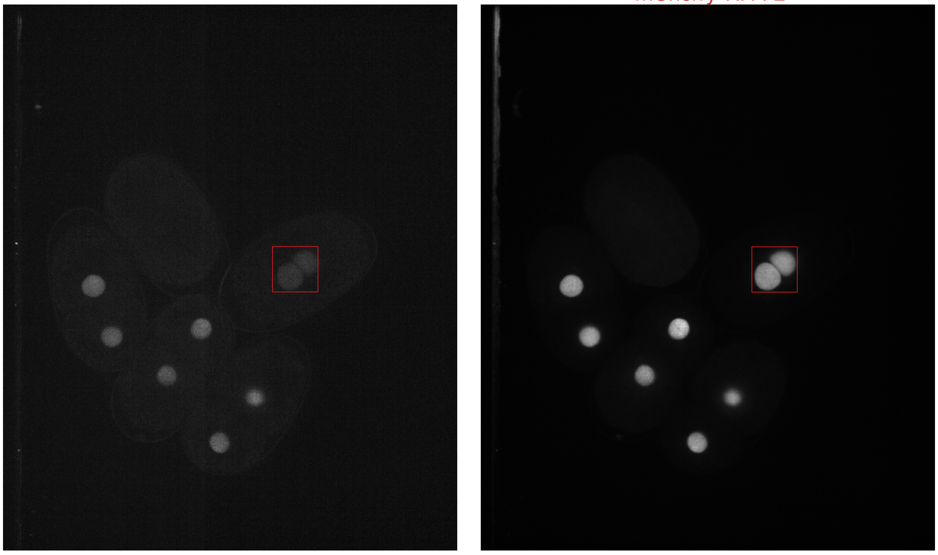

Figure 3H - *mcm-10*Δ + *rnr-1* RNAi - Prophase
This image is rotated by 180° in Appendix Movie S30.
The first Prophase timepoint in Figure 3H corresponds to frame 108 (timestamp 18’10”) of Appendix Movie S30.

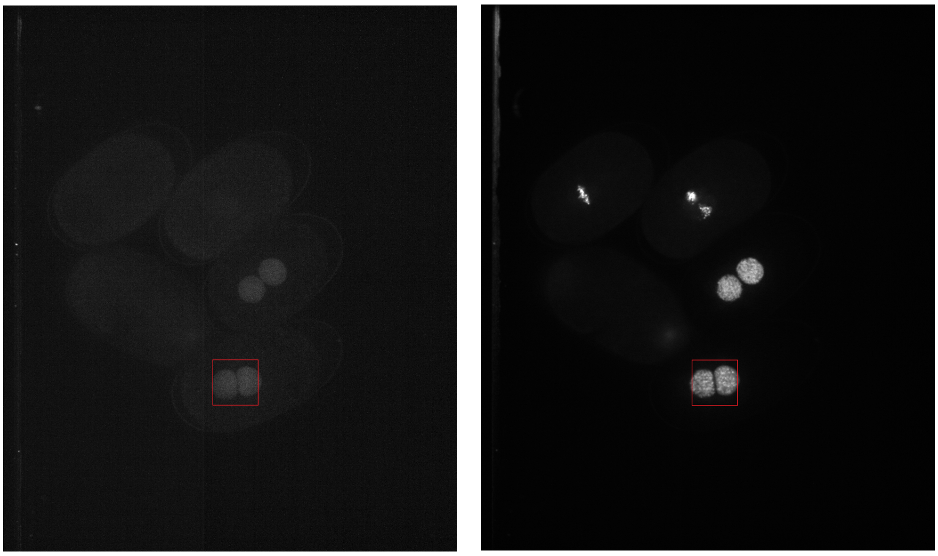

Figure 3H - *mcm-10*Δ + *cdc-45* RNAi - Prophase
The first Prophase timepoint in Figure 3H corresponds to frame 42 (timestamp 7’10”) of Appendix Movie S31.

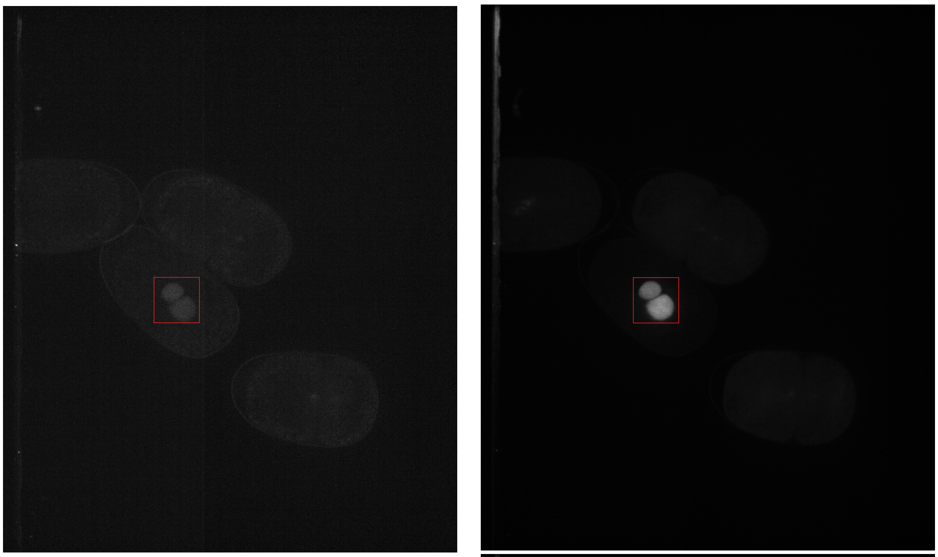

Figure 3H - *mcm-10*Δ + *GFP* RNAi - Prophase
The first Prophase timepoint in Figure 3H corresponds to frame 81 (timestamp 13’10”) of Appendix Movie S32.

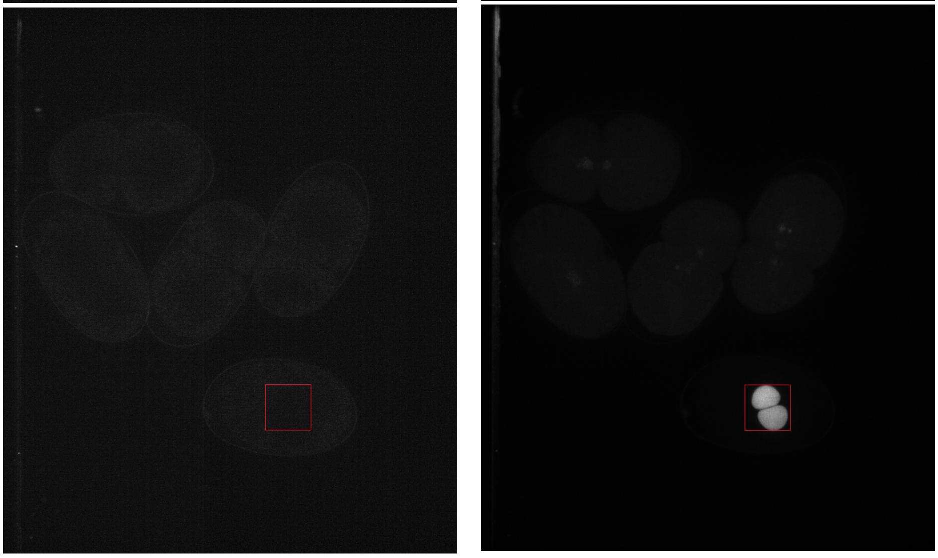

Supplement: Supplementary file 46 [file 44318_2026_915_MOESM50_ESM.zip › Figure 3_Source Data/3H/READ ME.pdf]
