# Supplementary material for: MCM10 and SLD-2/RECQL4 jointly activate the CMG helicase during metazoan DNA replication initiation
Source: EMBO J. 2026 Sep 28;45(21):7582–620. doi: 10.1038/s44318-026-00915-9 (PMC13728148; doi:10.1038/s44318-026-00915-9)

Figure 3A - *mcm-10∆* - 0’
The 0’ timepoint Figure 3E corresponds to frame 1 (timestamp 0’00”) of Appendix Movie S15.

SLD-2-GFP

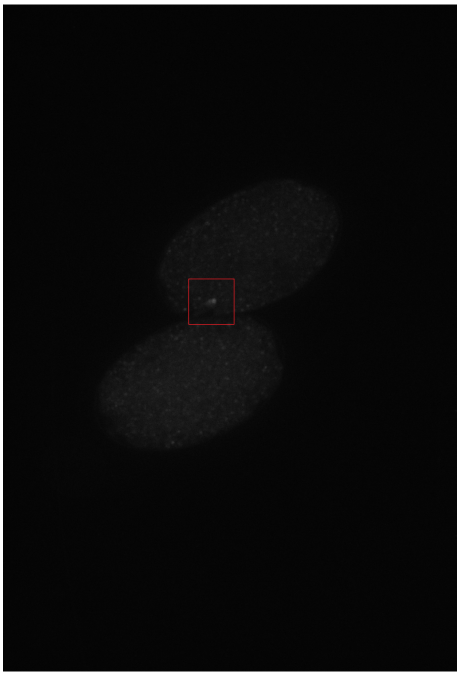

mCherry-H2B

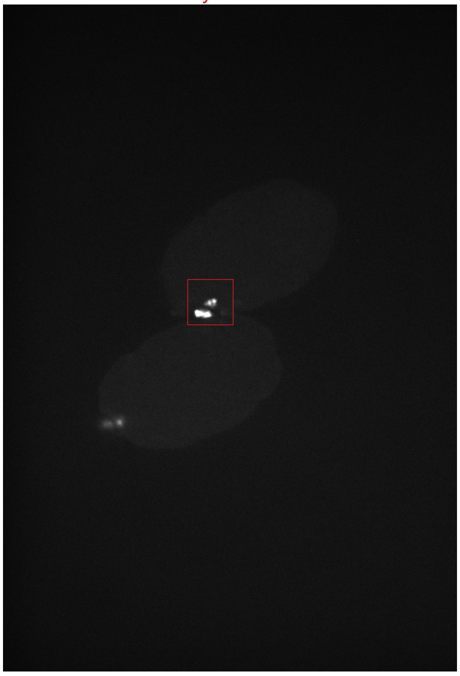

Figure 3A - *mcm-10∆* + *GFP* RNAi - 0’
This image is rotated by 270° in Figure 3E and in Appendix Movie S16.
The 0’ timepoint Figure 3E corresponds to frame 6 (timestamp 0’50”) of Appendix Movie S16.

SLD-2-GFP

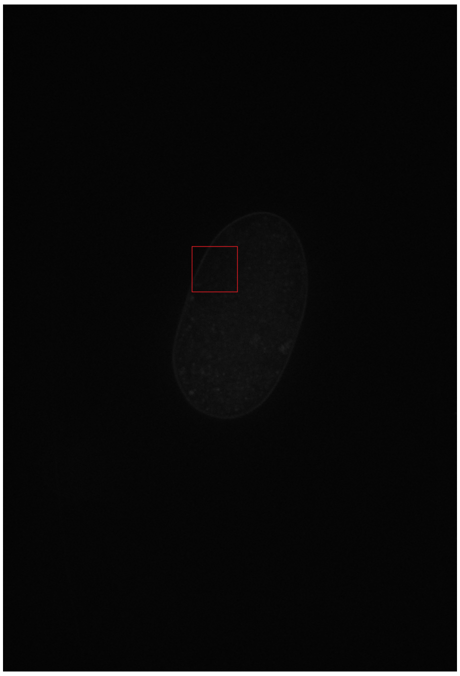

mCherry-H2B

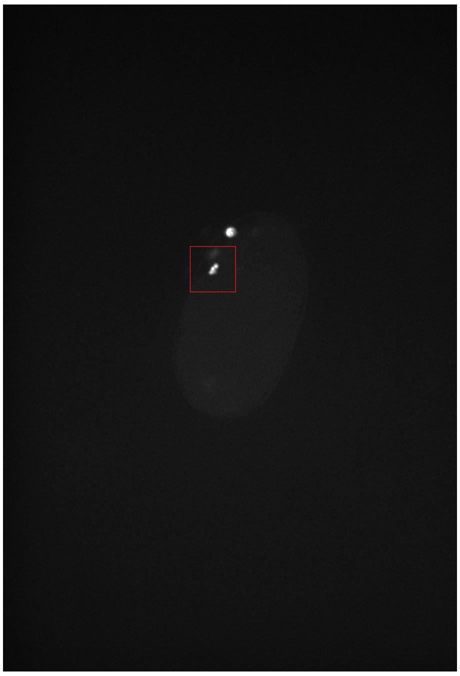

Supplement: Supplementary file 46 [file 44318_2026_915_MOESM50_ESM.zip › Figure 3_Source Data/3A/READ ME.pdf]
