# Supplementary material for: MCM10 and SLD-2/RECQL4 jointly activate the CMG helicase during metazoan DNA replication initiation
Source: EMBO J. 2026 Sep 28;45(21):7582–620. doi: 10.1038/s44318-026-00915-9 (PMC13728148; doi:10.1038/s44318-026-00915-9)

Figure 3F - *mcm-10*Δ - 0’
This image is rotated by 180° in Figure 1A and in Appendix Movie S25.
The 0’ timepoint Figure 3F corresponds to frame 6 (timestamp 0’50”) of Appendix Movie S25.

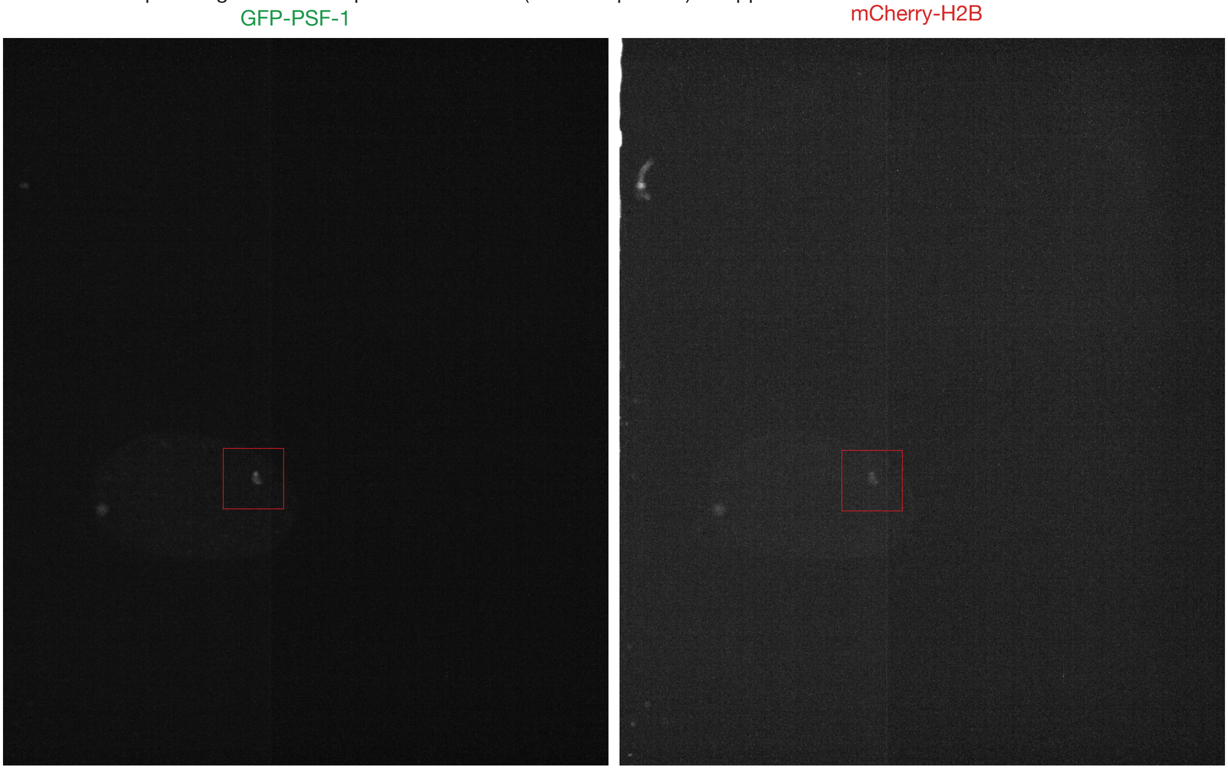

Figure 3F - *GFP* RNAi; *mcm-10*Δ - 0’
This image is rotated by 90° in Figure 1A and in Appendix Movie S26.
The 0’ timepoint Figure 3F corresponds to frame 12 (timestamp 1’50”) of Appendix Movie S26.

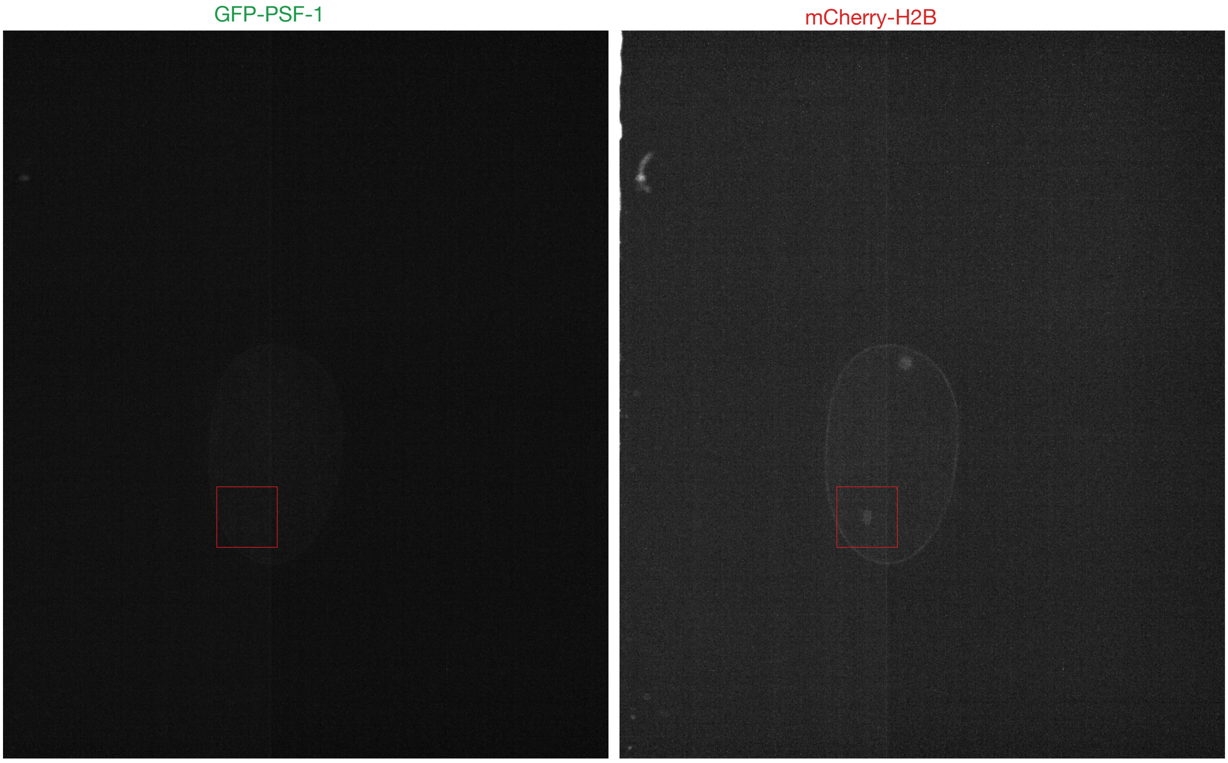

Supplement: Supplementary file 46 [file 44318_2026_915_MOESM50_ESM.zip › Figure 3_Source Data/3F/READ ME.pdf]
