# Supplementary material for: MCM10 and SLD-2/RECQL4 jointly activate the CMG helicase during metazoan DNA replication initiation
Source: EMBO J. 2026 Sep 28;45(21):7582–620. doi: 10.1038/s44318-026-00915-9 (PMC13728148; doi:10.1038/s44318-026-00915-9)

Figure 3G - *mcm-10*Δ - Prophase

The ‘Prophase’ timepoint Figure 3G corresponds to frame 18 (timestamp 2’40”) of Appendix Movie S27.

SLD-2-GFP TIM-1-mCherry

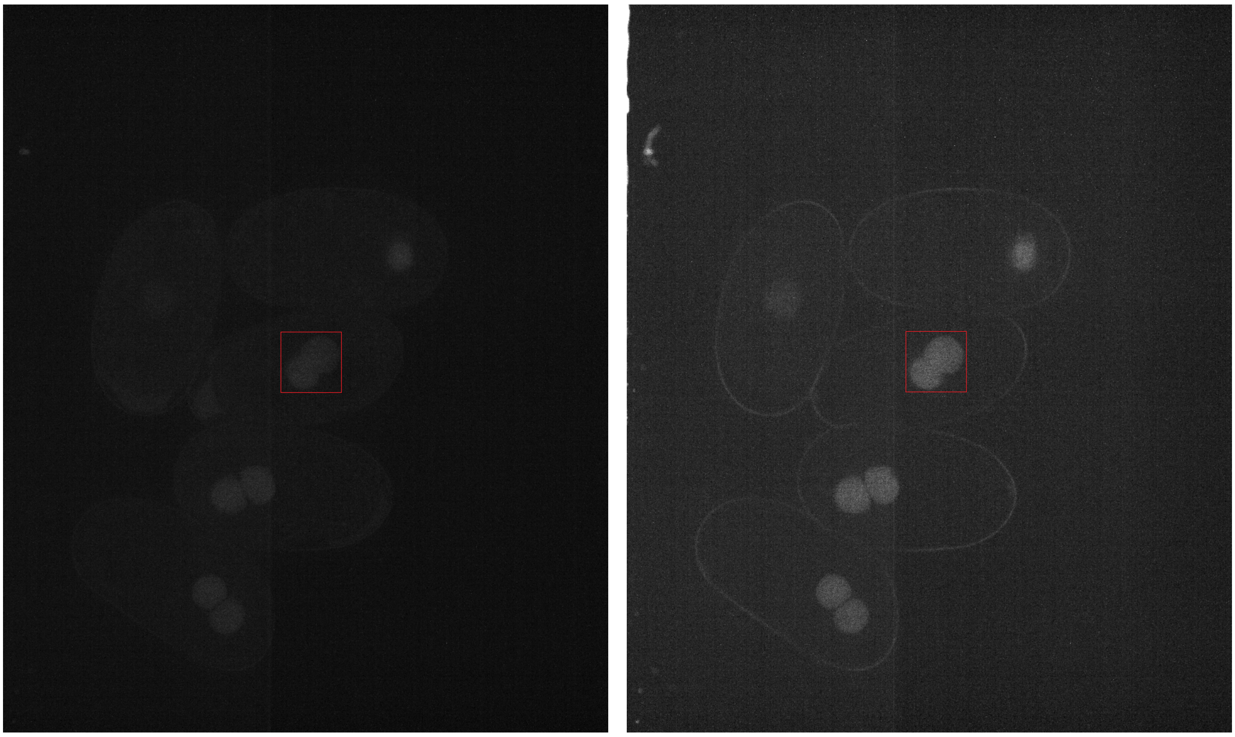

Figure 3G - *npl-4* RNAi + *mcm-10*Δ - Prophase

This image is rotated by 180° in Appendix Movie S28.

The ‘Prophase’ timepoint Figure 3G corresponds to frame 34 (timestamp 5’20”) of Appendix Movie S28.

SLD-2-GFP TIM-1-mCherry

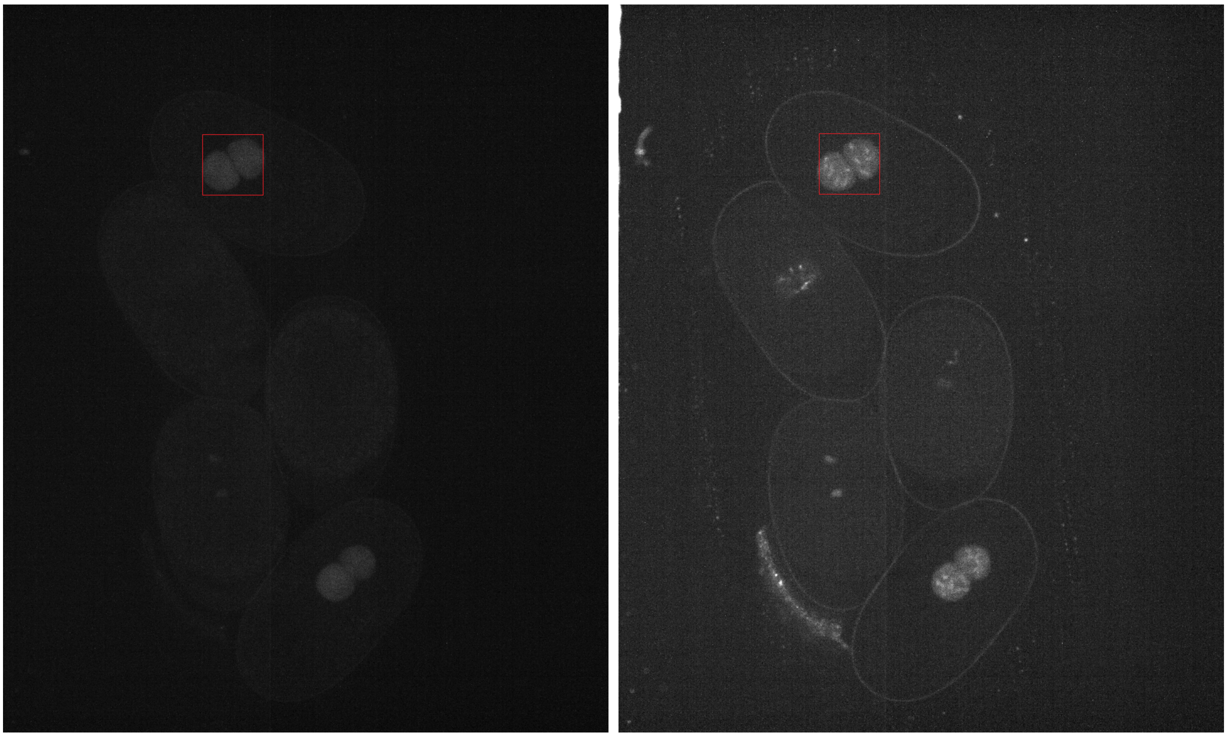

Supplement: Supplementary file 46 [file 44318_2026_915_MOESM50_ESM.zip › Figure 3_Source Data/3G/READ ME.pdf]
