# Supplementary material for: MCM10 and SLD-2/RECQL4 jointly activate the CMG helicase during metazoan DNA replication initiation
Source: EMBO J. 2026 Sep 28;45(21):7582–620. doi: 10.1038/s44318-026-00915-9 (PMC13728148; doi:10.1038/s44318-026-00915-9)
Supplement: Supplementary file 47 [file 44318_2026_915_MOESM51_ESM.zip › Figure 4_Source Data/4E/Figure 4E - CMG helicase assays with MCM-10.pdf]

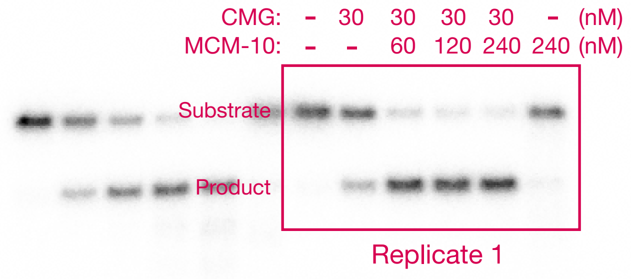
CMG: – 30 30 30 30 – (nM)
MCM-10: – – 60 120 240 240 (nM)
Substrate
Product
Replicate 1

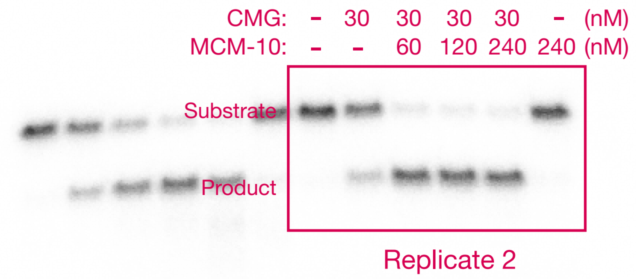
CMG: – 30 30 30 30 – (nM)
MCM-10: – – 60 120 240 240 (nM)
Substrate
Product
Replicate 2

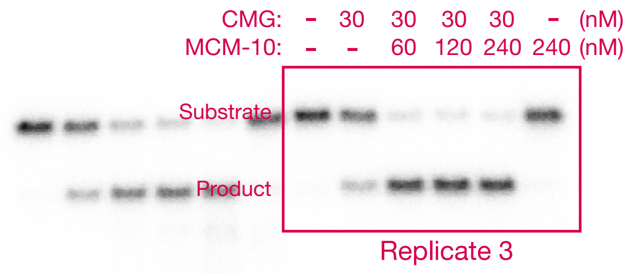
CMG: – 30 30 30 30 – (nM)
MCM-10: – – 60 120 240 240 (nM)
Substrate
Product
Replicate 3
