# Supplementary material for: MCM10 and SLD-2/RECQL4 jointly activate the CMG helicase during metazoan DNA replication initiation
Source: EMBO J. 2026 Sep 28;45(21):7582–620. doi: 10.1038/s44318-026-00915-9 (PMC13728148; doi:10.1038/s44318-026-00915-9)
Supplement: Supplementary file 47 [file 44318_2026_915_MOESM51_ESM.zip › Figure 4_Source Data/4A/Figure 4A _coomassie gel.pdf]

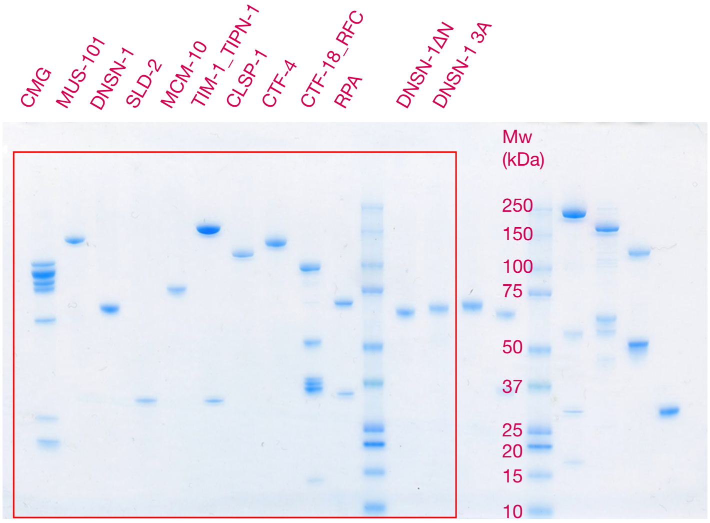
CMG
MUS-101
DNSN-1
SLD-2
MCM-10
TIM-1_TIPN-1
CLSP-1
CTF-4
CTF-18_RFC
RPA
DNSN-1ΔN
DNSN-1 3A
Mw
(kDa)
250
150
100
75
50
37
25
20
15
10
