# Supplementary material for: MCM10 and SLD-2/RECQL4 jointly activate the CMG helicase during metazoan DNA replication initiation
Source: EMBO J. 2026 Sep 28;45(21):7582–620. doi: 10.1038/s44318-026-00915-9 (PMC13728148; doi:10.1038/s44318-026-00915-9)
Supplement: Supplementary file 48 [file 44318_2026_915_MOESM52_ESM.zip › Figure 5_Source Data/5C/5C_Plates BromoTag-Mcm10 dTag-Recql4 +Recql4 1-468 clone2.pdf]

BT-Mcm10 dT-Recql4 +Recql4 1-468
dTAG13: +
AGB1: -

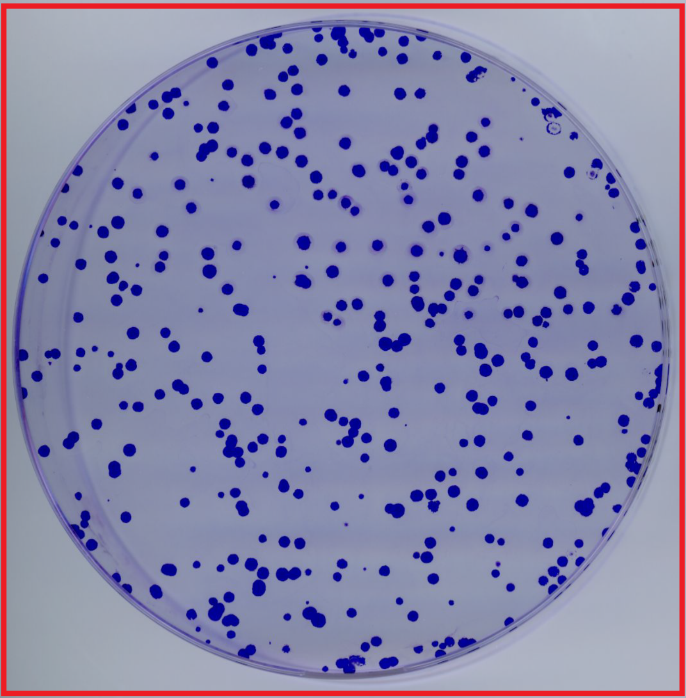

BT-Mcm10 dT-Recql4 +Recql4 1-468
dTAG13: -
AGB1: +

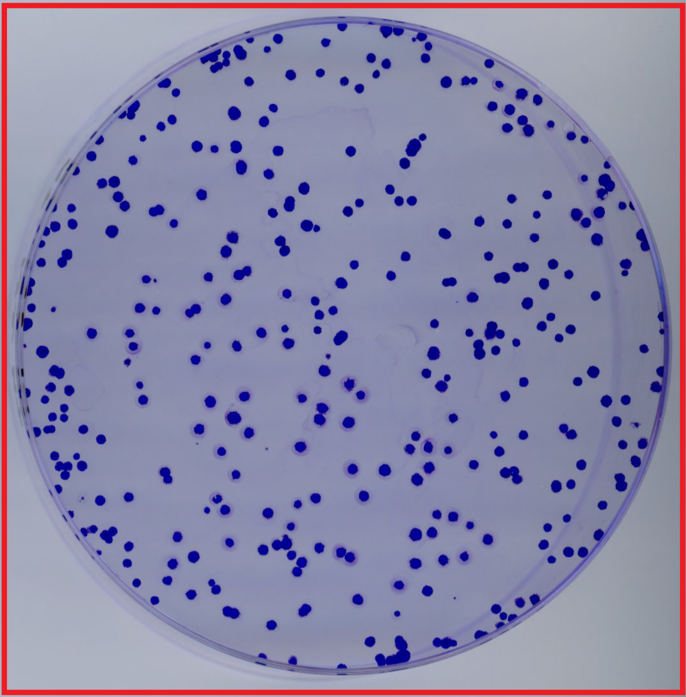

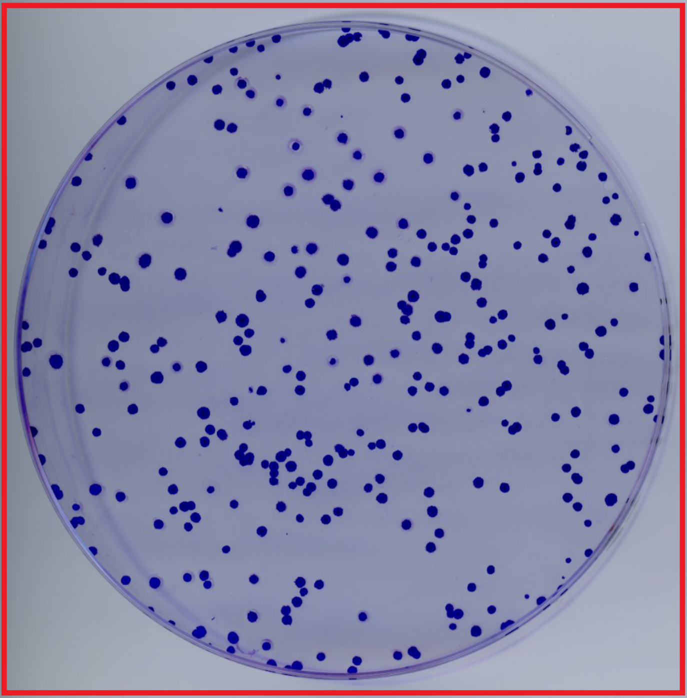

BT-Mcm10 dT-Recql4 +Recql4 1-468
dTAG13: +
AGB1: +

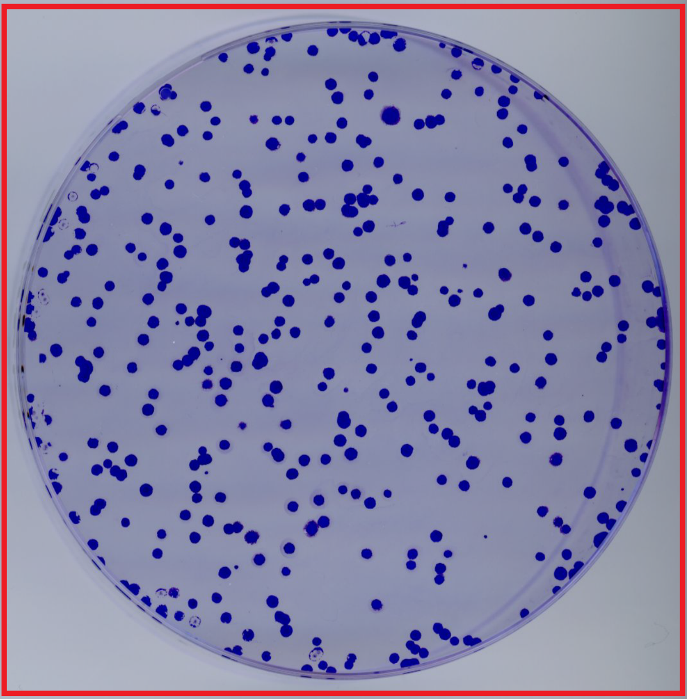

BT-Mcm10 dT-Recql4 +Recql4 1-468
dTAG13: -
AGB1: -
