# Supplementary material for: MCM10 and SLD-2/RECQL4 jointly activate the CMG helicase during metazoan DNA replication initiation
Source: EMBO J. 2026 Sep 28;45(21):7582–620. doi: 10.1038/s44318-026-00915-9 (PMC13728148; doi:10.1038/s44318-026-00915-9)
Supplement: Supplementary file 48 [file 44318_2026_915_MOESM52_ESM.zip › Figure 5_Source Data/5E/Figure 5E_Blot_Mcm10_Control.pdf]

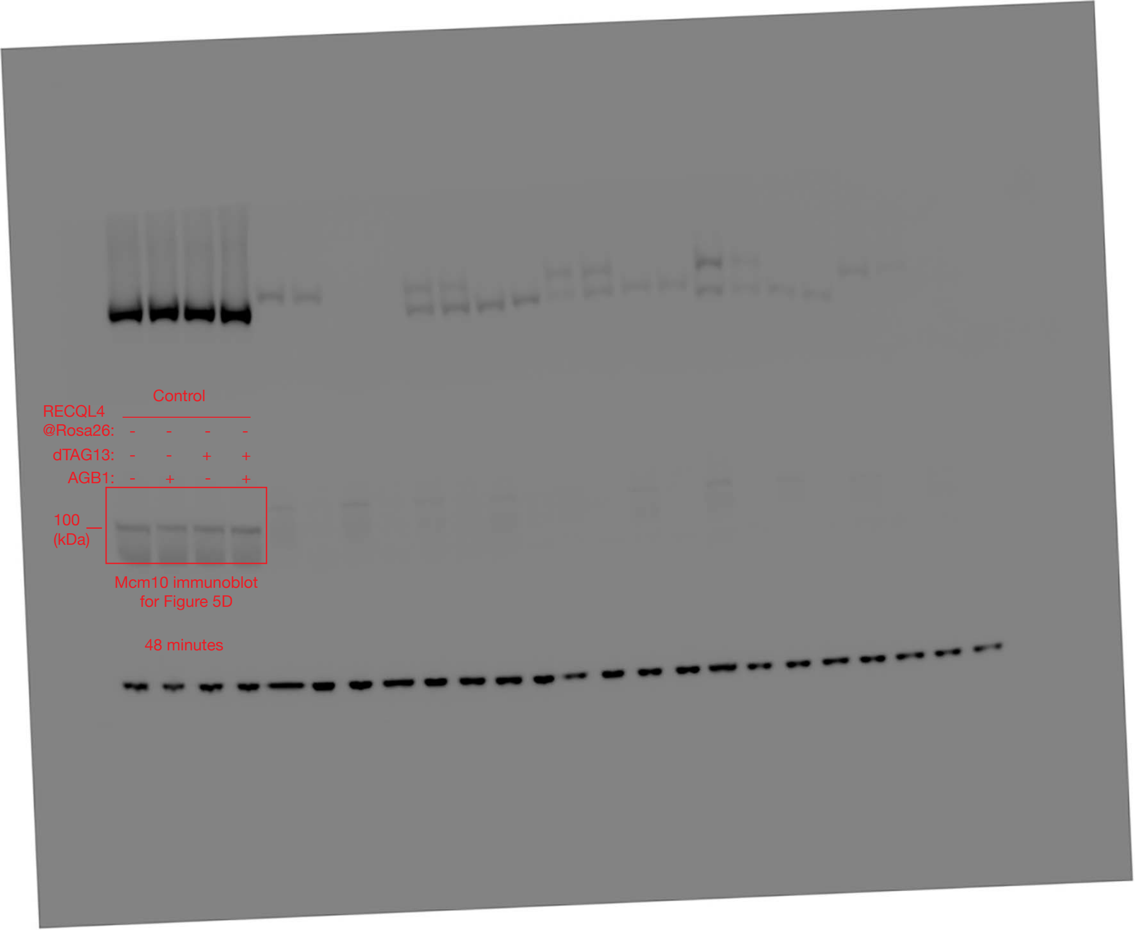
Control
RECQL4
@Rosa26: - - - -
dTAG13: - - + +
AGB1: - + - +
100
(kDa)
Mcm10 immunoblot
for Figure 5D
48 minutes
