# Supplementary material for: MCM10 and SLD-2/RECQL4 jointly activate the CMG helicase during metazoan DNA replication initiation
Source: EMBO J. 2026 Sep 28;45(21):7582–620. doi: 10.1038/s44318-026-00915-9 (PMC13728148; doi:10.1038/s44318-026-00915-9)
Supplement: Supplementary file 48 [file 44318_2026_915_MOESM52_ESM.zip › Figure 5_Source Data/5E/Figure 5E_Blot_Psf3.pdf]

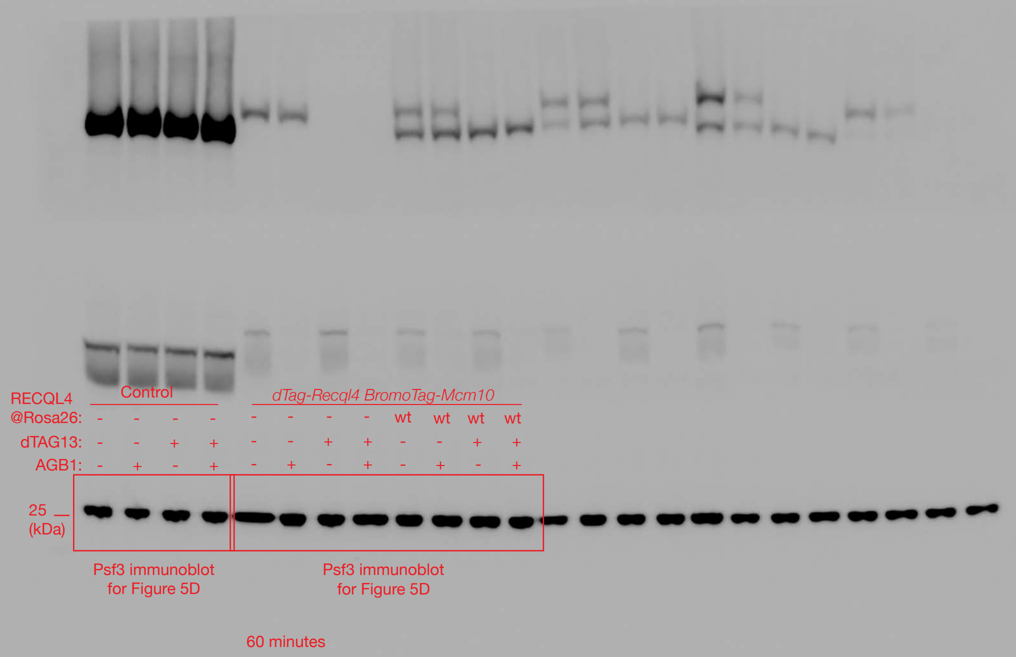
RECQL4
@Rosa26:
dTAG13:
AGB1:
Control
dTag-Recql4 BromoTag-Mcm10
- - - - - - - - wt wt wt wt
- - + + - - + + - - + +
- + - + - + - + - + - +
25
(kDa)
Psf3 immunoblot
for Figure 5D
Psf3 immunoblot
for Figure 5D
60 minutes
