# Supplementary material for: MCM10 and SLD-2/RECQL4 jointly activate the CMG helicase during metazoan DNA replication initiation
Source: EMBO J. 2026 Sep 28;45(21):7582–620. doi: 10.1038/s44318-026-00915-9 (PMC13728148; doi:10.1038/s44318-026-00915-9)
Supplement: Supplementary file 48 [file 44318_2026_915_MOESM52_ESM.zip › Figure 5_Source Data/5B/Figure 5B_Plates Mcm10 del_Recql4 gRNAs.pdf]

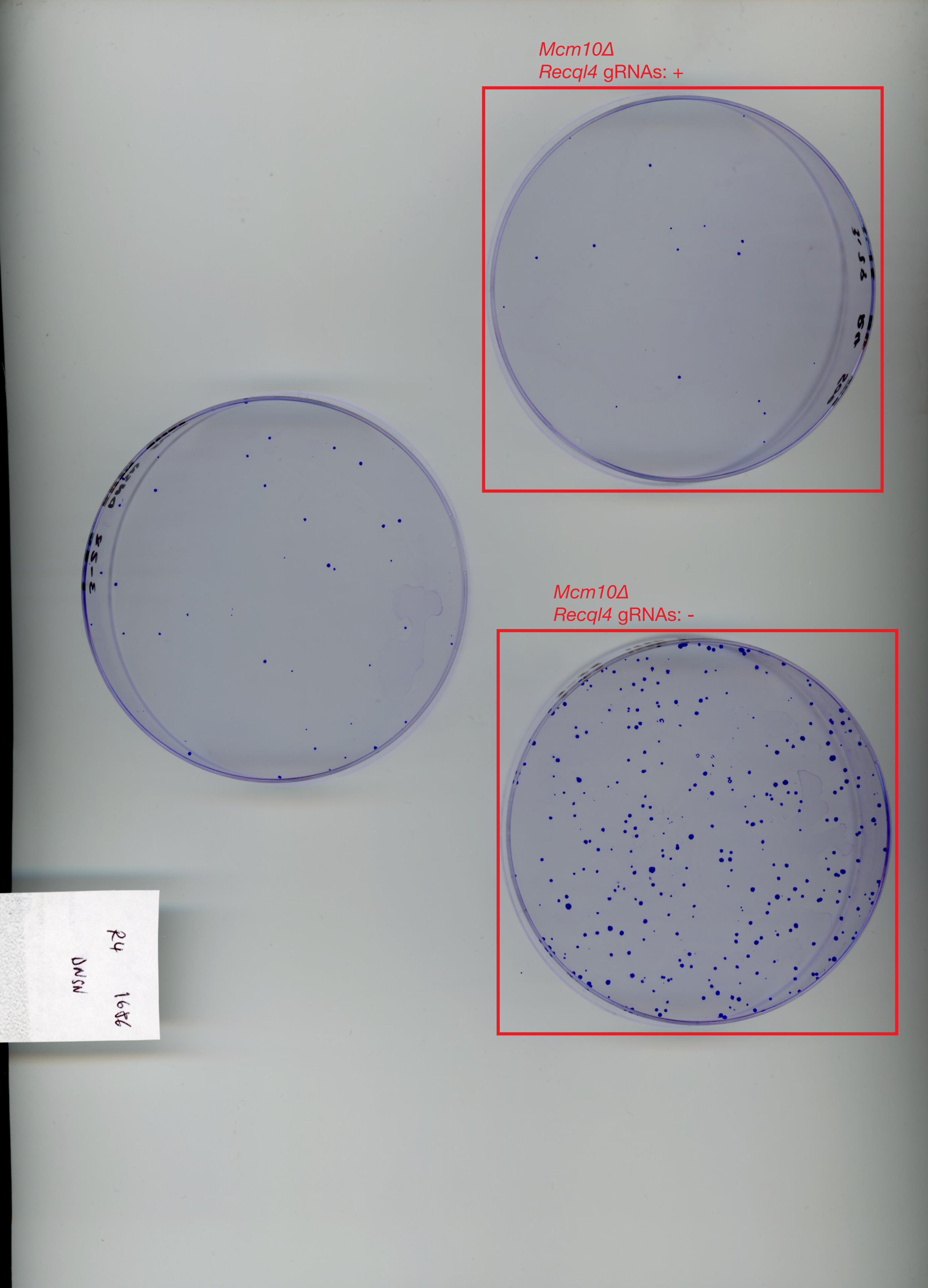
Mcm10Δ
Recql4 gRNAs: +
Mcm10Δ
Recql4 gRNAs: -
