# Supplementary material for: MCM10 and SLD-2/RECQL4 jointly activate the CMG helicase during metazoan DNA replication initiation
Source: EMBO J. 2026 Sep 28;45(21):7582–620. doi: 10.1038/s44318-026-00915-9 (PMC13728148; doi:10.1038/s44318-026-00915-9)
Supplement: Supplementary file 49 [file 44318_2026_915_MOESM53_ESM.zip › Figure 6_Source Data/6B/Figure 6B_Blot_Mcm10.pdf]

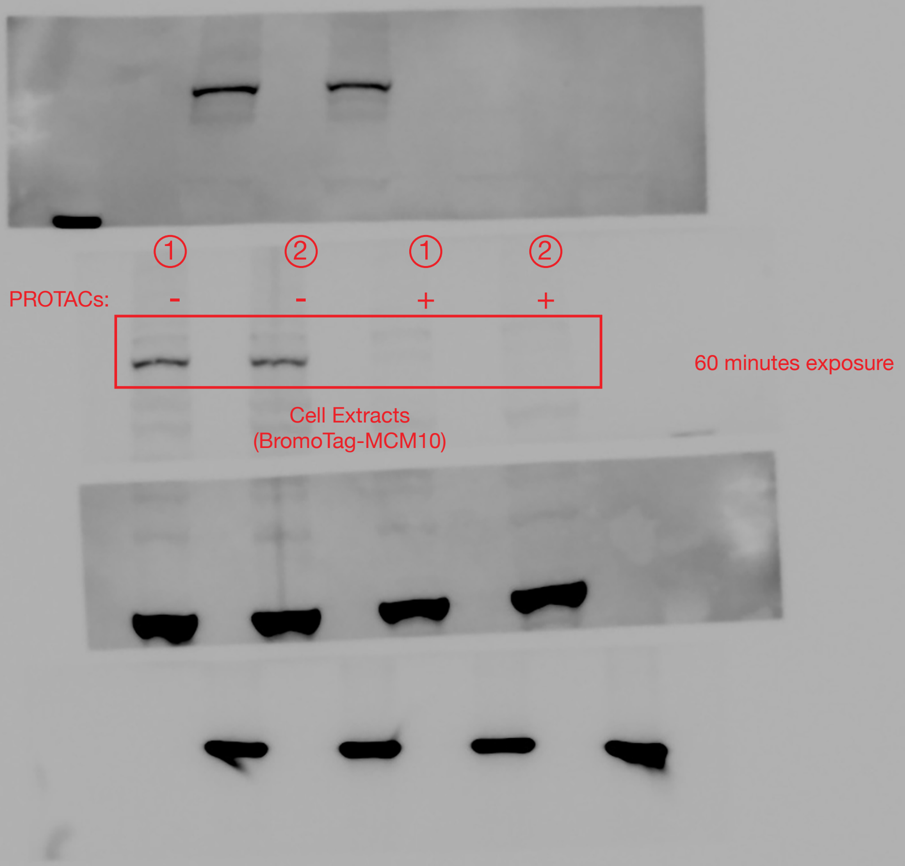

①
②
①
②
PROTACs:
-
-
+
+
60 minutes exposure
Cell Extracts
(BromoTag-MCM10)
