# Supplementary material for: MCM10 and SLD-2/RECQL4 jointly activate the CMG helicase during metazoan DNA replication initiation
Source: EMBO J. 2026 Sep 28;45(21):7582–620. doi: 10.1038/s44318-026-00915-9 (PMC13728148; doi:10.1038/s44318-026-00915-9)
Supplement: Supplementary file 50 [file 44318_2026_915_MOESM54_ESM.zip › Figure 7_Source Data/7F/Figure 7F_Blot MCM10.pdf]

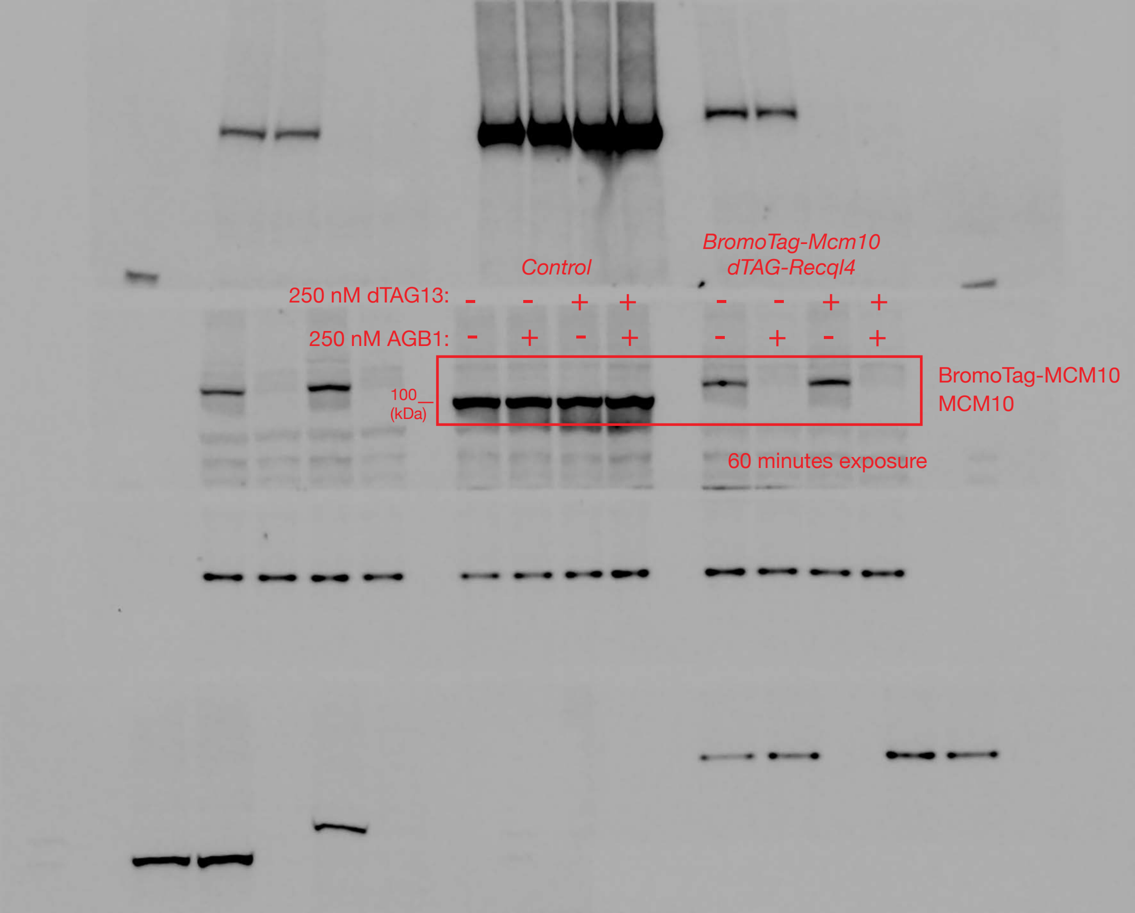
Control
BromoTag-Mcm10
dTAG-Recql4
250 nM dTAG13: - - + + - - + +
250 nM AGB1: - + - + - + - +
100
(kDa)
BromoTag-MCM10
MCM10
60 minutes exposure
