# Supplementary material for: MCM10 and SLD-2/RECQL4 jointly activate the CMG helicase during metazoan DNA replication initiation
Source: EMBO J. 2026 Sep 28;45(21):7582–620. doi: 10.1038/s44318-026-00915-9 (PMC13728148; doi:10.1038/s44318-026-00915-9)

Mouse ES cells (*BromoTag-Mcm10 dTAG-Recql4 GFP-Psf1 mCherry-Timeless*)

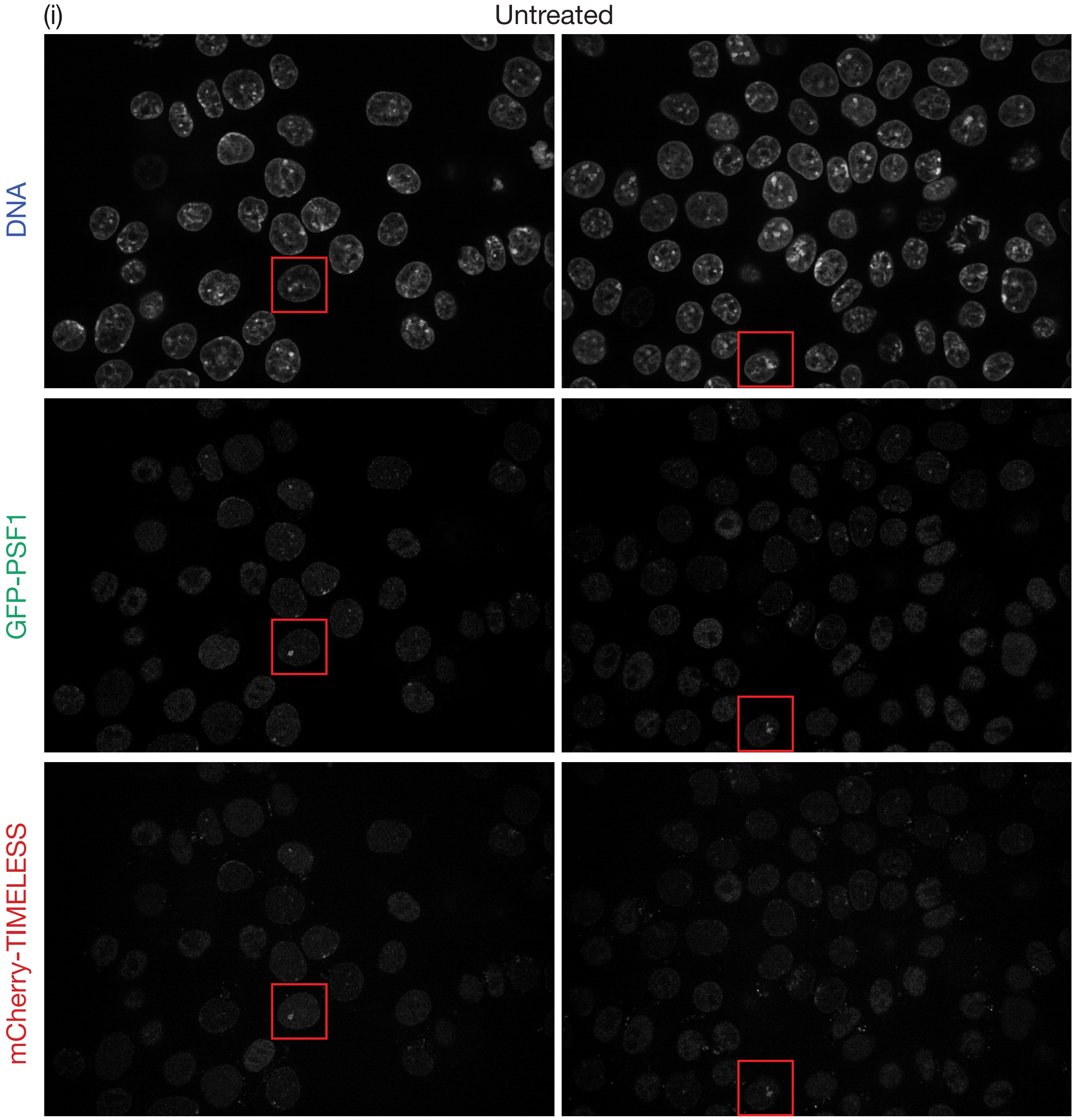

Mouse ES cells (*BromoTag-Mcm10 dTAG-Recql4 GFP-Psf1 mCherry-Timeless*)

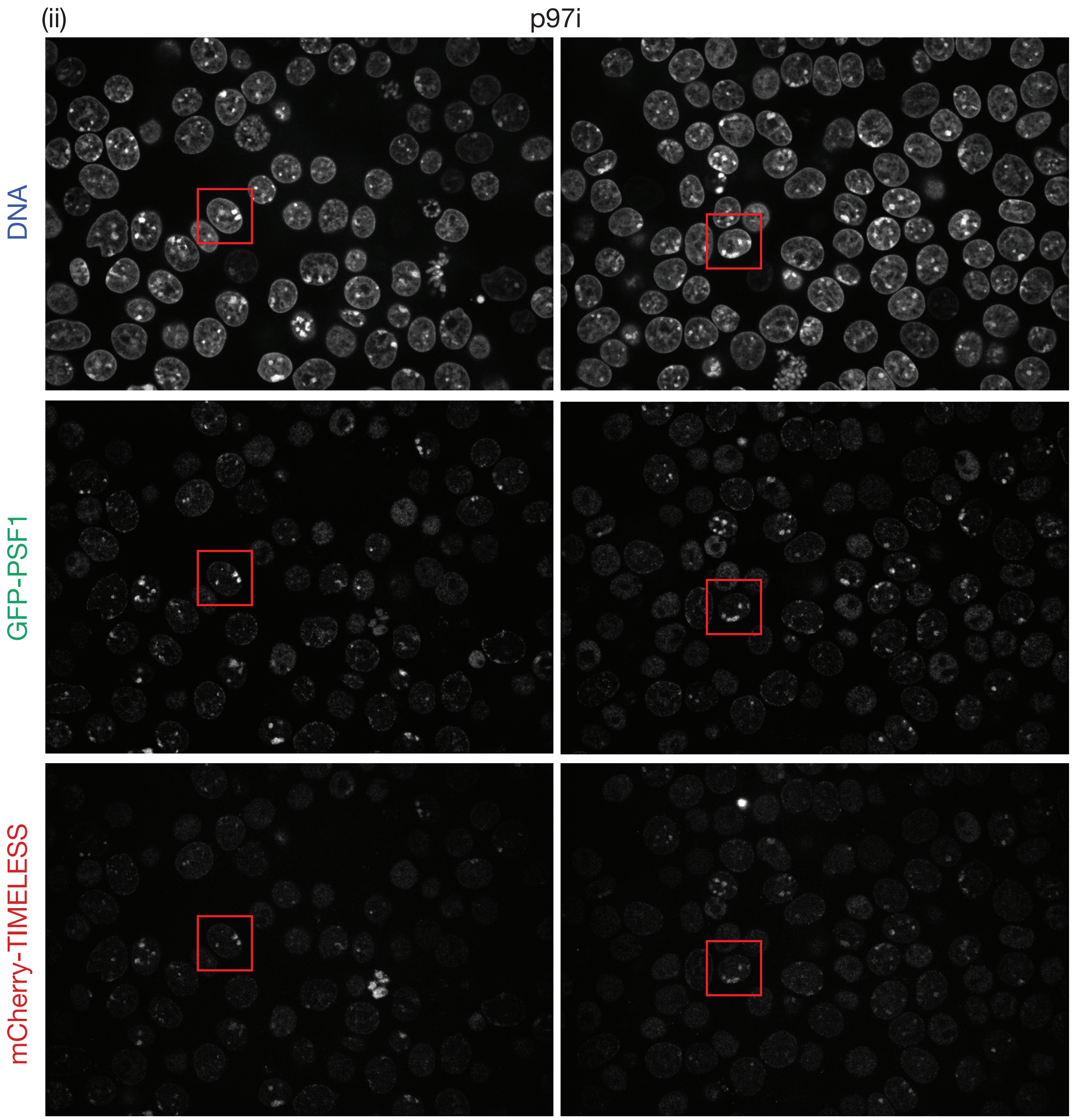

Mouse ES cells (*BromoTag-Mcm10 dTAG-Recql4 GFP-Psf1 mCherry-Timeless*)

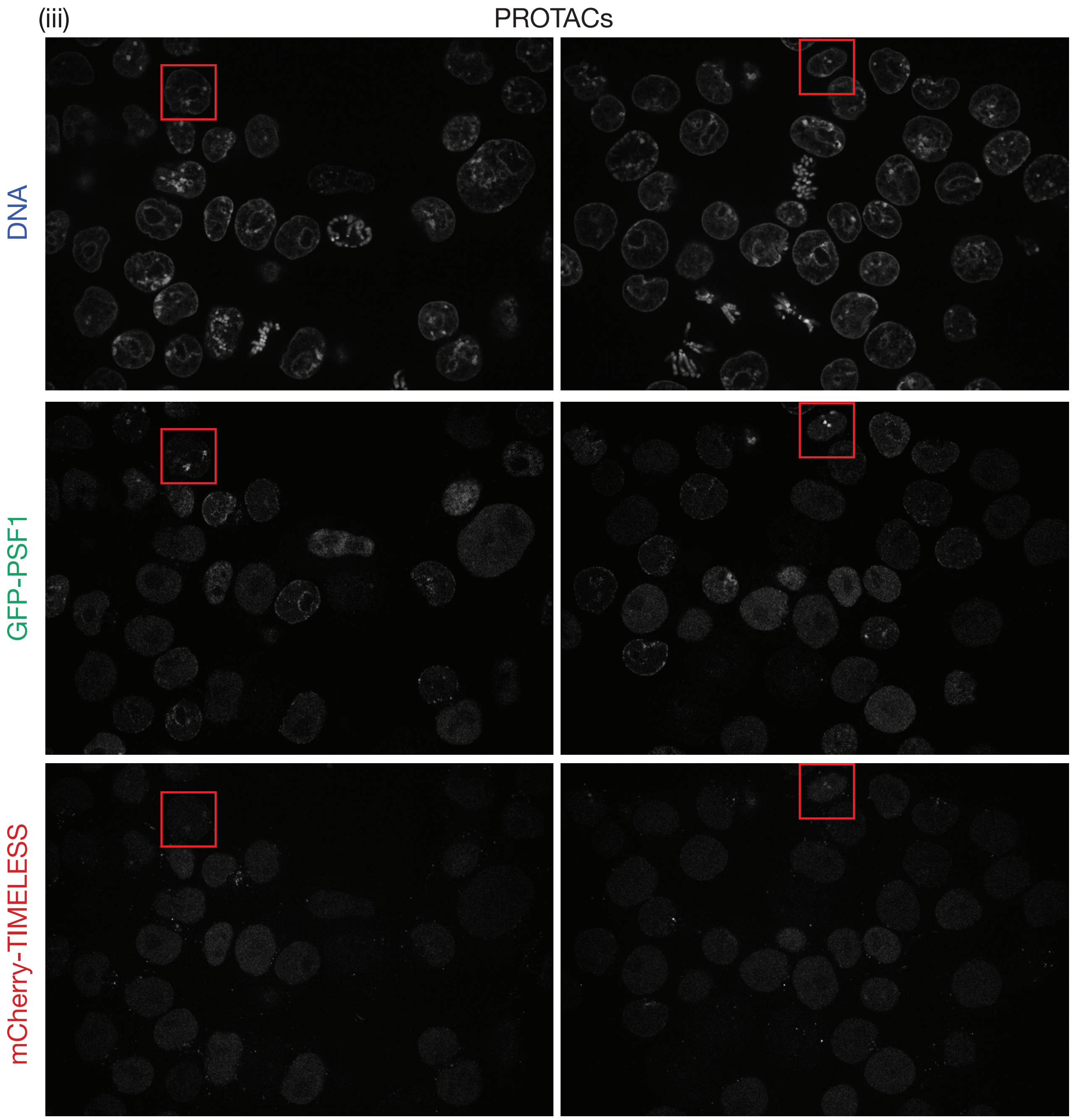

Supplement: Supplementary file 51 [file 44318_2026_915_MOESM55_ESM.zip › Figure 8_Source Data/8G/README.pdf]
