# Supplementary material for: MCM10 and SLD-2/RECQL4 jointly activate the CMG helicase during metazoan DNA replication initiation
Source: EMBO J. 2026 Sep 28;45(21):7582–620. doi: 10.1038/s44318-026-00915-9 (PMC13728148; doi:10.1038/s44318-026-00915-9)
Supplement: Supplementary file 51 [file 44318_2026_915_MOESM55_ESM.zip › Figure 8_Source Data/8F/Figure 8F_Blot PSF3.pdf]

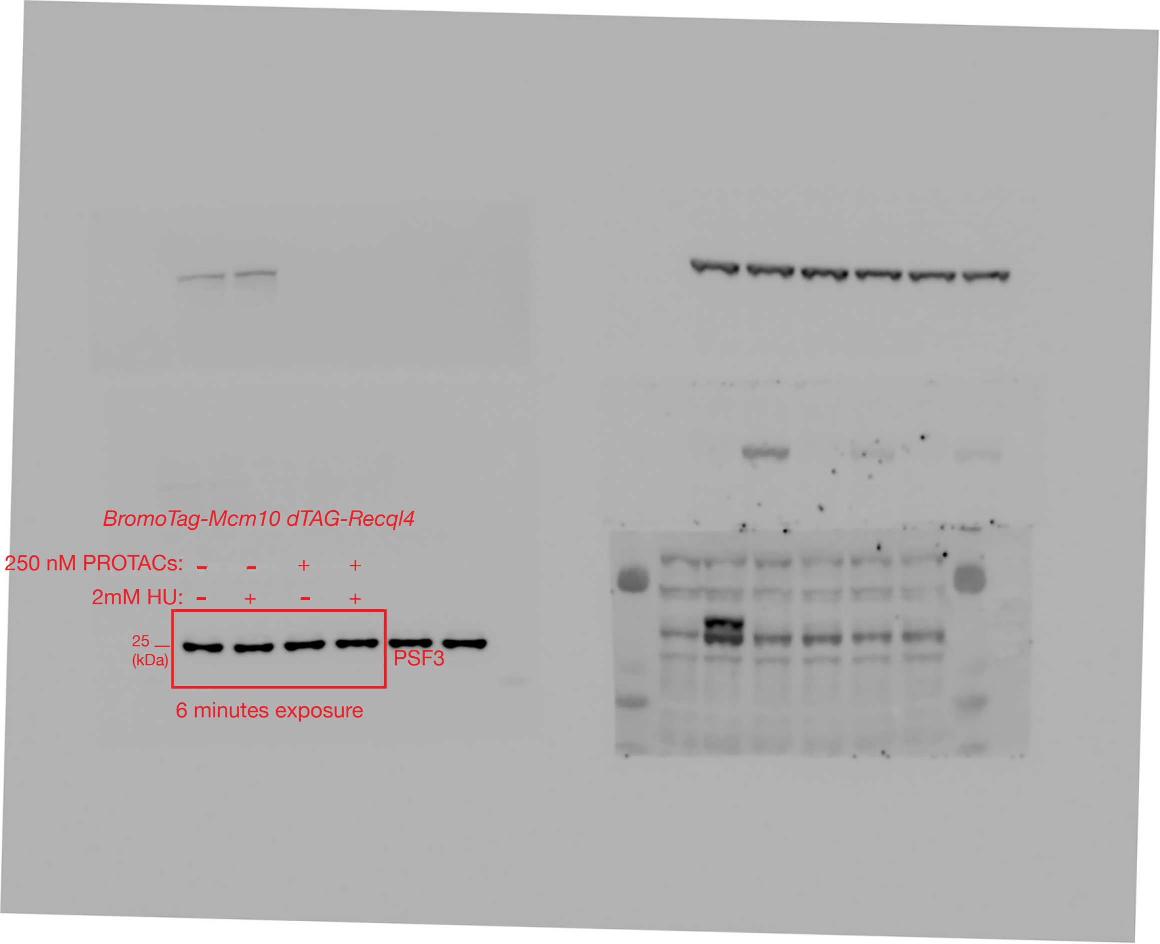
BromoTag-Mcm10 dTAG-Recql4
250 nM PROTACs: - - + +
2mM HU: - + - +
25
(kDa)
PSF3
6 minutes exposure
