# Supplementary material for: MCM10 and SLD-2/RECQL4 jointly activate the CMG helicase during metazoan DNA replication initiation
Source: EMBO J. 2026 Sep 28;45(21):7582–620. doi: 10.1038/s44318-026-00915-9 (PMC13728148; doi:10.1038/s44318-026-00915-9)
Supplement: Supplementary file 51 [file 44318_2026_915_MOESM55_ESM.zip › Figure 8_Source Data/8D/README.pdf]

This source data file contains the raw measurements used to generate the scatter plot shown in the main figure. Each row corresponds to a single nucleus.

The columns are:

Mean Intensity RPA32: Mean fluorescence intensity of the RPA32 signal for each nucleus (y-axis of the scatter plot).

Total Intensity DAPI: Total fluorescence intensity of the DAPI signal for each nucleus (x-axis of the scatter plot).

Cell type and treatment: Experimental group corresponding to each nucleus (UT = untreated; 14h+2h PROTACS = 'PROTACS' = 16h treatment with PROTACS; 2mM HU 2h = 'HU'; 14h PROTACS + 2h HU = 'PROTACS + HU' = 14h PROTACS + 2h PROTACS + HU). Genotype of all cells was *BromoTag-Mcm10 dTAG-Recql4*. This column contains multiple conditions and can be filtered.
