# Supplementary material for: MCM10 and SLD-2/RECQL4 jointly activate the CMG helicase during metazoan DNA replication initiation
Source: EMBO J. 2026 Sep 28;45(21):7582–620. doi: 10.1038/s44318-026-00915-9 (PMC13728148; doi:10.1038/s44318-026-00915-9)

Figure S2 - *sld-2* RNAi - 0’

The 0’ timepoint Figure S2 corresponds to frame 1 (timestamp 0’00”) of Appendix Movie S37.

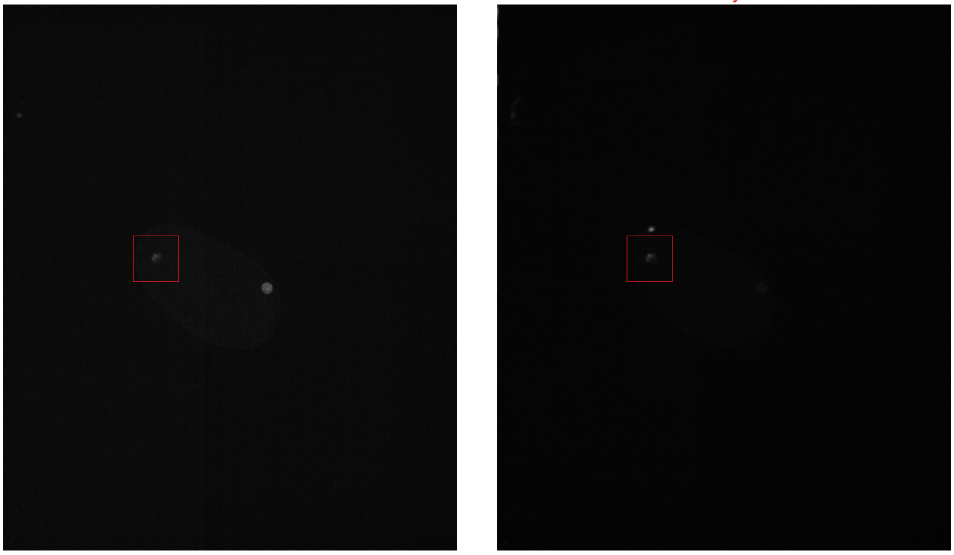

Figure S2 - *sld-2* + *cdc-45* RNAi - 0’

This image is rotated by 90° in Appendix Movie S38.

The 0’ timepoint Figure S2 corresponds to frame 8 (timestamp 1’10”) of Appendix Movie S38.

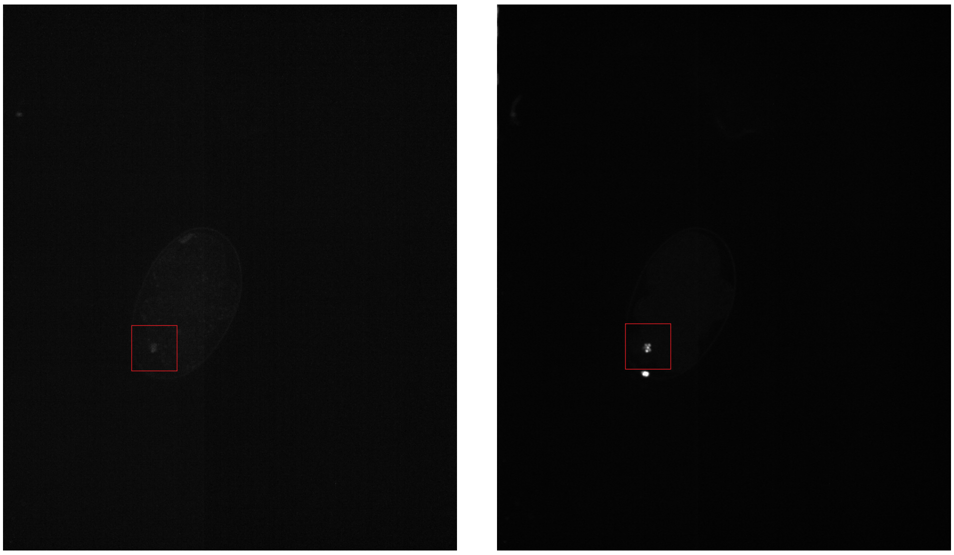

Supplement: Supplementary file 52 [file 44318_2026_915_MOESM56_ESM.zip › Source Data for Expanded View and Appendix/Appendix Figure S2/READ ME.pdf]
