# Supplementary material for: MCM10 and SLD-2/RECQL4 jointly activate the CMG helicase during metazoan DNA replication initiation
Source: EMBO J. 2026 Sep 28;45(21):7582–620. doi: 10.1038/s44318-026-00915-9 (PMC13728148; doi:10.1038/s44318-026-00915-9)
Supplement: Supplementary file 52 [file 44318_2026_915_MOESM56_ESM.zip › Source Data for Expanded View and Appendix/Appendix Figure S7/S7B/Appendix Figure S7B_agarose gel PCRs_panels left top and middle.pdf]

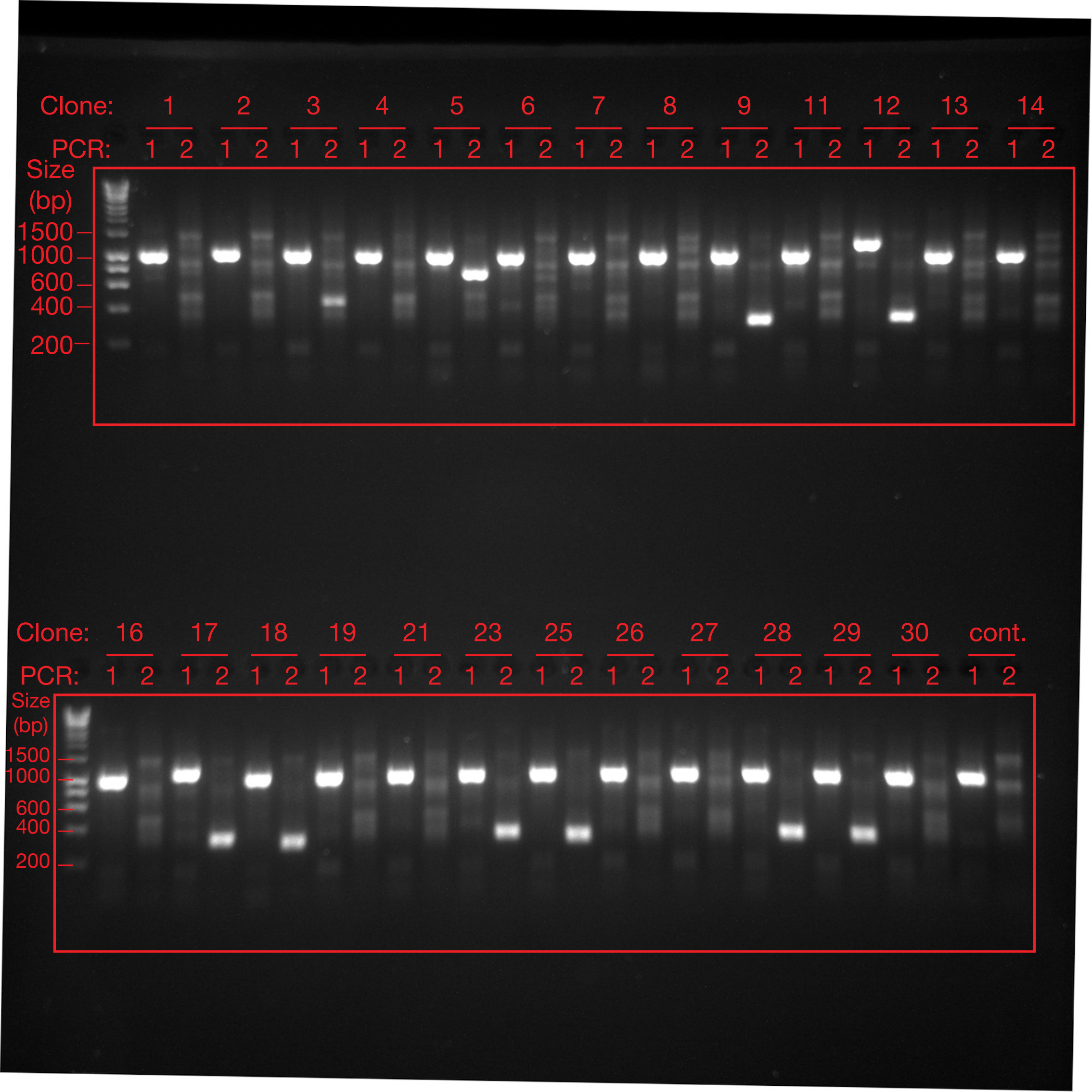
Clone: 1 2 3 4 5 6 7 8 9 11 12 13 14
PCR: 1 2 1 2 1 2 1 2 1 2 1 2 1 2 1 2 1 2 1 2 1 2 1 2 1 2
Size (bp)
1500
1000
600
400
200
Clone: 16 17 18 19 21 23 25 26 27 28 29 30 cont.
PCR: 1 2 1 2 1 2 1 2 1 2 1 2 1 2 1 2 1 2 1 2 1 2 1 2 1 2
Size (bp)
1500
1000
600
400
200
