# Supplementary material for: MCM10 and SLD-2/RECQL4 jointly activate the CMG helicase during metazoan DNA replication initiation
Source: EMBO J. 2026 Sep 28;45(21):7582–620. doi: 10.1038/s44318-026-00915-9 (PMC13728148; doi:10.1038/s44318-026-00915-9)
Supplement: Supplementary file 52 [file 44318_2026_915_MOESM56_ESM.zip › Source Data for Expanded View and Appendix/Appendix Figure S7/S7B/Appendix Figure S7B_agarose gel PCRs_panel right middle.pdf]

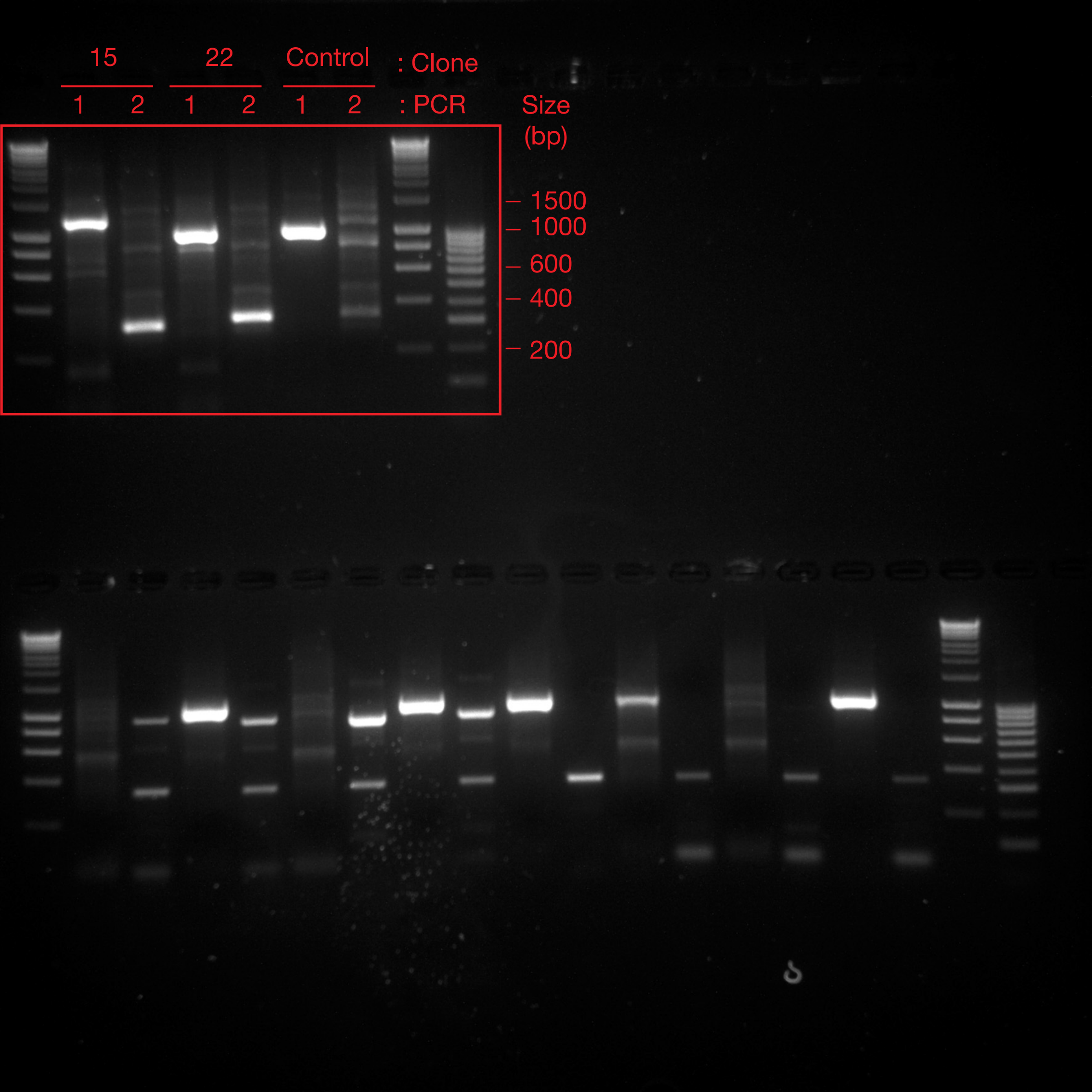
15
22
Control
: Clone
1
2
1
2
1
2
: PCR
Size
(bp)
1500
1000
600
400
200
