# Supplementary material for: MCM10 and SLD-2/RECQL4 jointly activate the CMG helicase during metazoan DNA replication initiation
Source: EMBO J. 2026 Sep 28;45(21):7582–620. doi: 10.1038/s44318-026-00915-9 (PMC13728148; doi:10.1038/s44318-026-00915-9)
Supplement: Supplementary file 52 [file 44318_2026_915_MOESM56_ESM.zip › Source Data for Expanded View and Appendix/Appendix Figure S7/S7B/Appendix Figure S7B_agarose gel PCRs_panels right and left bottom.pdf]

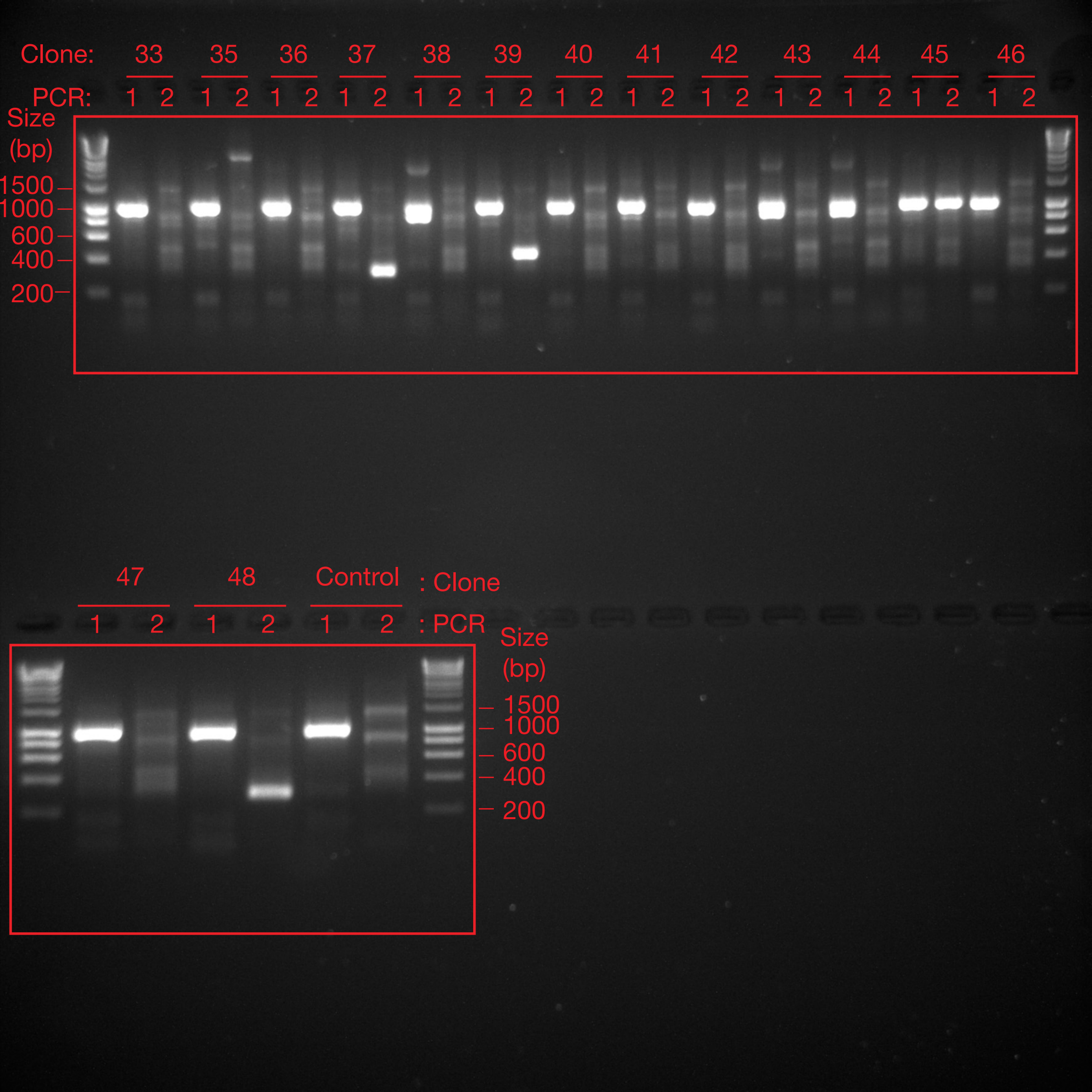

Clone: 33 35 36 37 38 39 40 41 42 43 44 45 46
PCR: 1 2 1 2 1 2 1 2 1 2 1 2 1 2 1 2 1 2 1 2 1 2 1 2 1 2
Size (bp)
1500
1000
600
400
200
47 48 Control : Clone
1 2 1 2 1 2 : PCR
Size (bp)
1500
1000
600
400
200
