# Supplementary material for: MCM10 and SLD-2/RECQL4 jointly activate the CMG helicase during metazoan DNA replication initiation
Source: EMBO J. 2026 Sep 28;45(21):7582–620. doi: 10.1038/s44318-026-00915-9 (PMC13728148; doi:10.1038/s44318-026-00915-9)
Supplement: Supplementary file 52 [file 44318_2026_915_MOESM56_ESM.zip › Source Data for Expanded View and Appendix/Appendix Figure S6/S6D/Appendix Figure S6D_blots for MCM10 and Psf3.pdf]

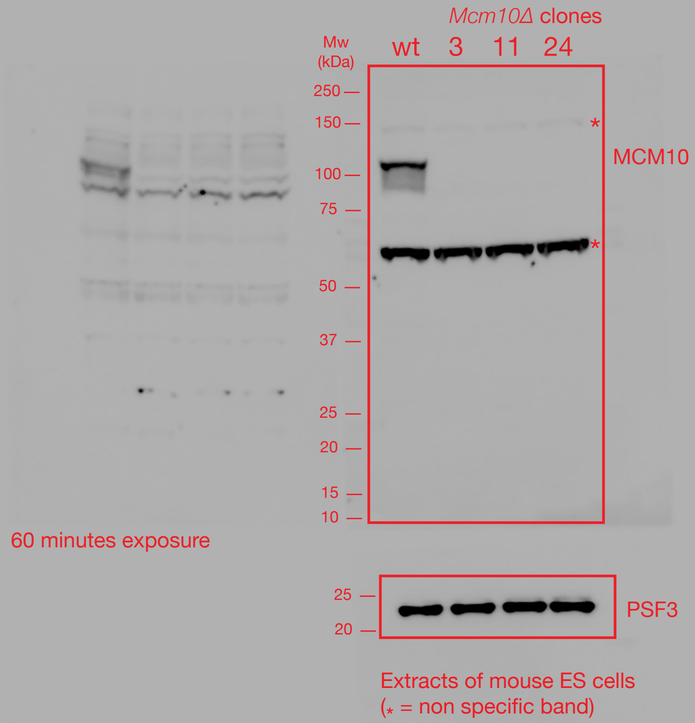
Mcm10Δ clones
Mw (kDa)
wt
3
11
24
250
150
100
75
50
37
25
20
15
10
*
*
MCM10
60 minutes exposure
25
20
PSF3
Extracts of mouse ES cells
(* = non specific band)
