# Supplementary material for: MCM10 and SLD-2/RECQL4 jointly activate the CMG helicase during metazoan DNA replication initiation
Source: EMBO J. 2026 Sep 28;45(21):7582–620. doi: 10.1038/s44318-026-00915-9 (PMC13728148; doi:10.1038/s44318-026-00915-9)
Supplement: Supplementary file 52 [file 44318_2026_915_MOESM56_ESM.zip › Source Data for Expanded View and Appendix/Appendix Figure S6/S6C/Appendix Figure S6C_agarose gel PCRs.pdf]

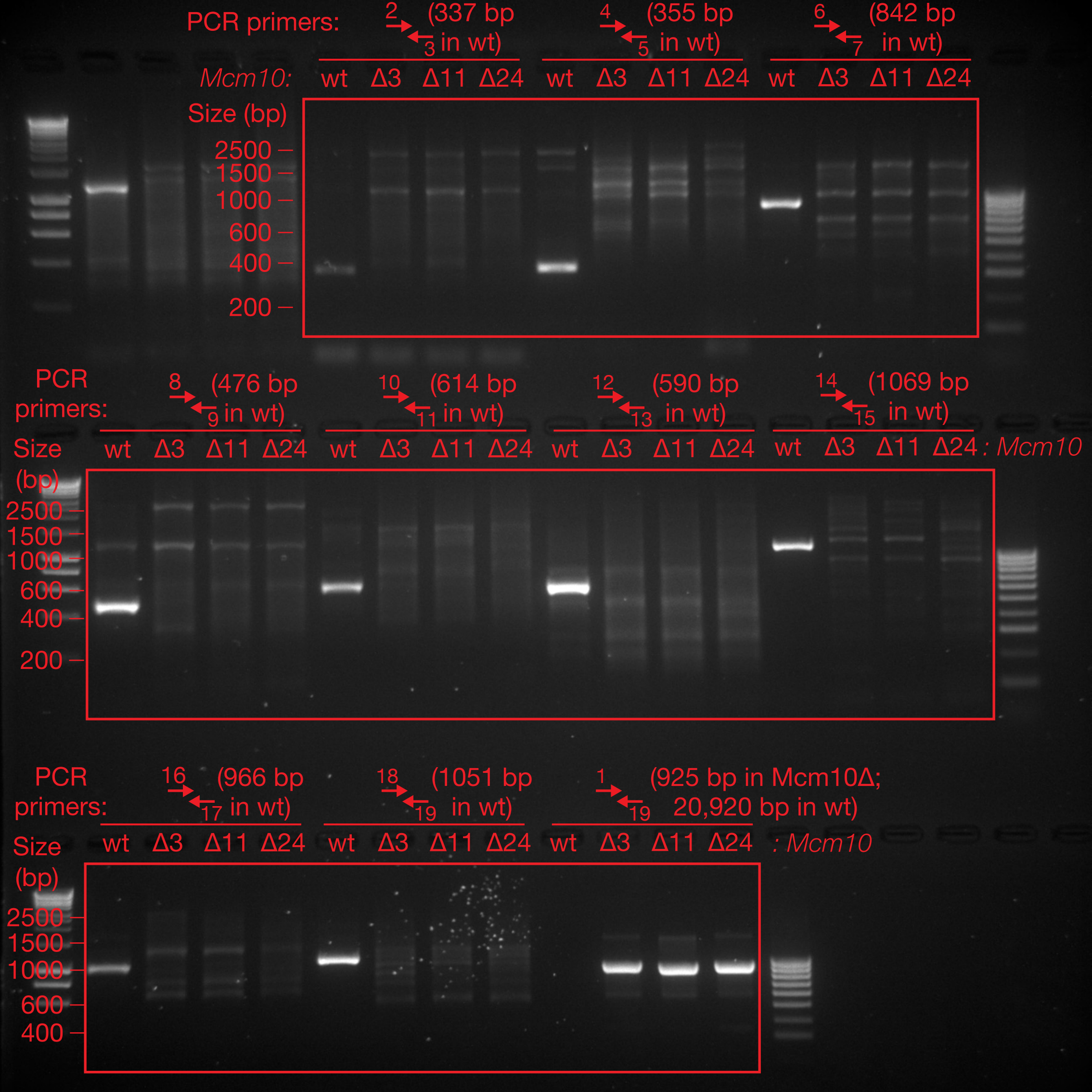

PCR primers:
2 3 (337 bp in wt)
4 5 (355 bp in wt)
6 7 (842 bp in wt)
Mcm10: wt Δ3 Δ11 Δ24 wt Δ3 Δ11 Δ24 wt Δ3 Δ11 Δ24
Size (bp)
2500
1500
1000
600
400
200
PCR primers:
8 9 (476 bp in wt)
10 11 (614 bp in wt)
12 13 (590 bp in wt)
14 15 (1069 bp in wt)
Size (bp)
wt Δ3 Δ11 Δ24 wt Δ3 Δ11 Δ24 wt Δ3 Δ11 Δ24 wt Δ3 Δ11 Δ24 : Mcm10
2500
1500
1000
600
400
200
PCR primers:
16 17 (966 bp in wt)
18 19 (1051 bp in wt)
1 19 (925 bp in Mcm10Δ; 20,920 bp in wt)
Size (bp)
wt Δ3 Δ11 Δ24 wt Δ3 Δ11 Δ24 wt Δ3 Δ11 Δ24 : Mcm10
2500
1500
1000
600
400
