# Supplementary material for: MCM10 and SLD-2/RECQL4 jointly activate the CMG helicase during metazoan DNA replication initiation
Source: EMBO J. 2026 Sep 28;45(21):7582–620. doi: 10.1038/s44318-026-00915-9 (PMC13728148; doi:10.1038/s44318-026-00915-9)

Figure S1A - *Control* - Prophase
The first Prophase timepoint in Figure S1A corresponds to frame 59 (timestamp 9’30”) of Appendix Movie S33.

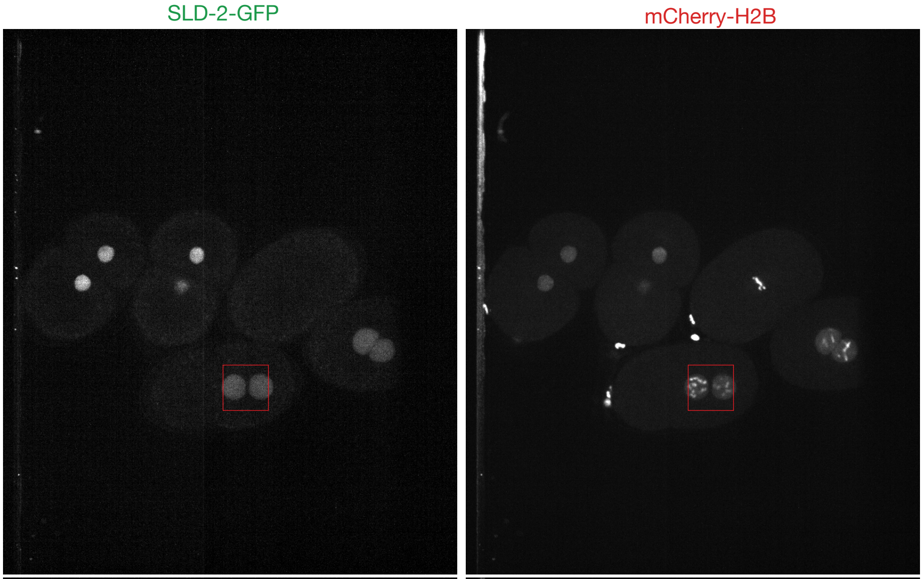

Figure S1A - *mcm-10*Δ - Prophase
This image is rotated by 270° in Figure S1A and in Appendix Movie S34.
The first Prophase timepoint in Figure S1A corresponds to frame 45 (timestamp 7’10”) of Appendix Movie S34.

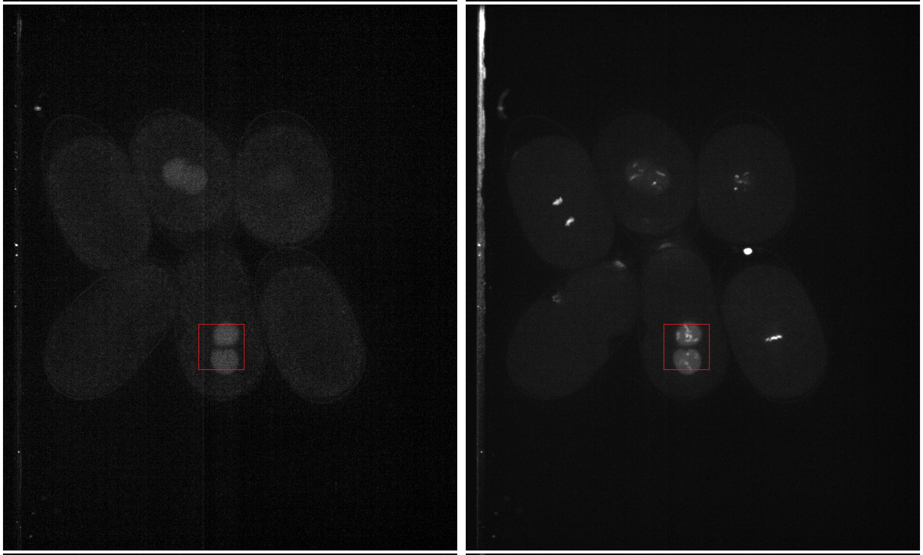

Figure S1A - *GFP* RNAi - Prophase
This image is rotated by 90° in Figure S1A and in Appendix Movie S35.
The first Prophase timepoint in Figure S1A corresponds to frame 22 (timestamp 3’20”) of Appendix Movie S35.

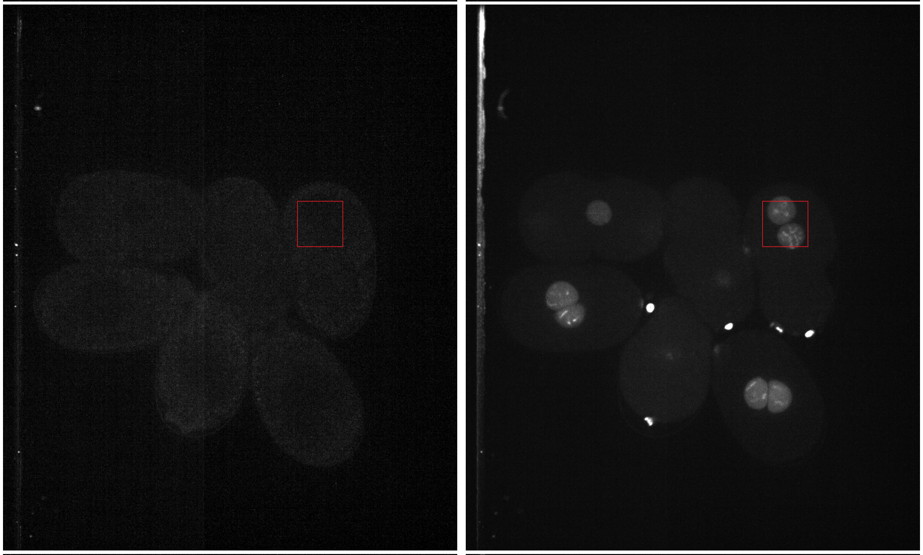

Figure S1A - *mcm-10*Δ + *GFP* RNAi - Prophase
This image is rotated by 270° in Figure S1A and in Appendix Movie S36.
The first Prophase timepoint in Figure S1A corresponds to frame 54 (timestamp 8’40”) of Appendix Movie S36.

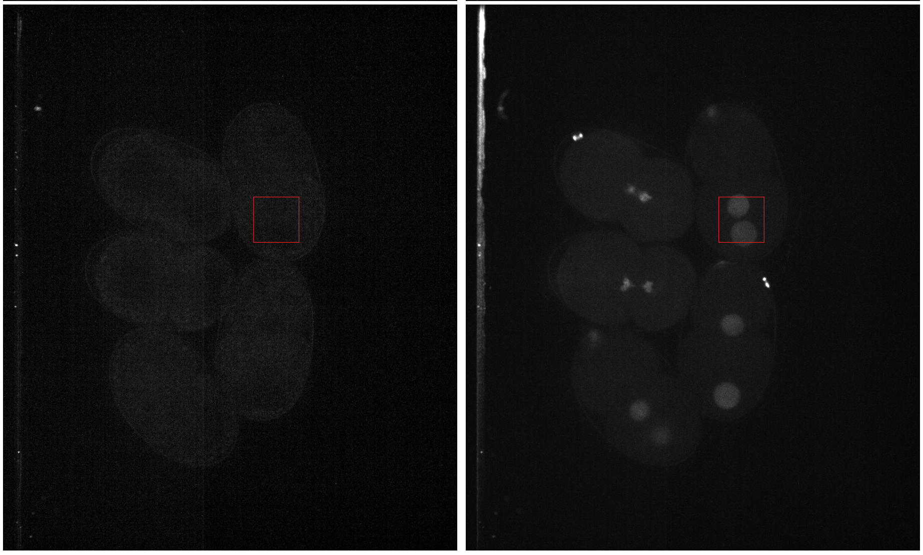

Supplement: Supplementary file 52 [file 44318_2026_915_MOESM56_ESM.zip › Source Data for Expanded View and Appendix/Appendix Figure S1/S1A/READ ME.pdf]
