# Supplementary material for: MCM10 and SLD-2/RECQL4 jointly activate the CMG helicase during metazoan DNA replication initiation
Source: EMBO J. 2026 Sep 28;45(21):7582–620. doi: 10.1038/s44318-026-00915-9 (PMC13728148; doi:10.1038/s44318-026-00915-9)
Supplement: Supplementary file 52 [file 44318_2026_915_MOESM56_ESM.zip › Source Data for Expanded View and Appendix/Appendix Figure S1/S1C/READ ME.pdf]

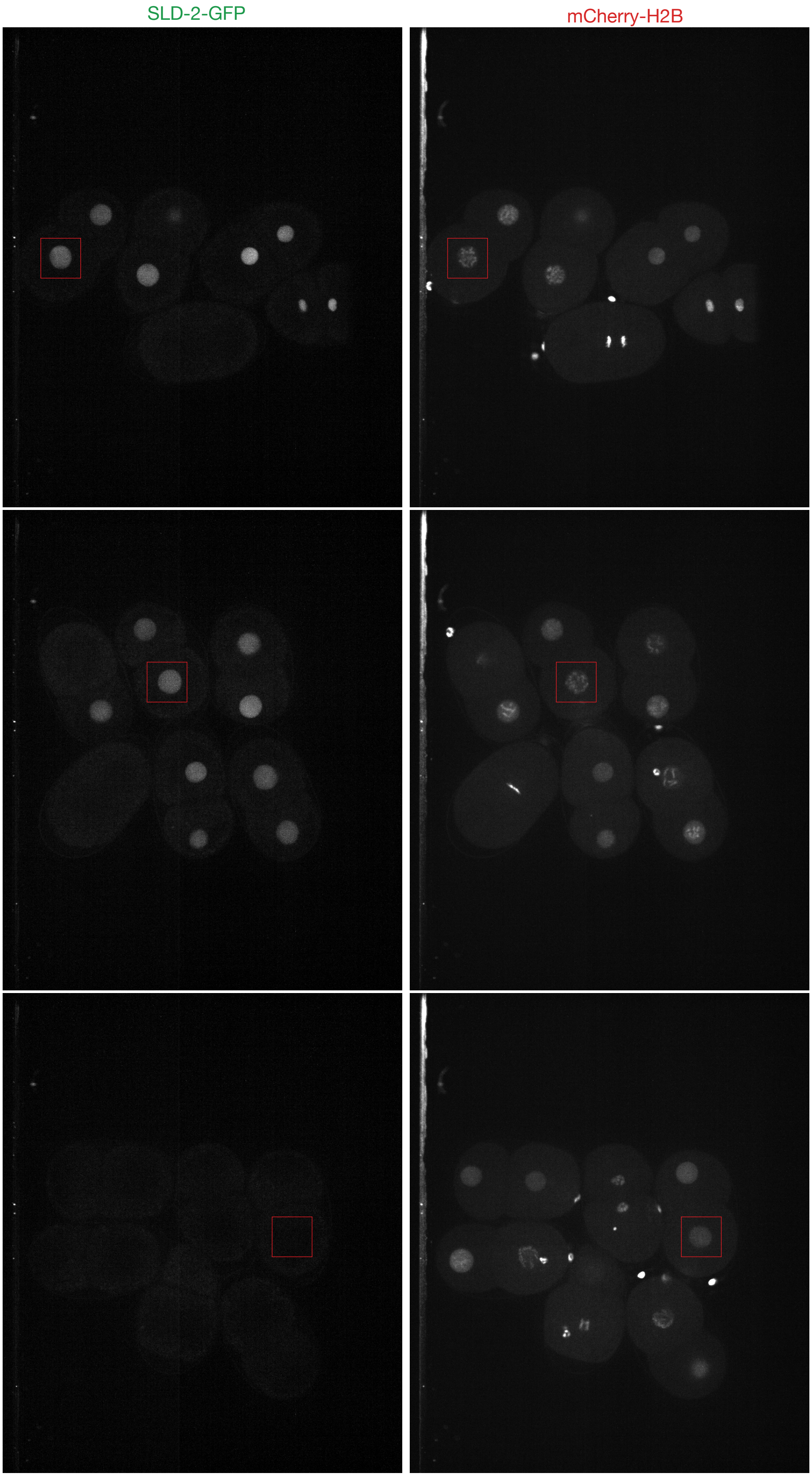

Figure S1C - *Control* - Prophase
The first Prophase timepoint in Figure S1C corresponds to frame 91 (timestamp 15’00”) of Appendix Movie S44.

Figure S1C - *mcm-10*∆ - Prophase
This image is rotated by 90° in Appendix Movie S45.
The first Prophase timepoint in Figure S1C corresponds to frame 116 (timestamp 19’10”) of Appendix Movie S45.

Figure S1C - *GFP* RNAi - Prophase
This image is rotated by 90° in Figure S1C and in Appendix Movie S43.
The first Prophase timepoint in Figure S1C corresponds to frame 114 (timestamp 18’50”) of Appendix Movie S43.
