# Supplementary material for: MCM10 and SLD-2/RECQL4 jointly activate the CMG helicase during metazoan DNA replication initiation
Source: EMBO J. 2026 Sep 28;45(21):7582–620. doi: 10.1038/s44318-026-00915-9 (PMC13728148; doi:10.1038/s44318-026-00915-9)

SLD-2-GFP mCherry-H2B

Figure S1B - *Control* - Prophase
The first Prophase timepoint in Figure S1B corresponds to frame 128 (timestamp 21’10”) of Appendix Movie S41.

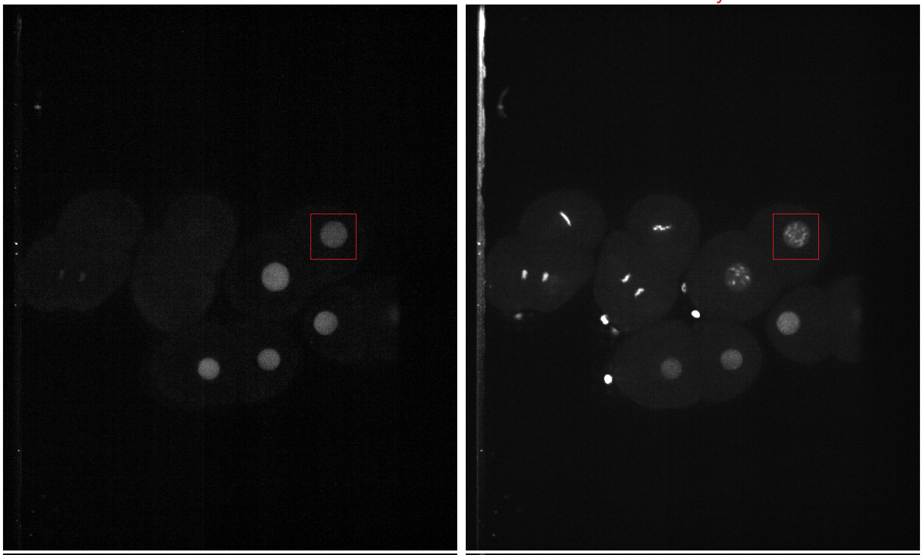

Figure S1B - *mcm-10*Δ - Prophase
This image is rotated by 90° in Figure S1B and by 270° in Appendix Movie S42.
The first Prophase timepoint in Figure S1B corresponds to frame 109 (timestamp 18’00”) of Appendix Movie S42.

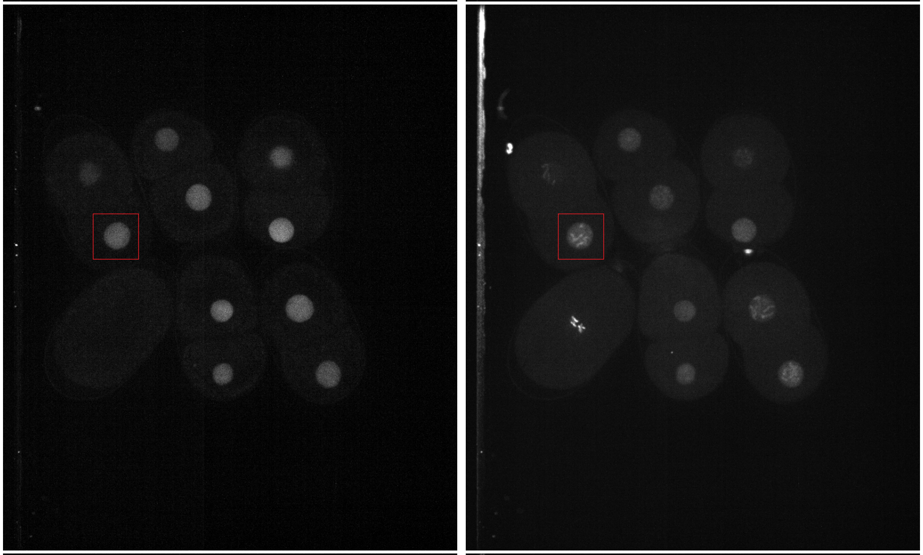

Figure S1B - *GFP* RNAi - Prophase
This image is rotated by 90° in Figure S1B and in Appendix Movie S43.
The first Prophase timepoint in Figure S1B corresponds to frame 140 (timestamp 23’10”) of Appendix Movie S43.

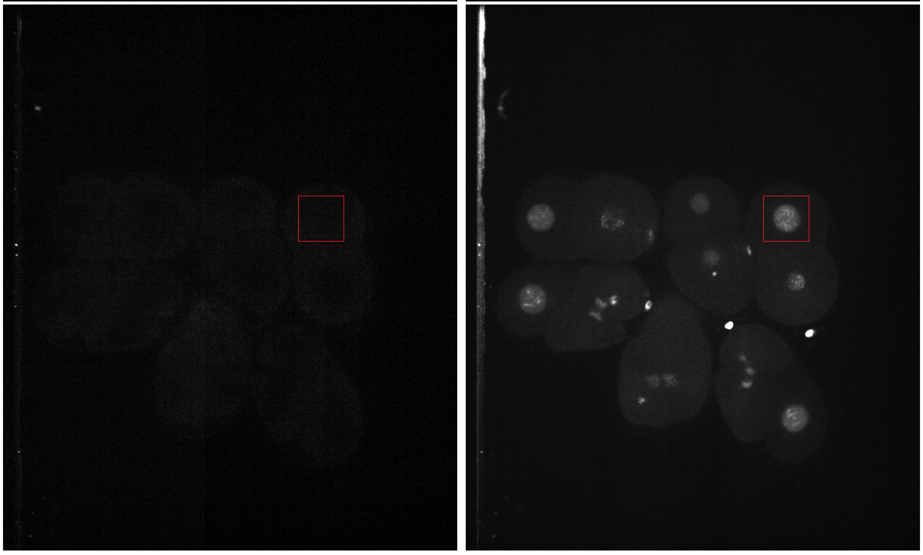

Supplement: Supplementary file 52 [file 44318_2026_915_MOESM56_ESM.zip › Source Data for Expanded View and Appendix/Appendix Figure S1/S1B/READ ME.pdf]
