# Supplementary material for: MCM10 and SLD-2/RECQL4 jointly activate the CMG helicase during metazoan DNA replication initiation
Source: EMBO J. 2026 Sep 28;45(21):7582–620. doi: 10.1038/s44318-026-00915-9 (PMC13728148; doi:10.1038/s44318-026-00915-9)
Supplement: Supplementary file 52 [file 44318_2026_915_MOESM56_ESM.zip › Source Data for Expanded View and Appendix/Appendix Figure S11/panel_D/README.pdf]

Each row corresponds to measurements from a single experimental well on the 96-well plate. Mean fluorescence intensity for each well was calculated using ScanR analysis software. Measurements were obtained using the Well Results feature with Nuclei selected as the reference gate. The columns are as follows:

Well: Identifier of the well on the 96-well plate.

Mean intensity EdU per nucleus per well: Mean EdU fluorescence intensity per nucleus calculated for all nuclei within a single well. This is the primary measurement obtained from ScanR analysis software.

Cell line: Name or identifier of the cell line used in the experiment.

EdU added: Whether EdU was added to the well or whether it was untreated with EdU to be used as threshold for discriminating EdU positive from negative cells.

('PROTACs 14h + 2h' = 16h + 250 nM PROTACs; 'PROTACs 14h + HU 2h' = '14h +250 nM PROTACs +2h (250nM PROTACs+ 2mM HU)).

The data shown in this figure are from one representative independent experiment. However, treatment with the PROTAC followed by HU was also performed in additional independent experiments, yielding a similar trend. In those experiments, the PROTAC was administered 4, 8, or 16 hours before the addition of HU.
