# Supplementary material for: MCM10 and SLD-2/RECQL4 jointly activate the CMG helicase during metazoan DNA replication initiation
Source: EMBO J. 2026 Sep 28;45(21):7582–620. doi: 10.1038/s44318-026-00915-9 (PMC13728148; doi:10.1038/s44318-026-00915-9)
Supplement: Supplementary file 52 [file 44318_2026_915_MOESM56_ESM.zip › Source Data for Expanded View and Appendix/Appendix Figure S11/panel_E/README.pdf]

Each row corresponds to measurements from a single experimental well on the 96-well plate. Mean fluorescence intensity for each well was calculated using ScanR analysis software. Measurements were obtained using the Well Results feature with Nuclei selected as the reference gate. The columns are as follows:

Well: Identifier of the well on the 96-well plate.

Mean intensity RPA32 per nucleus per well: Mean RPA32 fluorescence intensity per nucleus calculated for all nuclei within a single well. This is the primary measurement obtained from ScanR analysis software.

Cell line: Name or identifier of the cell line used in the experiment (B-M10; d-R4 = *BromoTag-Mcm10 dTag-Recql4*).

antibody: the antibody used.

PROTACS: Whether PROTACS were added for 16 hours.

HU: Whether 2mM HU was added to the cells in this well or not (if in combination with PROTACs, then was for last two-hours of the 16h treatment with PROTACs).

The data shown in this figure are from one representative independent experiment. However, treatment with the PROTAC followed by HU was also performed in additional independent experiments, yielding a similar trend. In those experiments, the PROTAC was administered 4, 8, or 16 hours before the addition of HU.
