# Supplementary material for: MCM10 and SLD-2/RECQL4 jointly activate the CMG helicase during metazoan DNA replication initiation
Source: EMBO J. 2026 Sep 28;45(21):7582–620. doi: 10.1038/s44318-026-00915-9 (PMC13728148; doi:10.1038/s44318-026-00915-9)
Supplement: Supplementary file 52 [file 44318_2026_915_MOESM56_ESM.zip › Source Data for Expanded View and Appendix/Figure EV4/EV4E-F/Figure EV4_CMG DNA binding assay replicate 3.pdf]

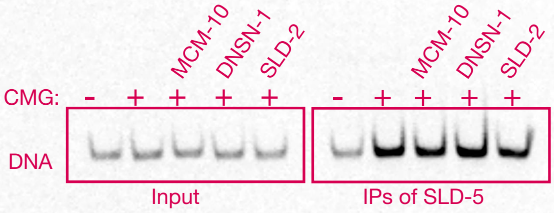
MCM-10
DNSN-1
SLD-2
MCM-10
DNSN-1
SLD-2
CMG: - + + + + - + + + +
DNA
Input
IPs of SLD-5
