# Supplementary material for: MCM10 and SLD-2/RECQL4 jointly activate the CMG helicase during metazoan DNA replication initiation
Source: EMBO J. 2026 Sep 28;45(21):7582–620. doi: 10.1038/s44318-026-00915-9 (PMC13728148; doi:10.1038/s44318-026-00915-9)
Supplement: Supplementary file 52 [file 44318_2026_915_MOESM56_ESM.zip › Source Data for Expanded View and Appendix/Figure EV3/EV3A/Immunoblots for MCM-2_SLD-5_DNSN-1.pdf]

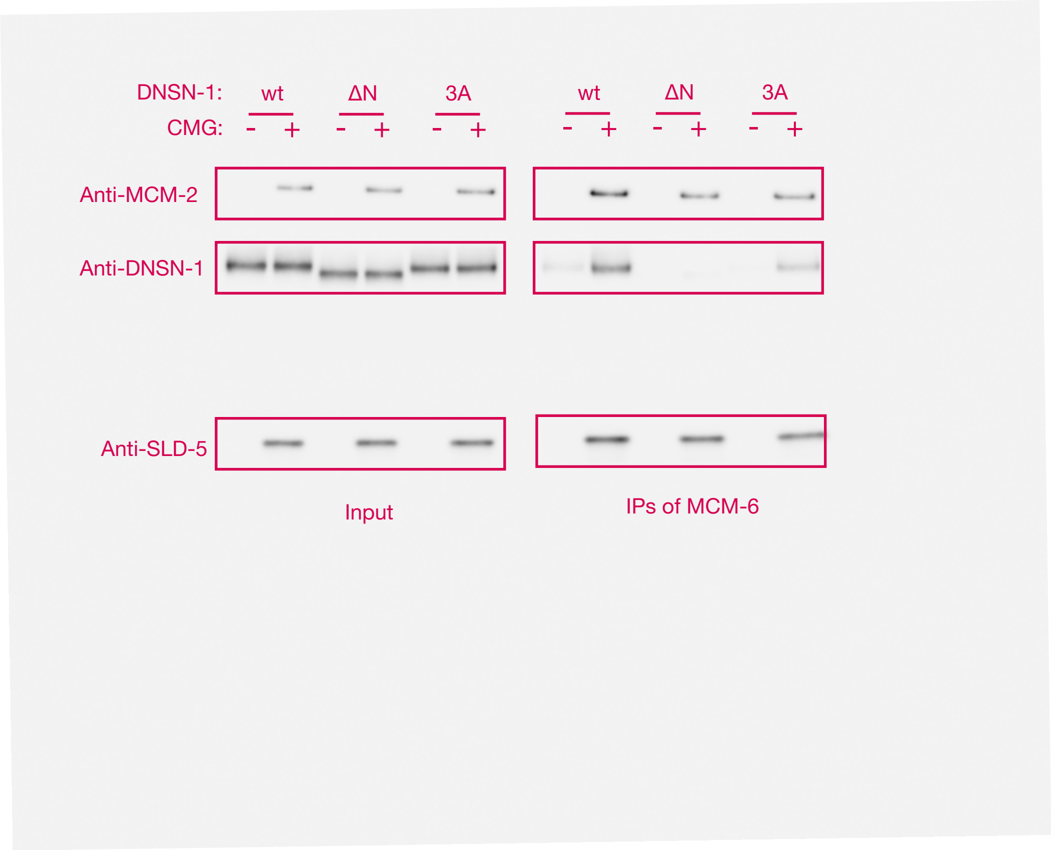
DNSN-1:
wt
ΔN
3A
wt
ΔN
3A
CMG:
- + - + - + - + - + - +
Anti-MCM-2
Anti-DNSN-1
Anti-SLD-5
Input
IPs of MCM-6
