# Supplementary material for: MCM10 and SLD-2/RECQL4 jointly activate the CMG helicase during metazoan DNA replication initiation
Source: EMBO J. 2026 Sep 28;45(21):7582–620. doi: 10.1038/s44318-026-00915-9 (PMC13728148; doi:10.1038/s44318-026-00915-9)
Supplement: Supplementary file 52 [file 44318_2026_915_MOESM56_ESM.zip › Source Data for Expanded View and Appendix/Figure EV3/EV3B/Figure EV3B - CMG helicase assay with DNSN-1 variants.pdf]

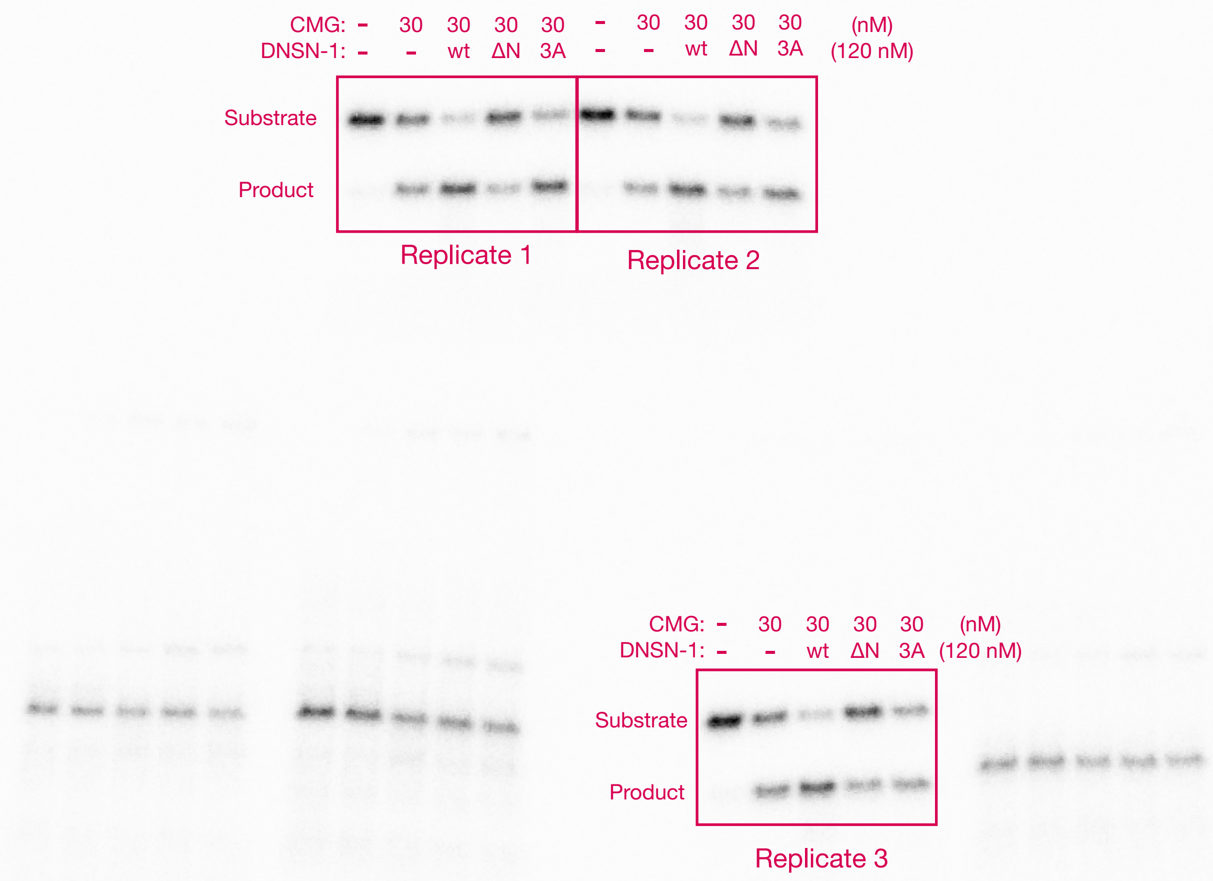

CMG: - 30 30 30 30 - 30 30 30 30 (nM)
DNSN-1: - - wt ΔN 3A - - wt ΔN 3A (120 nM)
Substrate
Product
Replicate 1
Replicate 2
CMG: - 30 30 30 30 (nM)
DNSN-1: - - wt ΔN 3A (120 nM)
Substrate
Product
Replicate 3
