# Supplementary material for: MCM10 and SLD-2/RECQL4 jointly activate the CMG helicase during metazoan DNA replication initiation
Source: EMBO J. 2026 Sep 28;45(21):7582–620. doi: 10.1038/s44318-026-00915-9 (PMC13728148; doi:10.1038/s44318-026-00915-9)
Supplement: Supplementary file 52 [file 44318_2026_915_MOESM56_ESM.zip › Source Data for Expanded View and Appendix/Appendix Figure S4/S4C/Figure S4C_Helicase assays with two 3' flaps.pdf]

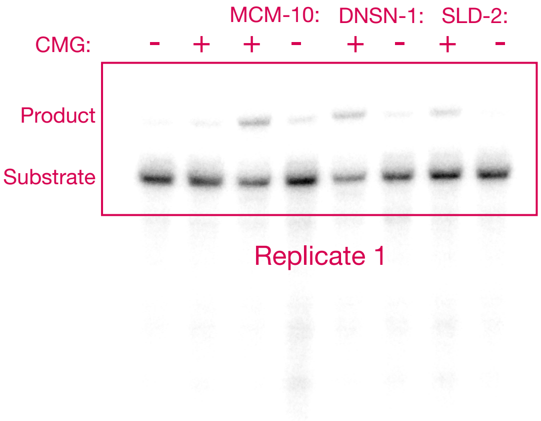

Replicate 1

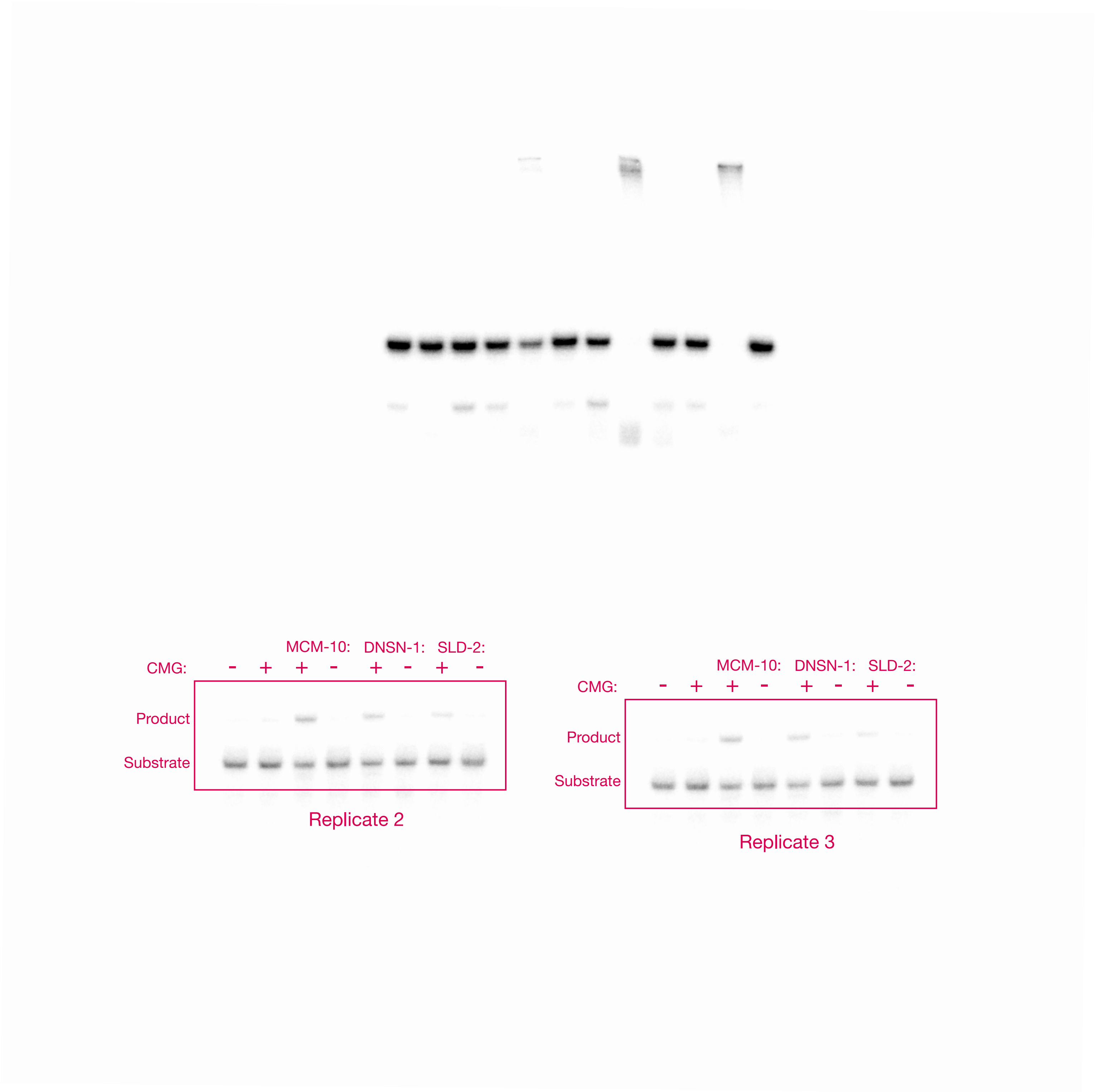
MCM-10: DNSN-1: SLD-2:
CMG: - + + - + - + -
Product
Substrate
Replicate 2
MCM-10: DNSN-1: SLD-2:
CMG: - + + - + - + -
Product
Substrate
Replicate 3
