# Supplementary material for: MCM10 and SLD-2/RECQL4 jointly activate the CMG helicase during metazoan DNA replication initiation
Source: EMBO J. 2026 Sep 28;45(21):7582–620. doi: 10.1038/s44318-026-00915-9 (PMC13728148; doi:10.1038/s44318-026-00915-9)
Supplement: Supplementary file 52 [file 44318_2026_915_MOESM56_ESM.zip › Source Data for Expanded View and Appendix/Appendix Figure S4/S4D/Figure S4D_Helicase assays with one 3' flap.pdf]

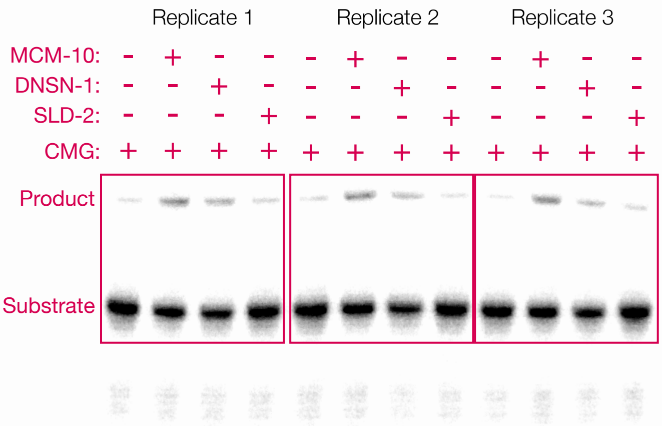

Replicate 1
Replicate 2
Replicate 3
MCM-10: - + - - - + - - - + - -
DNSN-1: - - + - - - + - - - + -
SLD-2: - - - + - - - + - - - +
CMG: + + + + + + + + + + + +
Product
Substrate
