# Supplementary material for: MCM10 and SLD-2/RECQL4 jointly activate the CMG helicase during metazoan DNA replication initiation
Source: EMBO J. 2026 Sep 28;45(21):7582–620. doi: 10.1038/s44318-026-00915-9 (PMC13728148; doi:10.1038/s44318-026-00915-9)
Supplement: Supplementary file 52 [file 44318_2026_915_MOESM56_ESM.zip › Source Data for Expanded View and Appendix/Appendix Figure S4/S4B/Figure S4B_Helicase assays with two 3' flaps.pdf]

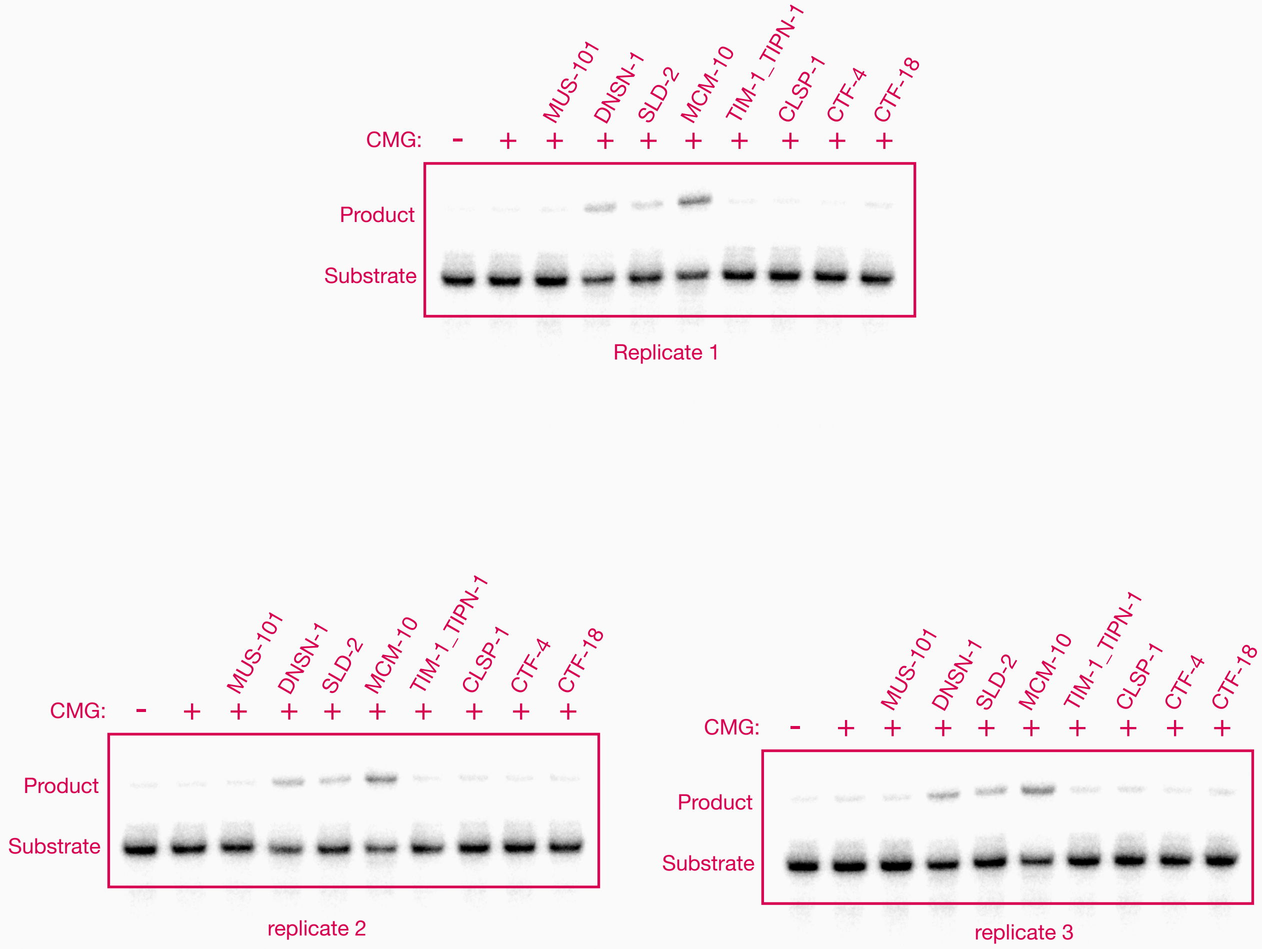
CMG: - + + + + + + + + +
MUS-101
DNSN-1
SLD-2
MCM-10
TIM-1_TIPN-1
CLSP-1
CTF-4
CTF-18
Product
Substrate
Replicate 1
CMG: - + + + + + + + + +
MUS-101
DNSN-1
SLD-2
MCM-10
TIM-1_TIPN-1
CLSP-1
CTF-4
CTF-18
Product
Substrate
replicate 2
CMG: - + + + + + + + + +
MUS-101
DNSN-1
SLD-2
MCM-10
TIM-1_TIPN-1
CLSP-1
CTF-4
CTF-18
Product
Substrate
replicate 3
