# Supplementary material for: MCM10 and SLD-2/RECQL4 jointly activate the CMG helicase during metazoan DNA replication initiation
Source: EMBO J. 2026 Sep 28;45(21):7582–620. doi: 10.1038/s44318-026-00915-9 (PMC13728148; doi:10.1038/s44318-026-00915-9)

Figure S3A - *rnr-1* RNAi - 0’

The 0’ timepoint Figure S3A corresponds to frame 21 (timestamp 3’10”) of Appendix Movie S39.

SLD-2-GFP mCherry-RPA-2

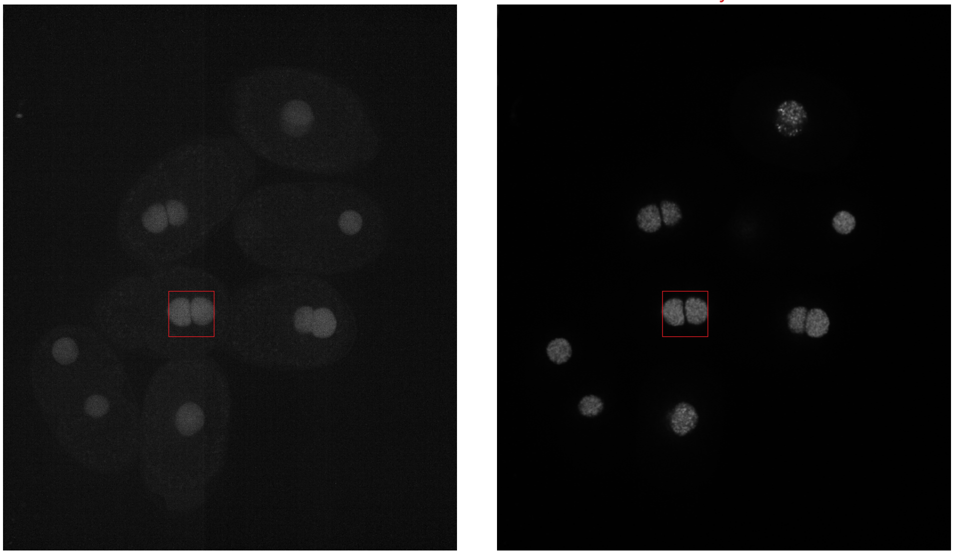

Figure S3A - *rnr-1* + *cdc-45* RNAi - 0’

The 0’ timepoint Figure S3A corresponds to frame 7 (timestamp 0’50”) of Appendix Movie S40.

SLD-2-GFP mCherry-RPA-2

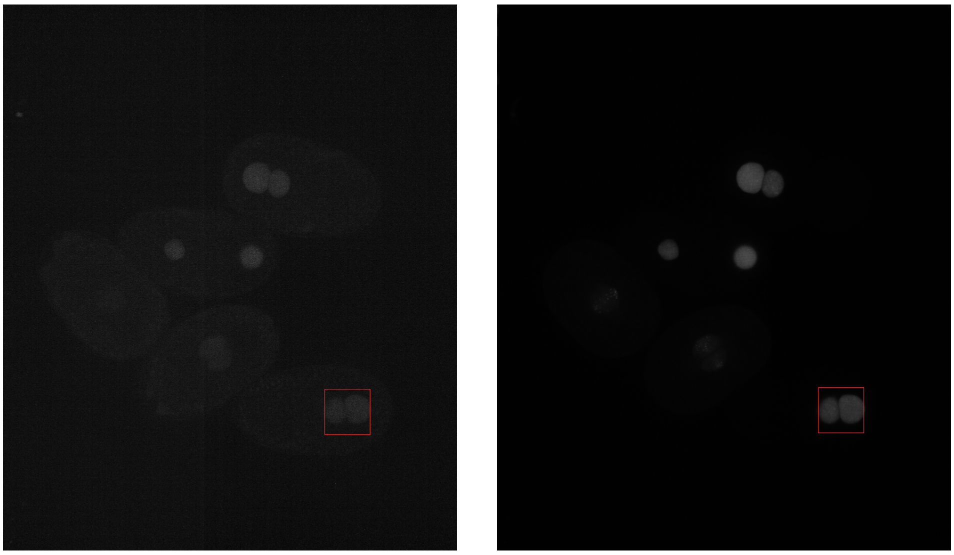

Supplement: Supplementary file 52 [file 44318_2026_915_MOESM56_ESM.zip › Source Data for Expanded View and Appendix/Appendix Figure S3/S3A/READ ME.pdf]
