# Supplementary material for: MCM10 and SLD-2/RECQL4 jointly activate the CMG helicase during metazoan DNA replication initiation
Source: EMBO J. 2026 Sep 28;45(21):7582–620. doi: 10.1038/s44318-026-00915-9 (PMC13728148; doi:10.1038/s44318-026-00915-9)
Supplement: Supplementary file 52 [file 44318_2026_915_MOESM56_ESM.zip › Source Data for Expanded View and Appendix/Appendix Figure S5/S5C/Appendix Figure S5C_Blot MCM10.pdf]

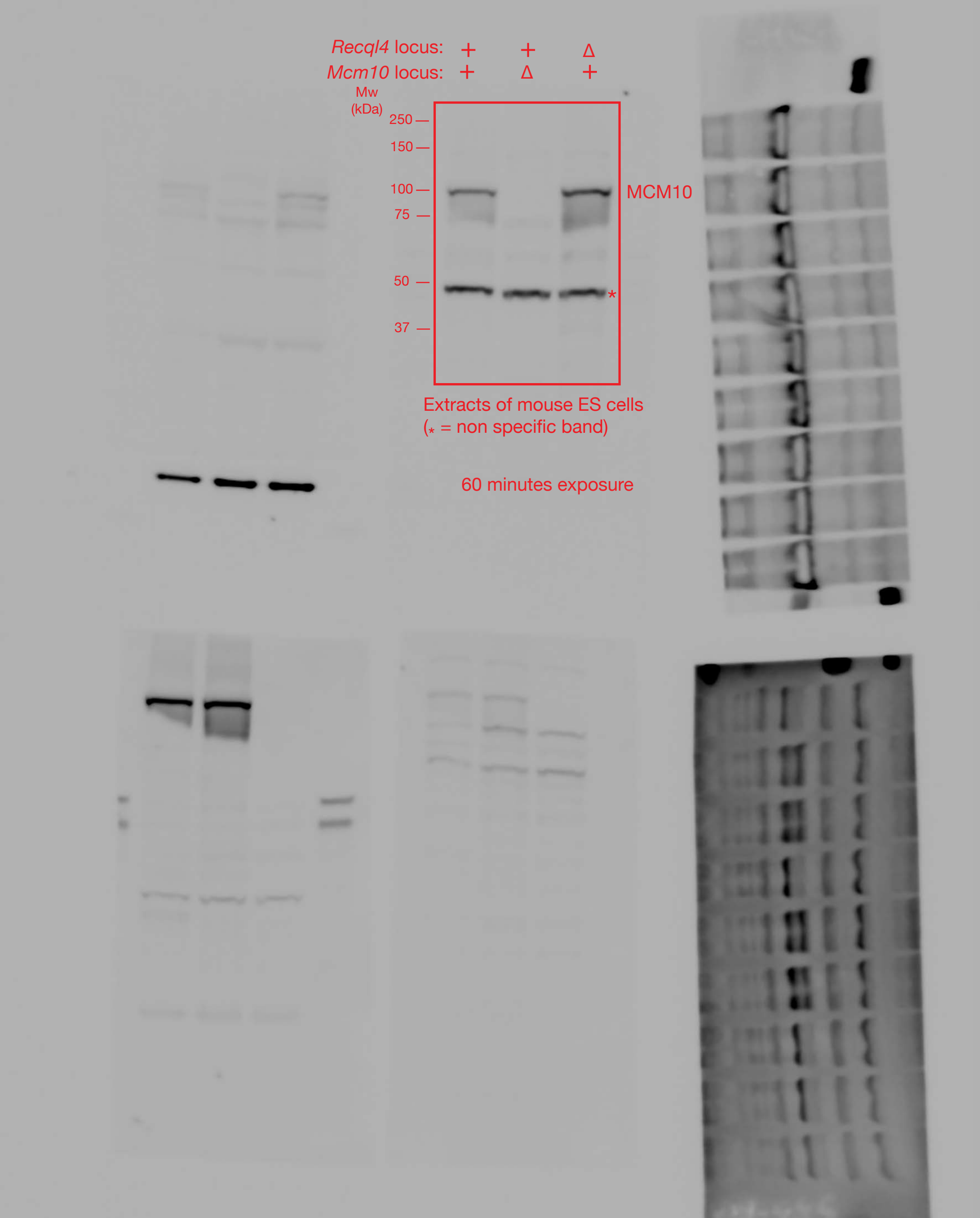
Recql4 locus: + + Δ
Mcm10 locus: + Δ +
Mw
(kDa)
250
150
100
75
50
37
MCM10
*
Extracts of mouse ES cells
(* = non specific band)
60 minutes exposure
