# Supplementary material for: MCM10 and SLD-2/RECQL4 jointly activate the CMG helicase during metazoan DNA replication initiation
Source: EMBO J. 2026 Sep 28;45(21):7582–620. doi: 10.1038/s44318-026-00915-9 (PMC13728148; doi:10.1038/s44318-026-00915-9)
Supplement: Supplementary file 52 [file 44318_2026_915_MOESM56_ESM.zip › Source Data for Expanded View and Appendix/Appendix Figure S12/panel_C/README.pdf]

This source data file contains the raw measurements used to generate the scatter plot shown in the main figure. Each row corresponds to a single nucleus.

The columns are:

Mean Intensity MCM2: Mean fluorescence intensity of the MCM2; Alexa546 signal for each nucleus (y-axis of the scatter plot).

Cell type and treatment: Experimental group corresponding to each nucleus. This column contains multiple conditions and can be filtered.

Total Intensity DAPI: Total fluorescence intensity of the DAPI signal for each nucleus (x-axis of the scatter plot).

EdU positive nucleus: A value of 0 in the row indicates a nucleus that was EdU negative and a 1 indicates an EdU positive nucleus.
