# Supplementary material for: MCM10 and SLD-2/RECQL4 jointly activate the CMG helicase during metazoan DNA replication initiation
Source: EMBO J. 2026 Sep 28;45(21):7582–620. doi: 10.1038/s44318-026-00915-9 (PMC13728148; doi:10.1038/s44318-026-00915-9)
Supplement: Supplementary file 52 [file 44318_2026_915_MOESM56_ESM.zip › Source Data for Expanded View and Appendix/Appendix Figure S12/panel_B/README.pdf]

Each row corresponds to measurements from a single experimental well on the 96-well plate. Mean fluorescence intensity for each well was calculated using ScanR analysis software. Measurements were obtained using the Well Results feature with Nuclei selected as the reference gate. The dataset includes raw measurements, plate-normalized values, and control-normalized values of EdU intensity. The columns are as follows:

Plate: Identifier of the experimental plate on which the sample was processed.

Mean EdU intensity per nucleus per well: Mean EdU fluorescence intensity per nucleus calculated for all nuclei within a single well. This is the primary measurement obtained from ScanR analysis software.

Cell line: Name or identifier of the cell line used in the experiment.

Wt mean: Average value of the control treatment used as the reference for treatment normalization, in this case Wild type mESCs.

Relative EdU intensity: EdU intensity normalized relative to the corresponding control treatment average. This value was used for the plots shown in the figure.
